# Effectiveness of integrated *Aedes albopictus* management in southern Switzerland

**DOI:** 10.1186/s13071-021-04903-2

**Published:** 2021-08-16

**Authors:** Damiana Ravasi, Diego Parrondo Monton, Matteo Tanadini, Eleonora Flacio

**Affiliations:** 1 Laboratory of Applied Microbiology, Department of Environment, Construction and Design, University of Applied Sciences and Arts of Southern Switzerland, via Mirasole 22A, 6500 Bellinzona, Switzerland; 2 Zurich Data Scientists GmbH, Sihlquai 131, 8005 Zurich, Switzerland

**Keywords:** *Aedes albopictus*, Integrated vector management, Ovitrap, Gravid *Aedes* trap, Surveillance, Control measures

## Abstract

**Background:**

The exotic invasive tiger mosquito, *Aedes albopictus*, appeared in southern Switzerland in 2003. The spread of the mosquito has been surveyed constantly since then, and an integrated vector management (IVM) has been implemented to control its numbers. The control measures focus on the aquatic phase of the mosquito with removal of breeding sites and applications of larvicides in public areas whereas private areas are reached through extensive public information campaigns. Here, we evaluated the efficacy of the IVM.

**Methods:**

Since all the municipalities with *Ae. albopictus* in southern Switzerland are currently implementing the IVM, Italian municipalities just across the Swiss-Italian border, where *Ae. albopictus* is present but no coordinated intervention programme is in place, served as control. Ovitraps and adult female traps were used to measure mosquito abundance in 2019. Generalised mixed-effects models were used to model the numbers of *Ae. albopictus* eggs and adult females collected. These numbers of *Ae. albopictus* eggs were compared to the numbers of eggs collected in 2012 and 2013 in a previous assessment of the IVM, using a hurdle model.

**Results:**

Mean numbers of *Ae. albopictus* eggs and adult females in 2019 were consistently higher in the municipalities not following an IVM programme. In these municipalities, there were about four times (3.8) more eggs than in the municipalities implementing an IVM programme. Also, the numbers of eggs and adult females increased steadily from the beginning of the *Ae. albopictus* reproductive season, reaching a peak in August. In contrast, the increase in numbers of *Ae. albopictus* was much more contained in the municipalities implementing an IVM programme, without reaching an evident peak. Comparison with data from 2012 and 2013 indicates that the gap between intervention and non-intervention areas may have almost doubled in the past 6 years.

**Conclusions:**

The results of the survey strongly support the efficacy of the IVM programme implemented in southern Switzerland compared to municipalities without defined control measures. With the constant implementation of an IVM, it appears possible to contain the numbers of *Ae. albopictus* at a manageable level, reducing the nuisance for the human population and the risk of arbovirus epidemics.

**Graphical Abstract:**

**Figure:**
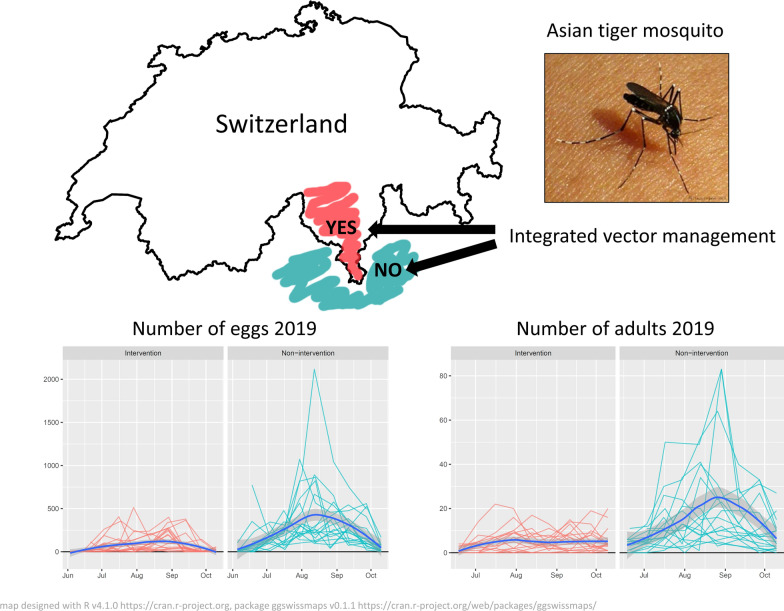

**Supplementary Information:**

The online version contains supplementary material available at 10.1186/s13071-021-04903-2.

## Background

*Aedes albopictus*, also known as the Asian tiger mosquito, is native to Southeast Asia and has been spreading globally in the last 40 years. This is probably due to both extrinsic factors, such as increase of global trade and travel, climate change and lack of efficient control, and intrinsic factors, such as strong physiological and ecological plasticity [1, 2]. Originally, a tree-hole breeding mosquito, *Ae. albopictus*, has managed to colonize suburban and urban settings, primarily due to its adaptation to using artificial containers (e.g. plant saucers, watering cans, plastic drums and catch basins) as breeding sites [1]. Since its first appearance in Italy in the 1990s, the species has expanded to most areas of the country [3] and has further spread to other European countries passively through the various human transportation networks (Mosquito Maps, http://ecdc.europa.eu/). In 2003, it appeared for the first time in the southernmost tip of Switzerland, in the Canton of Ticino (hereafter referred to as Ticino), at a service area on the European Route E35 close to the Italian border [4]. The spread of this vector in Ticino has actively been surveyed by the cantonal Working Group for Mosquitoes (Gruppo Lavoro Zanzare, GLZ) and control measures have been immediately implemented to prevent the establishment of the mosquito [5, 6]. Despite the containment measures, today the mosquito is established in most urban areas of Ticino.

The establishment of *Ae. albopictus* in suburban and urban areas represents a potential threat for public health because of its vectorial competence for at least 26 different arboviruses including dengue, chikungunya, Zika and yellow fever viruses [7]. In the last decade, *Aedes*-borne diseases have been increasing in Europe, with outbreaks of dengue and chikungunya in several countries [8]. In addition to the risk of virus transmission, the aggressive daytime biting behaviour of this mosquito causes major nuisance for people thus affecting their lifestyle, such as restricting outdoor activities [9]. Therefore, vector management becomes an important mechanism for disease prevention and nuisance reduction. Integrated vector management (IVM) is the approach recommended by the international health agencies and generally accepted [10–12]. It combines different intervention strategies, such as physical, chemical and biological control measures, aimed at reducing or eliminating the mosquito. Through a multi-sectoral approach, public health entities, other relevant agencies/organisations and the community are involved in the decision-making process aimed at optimizing the use of resources for vector control [12].

An IVM programme was implemented in 2000 in Ticino and gradually adapted during the years in accordance to the level of the spread of *Ae. albopictus* [5, 6], following the indications given in the ECDC guidelines for the surveillance of invasive mosquitoes [13]. Currently, more than 80 municipalities are involved in the programme, covering more than 90% of the total human population of Ticino. The surveillance is based on the detection and density estimation of *Ae. albopictus* with oviposition traps (ovitraps), the identification of breeding sites and the evaluation of reports from residents on the presence of the mosquito [5]. The control strategy consists of integrated measures to eliminate or reduce the densities of *Ae. albopictus* and is based on the collaboration among GLZ, municipal authorities, Civil Protection Units and citizens. The control measures focus on the aquatic phase of the mosquito and include the removal of breeding sites and the use in the public areas (mainly in catch basins) of larvicide applications scheduled weekly or monthly from May to October, depending on the biocide used. To reach private areas, an extensive information campaign is carried out every year, including community education through information events, door-to-door delivery of education material (leaflets), school education and use of mass media [5]. Citizens are strongly encouraged to remove temporary water containers from private properties and to cover or treat permanent water containers with obtainable *Bacillus thuringiensis* var. *israelensis* (Bti) granules (VectoBac® G, Valent Biosciences). The use of adulticides, less ecologically sustainable, is reserved for areas where an imported disease case is confirmed, to prevent autochthonous cases and outbreaks.

The monitoring and evaluation of the effectiveness of control methods are an essential part of the IVM. Equally important is the sharing of these evaluations, so that lessons can be learned and knowledge exchanged across countries [12]. The IVM programme adopted in Ticino was previously evaluated in 2012 and 2013 by comparing relative mosquito densities between Ticino and two neighbouring Italian provinces where ecological parameters are comparable but control measures were not carried out in a coordinated and comprehensive manner [14]. The seasonal and spatial abundance of *Ae. albopictus* in sylvatic and urban environments across the Swiss-Italian border was examined using ovitraps and a randomised sampling scheme. The results showed that egg data were useful to determine the efficacy of the intervention methods employed and that in the urban environments of the non-intervention area egg densities were 2.26 times higher compared to the intervention area. These findings showed that, although the spread of *Ae. albopictus* in Ticino could not be stopped, partly because of the continuous reintroduction of mosquitoes from Italy, the intervention programme avoided an explosive increase.

Here, we describe the results of the latest evaluation of the Ticino IVM programme, which was carried out in 2019, 6 years after the one effectuated by Suter and collaborators [14]. The design of the present study was based on the previous investigation. Since all the municipalities with *Ae. albopictus* in Ticino are currently implementing the IVM, we lacked control municipalities where the mosquito is present and no IVM programme is followed. As in Suter et al. [14], Italian municipalities just across the Swiss-Italian border, where *Ae. albopictus* is present but no coordinated intervention programme is in place, served as control. Indeed, the municipalities surveyed in the area across the Swiss-Italian border are very similar in many respects (e.g. history, climate, dimension and spatial structure) and geographically very close (located within a 7-km radius). Moreover, *Ae. albopictus* populations across the Swiss-Italian border have been shown to share a very similar genetic structure [15] due to the colonization process from North Italy to Switzerland. Ovitraps and Gravid *Aedes* Traps (GAT) were used as complementary approaches to measure indirectly and directly adult female mosquito abundance, respectively, and to determine the efficacy of the IVM programme in Ticino.

## Methods

### Study sites and design

**Fig. 1:**
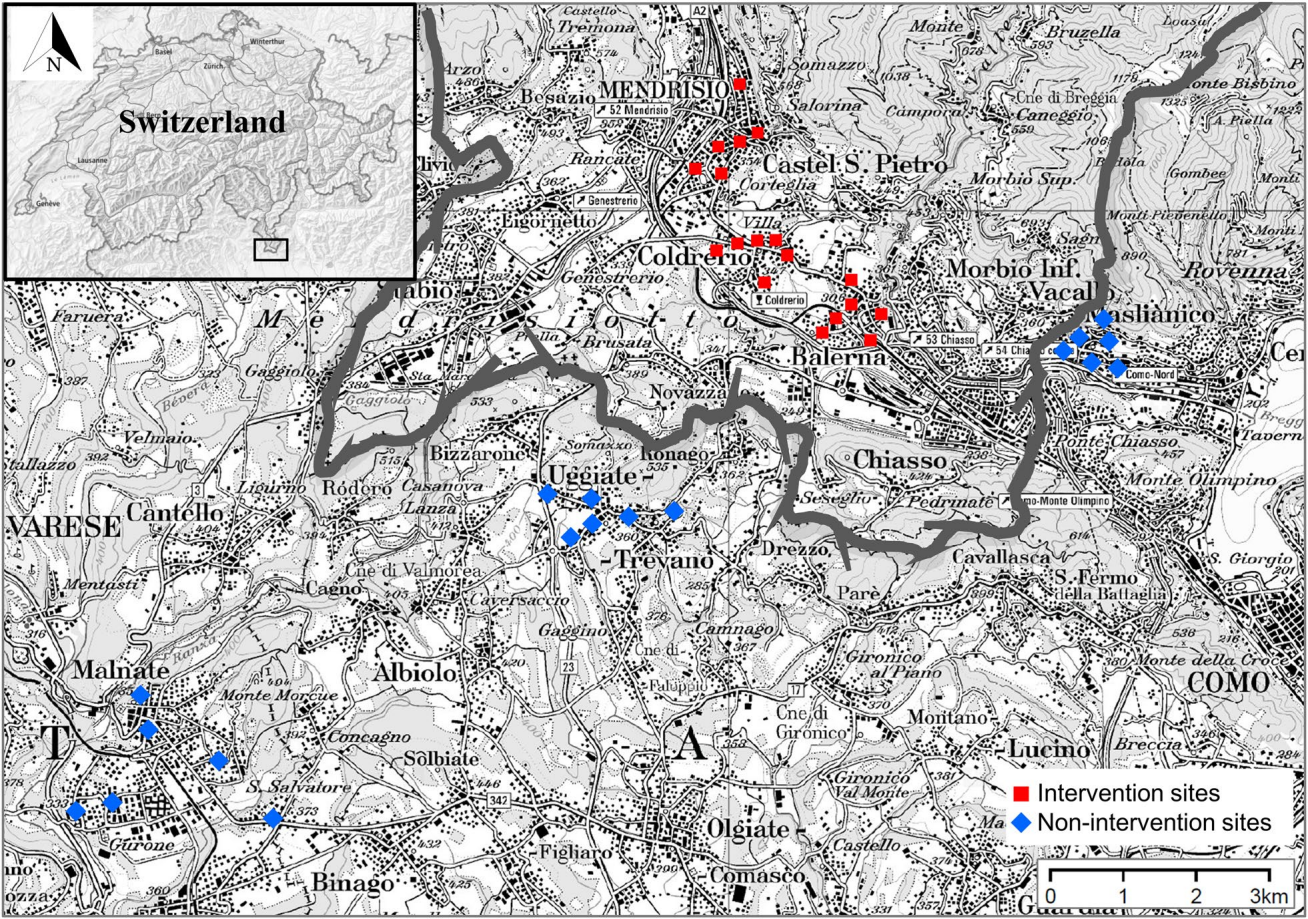
The six municipalities across the Swiss-Italian border, denoted by the thick dark grey line, surveyed in 2019. The red squares and blue diamonds represent sampling sites in intervention and non-intervention areas, respectively. Map modified from https://map.geoadmin.ch/

The field surveys were carried out in six small to medium-sized towns (3000 to 16,000 inhabitants) around the border area between Ticino in Switzerland and the Lombardy region in Italy (Fig. 1). The municipalities are located in the historical-geographical region of Insubria. The climate of this region is characterized by dry and sunny winters, with periods of foehn wind from the North with occasional heavy snowfall, rainfall, especially in the transitional seasons (spring and autumn), and sunny summers interrupted by downpours that can also be violent. The landscape of the region features foothills and typical components of Lombard agriculture next to residential, industrial and commercial urbanized areas. The particular geographical position of Insubria has been an incentive to build strong economic relations between Ticino and the neighbouring Italian provinces, resulting in intense traffic across the border, with on average over 67,900 Italian workers commuting to Switzerland in a single workday [16].

The six municipalities surveyed are located within a radius of 7 km, have similar dimension and urban structure, with a small-town centre surrounded by residential areas, and similar climatic (Additional file 1: Figure S1) and altitudinal characteristics (255–414 m a.s.l.). The three municipalities (i.e. Balerna, Coldrerio and Mendrisio) in the Mendrisiotto district in Ticino follow systematically the cantonal IVM since 2009 with monthly or weekly treatments of catch basins with diflubenzuron- or Bti-based products, respectively [5]. The treatment period starts at beginning mid-May, depending on the precipitation pattern, and lasts until mid-end September. In 2019, to encourage citizens to treat permanent breeding sites on private properties, Bti-based granules (VectoBac® G) were made freely available in each municipality. We categorized these three municipalities as “intervention” areas. The three municipalities in the provinces of Como (i.e. Maslianico and Uggiate-Trevano) and Varese (i.e. Malnate), in Lombardy, to our knowledge, did not follow an IVM and only applied adulticides irregularly. They were, therefore, categorized as “non-intervention” areas.

The territory of each municipality was divided into a grid of 250 × 250 m cells [5, 14]. Six cells, called sampling sites, were selected at random in urban context in each municipality. An ovitrap and a Gravid *Aedes* Trap (GAT, Biogents, Germany) were placed in each sampling site at a distance of 20–100 m from each other to avoid as much as possible interference in mosquito attraction on the ground at hidden, shaded, wind-protected locations close to vegetation. All traps were geo-referenced and uniquely labelled. The ovitraps were the same as used for the surveillance of *Ae. albopictus* in Ticino [5, 14]. Both ovitraps and GATs mimic breeding sites, attracting container-breeding mosquitoes in search of an oviposition site [13]. Ovitraps allow the invasive mosquitoes to deposit their eggs on a wooden slat and to fly away, while GATs capture the mosquitoes by means of an adhesive plastic sheet [17].

### Sample collection and processing

Field surveys were carried out from mid-end May (calendar week 21) to the beginning of October (calendar week 41) 2019. The slats of ovitraps and the sheets of the GATs were replaced at the same time every 14 (range 10 to 19) days with new ones [14]. As a result, ten collection rounds were executed for each ovitrap, except for ovitraps in Uggiate-Trevano, where the survey started 2 weeks later as for the survey with all GATs.

In addition to the already established *Ae. albopictus* and the indigenous species *Aedes geniculatus*, two other invasive container-breeding mosquito species, i.e. *Aedes japonicus* and *Aedes koreicus*, have started spreading across the study area since 2013 [18]. Although *Ae. japonicus* and *Ae. koreicus* have a lower vectorial competence and therefore lower public health significance compared to *Ae. albopictus* [19], their presence can introduce a confounding factor in the surveillance of *Ae. albopictus*. Indeed, while eggs of *Ae. geniculatus* can be easily distinguished by morphology from the other *Aedes* species, it is not possible to distinguish morphologically the eggs of *Ae. albopictus*, *Ae. japonicus* and *Ae. koreicus* without resorting to special microscopy equipment and expertise [20].

After transportation to the laboratory, the ovitrap wooden slats and GAT adhesive plastic sheets were examined using a stereo microscope (EZ4 D, Leica Microsystems, Germany) for the presence of *Aedes* eggs and adults, respectively. Adult mosquito females in GATs were identified to the species level by morphology and enumerated. To evaluate the presence of *Ae. koreicus* and *Ae. japonicus* in ovitraps, most (87%) of the positive ovitraps were analysed by matrix-assisted laser desorption/ionization time of flight mass spectrometry (MALDI-TOF MS). The rest of the positive ovitraps (13%) could not be analysed because of the low number (1–3) and low quality (dryness or other types of damage) of eggs present on the wooden slat. For this analysis, each wooden slat was divided into ten sectors. For each sector where eggs were present, three to five intact eggs were randomly picked and identified through MALDI-TOF MS with an AXIMA Confidence mass spectrometer (Shimadzu Biotech, Kyoto, Japan) following the method described in Schaffner et al. [20].

### Data analysis

The numbers of *Ae. albopictus* eggs and adult females were recorded in an Excel sheet together with additional information such as the sampling site, date, category of area, etc. (Additional file 2: Table S1, Additional file 3: Table S2 and Additional file 4: Table S3). Statistical analysis was performed through the freely available software R, version 4.0.3 [21]. All analyses are fully reproducible as data, code and package version control tools are available (Additional file 2: Table S1, Additional file 3: Table S2, Additional file 4: Table S3, Additional file 5: Dataset S1, Additional file 6: Dataset S2, Additional file 7: Dataset S3, Additional file 8: Text S1, Additional file 9: Text S2 and Additional file 10: Text S3). All statistical analyses are documented in Additional file 11: Text S4, Additional file 12: Text S5, Additional file 13: Text S6 and Additional file 14: Text S7.

A Spearman’s rank order correlation was used to evaluate the relationship between the number of eggs per ovitrap and the number of *Ae. albopictus* adult females in the GAT deployed in the same sampling site. Both variables were square-root transformed.

Three modelling analyses were performed. The first two analyses aimed at modelling the number of *Ae. albopictus* eggs found in ovitraps and the number of *Ae. albopictus* adult females caught with GATs in 2019, respectively. The third analysis focused on the number of eggs found in ovitraps in 2012, 2013 and 2019. The data for the years 2012 and 2013 come from the study published by Suter et al. [14]. These data are freely available at https://doi.org/10.1371/journal.pntd.0004315.s001. The area monitored in 2012 and 2013 (Additional file 15: Figure S2) was larger compared to the one monitored in 2019 but it included five of the six municipalities surveyed in 2019, namely Balerna, Coldrerio, Mendrisio, Maslianico and Uggiate-Trevano. Additionally, the non-intervention municipality Malnate, surveyed in 2019, was located just outside the southwest boundary of the area studied in 2012 and 2013. Therefore, we believe that the results of both surveys are comparable as well as representative of the situation in the urban communities across the Swiss-Italian border. Note that the study performed by Suter et al. [14] collected data in both sylvatic and urban environments. Since the present study focused on urban environments, for the third analysis we only used the “urban” data from Suter et al. [14] and the data collected in 2019.

The graphical analysis was performed with the ggplot2 package, version 3.3.3 [22]. All three analyses were performed with the glmmTMB function from the glmmTMB package version 1.0.2.1 [23]. Inference was performed with likelihood ratio tests (for *P*-values) and profiling likelihood methods (to estimate confidence intervals) [24]. The level of significance was set at *α* = 0.05. Different distributional families and non-nested models were compared with information criteria [25]. Model assumptions were assessed via usual residuals analyses. Quadratic effects were modelled via orthogonal polynomials.

#### First model

The response variable “number of eggs” (No..eggs.AEDES) was modelled with a generalised mixed-effects model. In particular, to account for the nature of the data, a negative binomial distribution was assumed. This allowed accounting for the fact that we are dealing with count data and that overdispersion is present. Alternative families where compared (Additional file 11: Text S4). The predictor of main interest “AREA” defined whether the trap was to be found in a sampling site under IVM (i.e. in intervention area) or not (i.e. in non-intervention area) and was included as a fixed effect. The other predictors were “Municipality”, “TRAP.ID.fac” (i.e. trap identity), “Day of the year”, “No..Days.ovitrap.in.field” (i.e. number of days that the trap was deployed in the field) and “Altitude” (i.e. altitude of the trap in meters a.s.l.). Municipality and TRAP.ID.fac were taken as random effects. No..Days.ovitrap.in.field was included to account for the “exposure” effect, as not all traps stayed exactly 14 days in the field (range 10 to 19 days). Traps that are left longer in the field are expected to contain more eggs. The seasonal effect of time (i.e. date when ovitrap collected, “Day of the year”) was modelled with a quadratic effect.

The equation used to fit the model is:

$$\begin{gathered} {\text{glmmTMB}}({\text{No}}..{\text{eggs}}.{\text{AEDES }}\sim {\text{ AREA }} + \hfill \\ {\text{poly}}\left( {{\text{Day}}.{\text{ovitrap}}.{\text{collected}},{\text{ degree }} = \, 2} \right) \, + \hfill \\ {\text{scale}}\left( {{\text{ALTITUDE}}} \right) \, + \hfill \\ {\text{No}}..{\text{Days}}.{\text{ovitrap}}.{\text{in}}.{\text{field }} + \hfill \\ \left( {1 \, |{\text{ TRAP}}.{\text{ID}}.{\text{fac}}} \right) \, + \, \left( {1 \, |{\text{ MUNICIPALITY}}} \right), \hfill \\ {\text{family }} = \, {\text{nbinom}}1, \hfill \\ {\text{data }} = {\text{ d}}.{\text{eggs}}.2019) \hfill \\ \end{gathered}$$

#### Second model

The same analysis as in the first model was applied to the response variable “number of *Ae. albopictus* adult females in GAT” (No..Ad..Albo.in.GAT hereafter). As the graphical analysis indicated that there might be an interaction between “Day of the year” and “AREA”, we fitted a model that included this two-fold interaction (Additional file 12: Text S5).

#### Third model

The number of eggs in urban areas in 2012 and 2013 from Suter et al. [14] and the number of eggs collected in 2019 were also modelled together. About half (45%) of the observations in this dataset were zeros. Therefore, the negative binomial used for the two other analyses was extended such that the excess of zeros could be accounted for. To do that, we fitted a hurdle model, where a part of the model focused on the presence-absence part of the data and another part of the model focused on the abundance (Additional file 13: Text S6). The presence-absence part of the model was modelled with binomial family, while the abundance part of the model was modelled with the truncated negative binomial family. The predictors used here were the same as in the previous two analyses, except for the “No..Days.ovitrap.in.field”, not present in Suter et al. [14] data and therefore not included. In addition, the variable “Year” (2012, 2013 and 2019) was added to the model. This model is also a generalised mixed-effects model.

## Results

In each of the six municipalities studied, six sampling sites were selected and one ovitrap and one GAT were deployed in each sampling site. The wooden slats of ovitraps and adhesive plastic sheets of GATs were replaced with new ones every 2 weeks (range 10–19 days). In five municipalities (i.e. Balerna, Coldrerio, Malnate, Maslianico and Mendrisio), ovitraps were deployed from mid-end May and inspected for ten consecutive rounds, with a total of 300 wooden slats collected (5 municipalities × 6 sampling sites × 1 ovitrap × 10 collection rounds). In Uggiate-Trevano, the deployment of ovitraps started 2 weeks later and inspection was carried out for nine rounds, with 54 wooden slats collected in total (1 municipality × 6 sampling sites × 1 ovitrap × 9 collection rounds). Deployment of GATs also started 2 weeks after the first deployment of ovitraps, in all six municipalities, and traps were inspected for nine rounds, with a total of 324 adhesive sheets collected (6 municipalities × 6 sampling sites × 1 GAT × 9 collection rounds).

Of the 354 and 324 times ovitraps and GATs were inspected, traps were found altered (e.g. turned over or with missing parts) 27 and 23 times, respectively (Table 1, Additional file 2: Table S1 and Additional file 3: Table S2). From the 327 intact wooden slats and 301 intact adhesive sheets, 263 (80.4%) and 256 (85.1%) were positive for *Aedes* spp. eggs and adult females, respectively. MALDI-TOF MS was used to evaluate the presence of different exotic *Aedes* species in 229 of the 263 positive wooden slats. For the majority (200, i.e. 87%) of the slats analysed, MALDI-TOF MS detected eggs of *Ae. albopictus* only. In the remaining 29 slats, the species *Ae. japonicus* and *Ae. koreicus* were also detected in June and July in all the municipalities surveyed. Of the 256 positive adhesive sheets, 249 (97%) captured only adults of *Ae. albopictus*. *Aedes japonicus* was found on five adhesive sheets in Balerna, Coldrerio and Mendrisio (five adults in total) in June and July. *Aedes koreicus* was captured on two adhesive sheets in Malnate and Uggiate-Trevano (six adults in total) in June and July. These results suggest that the large majority of the eggs found in the ovitraps were laid by *Ae. albopictus*. Therefore, the eggs in the ovitraps were all counted as *Ae. albopictus* eggs in the subsequent analyses. Moreover, a significant positive correlation (*rs*_(281)_ = 0.381, *P* < 0.0001) was observed between the number of eggs per ovitrap and the number of *Ae. albopictus* adult females in the GAT deployed 20–100 m from the corresponding ovitrap.

**Table 1:** Summary statistics of *Ae. albopictus* egg (ovitrap wooden slats) and adult female (GAT adhesive plastic sheets) counts in the six municipalities examined

Municipality (type of area)	Trap type (tot. deployed)	Altered	Positive (%)	*Ae. albopictus* egg/adult count per trap			
				Minimum	Median	Mean	Maximum
Balerna	Ovitrap (60)	11	38 (63.3)	0	25.0	56.8	407
(Intervention)	GAT (54)	0	42 (77.8)	0	4.0	4.1	13
Coldrerio	Ovitrap (60)	4	40 (66.7)	0	18.5	80.1	513
(Intervention)	GAT (54)	2	43 (79.6)	0	4.0	6.3	22
Mendrisio	Ovitrap (60)	0	35 (58.3)	0	13.5	59.3	401
(Intervention)	GAT (54)	2	37 (68.5)	0	2.0	3.2	16
Malnate	Ovitrap (60)	1	53 (88.3)	0	144.0	261.2	2,117
(Non-intervention)	GAT (54)	3	48 (88.9)	0	10.0	14.2	83
Maslianico	Ovitrap (60)	5	51 (85.0)	0	160.0	218.6	1,073
(Non-intervention)	GAT (54)	10	40 (74.1)	0	6.0	7.7	26
Uggiate-Trevano	Ovitrap (54)	6	46 (85.2)	0	184.5	223.8	864
(Non-intervention)	GAT (54)	6	46 (85.2)	0	13.0	18.0	64

In 2019, egg counts per ovitrap per inspection rounds of about 14 days ranged from 0 to 513 in the municipalities that were part of the intervention area (i.e. Balerna, Coldrerio and Mendrisio) and from 0 to 2117 in the municipalities not following a defined management plan (i.e. Malnate, Maslianico and Uggiate-Trevano) (Table 1). Mean *Ae. albopictus* egg counts were consistently higher in the non-intervention municipalities (Table 1).

**Fig. 2:**
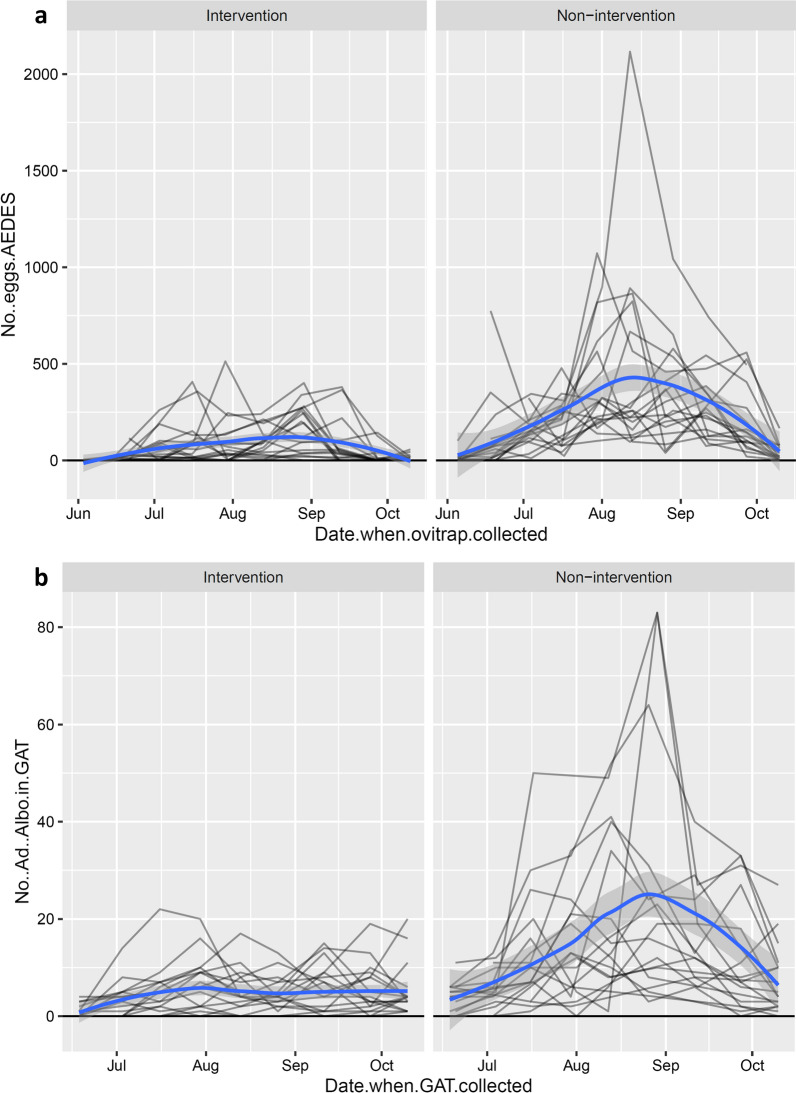
Numbers of *Ae. albopictus* eggs per ovitrap (**a**) and adult females per GAT (**b**) over time in intervention and non-intervention municipalities. Each line on the graphs represents a site. Note that these graphs represent the raw data. Smoothers (blue lines with grey confidence bands) were added to highlight seasonal trends

**Fig. 3:**
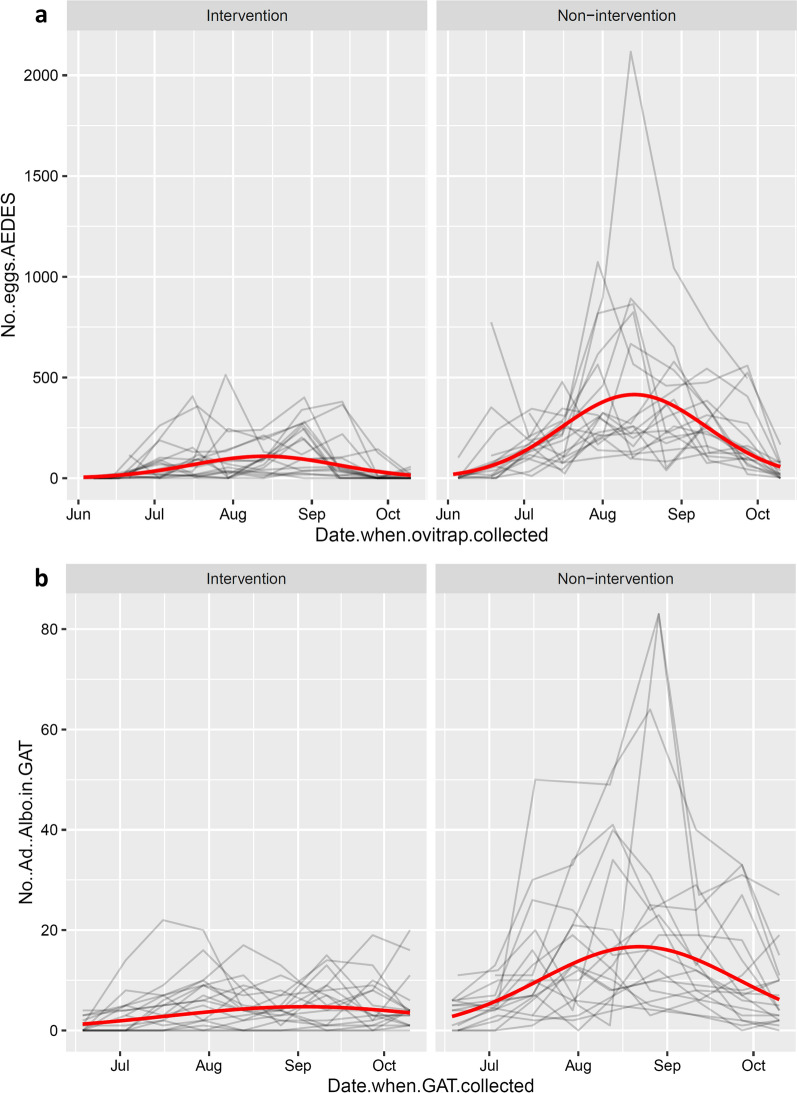
Observed data (grey lines) and model fit (red lines) for numbers of *Ae. albopictus* eggs per ovitrap (**a**) and adults per GAT (**b**) in intervention and non-intervention municipalities. Each grey line represents a site (i.e. the actual observations used to fit the model). The fit is visualized in the original scale (i.e. without any transformation). Note that the fitted values shown here are not adjusted to correct for bias due to Jensen’s inequality [26]. This applies to all graphs produced with fitted or predicted values here

The first eggs in the season were found already in the first period of the survey in late May to early June (Fig. 2a). In the non-intervention municipalities, there was a steady increase in the number of eggs with a peak in August, followed by a decrease in September and October, indicating the end of the reproductive season. In the intervention municipalities, the increase in the number of eggs was much more contained compared to the non-intervention municipalities, without an evident peak (Fig. 2a). The model fitted well (Additional file 11: Text S4) and the fitted values agreed with the raw data (Figs. 2a, 3a). The effect of AREA (i.e. intervention vs. non-intervention) was clearly present and biologically relevant (*P* < 0.0001). In non-intervention sites there were about four times (3.8) more eggs than in intervention sites (95% confidence interval, CI: 2.7–5.4). The estimated variability of the random effects indicated that there was very little variation among municipalities. Note, however, that the variance component “municipality” is estimated based on six municipalities only. This number does fulfil the minimal number of levels required to obtain a sensible estimated of a variance component, however, does not allow to estimate it with great precision [24]. As the main predictor of interest varies among municipalities, we have further inspected this limitation of the model. In particular, we also fitted a generalised mixed-effects model where municipality was taken as a fixed effect. The municipality estimates were then used to perform a post hoc test to compare the two AREA levels (i.e. “intervention” against “non-intervention”). The results of this alternative approach are fully in agreement with the model where municipality is taken as a random effect. In both models, AREA plays a very relevant role. Indeed, the estimated AREA effect was almost identical in these two models and also the inference procedure leads to equivalent results. Note that for the sake of brevity and to avoid redundancy, the results of this alternative but agreeing analysis are not shown in the appendices. There seemed to be some variation among ovitraps. To quantify these differences among traps we looked at the two most extreme estimated conditional modes: the “worst” ovitrap had − 40% eggs with respect to an “average” ovitrap; the “best” ovitrap had + 140% eggs with respect to an “average” ovitrap. Altitude did not seem to play a relevant role (*P* = 0.607). The number of days of trap deployment in the field had a significant effect (the model predicted about 10% more eggs for each additional day the ovitrap was deployed in the field; *P* = 0.003).

The number of *Ae. albopictus* adult females per GAT in 2019 ranged from 0 to 22 in the municipalities in intervention area and from 0 to 83 in the municipalities in non-intervention area. As for the egg counts, mean *Ae. albopictus* adult female counts were consistently higher in the non-intervention municipalities than in the intervention ones (Table 1). The same seasonal trend as for eggs, with a much more contained increase and no evident peak in numbers in the intervention municipalities, compared to the non-intervention ones, could be observed for adult females (Figs. 2b, 3b).

**Fig. 4:**
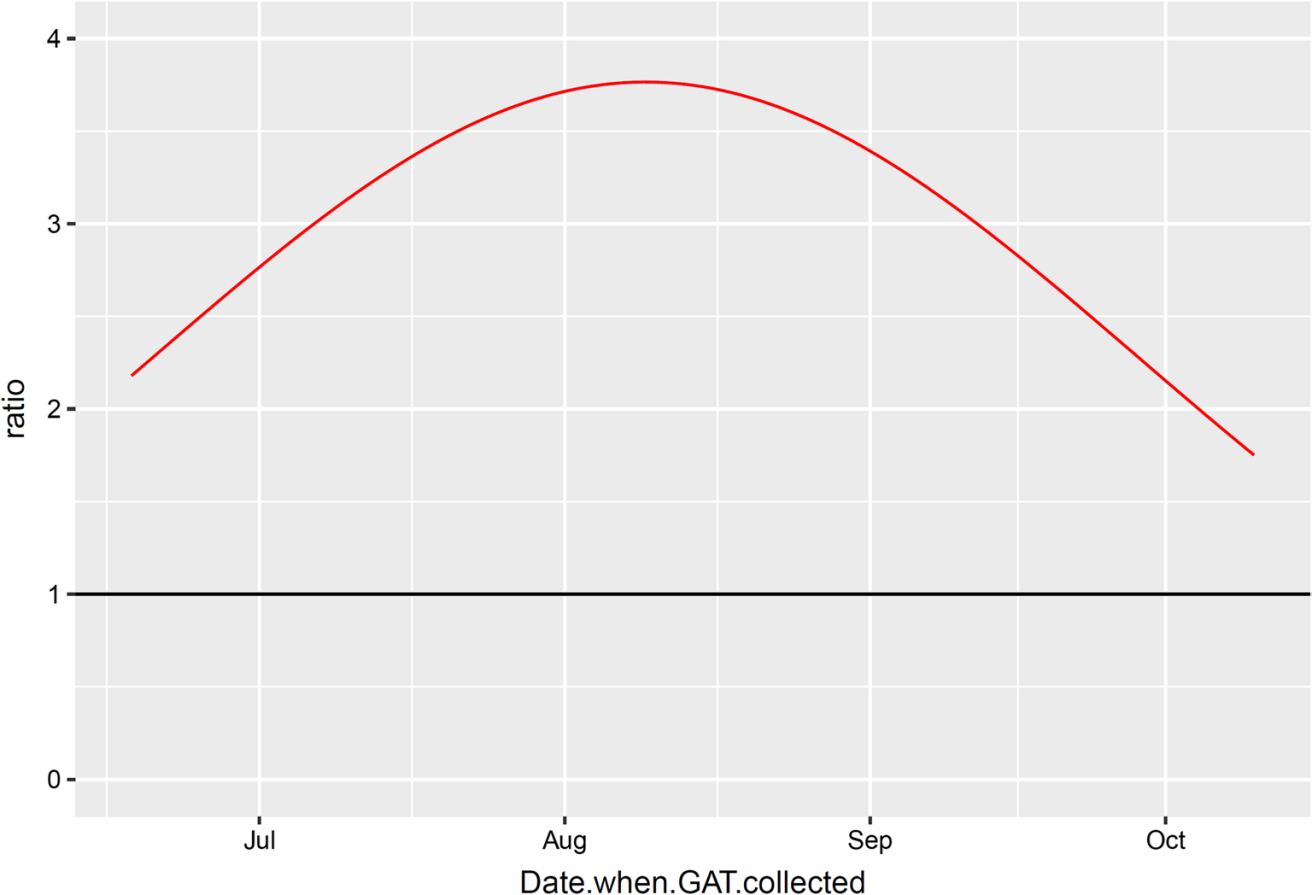
Evolution of the non-intervention/intervention ratio over time for the number of *Ae. albopictus* adult females per GAT. The non-linear effect of time is approximated with a quadratic function

The model fitted well (Additional file 12: Text S5) and the fitted values agreed with the raw data (Figs. 2b, 3b). The numbers of *Ae. albopictus* adult females per GAT were significantly higher in the non-intervention sites than in the intervention ones (*P* < 0.0001; see Additional file 12: Text S5). However, differently from the numbers of eggs, the ratio between non-intervention and intervention was not fixed over time (Fig. 4). The ratio varied between about two at the beginning and end of the season and increased to almost four in early/mid-August. Compared to the numbers of eggs, there was more variation among municipalities belonging to the same area group. Neither the altitude nor the number of days of trap deployment in the field seemed to play a relevant role (*P* = 0.095 and *P* = 0.566, respectively). There was non-negligible variability among traps (“worst” GAT: − 80% adults with respect to an “average” GAT; “best” trap: + 190% adults with respect to an “average” GAT).

**Fig. 5:**
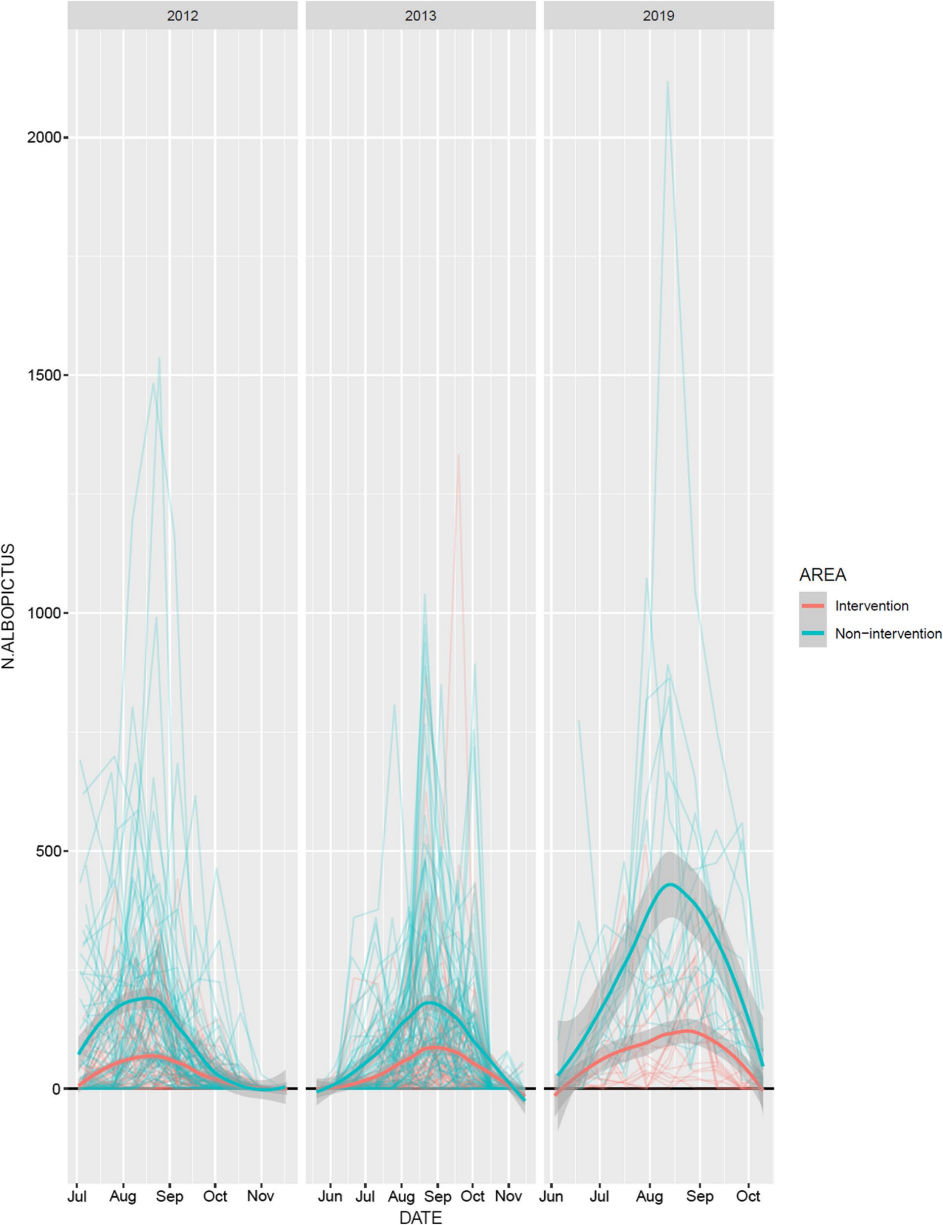
Numbers of eggs per ovitrap over time in intervention (red) and non-intervention (green) municipalities, for the years 2012, 2013 [14] and 2019. Each red and green fine line represents a trap. Smoothers (lines with grey confidence bands) were added to highlight seasonal trends

The difference between the intervention and non-intervention areas was quite evident also when we compared the numbers of eggs per ovitrap among 2012, 2013 [14] and 2019 (Fig. 5). Furthermore, this difference seems to have massively increased after 2013. The model included the three-fold interaction among time of the year (day), year and AREA. In other words, the data strongly supported the hypothesis that the seasonal bow-shaped pattern differed among years and among AREA levels.

**Fig. 6:**
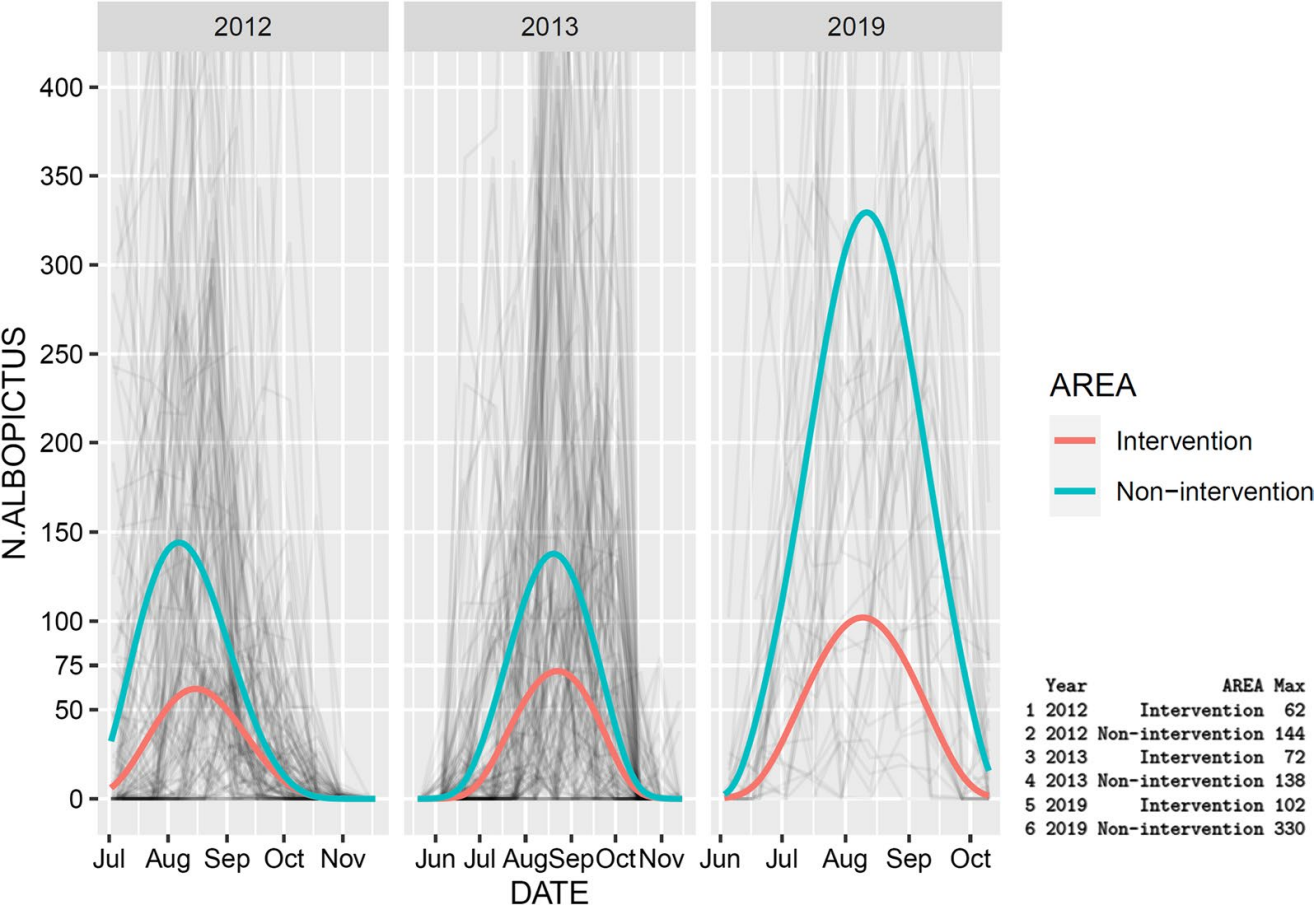
Observed data (grey lines) and model fits (green and red lines) for numbers of eggs per ovitrap in intervention and non-intervention municipalities in the years 2012, 2013 [14] and 2019. Each grey line represents an ovitrap (i.e. the actual observations used to fit the model). The fit is visualized in the original scale, without any transformation. Graphs zoomed to the area between 0 and 400 counts. The x-range is adapted to each panel. The maximal fitted values in each year-area combination are shown on the bottom right

The model fit also showed that more eggs were consistently found in the non-intervention than intervention sites and that the non-intervention/intervention ratio appears to have dramatically increased from 2013 to 2019 (Fig. 6). The non-intervention/intervention ratio was not fixed but varied over the time in 2012 and 2013, as well as across years. As this model is composed of two parts, it is not possible to show a simple graph of the evolution of the ratio over time and across years (Additional file 13: Text S6). However, the graph with the fitted values (Fig. 6) is very useful to understand what is happening over time. Indeed, the peak in the non-intervention sites went from fewer than 150 eggs in 2012 and 2013 to about 330 in 2019 (Fig. 6). On the other hand, the increase for intervention sites was much more moderate (i.e. from about 65 to about 100). The actual observations (i.e. the raw data) went up to more than 2000 in 2019, which compressed the graph. Therefore, the graphs in Fig. 6 are zoomed to the area of interest (i.e. between ) and 400 counts) to better compare curves.

In this model, altitude seemed to play a role (*P* = 0.051), with an expected negative effect on the numbers of eggs. There was some relevant variability among traps and among municipalities (Additional file 13: Text S6). The presence/absence process seemed to be affected more by municipality than by trap (Additional file 13: Text S6). The abundance, contrarily, seemed to be affected by trap more than by municipality (Additional file 13: Text S6). Note that, although the increase of the ratio over year seems to be very strong, additional years are required to draw robust conclusions about this trend. A future repetition of this study is indeed planned with a larger number of locations on both sides of the border.

## Discussion

The 2019 survey highlighted a conspicuous difference between intervention and non-intervention municipalities in the seasonal distribution of numbers of both eggs and adult females of *Ae. albopictus*. In the non-intervention municipalities, there was a steady increase in the number of eggs and adults starting in June with a peak in August, followed by a decrease in September and/or October, indicating the end of the mosquito reproductive season. In the intervention municipalities, the increase in the number of eggs and adults was much more contained, without an evident peak. This very clear pattern strongly supports the efficacy of the IVM programme implemented in Ticino, helping in keeping the numbers of *Ae. albopictus* very stable during the reproductive season.

In 2019, the number of *Ae. albopictus* eggs in the urban environment was 3.8 times higher in non-intervention sites than in intervention sites. Compared to the situation in 2012 and 2013, with a proportion of 2.26 [14], the divergence between non-intervention and intervention areas seems to have dramatically increased (note, however, that these two ratios are not formally compared). In the Italian communities, where no IVM programme was implemented, we noticed a striking increase in the number of eggs per ovitrap, with an average of about 50 eggs in 2012 and 2013 to more than 200 eggs in 2019. The fitted model showed a peak in eggs per ovitrap from less than 150 in 2012 and 2013 to about 330 in 2019. In comparison, in the Swiss sites following an IVM programme, the increase was much more moderate, with an average of about 20 eggs per ovitrap in 2012 and 2013, to between 57 and 80 eggs in 2019, while the fitted model showed a moderate increase in the peak of eggs from about 65 in 2012 and 2013 to about 100 eggs per ovitrap in 2019. These observations strongly suggest that the IVM programme implemented in Ticino helped keep the numbers of *Ae. albopictus* almost stable over the years in the urban environment. Contrarily, the absence of an IVM programme in the Italian communities across the border seems to lead to a deterioration of the situation, with an increase in numbers of *Ae. albopictus*. Further evaluations of the control system in the coming years will allow confirming whether this is a consolidated trend.

In 2013, Suter et al. [14] observed that few ovitraps (30%) in Ticino were positive for *Ae. albopictus* earlier in the warm season, in early/mid-June, while in Italy many traps (62%) were already positive during the same period. A possible explanation was the positive impact of control treatments at the end of the 2012 season and before the start of the 2013 season in lowering mosquito reproduction in Ticino. Another suggested possibility was that mosquito populations in Ticino, rather than being stable overwintering populations, were annually re-introduced from Italy, so that their numbers managed to pick up only later in the season. In early/mid-June 2019, in contrast, half of the ovitraps (50%) in Ticino were already positive for *Ae. albopictus*, and most ovitraps (80%) were positive in the non-intervention areas. It seems, therefore, that in 2019 the numbers of tiger mosquitoes in Ticino picked up faster compared to 2013. This could be due to the presence of more stable overwintering populations in 2019 compared to those of 2013. However, at the beginning of the 2019 survey, between end of May and beginning of June, very few ovitraps (7%) were positive in Ticino, while half of the traps (58%) were positive in the non-intervention municipalities. A likely explanation is that the larvicide treatments in Ticino at the end of the 2018 reproduction season reduced the number of mosquitoes laying diapausing eggs. This, in addition to the impact of control treatments at the start of the 2019 season, slowed down the annual reconstitution of mosquito populations in the intervention areas contrasting to the non-intervention areas.

A concern in the 2012 and 2013 survey was the use of egg counts from ovitraps to estimate and compare *Ae. albopictus* densities. Ovitrap data are considered appropriate to assess presence/absence of *Ae. albopictus* in a given site but not for adult population estimation because the relationship between the two parameters might be affected by several factors. For example, a single female mosquito might lay eggs at multiple breeding sites or the ovitraps may compete with nearby sites [13]. In 2019, gravid *Aedes* traps (GATs) were deployed in parallel to the ovitraps to compare the two surveillance methods. Both methods showed variability among traps in the same municipality and within traps themselves, with a higher variability among GATs. A possible explanation could be variability in the presence of breeding sites other than traps during the study period, with an effect more accentuated on the GATs, since individuals are captured with this method. Nevertheless, a significant positive correlation was found between eggs in ovitraps and number of *Ae. albopictus* adult females as shown by other studies [9, 27, 28]. The numbers of *Ae. albopictus* adult females per GAT followed the same trend as the numbers of eggs per ovitrap, being significantly higher in the non-intervention sites than in the intervention ones, with a ratio of non-intervention/intervention areas varying between two and four over the season. Therefore, we concluded that both egg and adult data are useful to determine the efficacy of intervention methods employed, or lack thereof.

It could be problematic to estimate the numbers of *Ae. albopictus* by using ovitraps because of the increasing presence of other invasive *Aedes* species, such as *Ae. japonicus* and *Ae. koreicus*, whose eggs cannot be clearly morphologically discerned from the eggs of *Ae. albopictus* and, therefore, could consequently introduce a bias in the evaluation of egg counts. However, the identification of randomly picked eggs through MALDI-TOF MS, combined with the morphological species determination of adults, confirmed that most mosquitoes found at the sampling sites were tiger mosquitoes. Thereby, the influence of other *Aedes* species on the results was negligible. Regardless, the containment measures adopted for *Ae. albopictus* also apply to other container-breeding *Aedes* species. Therefore, by lowering the density of the other depositing mosquitoes we were not introducing a bias in the data.

In terms of public health risk, a main concern related to the presence and abundance of the tiger mosquito is its role as a vector of arboviruses. Outbreaks of chikungunya and dengue viruses have already occurred in Italy and France [8]. In Switzerland, autochthonous cases of chikungunya and dengue viruses have not been reported so far and we are not aware of autochthonous cases in the Italian communities included in the present study. However, the number of imported cases in Switzerland, including Ticino, increases regularly (https://www.bag.admin.ch) as it does in the neighbouring Italian regions (https://www.epicentro.iss.it/arbovirosi/bollettini) and in other European countries. In 2008, after the 2007 chikungunya epidemic in the Emilia-Romagna region of northern Italy, Carrieri and collaborators [29] calculated the epidemic risk threshold in terms of numbers of eggs per ovitrap above which an arbovirus epidemic may initiate, in presence of imported human cases. A threshold of 250–450 and 451–750 eggs per ovitrap in 14 days was calculated for an epidemic of E1-A226V mutated and non-mutated form of the chikungunya virus, respectively [30]. In Ticino intervention areas, the mean number of eggs per ovitrap in 14 days in 2019 was between 57 and 80, while in non-intervention areas across the Italian border it was about 200. Maximum number of eggs per ovitrap in Ticino was between 400 and 500, while in non-intervention areas across the Italian border it was about 1000. Although the geographical characteristics of the Emilia-Romagna region are different from the area monitored in the present work, we perceive that the risk of an arbovirus epidemic is much more probable in the non-intervention areas. Moreover, even with the lower number of *Ae. albopictus* in Ticino, a study carried out in 2018 in six municipalities of the canton estimated that the risk of outbreak in the case of the introduction of chikungunya, dengue or Zika viruses was present in all the municipalities investigated [31]. Consequently, a strategy for preventing and managing potential arbovirus outbreaks, as well as the surveillance and control activities of *Ae. albopictus* according to the situation and level of epidemic risks, has been recently elaborated for Ticino [32].

The scope of this work was to evaluate the effectiveness of integrated control practices in the field, where not all variables can be controlled. As an observational study, we must be aware that differences between intervention sites and non-intervention sites could be explained by other yet unknown factors differing between Switzerland and Italy. This is particularly important given the fact that 36 sites inspected were grouped in six municipalities. Ideally, larger sample sizes will be required to strengthen the evidence in favour of IVM programmes, which requires considerable sampling and logistic efforts. Note that following an experimental approach, namely setting up untreated control sites in Ticino, where all municipalities follow an IVM programme, would be ethically unfeasible. The non-intervention control sites selected in Italy were very similar and geographically close to the intervention sites in Ticino.

Our results are in accordance with previous studies on integrated control strategies (e.g. [33–35]). These results strongly support the hypothesis that IVM plays an essential role in reducing the nuisance for the human population. In addition, from a public health point of view, it might limit both the risk of autochthonous transmission and the size of potential epidemics, as shown by Guzzetta et al. [36]. According to Baldacchino et al. [9], the most effective integrated control includes door-to-door education. The door-to-door education and treatment actions were included in the Ticino IVM between 2008 and 2010 [5]. During this period, breeding sites in private domains were removed directly by GLZ, Civil Protection Units or municipality workers after agreement with the residents. With the gradual spread of the mosquito to larger areas, this fine-scale approach became less and less sustainable. Therefore, the part of the IVM regarding private domains currently focuses on a less fine but constant approach over the years with extensive public information campaigns carried out every year, including for example information events and door-to-door delivery of education material [5]. In addition, the municipalities can issue a specific ordinance not permitting neglected breeding sites for the tiger mosquito on the municipality territory. Consequently, the GLZ and the municipality workers are allowed to conduct inspections to verify the presence of breeding sites in private domains and report violations of the ordinance.

Reintroductions of mosquitoes in Ticino from across the border are probably occurring every year. From our data, it is not possible to tell the effect of these reintroductions on the quantities of *Ae. albopictus* in Ticino. However, the results indicate that with the implementation of an IVM programme, it is possible to contain the numbers of *Ae. albopictus* at a manageable level, irrespective of possible constant reintroduction of individuals from outside the intervention areas. Although it would certainly be desirable to undertake concerted actions across the Swiss-Italian Insubria region, with the development and implementation of a transnational action plan for the surveillance and control of *Ae. albopictus*, it is possible to achieve containment of the vector also without cross-border concerted measures.

## Conclusions

In 2019, *Ae. albopictus* egg numbers in urban environment were about four times higher in non-intervention sites, on the Italian side of the Swiss-Italian border than in intervention sites in Ticino. The numbers of *Ae. albopictus* adult females followed the same trend. In addition, the comparison with the previous survey carried out in 2012 and 2013 indicates that this proportion seems to have almost doubled in the past 6 years. We acknowledge that other unknown factors might explain the difference in mosquito densities, and further studies are required to collect additional evidence (this is especially true for the increasing trend in ratios observed here). Nevertheless, the results here support the effectiveness of the IVM programme implemented in Ticino. Thus, the integration of control measures targeting the aquatic phase of the mosquito (i.e. removal of breeding sites and treatment of permanent ones with larvicides) and different public education strategies seem to help in keeping the numbers of *Ae. albopictus* almost stable during the reproductive season of the mosquito. In addition, the perpetuation of these measures seems to help keeping the numbers of *Ae. albopictus* almost stable even over the years in the urban environment. These are relatively simple measures that, if constantly maintained over the years, show their effectiveness in keeping the mosquito population in check.

## Supplementary Information

**Additional file 1**: **Figure S1**. Comparison of monthly average (a), minimum (b) maximum (c) temperatures and precipitations (d) between intervention and non-intervention areas.

**Additional file 2**: **Table S1**. Data from ovitraps with *Aedes albopictus* egg counts, in csv format. Variables included are WGS84.LAT (latitude of trap); WGS84.LNG (longitude of trap); ALTITUDE (altitude of trap); AREA (type of area, i.e. intervention or non-intervention); MUNICIPALITY; Date.when.ovitrap.installed; Date.when.ovitrap.collected; No..Days.ovitrap.in.field; Week.when.ovitrap.collected; No..eggs.AEDES; No..Eggs.AEDES.in.14.days; TRAP.ID.fac (trap identity); Day.ovitrap.collected and no.eggs.normalised.14.days.

**Additional file 3**: **Table S2**. Data from Gravid *Aedes* Traps (GATs) with *Aedes albopictus* female counts, in csv format. Variables included are WGS84.LAT (latitude of trap); WGS84.LNG (longitude of trap); ALTITUDE (altitude of trap); AREA (type of area, i.e. intervention or non-intervention); MUNICIPALITY; Date.when.GAT.installed; Date.when.GAT.collected; No..Days.GAT.in.field; Week.when.GAT.collected; No..Ad..Albo.in.GAT; No..Ad..Albo.in.14.days; TRAP.ID.fac (trap identity) and Day.GAT.collected.

**Additional file 4**:** Table S3**. *Aedes albopictus* egg counts from 2012, 2013 [14] and 2019 in csv format. Variables included are AREA (type of area, i.e. intervention or non-intervention); MUNICIPALITY; TRAP.ID.fac (trap identity); DATE; N.ALBOPICTUS (number of Ae. albopictus eggs); ALTITUDE (altitude of trap); Year; Day (day of the year) and study (Flacio = present study; Suter = Suter et al. [14]).

**Additional file 5**:** Dataset S1**. Data from ovitraps with *Aedes albopictus* egg counts in 2019, in RDS format (https://www.r-project.org/https://www.r-project.org/).

**Additional file 6**:** Dataset S2**. Data from Gravid *Aedes* Traps (GATs) with *Aedes albopictus* female counts in 2019, in RDS format (https://www.r-project.org/).

**Additional file 7**:** Dataset S3**. *Aedes albopictus* egg counts from 2012, 2013 [14] and 2019 in RDS format (https://www.r-project.org/).

**Additional file 8**:** Text S1**. R-script for the analysis of the number of *Aedes albopictus* eggs collected in 2019. This R-script has been extracted from the corresponding Rmarkdown file (https://rmarkdown.rstudio.com/). It allows for the full reproducibility of the analysis.

**Additional file 9**:** Text S2**. R-script for the analysis of the number of *Aedes albopictus* adult females collected in 2019. This R-script has been extracted from the corresponding Rmarkdown file (https://rmarkdown.rstudio.com/). It allows for the full reproducibility of the analysis.

**Additional file 10**:** Text S3**. R-script for the analysis of the number of *Aedes albopictus* eggs collected in 2012, 2013 [14] and 2019. This R-script has been extracted from the corresponding Rmarkdown file (https://rmarkdown.rstudio.com/). It allows for the full reproducibility of the analysis.

**Additional file 11**: **Text S4**. This pdf file documents the full analysis of the number of *Aedes albopictus* eggs collected in 2019. This pdf was created with Rmarkdown and allows for the full reproducibility of the analysis.

**Additional file 12**: **Text S5**. This pdf file documents the full analysis of the number of *Aedes albopictus* adult females collected in 2019. This pdf was created with Rmarkdown and allows for the full reproducibility of the analysis.

**Additional file 13**: **Text S6**. This pdf file documents the full analysis of the number of *Aedes albopictus* eggs collected in 2012, 2013 [14] and 2019. This pdf was created with Rmarkdown and allows for the full reproducibility of the analysis.

**Additional file 14**: **Text S7**. This pdf file documents additional data analyses of the number of *Aedes albopictus* eggs collected in 2019 performed at a reviewer’s request. This pdf was created with Rmarkdown and allows for the full reproducibility of the analysis.

**Additional file 15**: **Figure S2**. Study area in the 2012 and 2013 evaluation of the Ticino intervention programme [14]. The map was prepared using the geographic information system (GIS) software ArcGIS version 10.0 (ESRI Inc., USA).

## Data Availability

All data generated or analysed during this study are included in this published article and its supplementary information files.

## Abbreviations

GAT: Gravid *Aedes* trap
GLZ: Gruppo Lavoro Zanzare
IVM: Integrated vector management
MALDI-TOF MS: Matrix-assisted laser desorption/ionization time of flight mass spectrometry
