# Supplementary material for: Effectiveness of integrated Aedes albopictus management in southern Switzerland
Source: Parasit Vectors. 2021 Aug 16;14:405. doi: 10.1186/s13071-021-04903-2 (PMC8365973; doi:10.1186/s13071-021-04903-2)
Supplement: Supplementary file 1 — Additional file 1: Figure S1. Comparison of monthly average (a), minimum (b) maximum (c) temperatures and precipitations (d) between intervention and non-intervention areas. [file 13071_2021_4903_MOESM1_ESM.pdf]

Additional file 1 Fig. S1. Comparison of monthly average (a), minimum (b), maximum (c) temperatures and precipitations (d) between intervention and non-intervention areas.

We analysed the temperatures (a-c) and precipitations (d) of the meteorological stations of Mezzana, located in the intervention area between Balerna and Coldrerio (45.8528024, 8.9973972, altitude 340 m, data from https://www.agrometeo.ch/) and Olgiate Comasco, located in the non-intervention area, about 5 km south from Uggiate Trevano (45.77306208, 8.972629614, altitude 400 m, data https://www.arpalombardia.it). Monthly temperatures are very similar between intervention and non-intervention areas, both in 2012 and 2019. Monthly precipitations in 2019 are also very similar between the two areas.

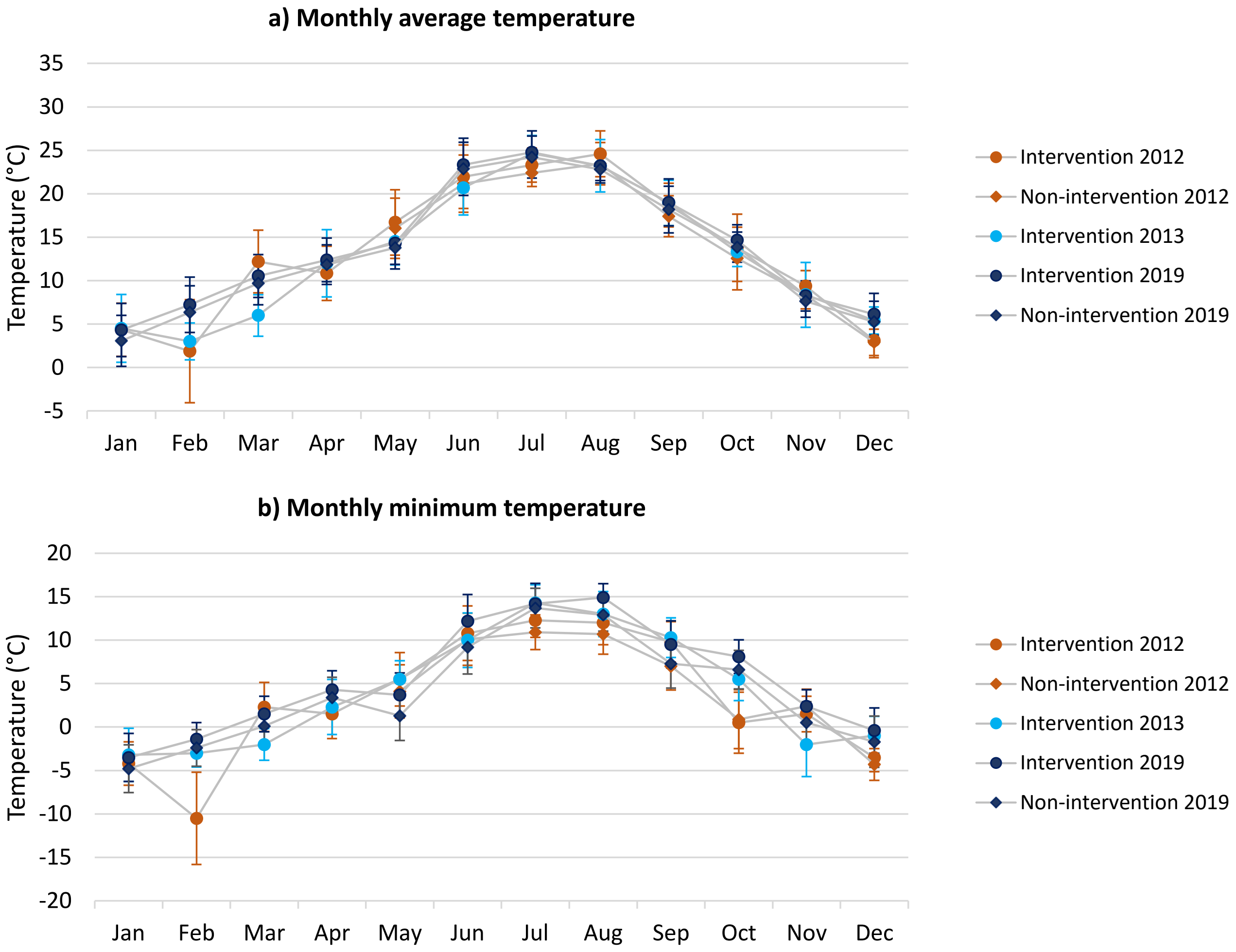

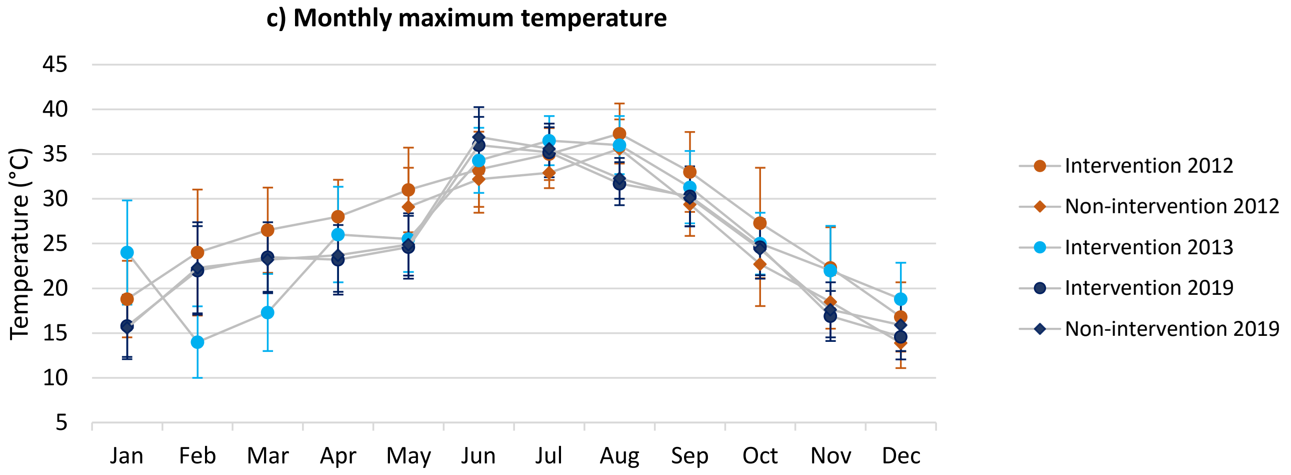
c) Monthly maximum temperature
Temperature (°C)
45
40
35
30
25
20
15
10
5
Jan
Feb
Mar
Apr
May
Jun
Jul
Aug
Sep
Oct
Nov
Dec
Intervention 2012
Non-intervention 2012
Intervention 2013
Intervention 2019
Non-intervention 2019

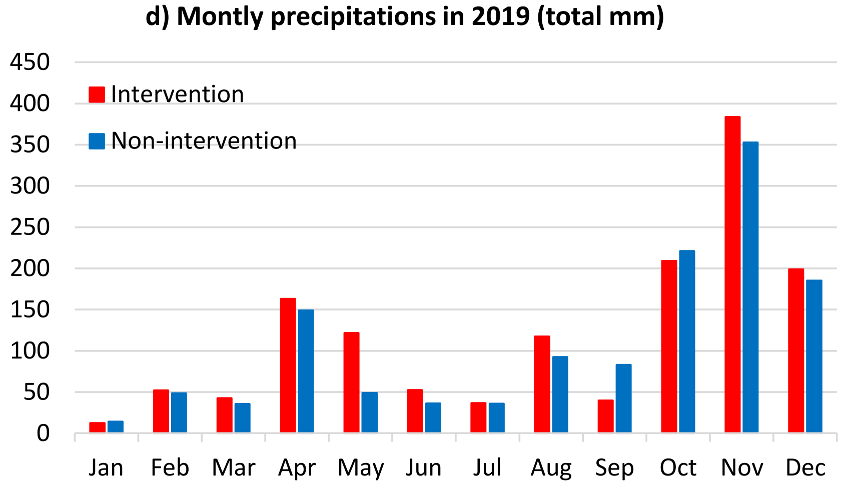
d) Montly precipitations in 2019 (total mm)
450
400
350
300
250
200
150
100
50
0
Intervention
Non-intervention
Jan
Feb
Mar
Apr
May
Jun
Jul
Aug
Sep
Oct
Nov
Dec
