# Supplementary material for: Effectiveness of integrated Aedes albopictus management in southern Switzerland
Source: Parasit Vectors. 2021 Aug 16;14:405. doi: 10.1186/s13071-021-04903-2 (PMC8365973; doi:10.1186/s13071-021-04903-2)
Supplement: Supplementary file 11 — Additional file 11: Text S4. This pdf file documents the full analysis of the number of Aedes albopictus eggs collected in 2019. This pdf was created with Rmarkdown and allows for the full reproducibility of the analysis. [file 13071_2021_4903_MOESM11_ESM.pdf]

Additional file 11: Data Analysis Nr. eggs 2019

Dr. Matteo Tanadini | Zurich Data Scientists (ZDS)

March 24, 2021

Contents

1 Reproducibility **2**

2 Loading packages **2**

3 Getting data **2**

4 Graphical Analysis **4**

4.1 AREA . . . 4
4.2 MUNICIPALITY . . . 5
4.3 TRAP.ID.fac . . . 6
4.4 Date.when.ovitrap.collected . . . 7
4.5 No..Days.ovitrap.in.field . . . 9
4.6 ALTITUDE . . . 10
4.7 Spatial structure . . . 11

5 Modelling **13**

5.1 Generalised Mixed-Effects Models . . . 13
5.2 Visualising the model fit . . . 15
5.3 Quantifying effects . . . 19
5.4 Model checking . . . 20
5.4.1 Checking the model equation . . . 20
5.4.2 Checking the structure of the random effects . . . 22
5.4.3 Checking normality of the random effects . . . 23
5.4.4 Checking the mean-variance relationship assumption . . . 23
5.4.5 Checking whether zero-inflation is needed . . . 24
5.4.6 Graphically evaluating the goodness-of-fit . . . 26

6 Conclusions **27**

7 Session Information **27**

1 Reproducibility

In order to make the analysis fully reproducible, we "freeze" package versions using the *{checkpoint}* package. In particular, we use all packages versions available on CRAN on first of March 2021.

```
## (in this chunk messages are omitted)
##
library(checkpoint)
checkpoint("2021-03-01",
           checkpointLocation = "../")
```

2 Loading packages

We load all add-on packages used in this analysis.

```
## (messages are omitted from this chunk)
##
library(dplyr)
library(lattice)
library(ggplot2)
library(glmmTMB)
library(lubridate)
library(gridExtra)
```

3 Getting data

Note that to ensure reproducibility, the platform-agnostic file type "RDS" is used. Nevertheless, the corresponding "csv" file is also provided.

```
d.eggs.2019 <- readRDS("../0_Data_Preparation_For_CH_vs_I/Created_Datasets/InterventionPaper_eggs.RDS")
##
str(d.eggs.2019)
```

```
tibble [327 x 14] (S3: tbl_df/tbl/data.frame)
 $ WGS84.LAT                  : num [1:327] 45.8 45.8 45.8 45.8 45.8 ...
 $ WGS84.LNG                  : num [1:327] 9 9 9 9 9 ...
 $ ALTITUDE                   : num [1:327] 263 263 263 263 263 263 263 263 316 316 ...
 $ AREA                       : Factor w/ 2 levels "Intervention",..: 1 1 1 1 1 1 1 1 1 1 ...
 $ MUNICIPALITY               : Factor w/ 6 levels "Balerna","Coldrerio",..: 1 1 1 1 1 1 1 1 1 1 ...
 $ Date.when.ovitrap.installed: POSIXct[1:327], format: "2019-05-23" "2019-06-09" ...
 $ Date.when.ovitrap.collected: POSIXct[1:327], format: "2019-06-09" "2019-06-21" ...
 $ No..Days.ovitrap.in.field  : num [1:327] 17 12 14 14 15 14 16 13 12 13 ...
 $ Week.when.ovitrap.collected: num [1:327] 23 25 31 33 35 37 39 41 25 29 ...
 $ No..eggs.AEDES             : num [1:327] 0 29 0 52 202 34 0 57 0 407 ...
 $ No..Eggs.AEDES.in.14.days  : num [1:327] 0 33.8 0 52 188.5 ...
 $ TRAP.ID.fac                : Factor w/ 36 levels "BAL-11a","BAL-2b",..: 1 1 1 1 1 1 1 1 2 2 ...
 $ Day.ovitrap.collected      : num [1:327] 160 172 211 225 240 254 270 283 172 197 ...
 $ no.eggs.normalised.14.days : num [1:327] 0 33.8 0 52 188.5 ...
```

```
print(d.eggs.2019, n = 5, width = Inf)
```

```
# A tibble: 327 x 14
  WGS84.LAT WGS84.LNG ALTITUDE AREA         MUNICIPALITY
      <dbl>     <dbl>    <dbl> <fct>        <fct>
1      45.8      9.00      263 Intervention Balerna
2      45.8      9.00      263 Intervention Balerna
3      45.8      9.00      263 Intervention Balerna
4      45.8      9.00      263 Intervention Balerna
5      45.8      9.00      263 Intervention Balerna
  Date.when.ovitrap.installed Date.when.ovitrap.collected
  <dttm>                      <dttm>
1 2019-05-23 00:00:00         2019-06-09 00:00:00
2 2019-06-09 00:00:00         2019-06-21 00:00:00
3 2019-07-16 00:00:00         2019-07-30 00:00:00
4 2019-07-30 00:00:00         2019-08-13 00:00:00
5 2019-08-13 00:00:00         2019-08-28 00:00:00
  No..Days.ovitrap.in.field Week.when.ovitrap.collected No..eggs.AEDES
                      <dbl>                       <dbl>          <dbl>
1                        17                          23              0
2                        12                          25             29
3                        14                          31              0
4                        14                          33             52
5                        15                          35            202
  No..Eggs.AEDES.in.14.days TRAP.ID.fac Day.ovitrap.collected
                      <dbl> <fct>                       <dbl>
1                       0   BAL-11a                       160
2                      33.8 BAL-11a                       172
3                       0   BAL-11a                       211
4                      52   BAL-11a                       225
5                     189.  BAL-11a                       240
  no.eggs.normalised.14.days
                       <dbl>
1                        0
2                       33.8
3                        0
4                       52
5                      189.
# ... with 322 more rows
```

4 Graphical Analysis

We graphically analyse the marginal effect that the available predictors have on the response variable (i.e. "number of eggs").

4.1 AREA

The predictor *AREA* defines whether the trap is to be found in a "treated" site (i.e. "Intervention") or not (i.e. "Non-intervention").

```
ggplot(data = d.eggs.2019,
                   mapping = aes(y = No..eggs.AEDES)) +
  geom_boxplot(mapping = aes(x = AREA))
```

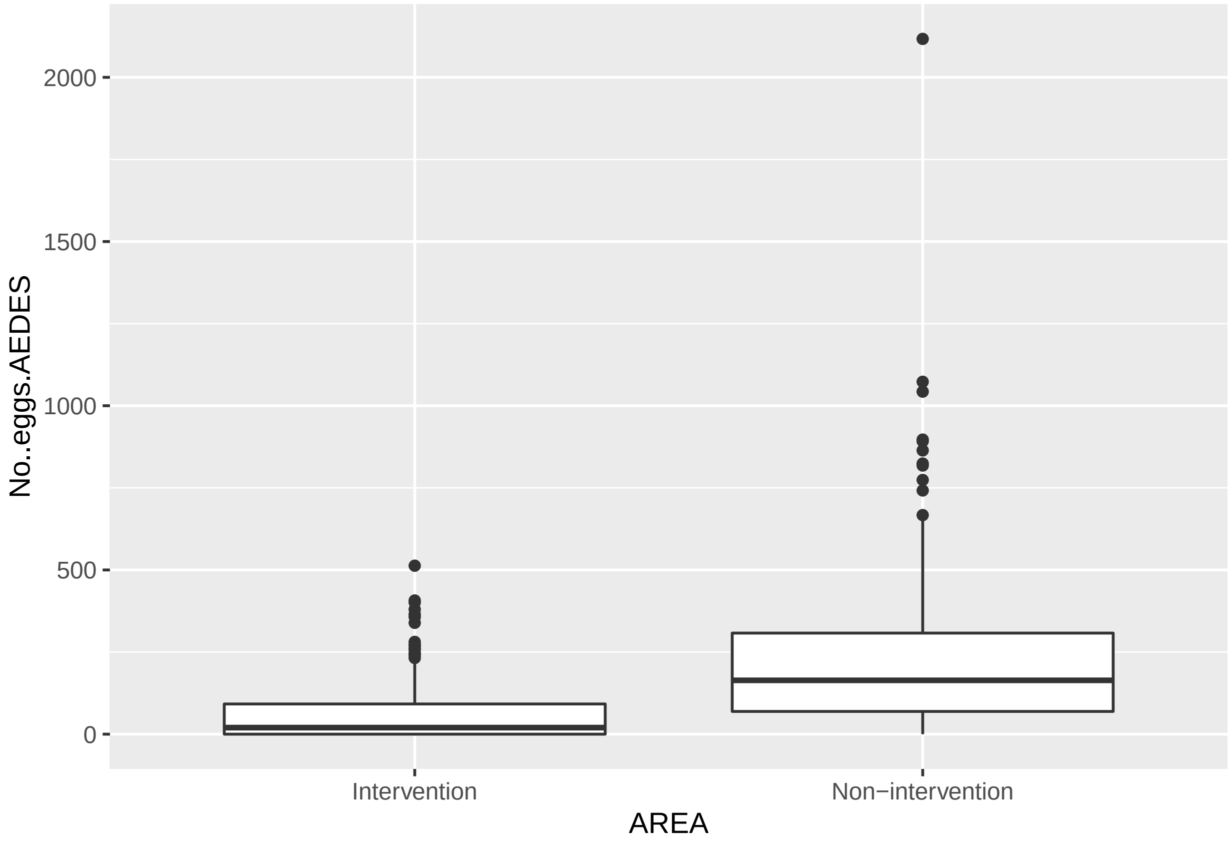

The most striking pattern on this graph is the skewness of the data. This does not come unexpected as we are dealing with count data. Therefore, as a standard practice for this type of data, we are going to visualise the effect of all predictors on the square-root-transformed response variable[1].

Note also that we will then fit a Negative Binomial model whose canonical link function is the natural logarithm. Obviously, the logarithm cannot be applied to display the raw data as it contains zeros.

Note that since very many graphs have a similar structure, we first create a "proto graph" that is then adapted for each predictor.

[1]The square-root-transformation is the "variance-stabilising transformation" for count data (CITE BOOK HELD).

```
gg.proto <- ggplot(data = d.eggs.2019,
                   mapping = aes(y = No..eggs.AEDES)) +
  scale_y_sqrt() +
  geom_hline(yintercept = 0)
```

Let's now recreate the graph for the effect of *AREA* with the response variable appropriately transformed.

```
gg.proto +
  geom_boxplot(mapping = aes(x = AREA))
```

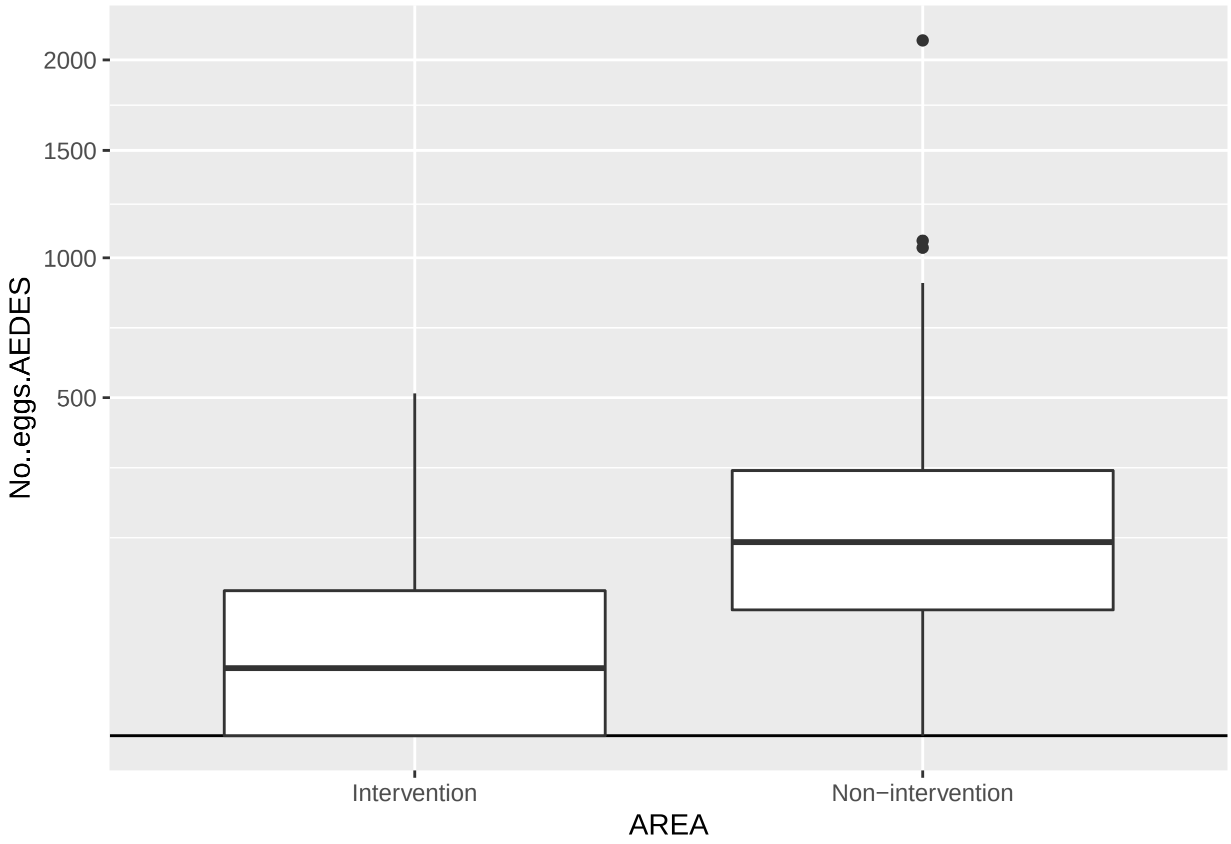

There seems to be quite a difference between the "Intervention" and the "non-intervention" groups. Note, also that the square-root transformation successfully stabilised the variance.

4.2 MUNICIPALITY

```
gg.proto +
  geom_boxplot(mapping = aes(x = MUNICIPALITY,
                             colour = AREA))
```

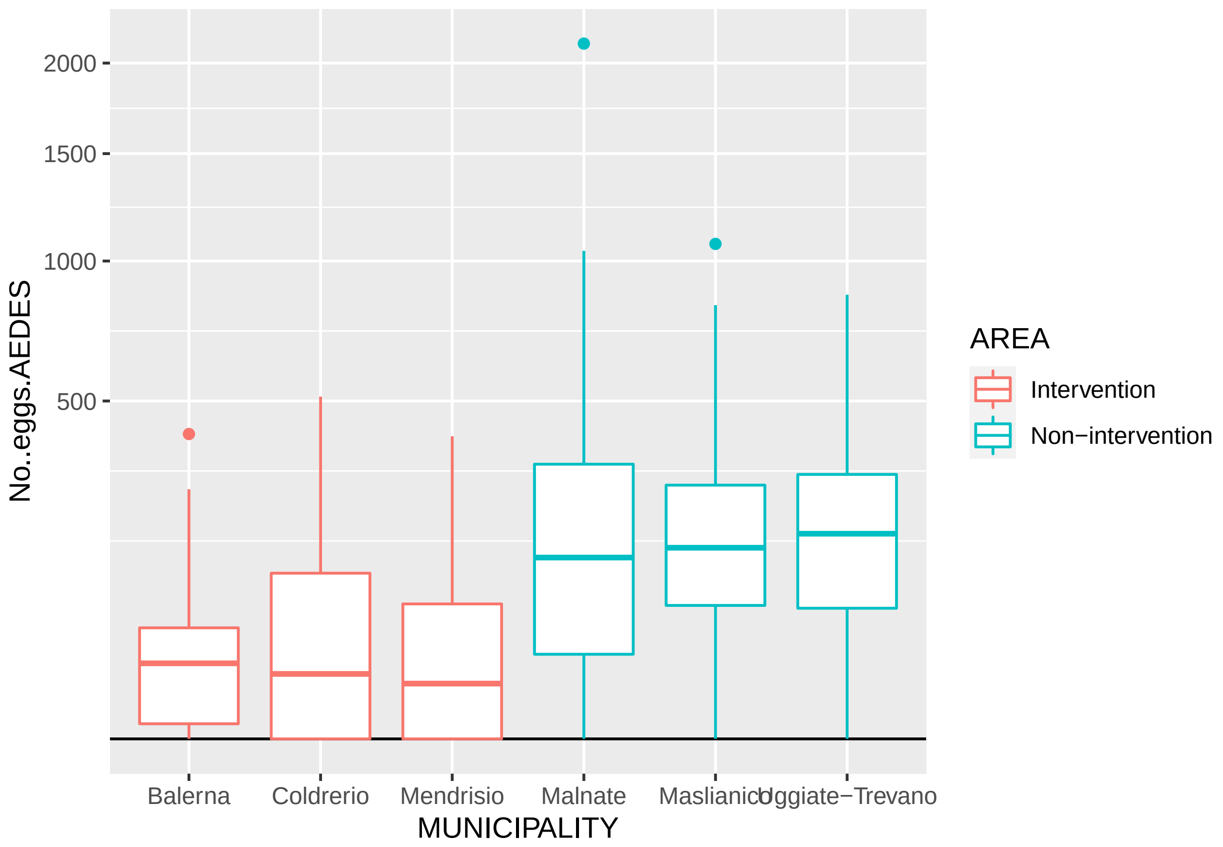

In terms of median values, there is very little variation among municipalities that belong to the same *AREA* group. In other words, the differences in median among municipalities seem to be fully explained by "Intervention"/"Non-intervention" only.

4.3 TRAP.ID.fac

```
gg.proto +
  geom_boxplot(mapping = aes(x = TRAP.ID.fac,
                             colour = MUNICIPALITY)) +
  theme(axis.text.x = element_text(angle = 90))
```

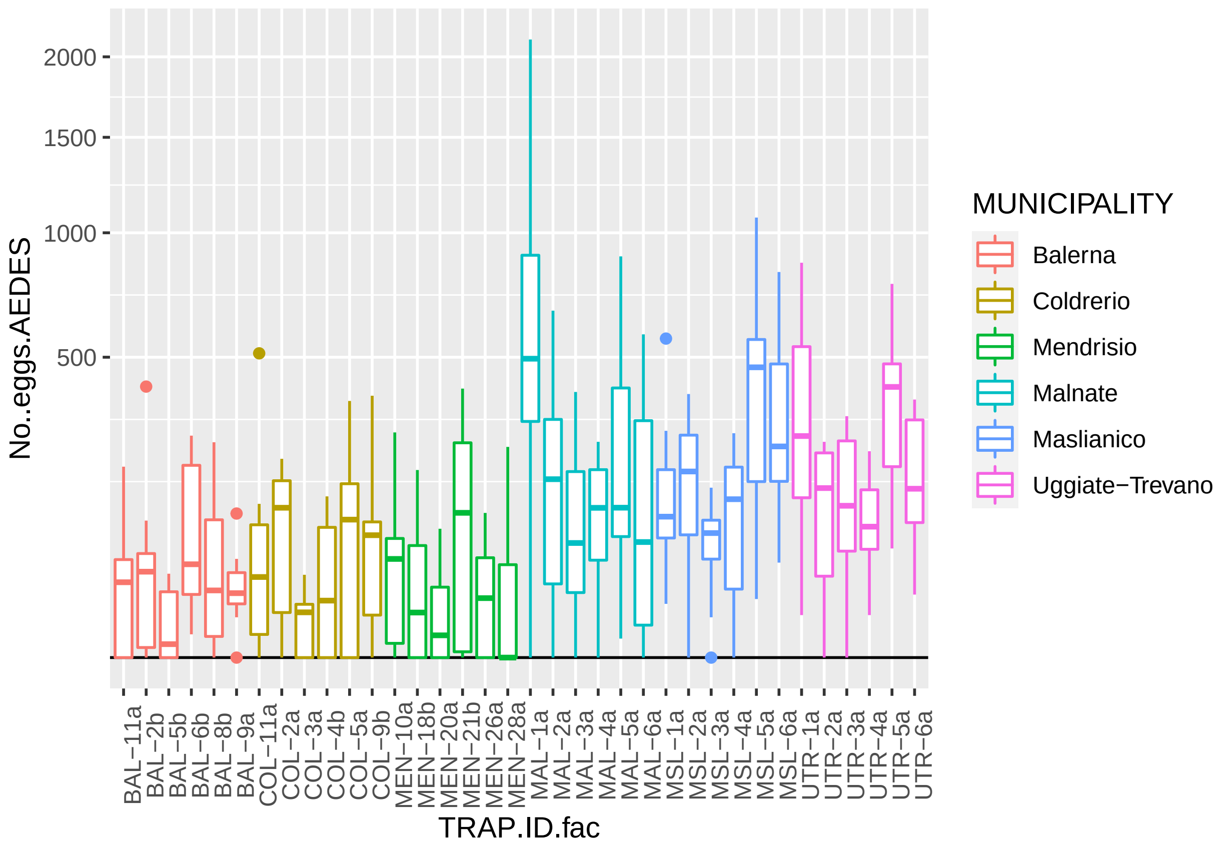

Interestingly, in terms of medians there is some non-negligible variation within municipalities (i.e. among traps of the same site). However, this variation is rather small compared to the difference among "Intervention" and "Non-intervention" traps.

4.4 Date.when.ovitrap.collected

Let's include the time component in these graphs. Here we visualise the counts for each trap over time (as a solid line). Note that panelling is used to differentiate among *AREA* levels. Indeed, as *AREA* is the predictor of main interest in this analysis, we may want to include its interactions (when needed) in the model. A smoother is added in both panels to highlight the "shared" time trend within both groups.

```
## (messages are omitted from this chunk)
##
ggplot(data = d.eggs.2019,
       mapping = aes(y = No..eggs.AEDES,
                     x = Date.when.ovitrap.collected,
                     group = TRAP.ID.fac)) +
  scale_y_sqrt(breaks = c(0,50,100,200,500,1000,2000),
               minor_breaks = FALSE) +
  geom_hline(yintercept = 0) +
  geom_line(alpha = 0.4) +
  facet_wrap(~AREA) +
  geom_smooth(mapping = aes(group = 1), colour = "#3366FF")
```

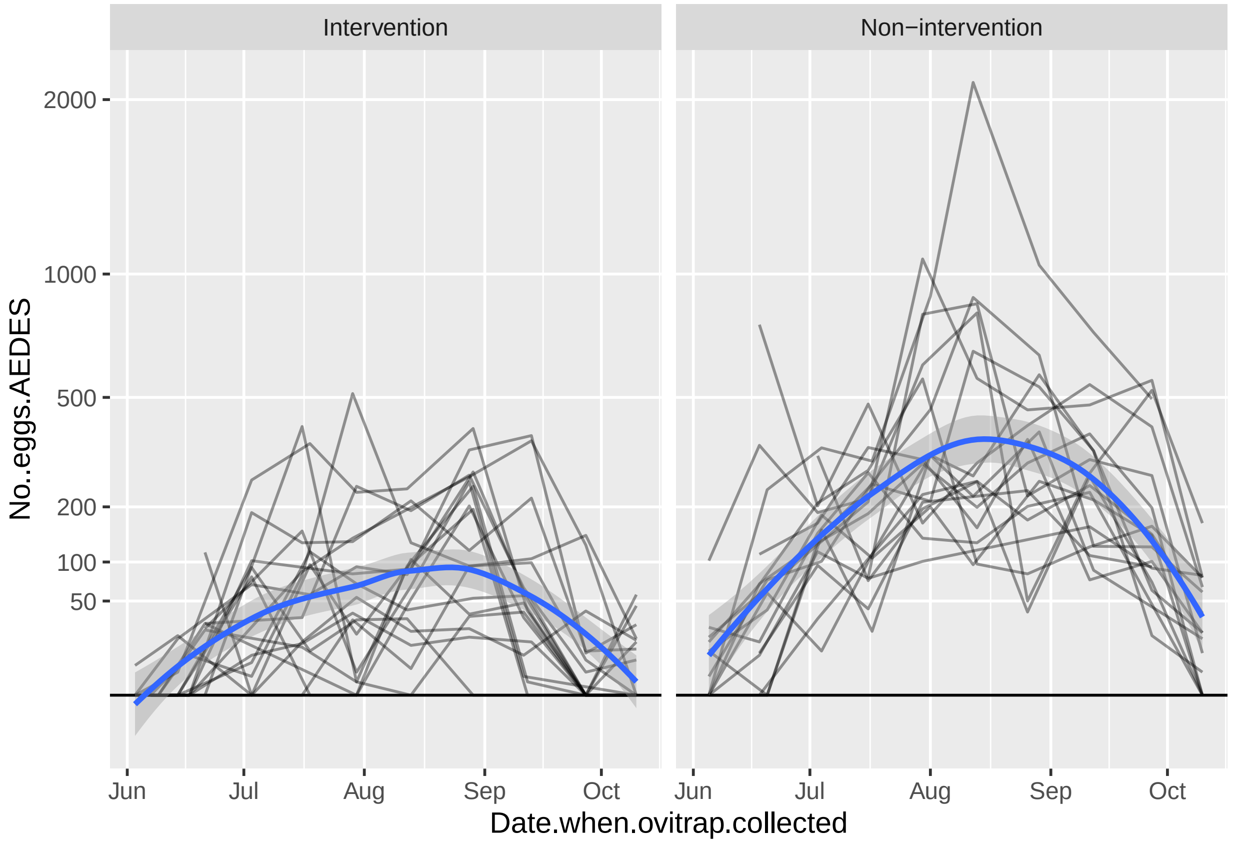

There is a clear bow-shaped seasonal pattern in both groups. This pattern is then modelled as a quadratic effect. Considering that we are fitting a multiplicative model (i.e. link function is the natural logarithm), it is possible that an interaction between *AREA* and *Date* is not needed.

For the main publication part we also reproduce the same graph in the original scale.

```
## (messages are omitted from this chunk)
##
ggplot(data = d.eggs.2019,
       mapping = aes(y = No..eggs.AEDES,
                     x = Date.when.ovitrap.collected,
                     group = TRAP.ID.fac)) +
  # scale_y_sqrt() + ## Not sqrt-transformed
  geom_hline(yintercept = 0) +
  geom_line(show.legend = FALSE,
            alpha = 0.4) +
  facet_wrap(~AREA) +
  geom_smooth(mapping = aes(group = 1), colour = "#3366FF")
```

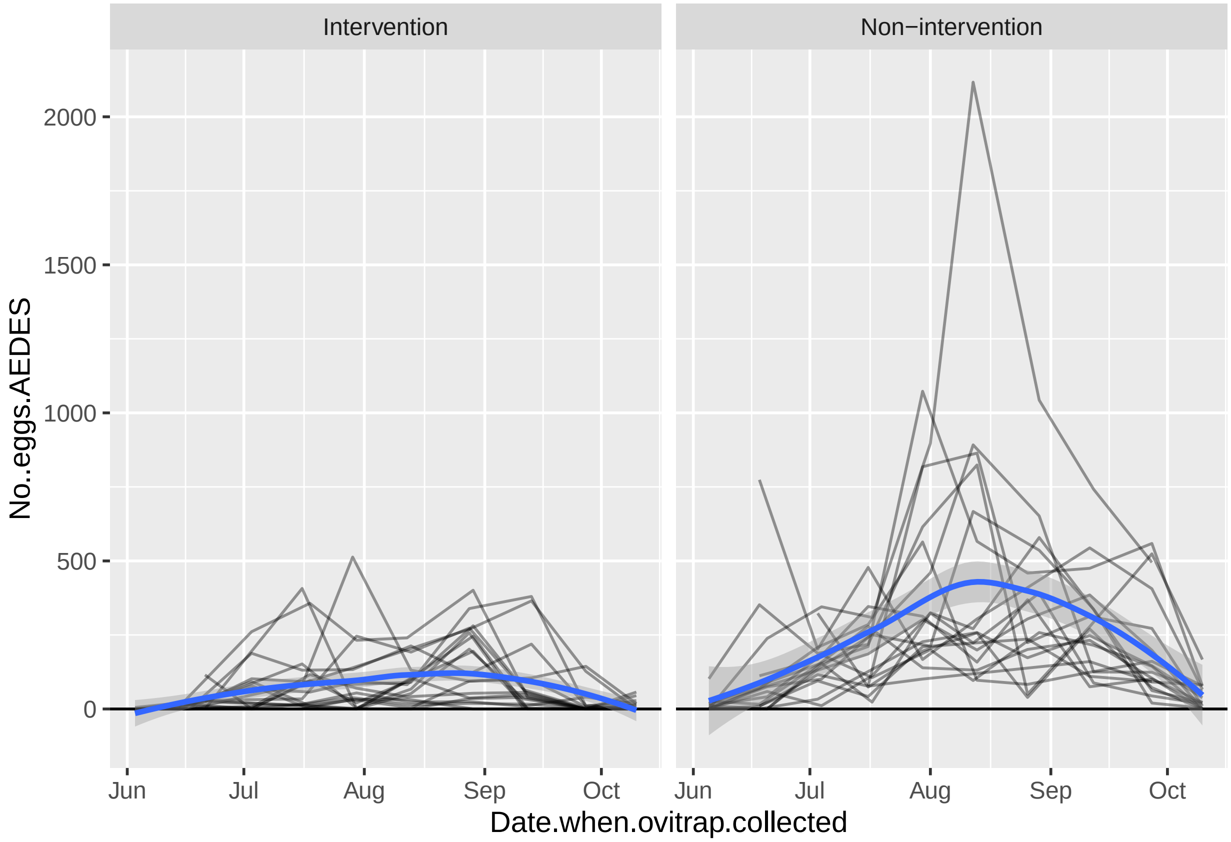

The actual counts highlights the dramatic difference between the the two treatments.

Note that x-axis here reports the date of the ovitrap collection. For the modelling phase, we are going to use the "Day of the year" as a predictor. Using one or the other is fully equivalent. However, Dates makes graphs more readable (as months are reported). While using a numeric variable for modelling is simpler than using a Date object.

4.5 No..Days.ovitrap.in.field

Each trap was not left the exact same number of days in the field. Therefore, we may want to account for the "exposure" effect. In theory, we would expect that traps that are left longer in the field contain more eggs.

```
## (warnings and messages are omitted from this chunk)
##
gg.proto +
  aes(x = No..Days.ovitrap.in.field) +
  geom_point() +
  geom_smooth() +
  facet_wrap(~ AREA)
```

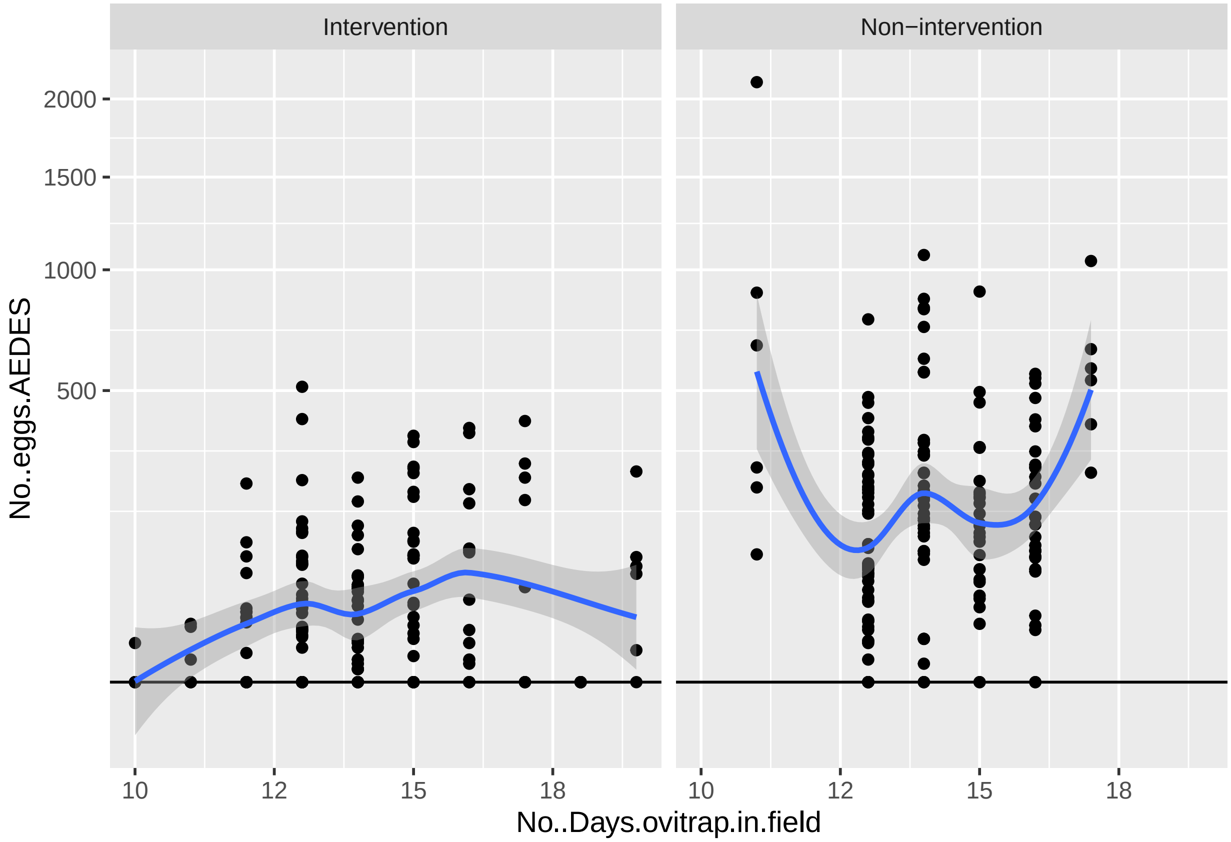

There is no clear effect of this predictor on the response variable.

4.6 ALTITUDE

Let's visualise the marginal effect of altitude, while panelling for *AREA*.

```
## (messages are omitted from this chunk)
##
gg.proto +
  aes(x = ALTITUDE) +
  geom_point() +
  geom_smooth() +
  facet_wrap(. ~ AREA)
```

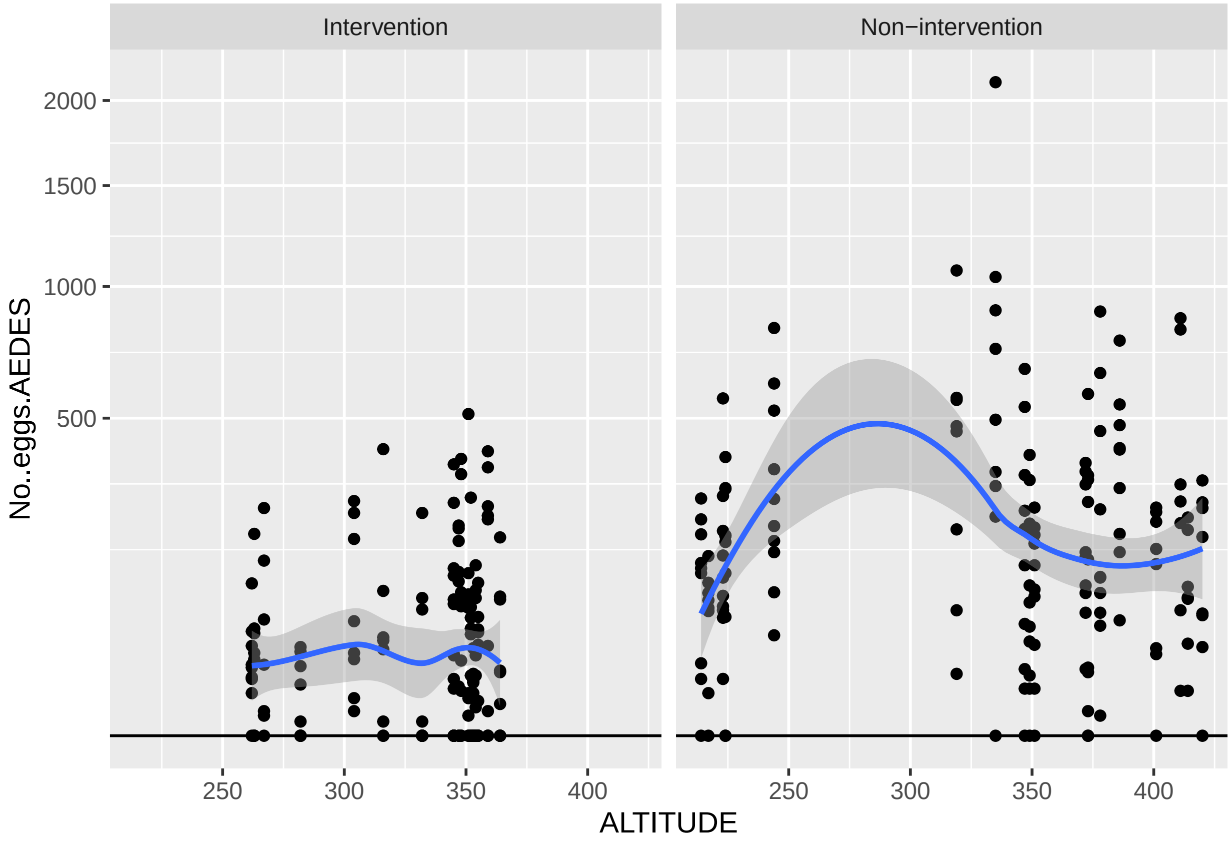

Altitude does not seem to have a clear effect either.

4.7 Spatial structure

Let's visualise the spatial structure of the 36 traps. We use colours to discriminate between "Intervention" and "Non-intervention" sites.

```
xyplot(WGS84.LAT ~ WGS84.LNG,
       data = d.eggs.2019,
       groups = AREA,
       type = c("p", "g"),
       auto.key = TRUE,
       aspect = 1)
```

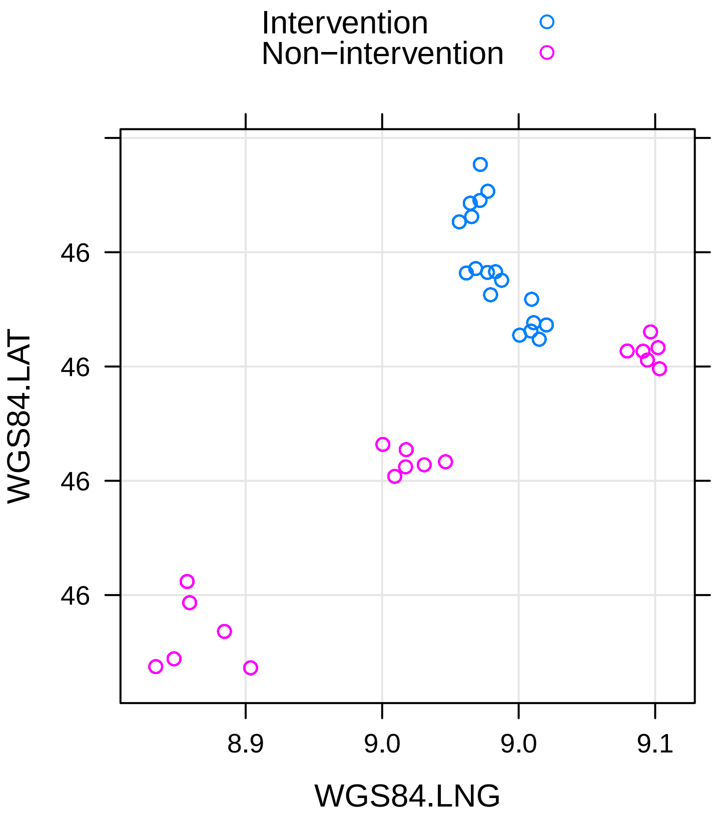

Let's now highlight the six municipalities.

```
xyplot(WGS84.LAT ~ WGS84.LNG,
       data = d.eggs.2019,
       groups = MUNICIPALITY,
       type = c("p", "g"),
       auto.key = list(space = "right"),
       aspect = 1)
```

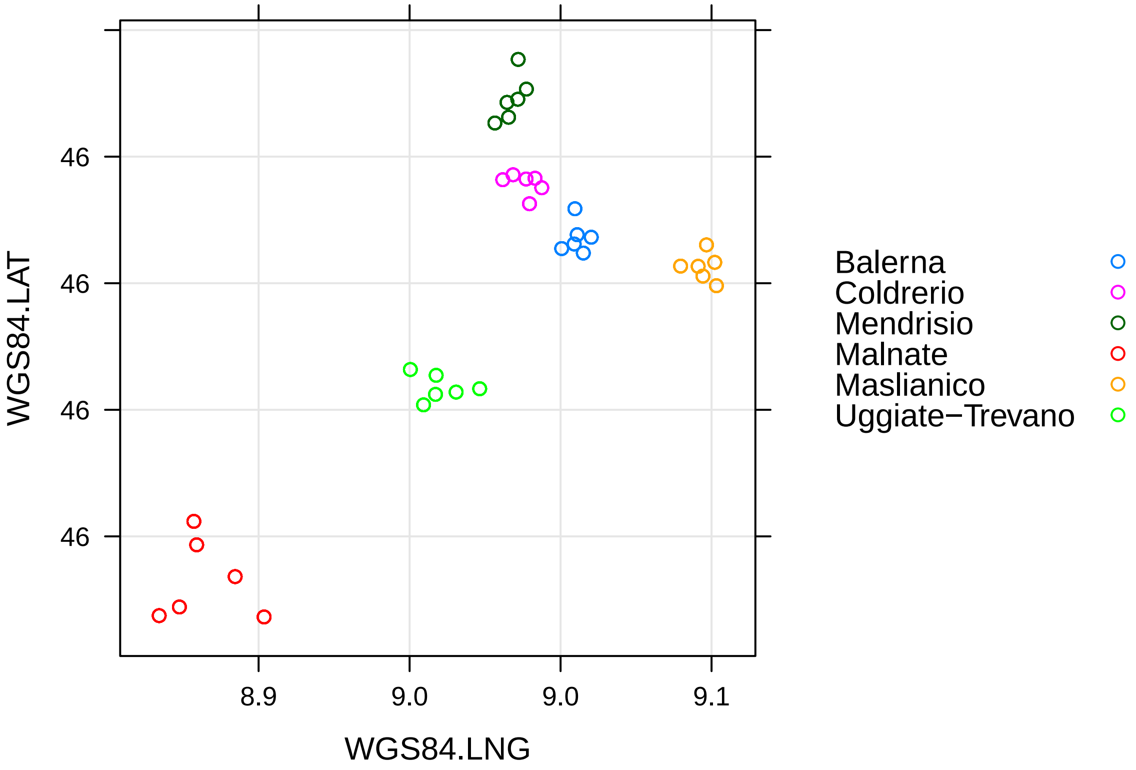

There are six traps in each municipality. Each trap is measured 10 times.

5 Modelling

5.1 Generalised Mixed-Effects Models

Below we implement the solution that accounts for overdispersion via the negative binomial family. We use the *glmmTMB()* function found in the *{glmmTMB}* package. Note that in this model we can include all nested predictors with no problems of non-identifiability (i.e. rank-deficiency). In particular, *AREA* that is the predictor of main interest, is included as a fixed effect, while *MUNICIPALITY* and *TRAP.ID.fac* are taken as random effects. Note that *ALTITUDE* is scaled to fasten model fitting.

Note that to model the time effect we use orthogonal polynomials (i.e. the *poly()* function) such that collinearity issues are avoided.

```
## (warning are omitted from this chunk)
##
mod.nb.1 <- glmmTMB(No..eggs.AEDES ~ AREA +
                      poly(Day.ovitrap.collected, degree = 2) +
                      scale(ALTITUDE) +
                      No..Days.ovitrap.in.field +
                      (1 | TRAP.ID.fac) + (1 | MUNICIPALITY),
                    family = "nbinom1",
                    data = d.eggs.2019)
```

```r
##
summary(mod.nb.1)
```

```
 Family: nbinom1  ( log )
Formula:
No..eggs.AEDES ~ AREA + poly(Day.ovitrap.collected, degree = 2) +
    scale(ALTITUDE) + No..Days.ovitrap.in.field + (1 | TRAP.ID.fac) +
    (1 | MUNICIPALITY)
Data: d.eggs.2019

     AIC      BIC   logLik deviance df.resid
    3336     3370    -1659     3318      318

Random effects:

Conditional model:
 Groups       Name        Variance Std.Dev.
 TRAP.ID.fac  (Intercept) 1.76e-01 4.20e-01
 MUNICIPALITY (Intercept) 4.51e-09 6.72e-05
Number of obs: 327, groups:  TRAP.ID.fac, 36; MUNICIPALITY, 6

Overdispersion parameter for nbinom1 family ():  121

Conditional model:
                                         Estimate Std. Error z value Pr(>|z|)
(Intercept)                                2.4370     0.4678    5.21  1.9e-07
AREANon-intervention                       1.3387     0.1723    7.77  7.8e-15
poly(Day.ovitrap.collected, degree = 2)1   3.9889     1.0696    3.73  0.00019
poly(Day.ovitrap.collected, degree = 2)2 -15.5900     1.0701  -14.57  < 2e-16
scale(ALTITUDE)                            0.0415     0.0807    0.51  0.60690
No..Days.ovitrap.in.field                  0.0909     0.0307    2.96  0.00310

(Intercept)                              ***
AREANon-intervention                     ***
poly(Day.ovitrap.collected, degree = 2)1 ***
poly(Day.ovitrap.collected, degree = 2)2 ***
scale(ALTITUDE)
No..Days.ovitrap.in.field                **
---
Signif. codes:  0 '***' 0.001 '**' 0.01 '*' 0.05 '.' 0.1 ' ' 1
```

The model fits in a few seconds and there are no convergence problems. Note that the warnings omitted here are harmless (see developers comments about these warnings here).

As expected from the graphical analysis, the estimated variance of the *MUNICIPALITY* random effect seems to be very small. Let's inspect this further.

```r
options(scipen = 999) ## to drop scientific notation
VarCorr(mod.nb.1)
```

```
Conditional model:
 Groups       Name        Std.Dev.
 TRAP.ID.fac  (Intercept) 0.4196126
 MUNICIPALITY (Intercept) 0.0000672
```

```
options(scipen = 0) ## to set it back to default
```

The variability of *MUNICIPALITY* is extremely small compared to *TRAP.ID.fac* and it is even close to zero, which is the boundary of the parameter space. Given that *MUNICIPALITY* is a design variable and the model converges we don't drop it from the model.

5.2 Visualising the model fit

We start by visualising the fitted values of the model (in red) along with the observed values (in gray). Note that all these plots still use a square-root transformed y-axis.

```
## (warning are omitted from this chunk)
##
d.eggs.2019$fit.mod.nb.1 <- fitted(mod.nb.1)
##
ggplot(data = d.eggs.2019,
       mapping = aes(y = No..eggs.AEDES,
                     x = Date.when.ovitrap.collected,
                     group = TRAP.ID.fac)) +
  scale_y_sqrt() +
  geom_line(alpha = 0.2) +
  facet_wrap(. ~ AREA) +
  geom_line(mapping = aes(y = fit.mod.nb.1),
            col = "red",
            alpha = 0.8)
```

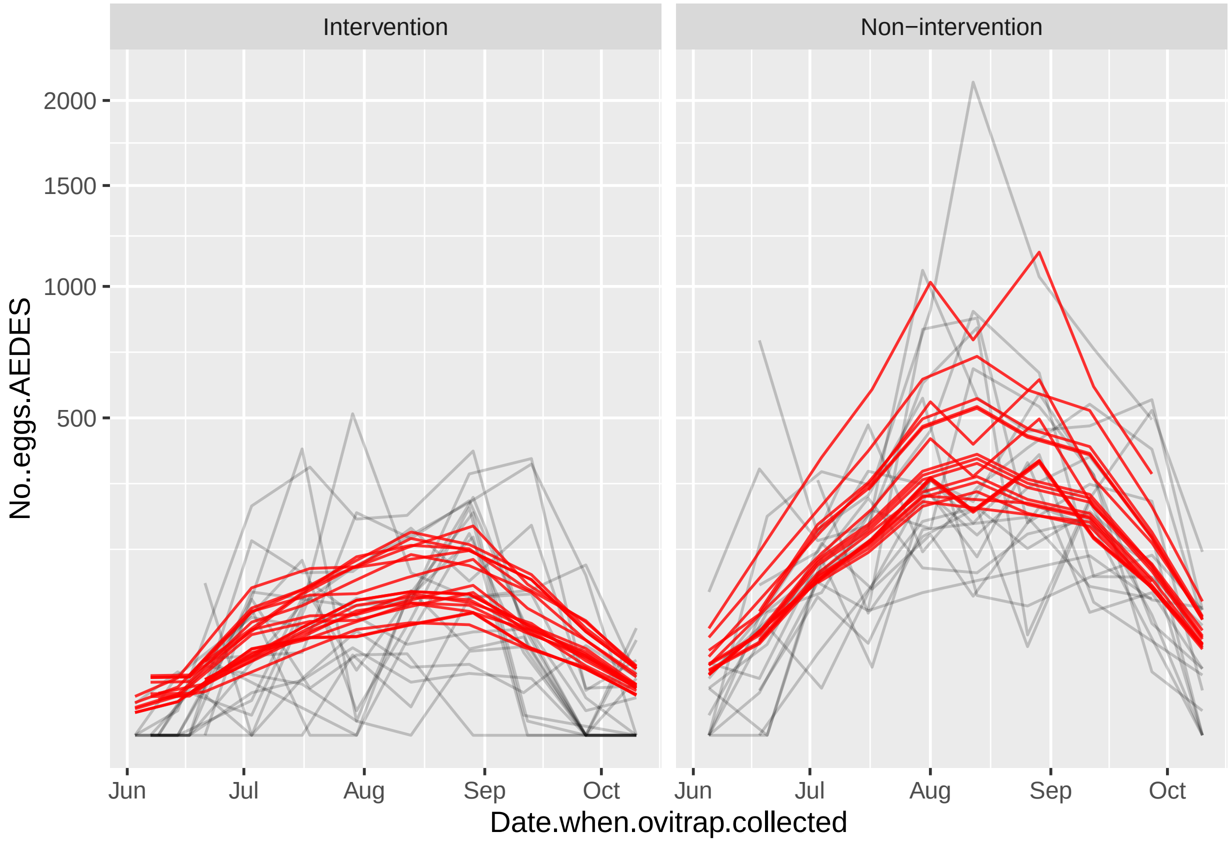

Note that the predicted values for each trap are not nice and quadratic because i) we are evaluating the model at 10 discrete dates only (i.e. the observed dates) and ii) because the model also contains the continuous predictor *No..Days.ovitrap.in.field* that varies over time.

Note also that the fitted value shown here are not adjusted to correct for bias due to Jensen's inequality. This applies to all graphs produced with fitted or predicted values here.

In the next graph we are going to show the predictions at population level (i.e. without the estimated random effects). In addition, we keep *No..Days.ovitrap.in.field* and *ALTITUDE* constant at their mean values and we make predictions on a finer grid (i.e. daily predictions).

```
## (results and warnigns re omitted from this chunk)
##
## 1. Creating the prediction dataset with expand.grid()
d.predict <- expand.grid(
  AREA = levels(d.eggs.2019$AREA),
  Date.when.ovitrap.collected = seq(
    from = min(d.eggs.2019$Date.when.ovitrap.collected),
    to = max(d.eggs.2019$Date.when.ovitrap.collected),
    by = "day"),
  ALTITUDE = mean(d.eggs.2019$ALTITUDE),
  No..Days.ovitrap.in.field = mean(
    d.eggs.2019$No..Days.ovitrap.in.field),
  ## NB: predict.glmmTMB() requires RE even for pop-level predictions.
  TRAP.ID.fac = "BAL-11a",
```

```
  MUNICIPALITY = "Balerna")
##
dim(d.predict)
str(d.predict)
##
## 2. Converting Date to Day
d.predict$Day.ovitrap.collected <- yday(d.predict$Date.when.ovitrap.collected)
##
dim(d.predict)
str(d.predict)
##
## 3. Making predictions at population level
d.predict$pred.pop.level.mod.nb.1 <- predict(mod.nb.1,
                                   type = "response",
                                   newdata = d.predict,
                                   re.form = NA)
##
## 4. Producing the graph
ggplot(data = d.eggs.2019,
       mapping = aes(y = No..eggs.AEDES,
                     x = Date.when.ovitrap.collected,
                     group = TRAP.ID.fac)) +
  scale_y_sqrt() +
  geom_line(alpha = 0.2) +
  facet_wrap(. ~ AREA) +
  ## adding pop-level predictions
  geom_line(data = d.predict,
            size = 1,
            mapping = aes(y = pred.pop.level.mod.nb.1),
            col = "red")
```

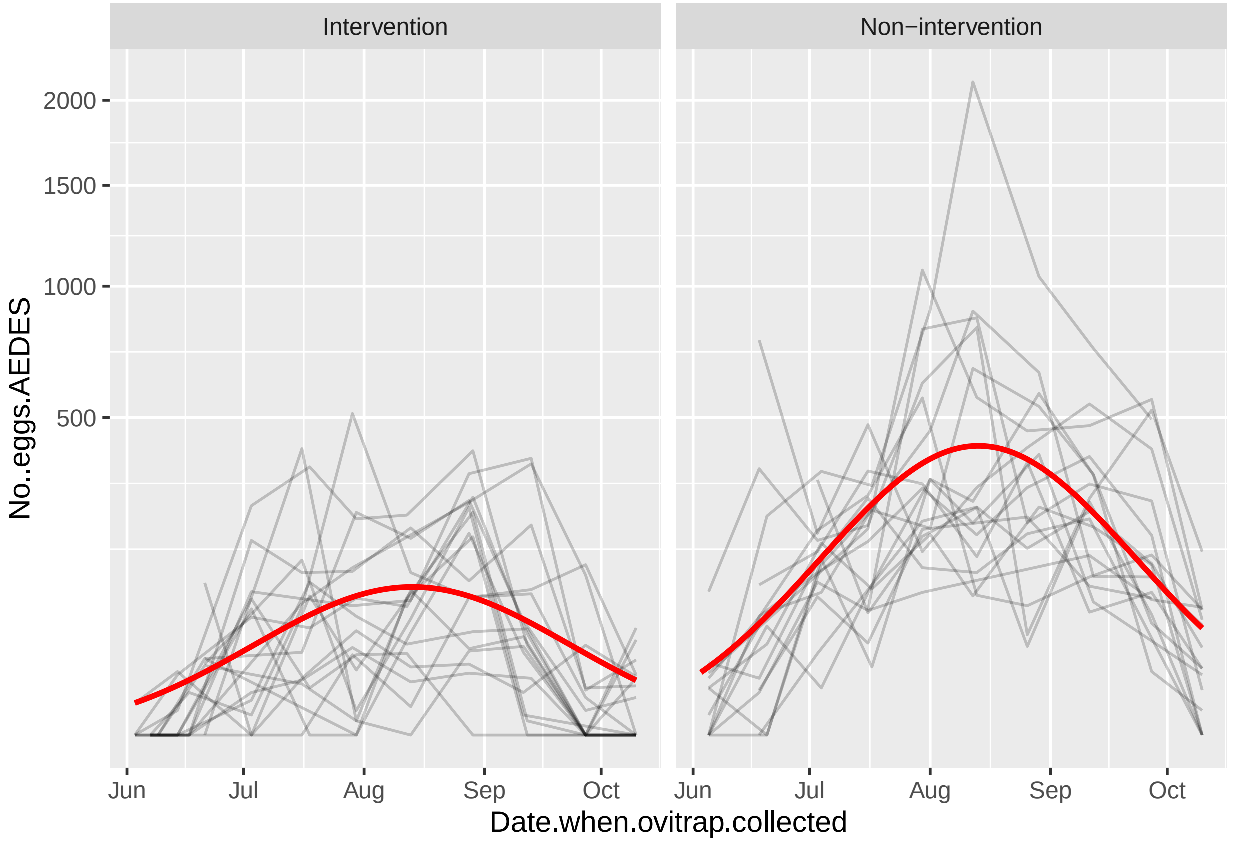

The fit looks sensible and the difference among the two groups is quite clear.

Side remark: Note that the fit does not look perfectly quadratic because the fit is done in the natural logarithm scala (see link function).

To better appreciate the group differences, we visualise the fit in the original scala (i.e. without any transformation).

```
ggplot(data = d.eggs.2019,
       mapping = aes(y = No..eggs.AEDES,
                     x = Date.when.ovitrap.collected,
                     group = TRAP.ID.fac)) +
  # scale_y_sqrt() +  ## No sqrt-root transformation
  geom_line(alpha = 0.2) +
  facet_wrap(. ~ AREA) +
  geom_line(data = d.predict,
            size = 1,
            mapping = aes(y = pred.pop.level.mod.nb.1),
            col = "red")
```

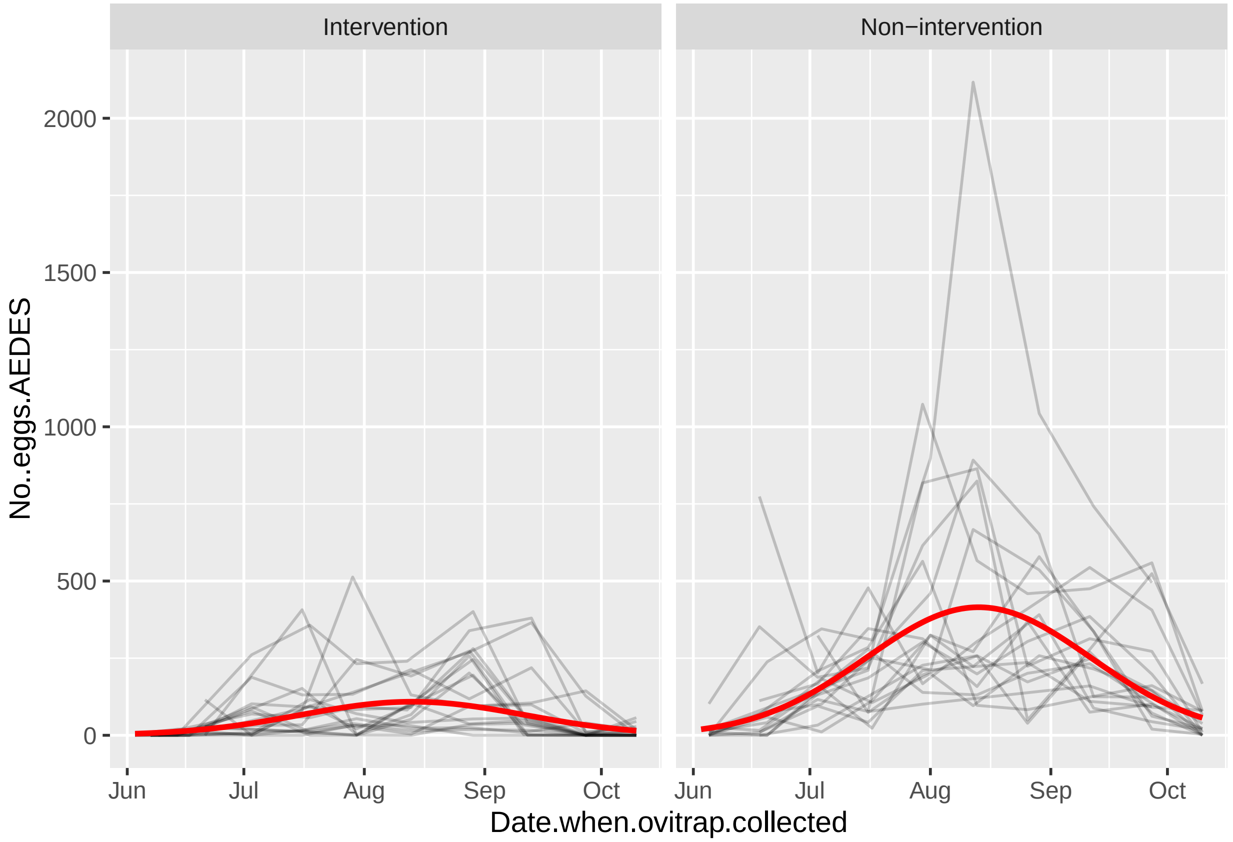

In the original scala, the difference between "intervention" and "Non-intervention" is dramatic.

5.3 Quantifying effects

Let's quantify the effect of *Non-intervention*.

```
exp(fixef(mod.nb.1)$cond["AREANon-intervention"])
```

```
AREANon-intervention
                 3.8
```

In the "Non-intervention" sites there are about four times more mosquitos than in the "Intervention" sites.

Let's compute a confidence interval for this estimate. Note that we are using profiling likelihood methods here.

```
## (this chunk is cached)
##
prof.mod.nb.1 <- profile(mod.nb.1, parm = "AREANon-intervention")
##
exp(confint(prof.mod.nb.1))
```

```
                      2.5 % 97.5 %
AREANon-intervention   2.7    5.4
```

The 95% confidence interval for this parameter ranges from 2.7 to 5.4. There is thus strong evidence that an important difference is present between "Intervention" and "Non-intervention" sites.

5.4 Model checking

5.4.1 Checking the model equation

Let's check the assumption that, in the linear predictor scale, the effect of *Day.ovitrap.collected* is quadratic and additive with the effect of *AREA*. To test hypotheses we use the "Pearson" residuals (the *residuals.glmmTMB()* method only implements "pearson" and "response" residuals).

```
## (messages and warnigns are omitted from this chunk)
##
d.eggs.2019$res.mod.nb.1 <- resid(mod.nb.1, type = "pearson")
##
ggplot(data = d.eggs.2019,
       mapping = aes(y = res.mod.nb.1,
                     x = Date.when.ovitrap.collected)) +
  geom_hline(yintercept = 0) +
  geom_point() +
  facet_wrap(. ~ AREA) +
  geom_smooth()
```

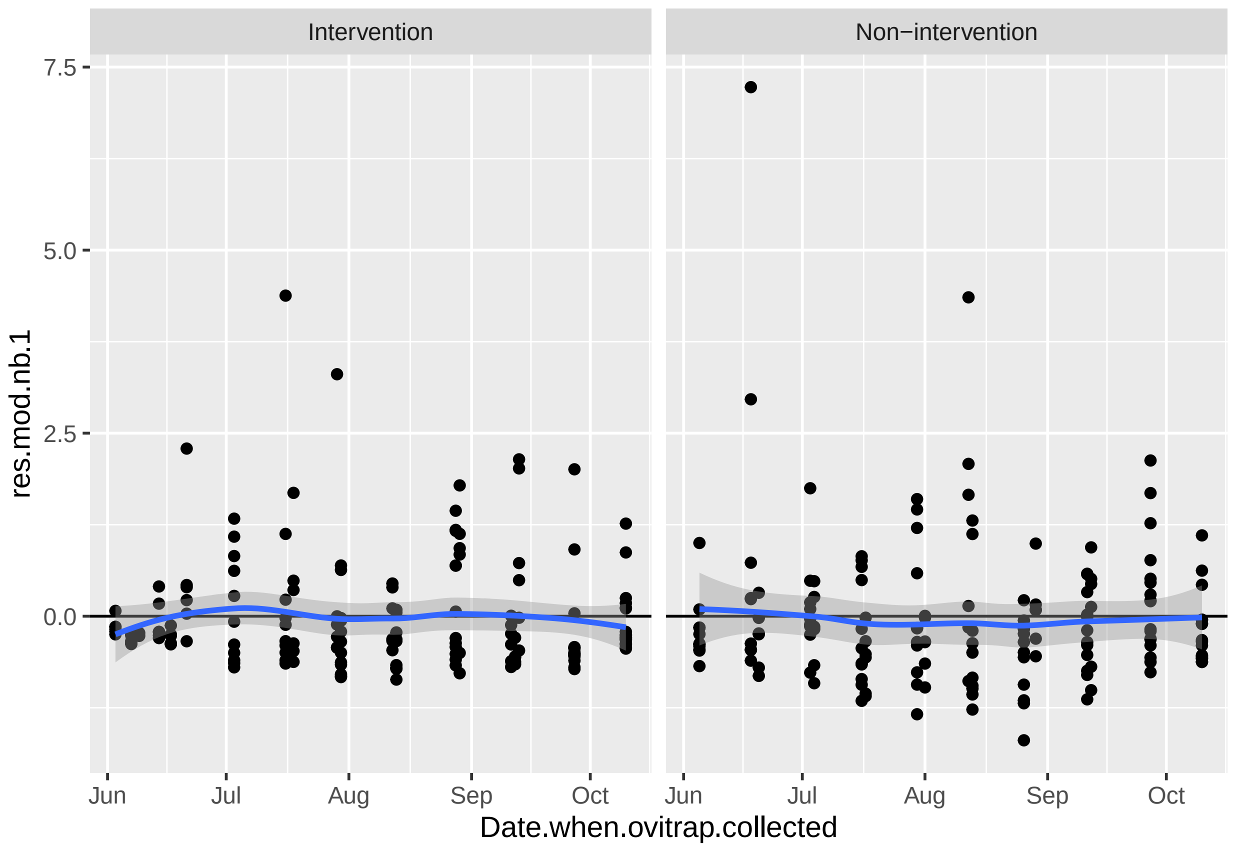

There is no indication that the quadratic effect is not enough to model the non-linear effect of “Date” and there is no indication that an interaction between *AREA* and *Day* is needed.

To confirm this, we can formally check whether the two-fold interaction betwen *AREA* and *Day* is needed with a Likelihood Ratio Test.

```
## (warning are omitted from this chunk)
##
mod.nb.2 <- update(mod.nb.1, . ~ . +
                     AREA:poly(Day.ovitrap.collected, degree = 2))
##
anova(mod.nb.1,
      mod.nb.2, test = "Chisq")
```

```
Data: d.eggs.2019
Models:
mod.nb.1: No..eggs.AEDES ~ AREA + poly(Day.ovitrap.collected, degree = 2) + , zi=~0, disp=~1
mod.nb.1:     scale(ALTITUDE) + No..Days.ovitrap.in.field + (1 | TRAP.ID.fac) + , zi=~0, disp=~1
mod.nb.1:     (1 | MUNICIPALITY), zi=~0, disp=~1
mod.nb.2: No..eggs.AEDES ~ AREA + poly(Day.ovitrap.collected, degree = 2) + , zi=~0, disp=~1
mod.nb.2:     scale(ALTITUDE) + No..Days.ovitrap.in.field + (1 | TRAP.ID.fac) + , zi=~0, disp=~1
mod.nb.2:     (1 | MUNICIPALITY) + AREA:poly(Day.ovitrap.collected, degree = 2), zi=~0, disp=~1
         Df  AIC  BIC logLik deviance Chisq Chi Df Pr(>Chisq)
mod.nb.1  9 3336 3370  -1659     3318
mod.nb.2 11 3336 3378  -1657     3314  4.03      2       0.13
```

The formal test confirms that an interaction is not needed.

5.4.2 Checking the structure of the random effects

Let's now check at trap level whether assuming a simple random intercept is enough to model these short time series. In other words, we are graphically testing whether time correlation is present.

```
ggplot(data = d.eggs.2019,
       mapping = aes(y = res.mod.nb.1,
                     x = Date.when.ovitrap.collected)) +
  geom_hline(yintercept = 0) +
  geom_line() +
  geom_point() +
  facet_wrap(. ~ TRAP.ID.fac, scales = "free_y") +
  theme(strip.text.x = element_blank(),
        axis.text.y = element_blank(),
        axis.ticks.y = element_blank(),
        axis.text.x = element_text(angle = 45))
```

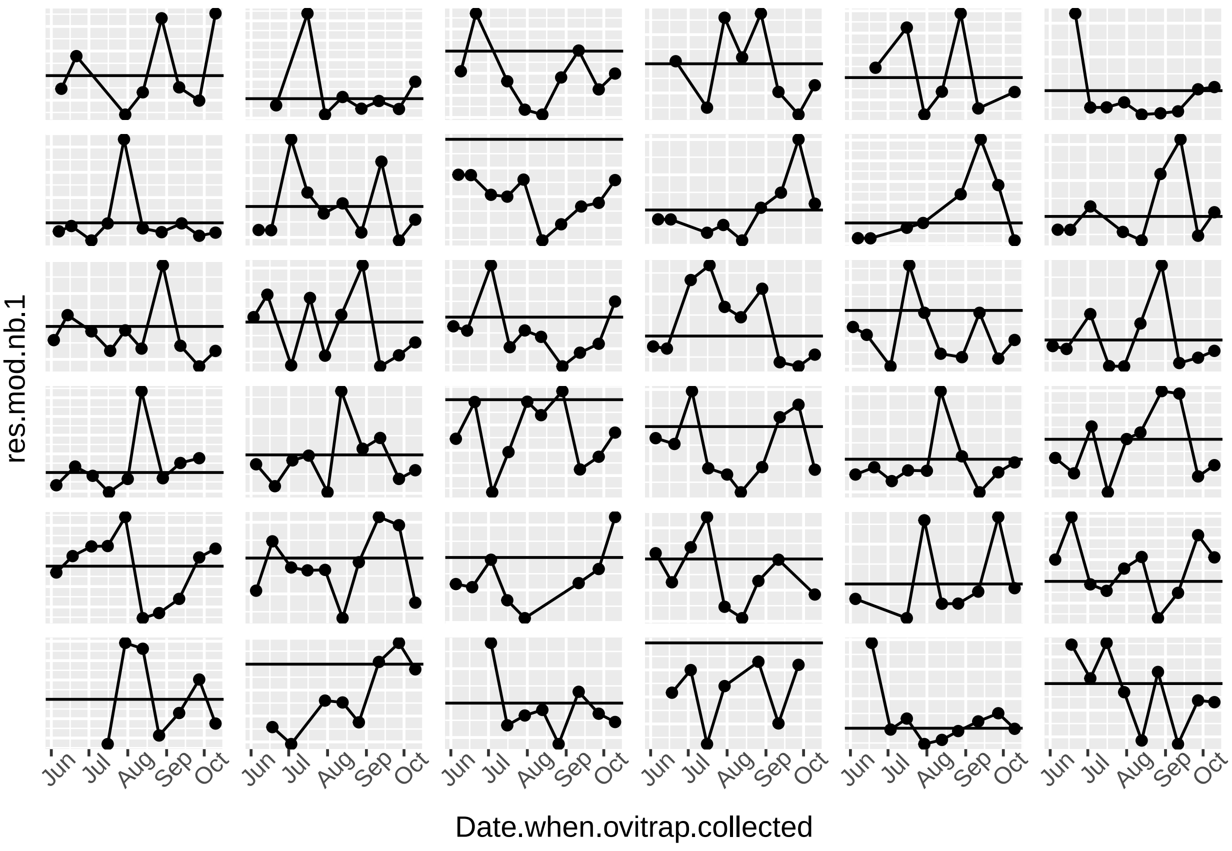

There is no evidence of temporal structure left at trap level.

5.4.3 Checking normality of the random effects

The negative binomial model we fitted assumes a normal distribution of the random effects (in the linear predictor space).

```
par(mfrow = c(1,2))
##
qqnorm(unlist(ranef(mod.nb.1)$cond$TRAP.ID.fac))
qqline(unlist(ranef(mod.nb.1)$cond$TRAP.ID.fac))
##
qqnorm(unlist(ranef(mod.nb.1)$cond$MUNICIPALITY))
qqline(unlist(ranef(mod.nb.1)$cond$MUNICIPALITY))
```

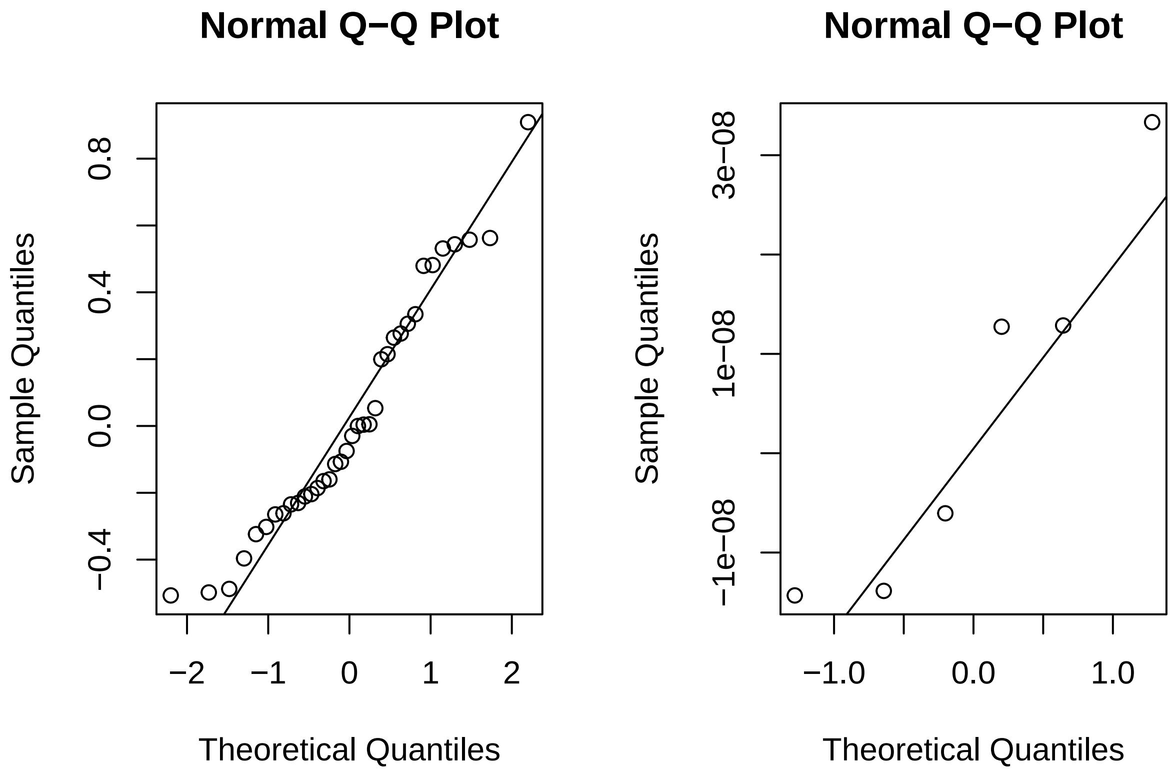

The normality assumption about the random effects does not appear to be violated for traps. Little can be said for *MUNICIPALITY* that has only six levels.

5.4.4 Checking the mean-variance relationship assumption

The model fitted here assumes that the variance of the observations increases linearly with the mean value (i.e. family is “nbinom1”). Let’s formally check this.

```
## (warning are omitted from this chunk)
##
```

```r
mod.nb.1.quad <- update(mod.nb.1, family = "nbinom2")
##
AIC(mod.nb.1, mod.nb.1.quad)
```

```
              df  AIC
mod.nb.1       9 3336
mod.nb.1.quad  9 3495
```

```r
BIC(mod.nb.1, mod.nb.1.quad)
```

```
              df  BIC
mod.nb.1       9 3370
mod.nb.1.quad  9 3529
```

Indeed, both information criteria agree with the current choice of the family.

5.4.5 Checking whether zero-inflation is needed

We first check graphically whether the zero-inflation is present. Note that we are simulating from the fitted model.

```r
set.seed(2)
##
xy.1 <- densityplot(~sqrt(simulate(mod.nb.1)),
                    xlim = c(-10, 50),
                    ylim = c(0, 0.06),
                    main = "Simulated from model")
##
xy.2 <- densityplot(~sqrt(d.eggs.2019$No..eggs.AEDES),
                    xlim = c(-10, 50),
                    ylim = c(0, 0.06),
                    main = "Observed data")
##
plot(xy.1, split = c(1,1, 2,1))
plot(xy.2, split = c(2,1, 2,1), newpage = FALSE)
```

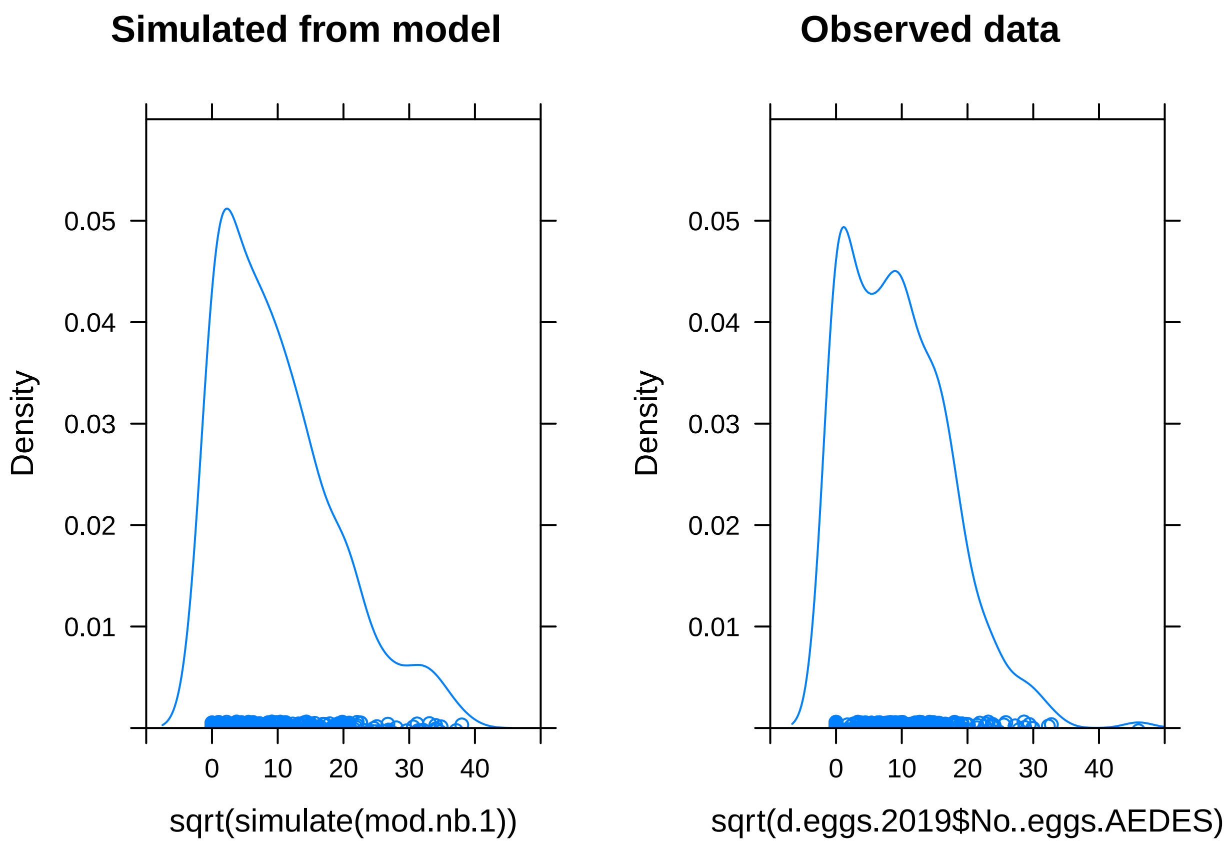

This graphs indicates that some very little zero-inflation may be present.

Let's now fit the zero-inflated models and compare the information criteria.

```
## (warning are omitted from this chunk)
##
trunc.mod.nb.1.linear <- update(mod.nb.1,
                                family = "truncated_nbinom1",
                                ziformula = ~ .)
trunc.mod.nb.1.quadratic <- update(mod.nb.1,
                                family = "truncated_nbinom2",
                                ziformula = ~ .)
##
AIC(mod.nb.1, trunc.mod.nb.1.linear, trunc.mod.nb.1.quadratic)
```

```
                         df  AIC
mod.nb.1                  9 3336
trunc.mod.nb.1.linear    17   NA
trunc.mod.nb.1.quadratic 17 3343
```

```
BIC(mod.nb.1, trunc.mod.nb.1.linear, trunc.mod.nb.1.quadratic)
```

```
                         df  BIC
mod.nb.1                  9 3370
trunc.mod.nb.1.linear    17   NA
trunc.mod.nb.1.quadratic 17 3407
```

The ZI model with the linear parametrisation does not converge. The ZI model with the quadratic parametrisation. Both information criteria agree that zero-inflation is not needed.

5.4.6 Graphically evaluating the goodness-of-fit

Observed values and fitted values are correlated to quantify the goodness of fit. This is the graphical equivalent of an R^2. Note that fitted values do include the estimated random effects here. Due to the skewness of the data, the graph is produced in the quare-root-transformed space as well.

```
gg.obsPred.NB <- ggplot(data = d.eggs.2019,
       mapping = aes(y = No..eggs.AEDES,
                     x = fit.mod.nb.1)) +
  geom_point() +
  geom_abline(intercept = 0, slope = 1) +
  ggtitle("NB: Obs vs. predicted") +
  coord_fixed()
##
gg.obsPred.NB.Sqrt <- gg.obsPred.NB +
  scale_y_sqrt() + scale_x_sqrt() +
  ggtitle("NB: Obs vs. predicted -- sqrt()") +
  coord_fixed()
```

```
Coordinate system already present. Adding new coordinate system, which will replace the existing one.
```

```
##
grid.arrange(gg.obsPred.NB, gg.obsPred.NB.Sqrt, ncol = 2)
```

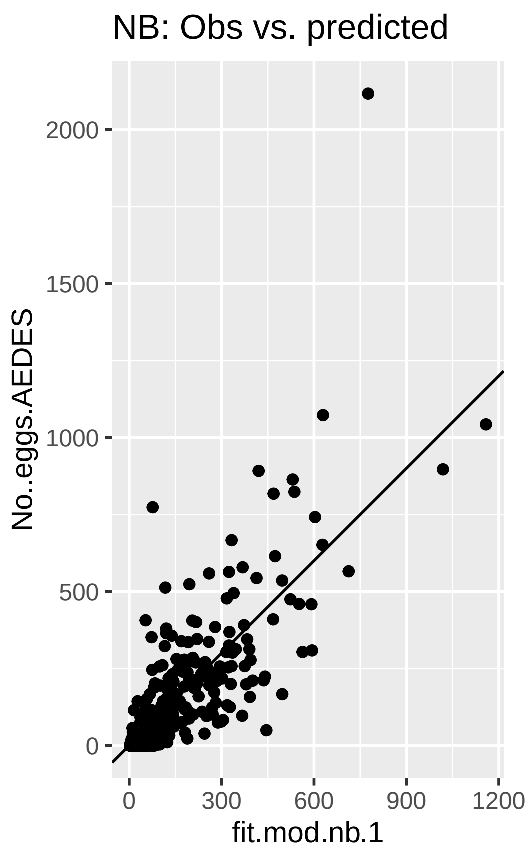

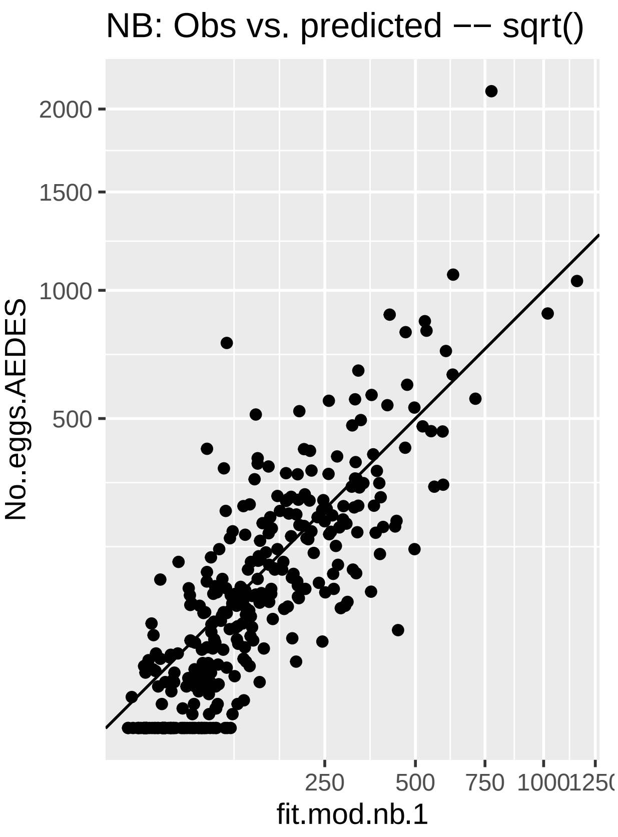

The model fit looks quite reasonable.

6 Conclusions

For this dataset we can conclude that:

- The effect "Intervention" vs. "Non-intervention" is clearly present and biologically relevant.
- In particular, there are about four times more eggs in the "Non-intervention" sites.
- The effect of time is non-linear and was successfully approximated here with a quadratic function here.
- Altitude and number of days in the field do not appears to play a relevant role.
- There does not seem to be relevant differences among municipalities.
- There are relevant differences among traps.

7 Session Information

```
sessionInfo()
```

```
R version 4.0.4 (2021-02-15)
Platform: x86_64-w64-mingw32/x64 (64-bit)
Running under: Windows 10 x64 (build 19041)
```

```
Matrix products: default

locale:
[1] LC_COLLATE=English_Switzerland.1252  LC_CTYPE=English_Switzerland.1252
[3] LC_MONETARY=English_Switzerland.1252 LC_NUMERIC=C
[5] LC_TIME=English_Switzerland.1252

attached base packages:
[1] stats     graphics  grDevices utils     datasets  methods   base

other attached packages:
[1] gridExtra_2.3     lubridate_1.7.9.2 glmmTMB_1.0.2.1   ggplot2_3.3.3
[5] lattice_0.20-41   dplyr_1.0.4       checkpoint_0.4.10 knitr_1.31

loaded via a namespace (and not attached):
 [1] Rcpp_1.0.6        nloptr_1.2.2.2    pillar_1.5.0      compiler_4.0.4
 [5] TMB_1.7.19        tools_4.0.4       boot_1.3-26       statmod_1.4.35
 [9] digest_0.6.27     lme4_1.1-26       nlme_3.1-152      evaluate_0.14
[13] lifecycle_1.0.0   tibble_3.0.6      gtable_0.3.0      mgcv_1.8-33
[17] pkgconfig_2.0.3   rlang_0.4.10      Matrix_1.3-2      rstudioapi_0.13
[21] cli_2.3.1         yaml_2.2.1        xfun_0.22         withr_2.4.1
[25] stringr_1.4.0     generics_0.1.0    vctrs_0.3.6       grid_4.0.4
[29] tidyselect_1.1.0  glue_1.4.2        R6_2.5.0          fansi_0.4.2
[33] rmarkdown_2.7     minqa_1.2.4       farver_2.0.3      purrr_0.3.4
[37] magrittr_2.0.1    MASS_7.3-53       scales_1.1.1      ellipsis_0.3.1
[41] htmltools_0.5.1.1 splines_4.0.4     assertthat_0.2.1  colorspace_2.0-0
[45] labeling_0.4.2    utf8_1.1.4        stringi_1.5.3     munsell_0.5.0
[49] crayon_1.4.1
```
