# Supplementary material for: Effectiveness of integrated Aedes albopictus management in southern Switzerland
Source: Parasit Vectors. 2021 Aug 16;14:405. doi: 10.1186/s13071-021-04903-2 (PMC8365973; doi:10.1186/s13071-021-04903-2)
Supplement: Supplementary file 12 — Additional file 12: Text S5. This pdf file documents the full analysis of the number of Aedes albopictus adult females collected in 2019. This pdf was created with Rmarkdown and allows for the full reproducibility of the analysis. [file 13071_2021_4903_MOESM12_ESM.pdf]

Additional file 12: Data Analysis Nr. adults 2019

Dr. Matteo Tanadini | Zurich Data Scientists (ZDS)

March 24, 2021

Contents

1 Reproducibility **2**

2 Loading packages **2**

3 Getting data **2**

4 Graphical Analysis **4**

4.1 AREA . . . 4
4.2 MUNICIPALITY . . . 5
4.3 TRAP.ID.fac . . . 5
4.4 Date.when.GAT.collected . . . 6
4.5 No..Days.GAT.in.field . . . 8
4.6 ALTITUDE . . . 9
4.7 Spatial structure . . . 10

5 Modelling **10**

5.1 Generalised Mixed-Effects Models . . . 10
5.2 Visualising the model fit . . . 12
5.3 Quantifying effects . . . 16
5.4 Model checking . . . 17
5.4.1 Checking the model equation . . . 18
5.4.2 Checking the structure of the random effects . . . 18
5.4.3 Checking normality of the random effects . . . 19
5.4.4 Checking the mean-variance relationship assumption . . . 20
5.5 Checking whether zero-inflation is needed . . . 21
5.5.1 Graphically evaluating the goodness-of-fit . . . 23

6 Conclusions **24**

7 Session Information **24**

1 Reproducibility

In order to make the analysis fully reproducible, we "freeze" package versions using the *{checkpoint}* package. In particular, we use all packages versions available on CRAN on first of March 2021.

```
## (in this chunk messages are omitted)
##
library(checkpoint)
checkpoint("2021-01-01",
           checkpointLocation = "../")
```

2 Loading packages

We load all add-on packages used in this analysis.

```
## (messages are omitted from this chunk)
##
library(dplyr)
library(lattice)
library(ggplot2)
library(glmmTMB)
library(lubridate)
library(gridExtra)
library(tidyr)
```

3 Getting data

Note that to ensure reproducibility, the platform-agnostic file type "RDS" is used. Nevertheless, the corresponding "csv" file is also provided.

```
d.adults.2019 <- readRDS("../0_Data_Preparation_For_CH_vs_I/Created_Datasets/InterventionPaper_adults.R
##
str(d.adults.2019)
```

```
tibble [301 x 13] (S3: tbl_df/tbl/data.frame)
 $ WGS84.LAT              : num [1:301] 45.8 45.8 45.8 45.8 45.8 ...
 $ WGS84.LNG              : num [1:301] 9 9 9 9 9 ...
 $ ALTITUDE               : num [1:301] 263 263 263 263 263 263 263 263 263 316 ...
 $ AREA                   : Factor w/ 2 levels "Intervention",..: 1 1 1 1 1 1 1 1 1 1 ...
 $ MUNICIPALITY           : Factor w/ 6 levels "Balerna","Coldrerio",..: 1 1 1 1 1 1 1 1 1 1 ...
 $ Date.when.GAT.installed: POSIXct[1:301], format: "2019-06-05" "2019-06-18" ...
 $ Date.when.GAT.collected: POSIXct[1:301], format: "2019-06-18" "2019-07-03" ...
 $ No..Days.GAT.in.field  : num [1:301] 13 15 13 14 14 13 16 16 13 13 ...
 $ Week.when.GAT.collected: num [1:301] 25 27 29 31 33 35 37 39 41 25 ...
 $ No..Ad..Albo.in.GAT    : num [1:301] 2 5 7 9 7 8 4 8 4 0 ...
 $ No..Ad..Albo.in.14.days: num [1:301] 2.15 4.67 7.54 9 7 ...
 $ TRAP.ID.fac            : Factor w/ 36 levels "BAL-11a","BAL-2b",..: 1 1 1 1 1 1 1 1 1 2 ...
 $ Day.GAT.collected      : num [1:301] 169 184 197 211 225 238 254 270 283 169 ...
```

```r
print(d.adults.2019, n = 5, width = Inf)
```

```
# A tibble: 301 x 13
  WGS84.LAT WGS84.LNG ALTITUDE AREA         MUNICIPALITY Date.when.GAT.installed
      <dbl>     <dbl>    <dbl> <fct>        <fct>        <dttm>
1      45.8      9.00      263 Intervention Balerna      2019-06-05 00:00:00
2      45.8      9.00      263 Intervention Balerna      2019-06-18 00:00:00
3      45.8      9.00      263 Intervention Balerna      2019-07-03 00:00:00
4      45.8      9.00      263 Intervention Balerna      2019-07-16 00:00:00
5      45.8      9.00      263 Intervention Balerna      2019-07-30 00:00:00
  Date.when.GAT.collected No..Days.GAT.in.field Week.when.GAT.collected
  <dttm>                                  <dbl>                   <dbl>
1 2019-06-18 00:00:00                        13                      25
2 2019-07-03 00:00:00                        15                      27
3 2019-07-16 00:00:00                        13                      29
4 2019-07-30 00:00:00                        14                      31
5 2019-08-13 00:00:00                        14                      33
  No..Ad..Albo.in.GAT No..Ad..Albo.in.14.days TRAP.ID.fac Day.GAT.collected
                <dbl>                   <dbl> <fct>                   <dbl>
1                   2                    2.15 BAL-11a                   169
2                   5                    4.67 BAL-11a                   184
3                   7                    7.54 BAL-11a                   197
4                   9                    9    BAL-11a                   211
5                   7                    7    BAL-11a                   225
# ... with 296 more rows
```

4 Graphical Analysis

We graphically analyse the marginal effect that the available predictors have on the response variable (i.e. "number of eggs").

4.1 AREA

The predictor *AREA* defines whether the trap is to be found in a "treated" site (i.e. "Intervention") or not (i.e. "Non-intervention").

Note that since very many graphs have a similar structure, we first create a "proto graph" that is then adapted for each predictor.

```
gg.proto <- ggplot(data = d.adults.2019,
                   mapping = aes(y = No..Ad..Albo.in.GAT)) +
  scale_y_sqrt() +
  geom_hline(yintercept = 0)
```

Let's now recreate the graph for the effect of *AREA* with the response variable appropriately transformed.

```
gg.proto +
  geom_boxplot(mapping = aes(x = AREA))
```

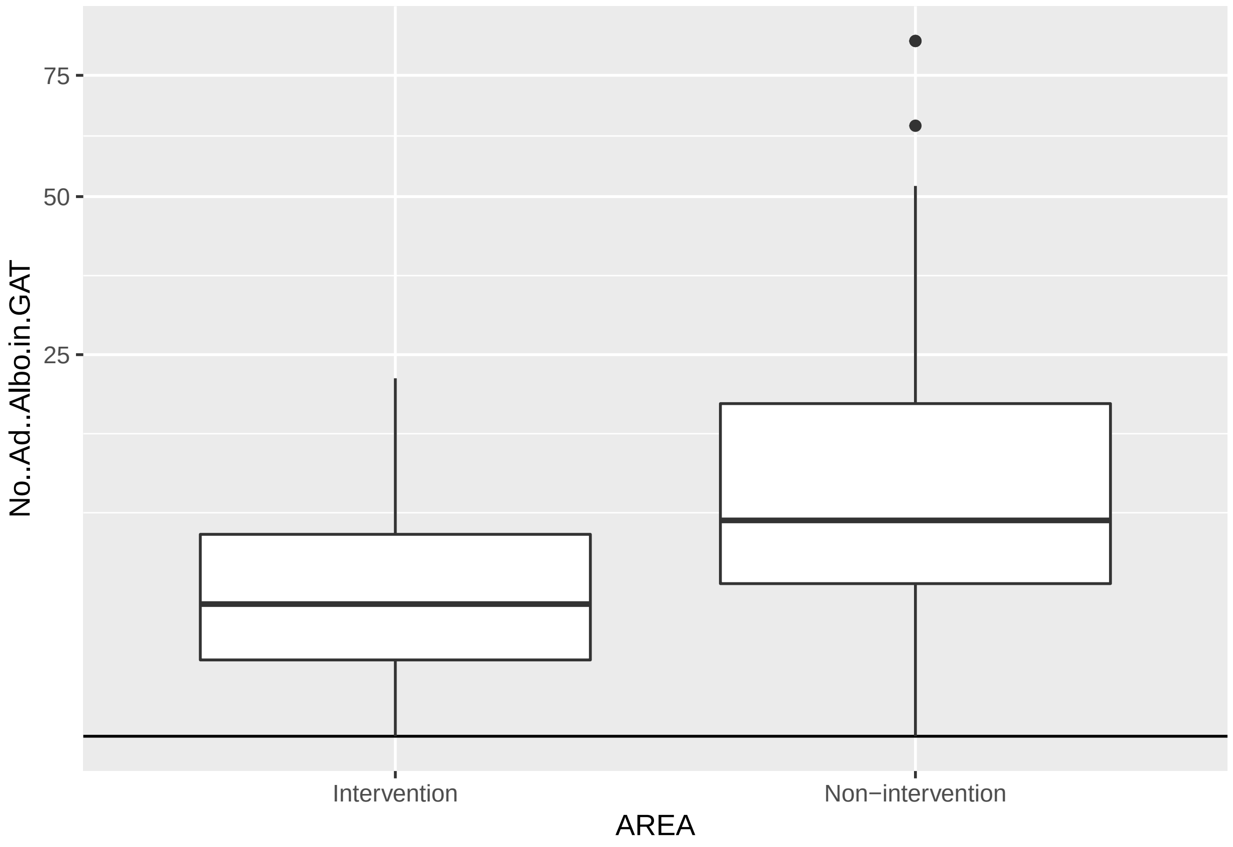

There seems to be quite a difference between the "Intervention" and the "non-intervention" groups. Note, also that the square-root transformation successfully stabilised the variance.

4.2 MUNICIPALITY

```
gg.proto +
  geom_boxplot(mapping = aes(x = MUNICIPALITY,
                             colour = AREA))
```

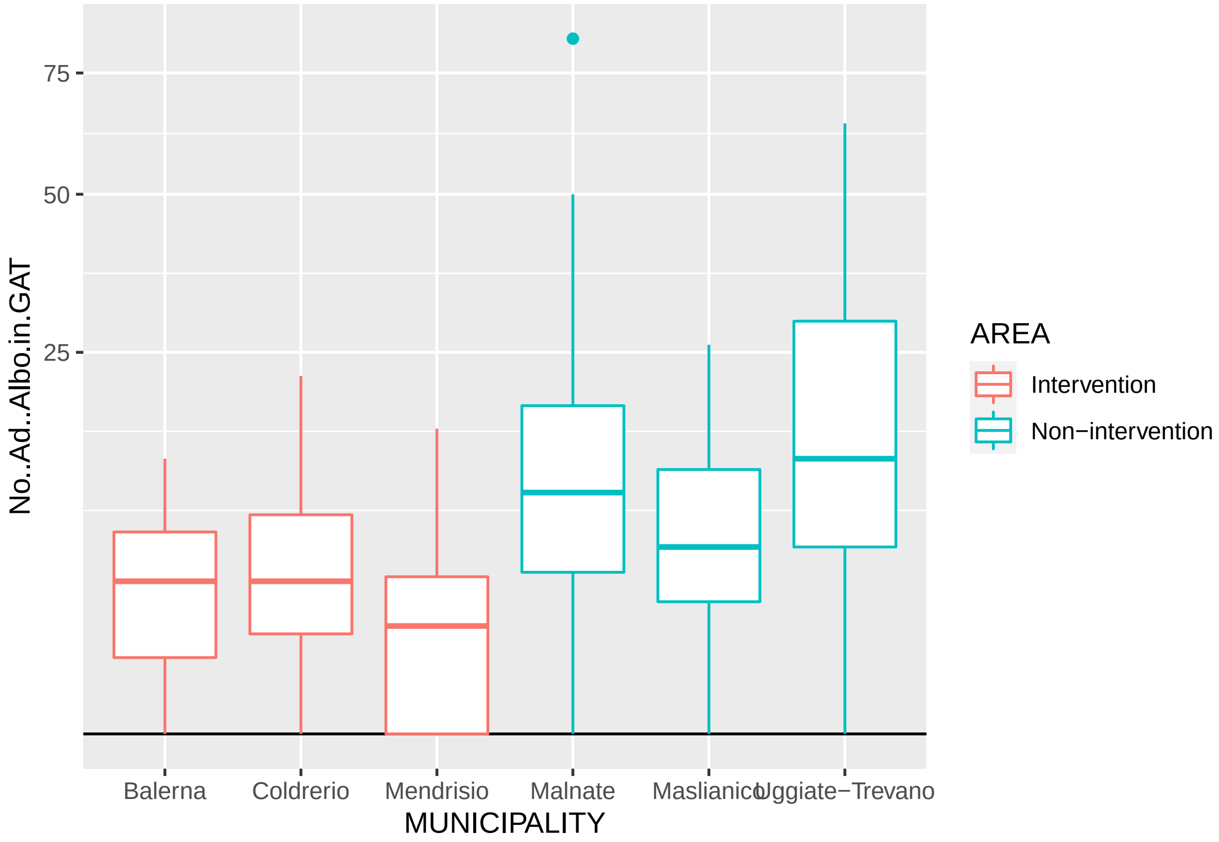

In terms of median values, there is little variation among municipalities that belong to the same *AREA* group. Compared to the "nr. of eggs" data, we do see more variation among municipalities that is not due to *AREA* only.

4.3 TRAP.ID.fac

```
gg.proto +
  geom_boxplot(mapping = aes(x = TRAP.ID.fac,
                             colour = MUNICIPALITY)) +
  theme(axis.text.x = element_text(angle = 90))
```

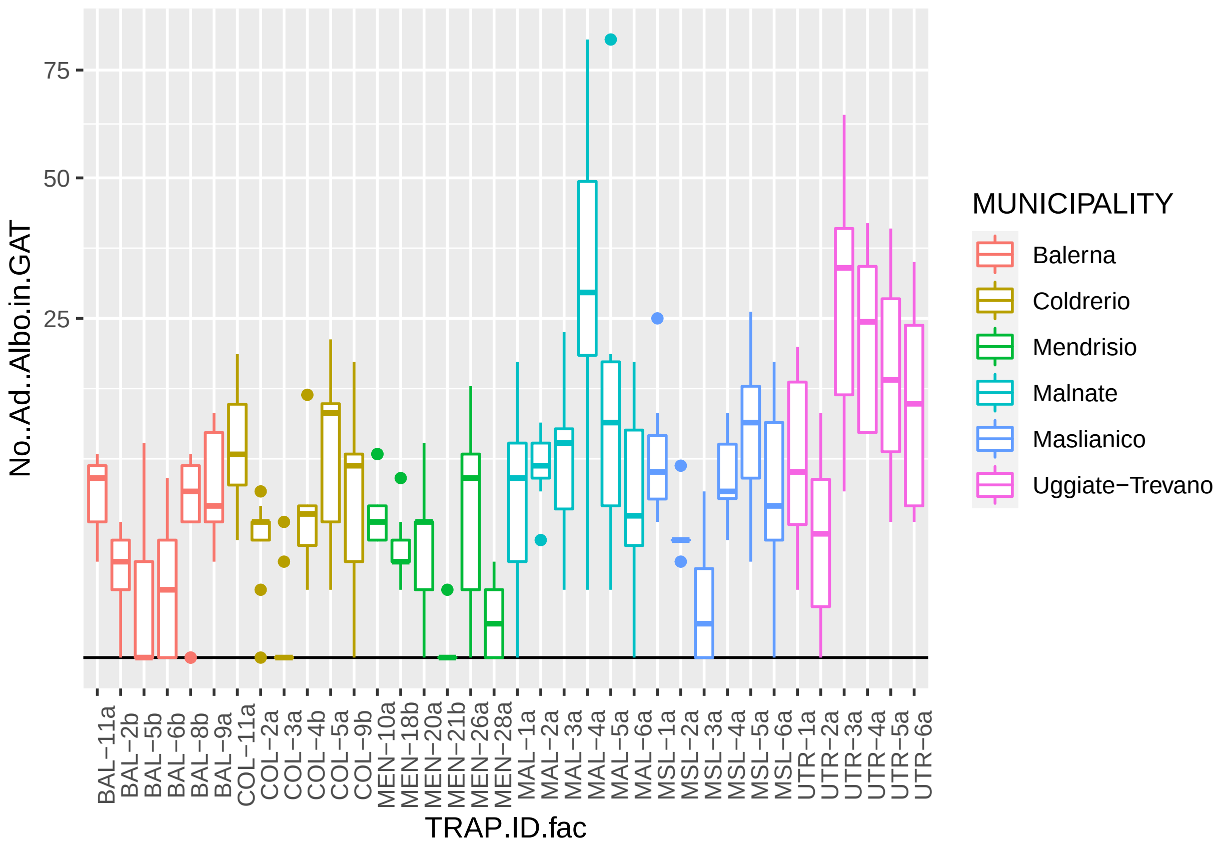

In terms of medians there is some non-negligible variation within municipalities (i.e. among traps of the same site).

4.4 Date.when.GAT.collected

Let's include the time component in these graphs. Here we visualise the counts for each trap over time (as a solid line). Note that panelling is used to differentiate among *AREA* levels. Indeed, as *AREA* is the predictor of main interest in this analysis, we may want to include its interactions (when needed) in the model. A smoother is added in both panels to highlight the "shared" time trend within both groups.

```
## (messages are omitted from this chunk)
##
ggplot(data = d.adults.2019,
       mapping = aes(y = No..Ad..Albo.in.GAT,
                     x = Date.when.GAT.collected,
                     group = TRAP.ID.fac)) +
  scale_y_sqrt(breaks = c(0,50,100,200,500,1000,2000),
               minor_breaks = FALSE) +
  geom_hline(yintercept = 0) +
  geom_line(show.legend = FALSE,
            alpha = 0.4) +
  facet_wrap(~AREA) +
  geom_smooth(mapping = aes(group = 1), colour = "#3366FF")
```

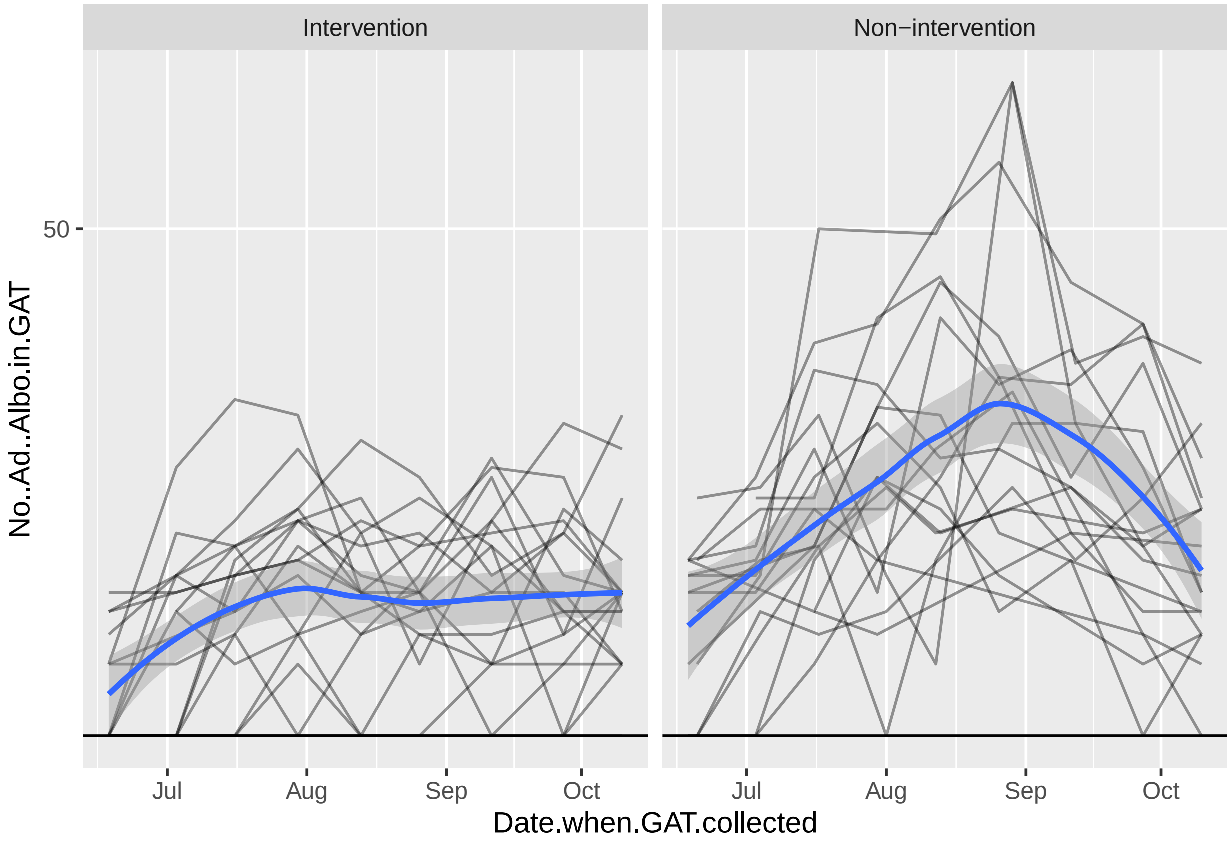

There is a clear non-linear seasonal pattern in both groups. This pattern is then modelled as a quadratic effect. In this case, an interaction may be needed to allow different quadratic shapes.

For the main publication part we also reproduce the same graph in the original scale.

```
## (messages are omitted from this chunk)
##
ggplot(data = d.adults.2019,
       mapping = aes(y = No..Ad..Albo.in.GAT,
                     x = Date.when.GAT.collected,
                     group = TRAP.ID.fac)) +
  # scale_y_sqrt() + ## Not sqrt-transformed
  geom_hline(yintercept = 0) +
  geom_line(show.legend = FALSE,
            alpha = 0.4) +
  facet_wrap(~AREA) +
  geom_smooth(mapping = aes(group = 1), colour = "#3366FF")
```

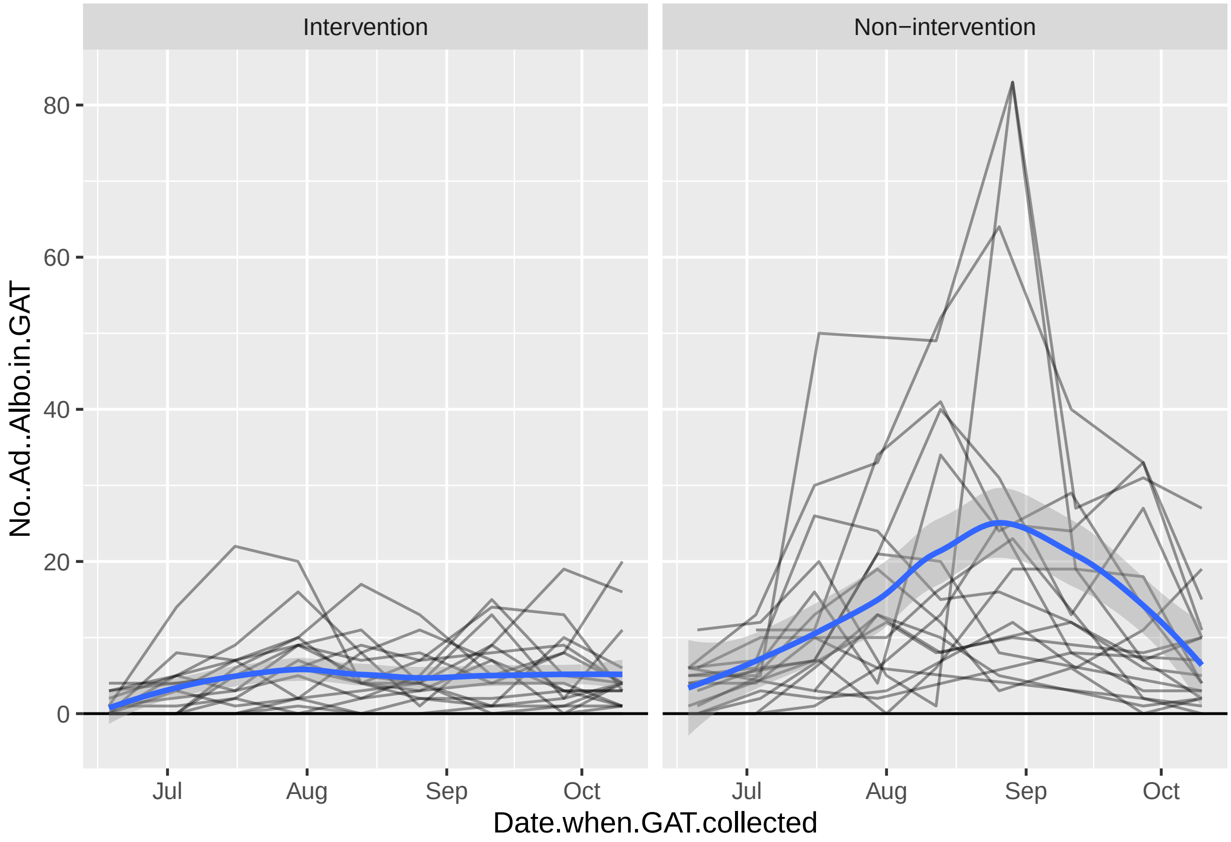

The actual counts highlights the dramatic difference between the the two treatments.

Note that x-axis here reports the date of the GAT collection. For the modelling phase, we are going to use the "Day of the year" as a predictor. Using one or the other is fully equivalent. However, Dates makes graphs more readable (as months are reported). While using a numeric variable for modelling is simpler than using a Date object.

4.5 No..Days.GAT.in.field

Each trap was not left the exact same number of days in the field. Therefore, we may want to account for the "exposure" effect. In theory, we would expect that traps that are left longer in the field contain more eggs.

```
## (warnings and messages are omitted from this chunk)
##
gg.proto +
  aes(x = No..Days.GAT.in.field) +
  geom_point() +
  geom_smooth() +
  facet_wrap(~ AREA)
```

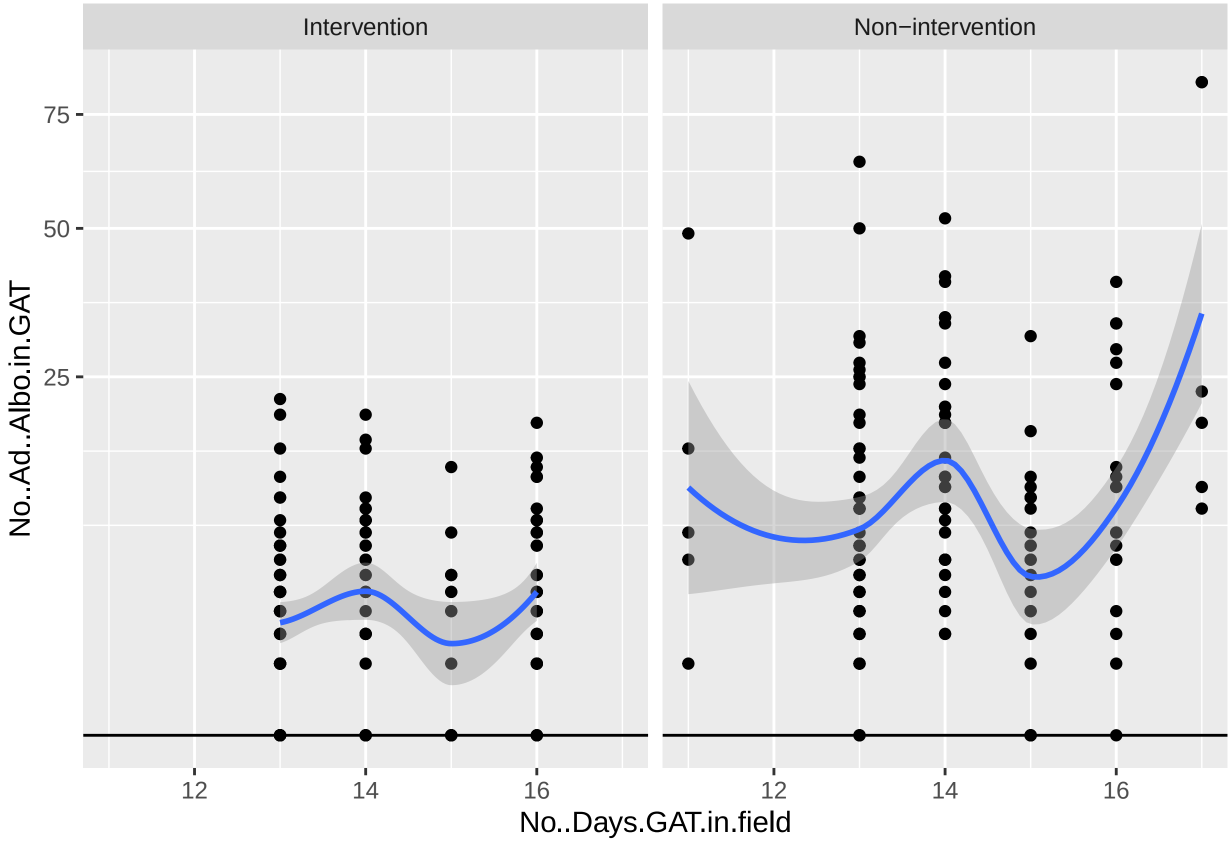

There is no clear effect of this predictor on the response variable.

4.6 ALTITUDE

Let's visualise the marginal effect of altitude while panelling for *AREA*.

```
## (messages are omitted from this chunk)
##
gg.proto +
  aes(x = ALTITUDE) +
  geom_point() +
  geom_smooth() +
  facet_wrap(. ~ AREA)
```

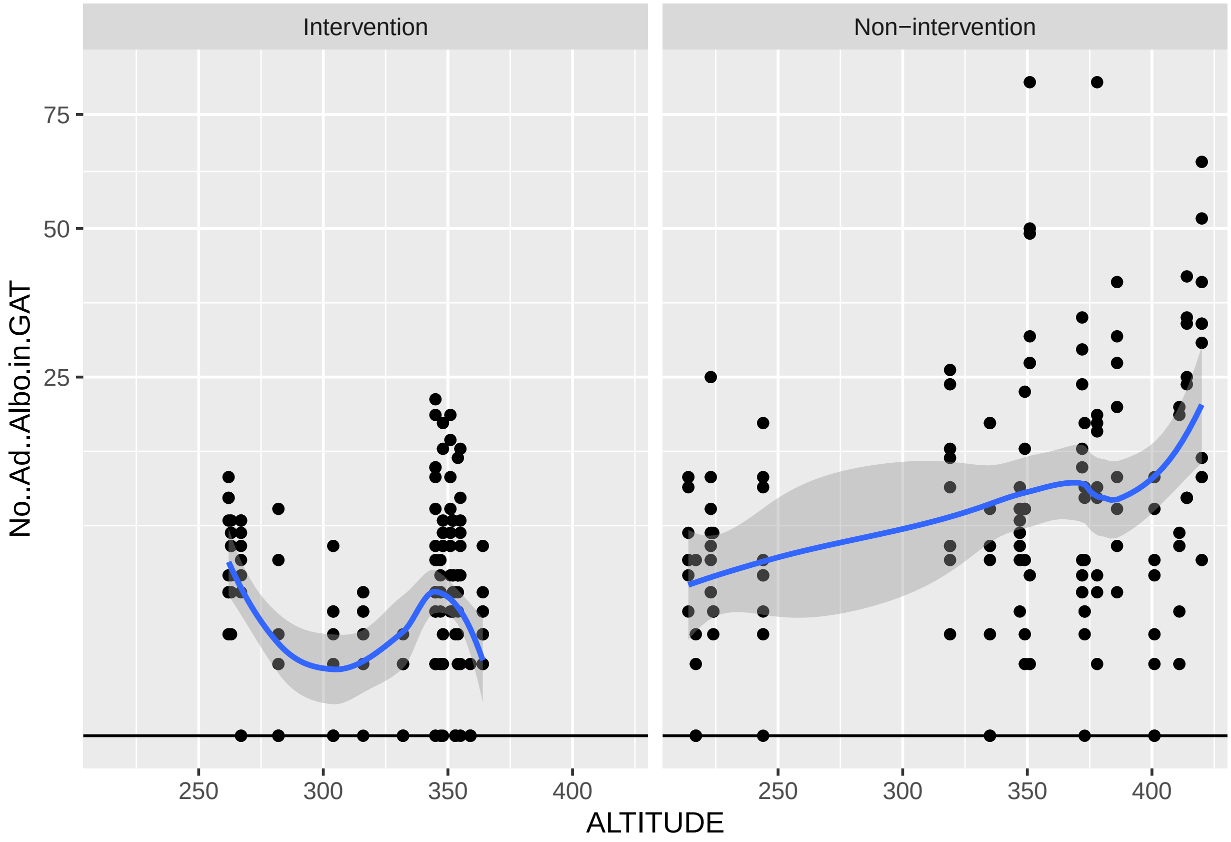

Altitude does not seem to have a clear effect either.

4.7 Spatial structure

See Appendix 1 (i.e. nr. of eggs).

5 Modelling

5.1 Generalised Mixed-Effects Models

Let's fit the starting model. As the graphical analysis indicated that there may be an interaction between *Day* and *AREA* we fit a model that includes this two-fold interaction.

```
## (warning are omitted from this chunk)
##
mod.nb.1 <- glmmTMB(No..Ad..Albo.in.GAT ~ AREA *
                      poly(Day.GAT.collected, degree = 2) +
                      No..Days.GAT.in.field +
                      scale(ALTITUDE) +
                      (1 | TRAP.ID.fac) + (1 | MUNICIPALITY),
                    family = "nbinom2",
                    data = d.adults.2019)
```

```r
##
summary(mod.nb.1)
```

```
 Family: nbinom2  ( log )
Formula:
No..Ad..Albo.in.GAT ~ AREA * poly(Day.GAT.collected, degree = 2) +
    No..Days.GAT.in.field + scale(ALTITUDE) + (1 | TRAP.ID.fac) +
    (1 | MUNICIPALITY)
Data: d.adults.2019

     AIC      BIC   logLik deviance df.resid
    1720     1761     -849     1698      290

Random effects:

Conditional model:
 Groups       Name        Variance Std.Dev.
 TRAP.ID.fac  (Intercept) 5.17e-01 0.719103
 MUNICIPALITY (Intercept) 1.25e-08 0.000112
Number of obs: 301, groups:  TRAP.ID.fac, 36; MUNICIPALITY, 6

Overdispersion parameter for nbinom2 family (): 3.13

Conditional model:
                                                          Estimate Std. Error
(Intercept)                                                 0.9007     0.5105
AREANon-intervention                                        1.0500     0.2577
poly(Day.GAT.collected, degree = 2)1                        5.2765     1.2280
poly(Day.GAT.collected, degree = 2)2                       -4.5174     1.1984
No..Days.GAT.in.field                                       0.0194     0.0338
scale(ALTITUDE)                                             0.2050     0.1229
AREANon-intervention:poly(Day.GAT.collected, degree = 2)1  -1.5521     1.6075
AREANon-intervention:poly(Day.GAT.collected, degree = 2)2  -4.1381     1.5691
                                                          z value Pr(>|z|)
(Intercept)                                                  1.76  0.07764 .
AREANon-intervention                                         4.07  4.6e-05 ***
poly(Day.GAT.collected, degree = 2)1                         4.30  1.7e-05 ***
poly(Day.GAT.collected, degree = 2)2                        -3.77  0.00016 ***
No..Days.GAT.in.field                                        0.57  0.56586
scale(ALTITUDE)                                              1.67  0.09521 .
AREANon-intervention:poly(Day.GAT.collected, degree = 2)1   -0.97  0.33429
AREANon-intervention:poly(Day.GAT.collected, degree = 2)2   -2.64  0.00836 **
---
Signif. codes:  0 '***' 0.001 '**' 0.01 '*' 0.05 '.' 0.1 ' ' 1
```

The model fits in a few seconds and there are no convergence problems. Note that the warnings omitted here are harmless (see developers comments about these warnings here).

Note that the z-based p-values indicate that the interaction between *Day* and *AREA* is truly needed. Let's formally test that.

```r
## (this chunk is cached and warnings are omitted)
##
```

```
mod.nb.2 <- update(mod.nb.1,
                   . ~ . - AREA:poly(Day.GAT.collected, degree = 2))
anova(mod.nb.1, mod.nb.2,
      test = "Chisq")
```

```
Data: d.adults.2019
Models:
mod.nb.2: No..Ad..Albo.in.GAT ~ AREA + poly(Day.GAT.collected, degree = 2) + , zi=~0, disp=~1
mod.nb.2:     No..Days.GAT.in.field + scale(ALTITUDE) + (1 | TRAP.ID.fac) + , zi=~0, disp=~1
mod.nb.2:     (1 | MUNICIPALITY), zi=~0, disp=~1
mod.nb.1: No..Ad..Albo.in.GAT ~ AREA * poly(Day.GAT.collected, degree = 2) + , zi=~0, disp=~1
mod.nb.1:     No..Days.GAT.in.field + scale(ALTITUDE) + (1 | TRAP.ID.fac) + , zi=~0, disp=~1
mod.nb.1:     (1 | MUNICIPALITY), zi=~0, disp=~1
         Df  AIC  BIC logLik deviance Chisq Chi Df Pr(>Chisq)
mod.nb.2  9 1725 1758   -853     1707
mod.nb.1 11 1720 1761   -849     1698  8.63      2      0.013 *
---
Signif. codes:  0 '***' 0.001 '**' 0.01 '*' 0.05 '.' 0.1 ' ' 1
```

Indeed, the interaction term between *AREA* and time is needed.

The estimated variance of the *MUNICIPALITY* random effect seems to be very small again (see nr. of eggs analysis). Let's inspect this further.

```
options(scipen = 999) ## to drop scientific notation
VarCorr(mod.nb.1)
```

```
Conditional model:
 Groups       Name        Std.Dev.
 TRAP.ID.fac  (Intercept) 0.719103
 MUNICIPALITY (Intercept) 0.000112
```

```
options(scipen = 0) ## to set it back to default
```

The variability of *MUNICIPALITY* is extremely small compared to *TRAP.ID.fac* and it is even close to zero, which is the boundary of the parameter space. Given that *MUNICIPALITY* is a design variable and the model converges we don't drop it from the model.

5.2 Visualising the model fit

We start by visualising the fitted values of the model (in red) along with the observed values (in gray). Note that all these plots still use a square-root transformed y-axis.

```
## (warning are omitted from this chunk)
##
d.adults.2019$fit.mod.nb.1 <- fitted(mod.nb.1)
##
ggplot(data = d.adults.2019,
       mapping = aes(y = No..Ad..Albo.in.GAT,
                     x = Date.when.GAT.collected,
```

```
                      group = TRAP.ID.fac)) +
  scale_y_sqrt() +
  geom_line(alpha = 0.2) +
  facet_wrap(. ~ AREA) +
  geom_line(mapping = aes(y = fit.mod.nb.1),
            col = "red",
            alpha = 0.8)
```

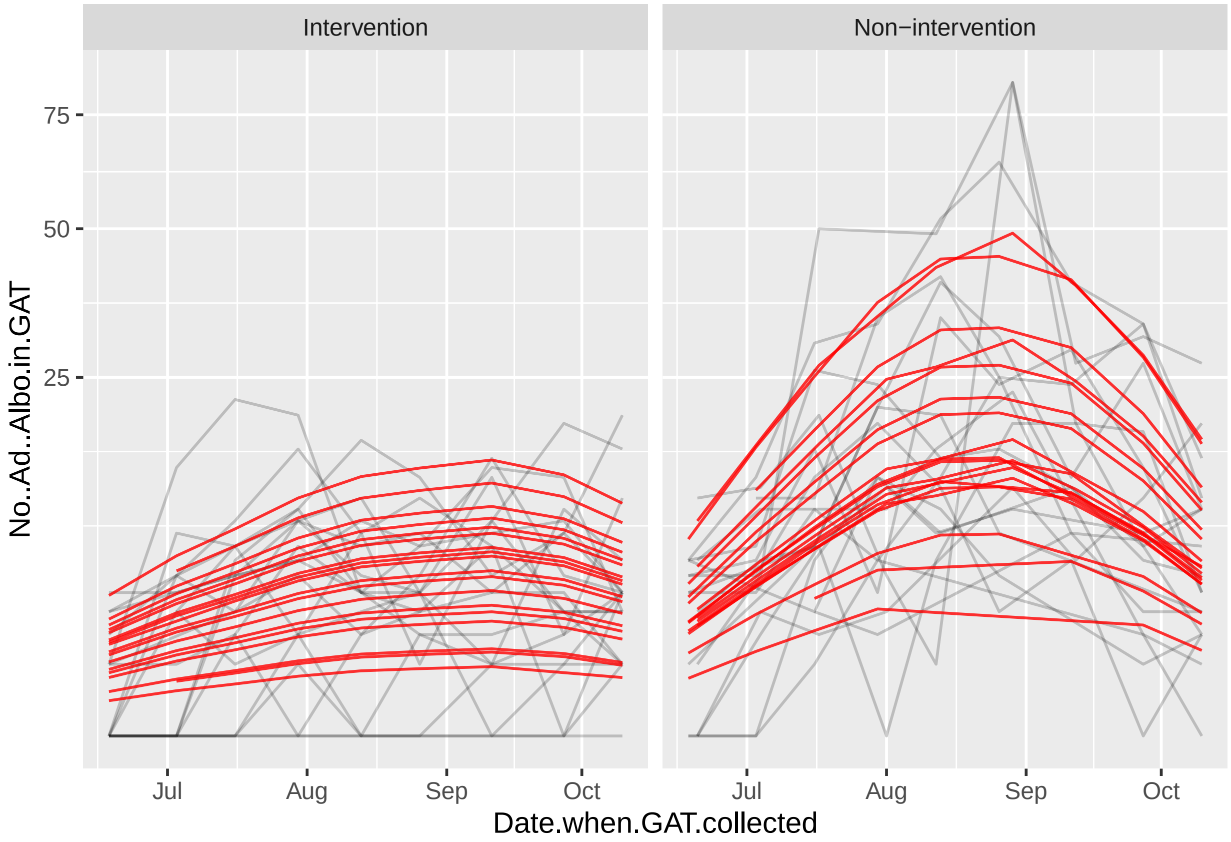

The model fit look sensible.

Note that the predicted values for each trap are not nice and quadratic because i) we are evaluating the model at 10 discrete dates only (i.e. the observed dates) and ii) because the model also contains the continuous predictor *No..Days.GAT.in.field*.

In the next graph we are going to show the predictions at population level (i.e. without the estimated random effects). In addition, we keep *No..Days.GAT.in.field* and *ALTITUDE* constant at their mean value and we make predictions on a finer grid.

```
## (results are omitted from this chunk)
## (warnings are omitted from this chunk)
##
## 1. Creating the prediction dataset with expand.grid()
d.predict <- expand.grid(
```

```
  AREA = levels(d.adults.2019$AREA),
  Date.when.GAT.collected = seq(
    from = min(d.adults.2019$Date.when.GAT.collected),
    to = max(d.adults.2019$Date.when.GAT.collected),
    by = "day"),
  ALTITUDE = mean(d.adults.2019$ALTITUDE),
  No..Days.GAT.in.field = mean(
    d.adults.2019$No..Days.GAT.in.field),
  ## NB: predict.glmmTMB() requires RE even for pop-level predictions.
  TRAP.ID.fac = "BAL-11a",
  MUNICIPALITY = "Balerna")
##
dim(d.predict)
str(d.predict)
##
## 2. Converting Date to Day
d.predict$Day.GAT.collected <- yday(d.predict$Date.when.GAT.collected)
##
dim(d.predict)
str(d.predict)
##
## 3. Making predictions at population level
d.predict$pred.pop.level.mod.nb.1 <- predict(mod.nb.1,
                                         type = "response",
                                         newdata = d.predict,
                                         re.form = NA)
##
## 4. Producing the graph
ggplot(data = d.adults.2019,
       mapping = aes(y = No..Ad..Albo.in.GAT,
                     x = Date.when.GAT.collected,
                     group = TRAP.ID.fac)) +
  scale_y_sqrt() +
  geom_line(alpha = 0.2) +
  facet_wrap(. ~ AREA) +
  ## adding pop-level predictions
  geom_line(data = d.predict,
            size = 1,
            mapping = aes(y = pred.pop.level.mod.nb.1),
            col = "red")
```

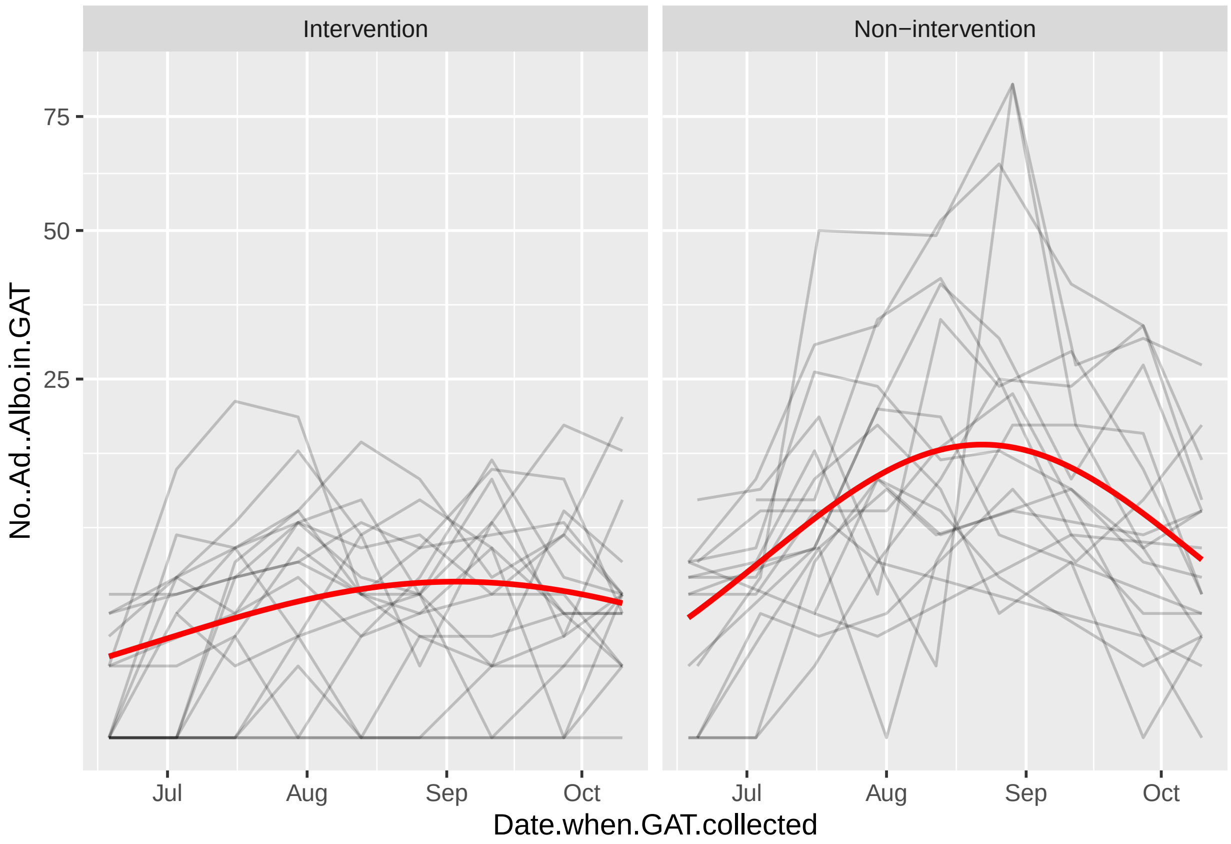

The fit looks sensible and the difference among the two groups is still quite clear.

Side remark: Note that the fit does not look perfectly quadratic because the fit is done in the natural logarithm scala (see link function).

To better appreciate the group differences, we visualise the fit in the original scala (i.e. without any transformation).

```
ggplot(data = d.adults.2019,
       mapping = aes(y = No..Ad..Albo.in.GAT,
                     x = Date.when.GAT.collected,
                     group = TRAP.ID.fac)) +
  # scale_y_sqrt() +  ## No sqrt-root transformation
  geom_line(alpha = 0.2) +
  facet_wrap(. ~ AREA) +
  geom_line(data = d.predict,
            size = 1,
            mapping = aes(y = pred.pop.level.mod.nb.1),
            col = "red")
```

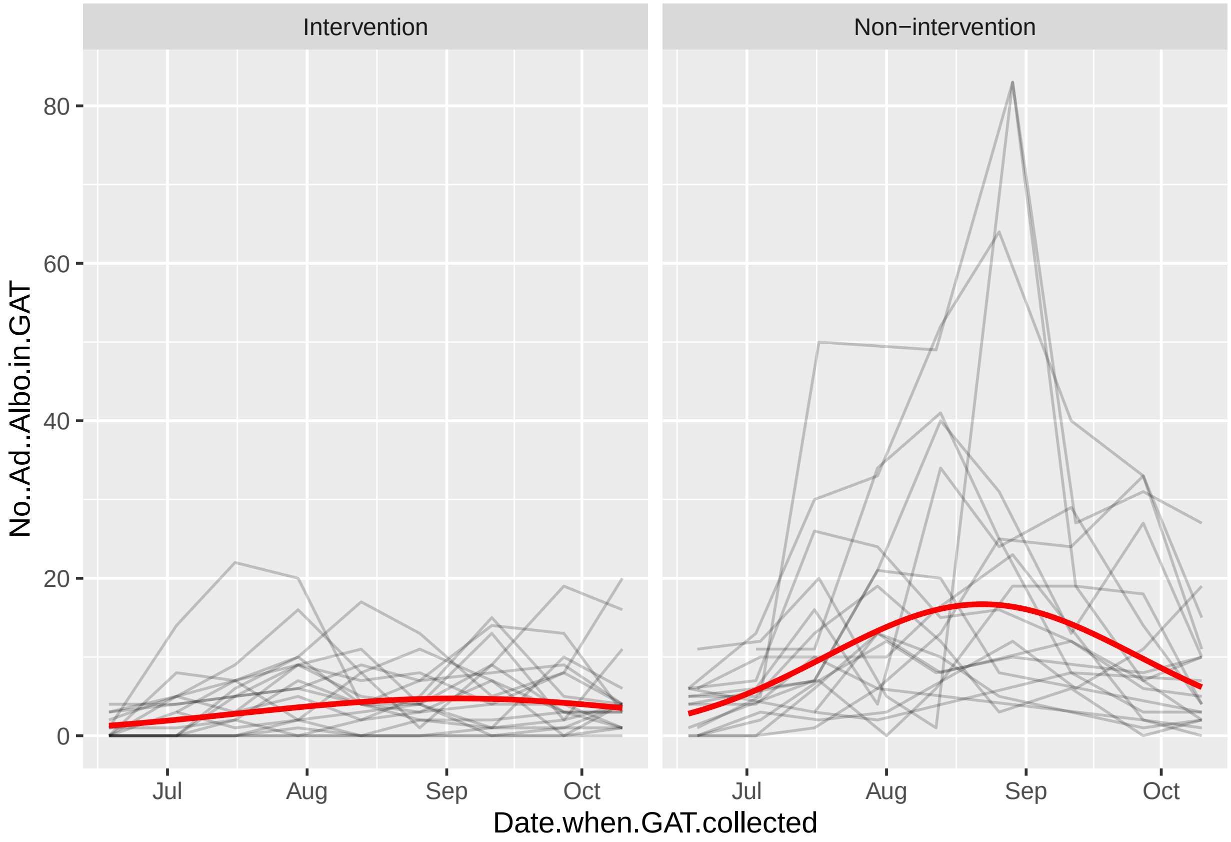

In the original scala, the difference between "intervention" and "Non-intervention" is dramatic.

5.3 Quantifying effects

In this model an interaction is present, therefore, we cannot simply look at the *Non-intervention* coefficient to evaluate the differences between "Non-intervention" and "Intervention". In particular, in this model the difference between the two groups changes over time.

Let's visualise how the ratio between the two groups changes over time. We first adapt the *d.predict* dataset and compute the ratio between predicted number of adults in "Non-intervention" and "Intervention".

```
## (results are omitted from this chunk)
##
d.predict.short <- d.predict %>%
  select(-TRAP.ID.fac, -MUNICIPALITY) %>%
  unique()
##
dim(d.predict.short)
head(d.predict.short)
##
##
d.predict.short.wide <- d.predict.short %>%
  spread(key = AREA,
         value = pred.pop.level.mod.nb.1)
##
```

```r
dim(d.predict.short.wide)
head(d.predict.short.wide)
##
##
d.predict.short.wide$ratio <- d.predict.short.wide$`Non-intervention` /
  d.predict.short.wide$Intervention
##
dim(d.predict.short.wide)
head(d.predict.short.wide)
```

We visualise the ratio evolution over time.

```r
ggplot(data = d.predict.short.wide,
       mapping = aes(y = ratio,
                     x = Date.when.GAT.collected)) +
  geom_line(col = "red") +
  ylim(c(0,4)) +
  geom_hline(yintercept = 1) ## H0: ratio is 1
```

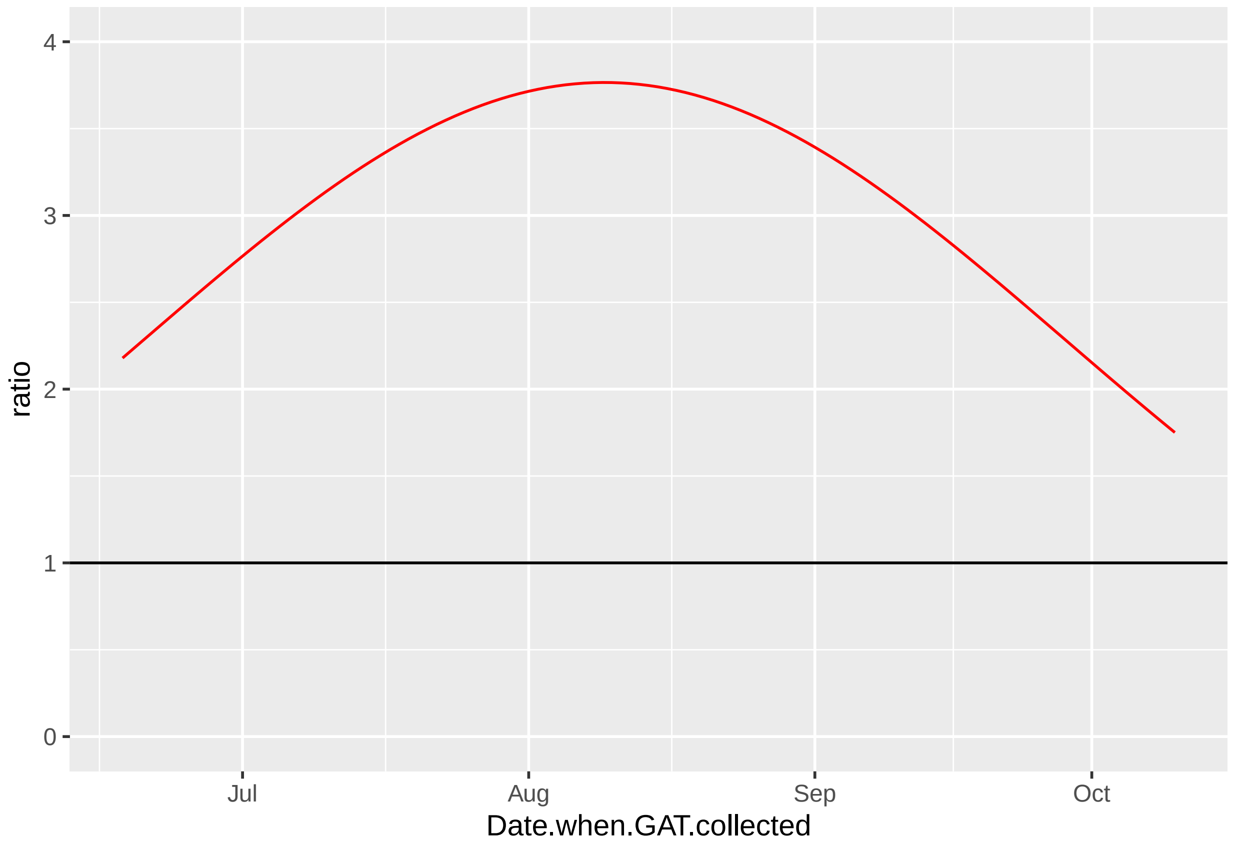

The ratio stays constantly above two and reaches a maximum of almost four in early August.

5.4 Model checking

5.4.1 Checking the model equation

Let's check the assumption that, in the linear predictor scale, the effect of *Day.GAT.collected* is quadratic. To test hypotheses we use the "Pearson" residuals.

```
## (messages and warnings are omitted from this chunk)
##
d.adults.2019$res.mod.nb.1 <- resid(mod.nb.1, type = "pearson")
##
ggplot(data = d.adults.2019,
       mapping = aes(y = res.mod.nb.1,
                     x = Date.when.GAT.collected)) +
  geom_hline(yintercept = 0) +
  geom_point() +
  facet_wrap(. ~ AREA) +
  geom_smooth()
```

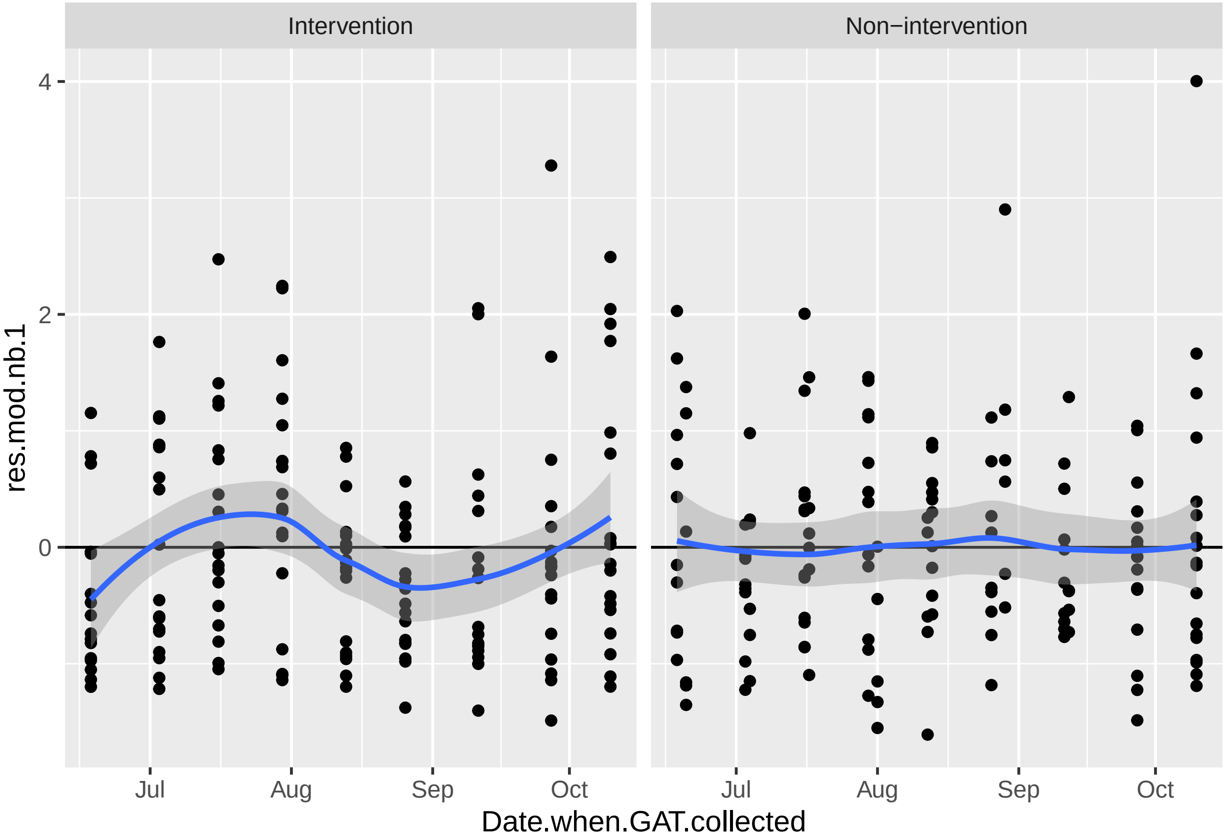

The model assumptions seem to be fulfilled. A slight deviation from linearity for the "Intervention" group is present. Note that this model does contain an interaction between time and *AREA* and therefore, will not be further complexified.

5.4.2 Checking the structure of the random effects

Let's now check at trap level whether assuming a simple random intercept is enough to model these short time series. In other words, we are graphically testing whether time correlation is present.

```
ggplot(data = d.adults.2019,
       mapping = aes(y = res.mod.nb.1,
                     x = Date.when.GAT.collected)) +
  geom_hline(yintercept = 0) +
  geom_line() +
  geom_point() +
  facet_wrap(. ~ TRAP.ID.fac, scales = "free_y") +
  theme(strip.text.x = element_blank(),
        axis.text.y = element_blank(),
        axis.ticks.y = element_blank(),
        axis.text.x = element_text(angle = 45))
```

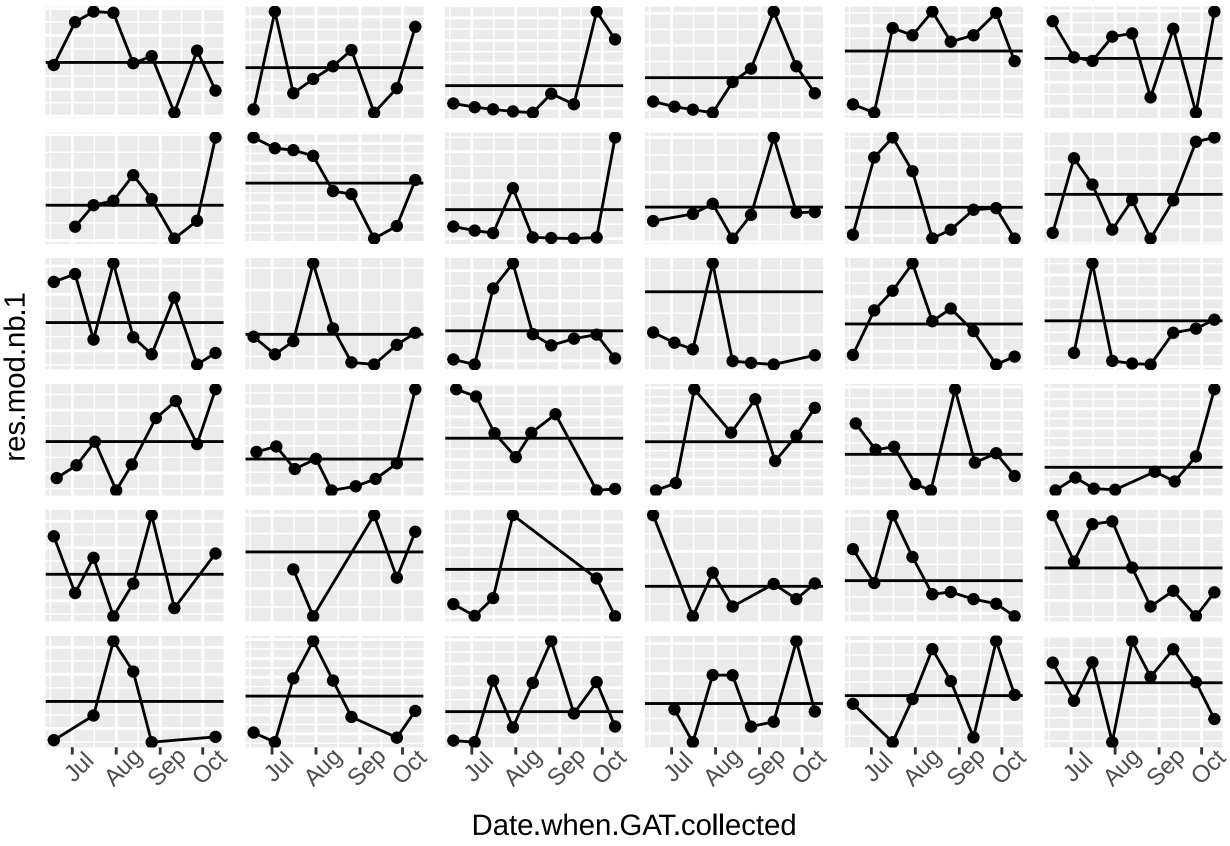

There is no evidence of temporal structure left at trap level.

5.4.3 Checking normality of the random effects

The negative binomial model we fitted assumes a normal distribution of the random effects (in the linear predictor space). The negative binomial model we fitted assumes a normal distribution of the random effects (in the linear predictor space).

```
par(mfrow = c(1,2))
##
qqnorm(unlist(ranef(mod.nb.1)$cond$TRAP.ID.fac))
```

```
qqline(unlist(ranef(mod.nb.1)$cond$TRAP.ID.fac))
##
qqnorm(unlist(ranef(mod.nb.1)$cond$MUNICIPALITY))
qqline(unlist(ranef(mod.nb.1)$cond$MUNICIPALITY))
```

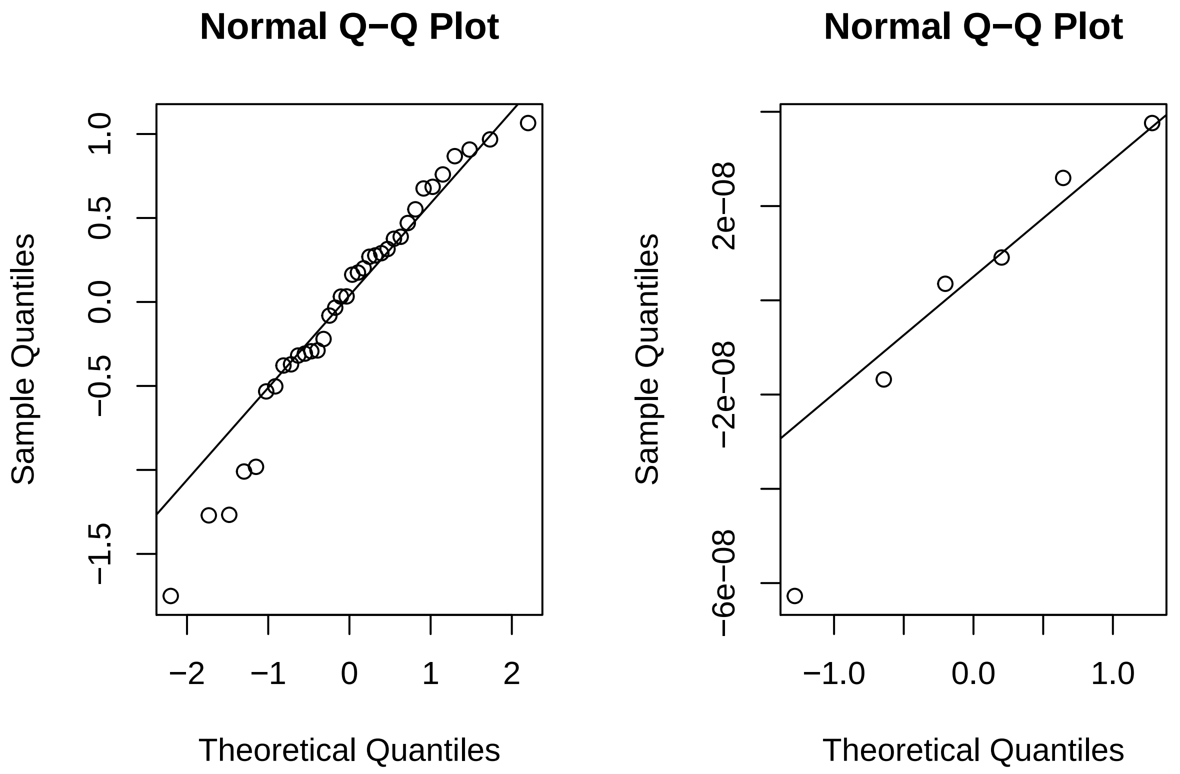

The normality assumption about the random effects does not appear to be violated for traps. Little can be said for *MUNICIPALITY* that has only six levels. Nevertheless, it is irrelevant as it estimated variance is close to zero.

5.4.4 Checking the mean-variance relationship assumption

The model fitted here assumes that the variance of the observations increases quadratically with the mean value (i.e. family is “nbinom2”). Let’s formally check this.

```
## (warning are omitted from this chunk)
##
mod.nb.1.linear <- update(mod.nb.1, family = "nbinom1")
##
AIC(mod.nb.1, mod.nb.1.linear)
```

```
                df  AIC
mod.nb.1        11 1720
mod.nb.1.linear 11 1722
```

```r
BIC(mod.nb.1, mod.nb.1.linear)
```

```
                df  BIC
mod.nb.1        11 1761
mod.nb.1.linear 11 1763
```

Both information criteria agree that the current family choice is very slightly superior to the "linear" parametrisation.

5.5 Checking whether zero-inflation is needed

We first check graphically whether the zero-inflation is present. Note that we are simulating from the fitted model.

```r
set.seed(2)
##
xy.1 <- densityplot(~sqrt(simulate(mod.nb.1)),
                    xlim = c(-2, 15),
                    ylim = c(0, 0.30),
                    main = "Simulated from model")
##
xy.2 <- densityplot(~sqrt(d.adults.2019$No..Ad..Albo.in.GAT),
                    xlim = c(-2, 15),
                    ylim = c(0, 0.30),
                    main = "Observed data")
##
plot(xy.1, split = c(1,1, 2,1))
plot(xy.2, split = c(2,1, 2,1), newpage = FALSE)
```

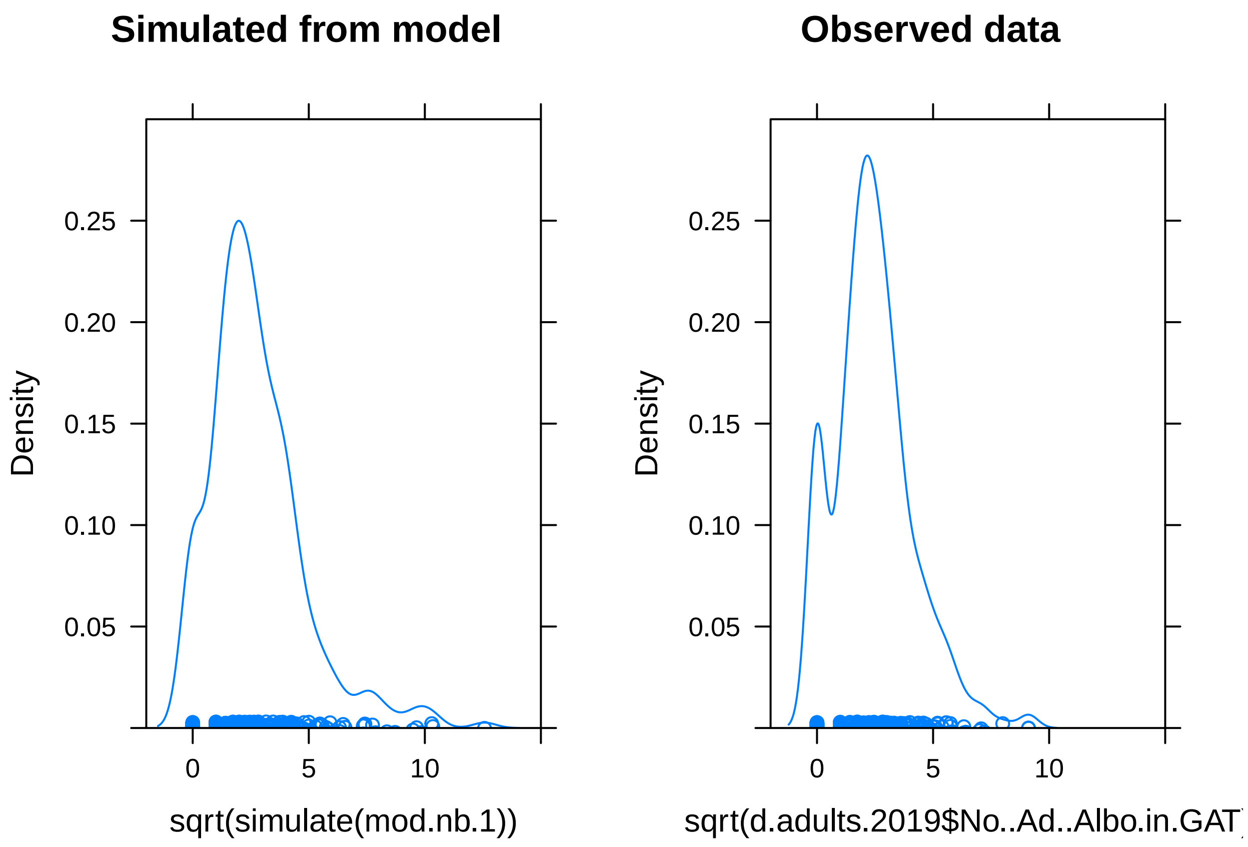

There is a slight over-abundance of zeros.

Let's now fit the zero-inflated models and compare the information criteria.

```
## (warning are omitted from this chunk)
##
trunc.mod.nb.1.linear <- update(mod.nb.1,
                                family = "truncated_nbinom1",
                                ziformula = ~ .)
trunc.mod.nb.1.quadratic <- update(mod.nb.1,
                                family = "truncated_nbinom2",
                                ziformula = ~ .)
##
AIC(mod.nb.1, trunc.mod.nb.1.linear, trunc.mod.nb.1.quadratic)
```

```
                         df  AIC
mod.nb.1                 11 1720
trunc.mod.nb.1.linear    21   NA
trunc.mod.nb.1.quadratic 21 1716
```

```
BIC(mod.nb.1, trunc.mod.nb.1.linear, trunc.mod.nb.1.quadratic)
```

```
                         df  BIC
mod.nb.1                 11 1761
trunc.mod.nb.1.linear    21   NA
trunc.mod.nb.1.quadratic 21 1794
```

The ZI model with linear parametrisation does not converge. The ZI model with the quadratic parametrisation does converge. AIC would favour the ZI-quadratic model over the current model. BIC would not. As there is no agreement, we retain *mod.nb.1* as the final model.

Note also the interpretation of ZI models, although very powerful, is way more complicated and inappropriate for this analysis (communication to policy-makers).

5.5.1 Graphically evaluating the goodness-of-fit

Observed values and fitted values are correlated to quantify the goodness of fit. This is the graphical equivalent of an R^2. Note that fitted values do include the estimated random effects here. Due to the skewness of the data, the graph is produced in the quare-root-transformed space as well.

```
gg.obsPred.NB <- ggplot(data = d.adults.2019,
       mapping = aes(y = No..Ad..Albo.in.GAT,
                     x = fit.mod.nb.1)) +
  geom_point() +
  geom_abline(intercept = 0, slope = 1) +
  ggtitle("NB: Obs vs. predicted") +
  coord_fixed()
##
gg.obsPred.NB.Sqrt <- gg.obsPred.NB +
  scale_y_sqrt() + scale_x_sqrt() +
  ggtitle("NB: Obs vs. predicted -- sqrt()") +
  coord_fixed()
```

```
Coordinate system already present. Adding new coordinate system, which will replace the existing one.
```

```
##
grid.arrange(gg.obsPred.NB, gg.obsPred.NB.Sqrt, ncol = 2)
```

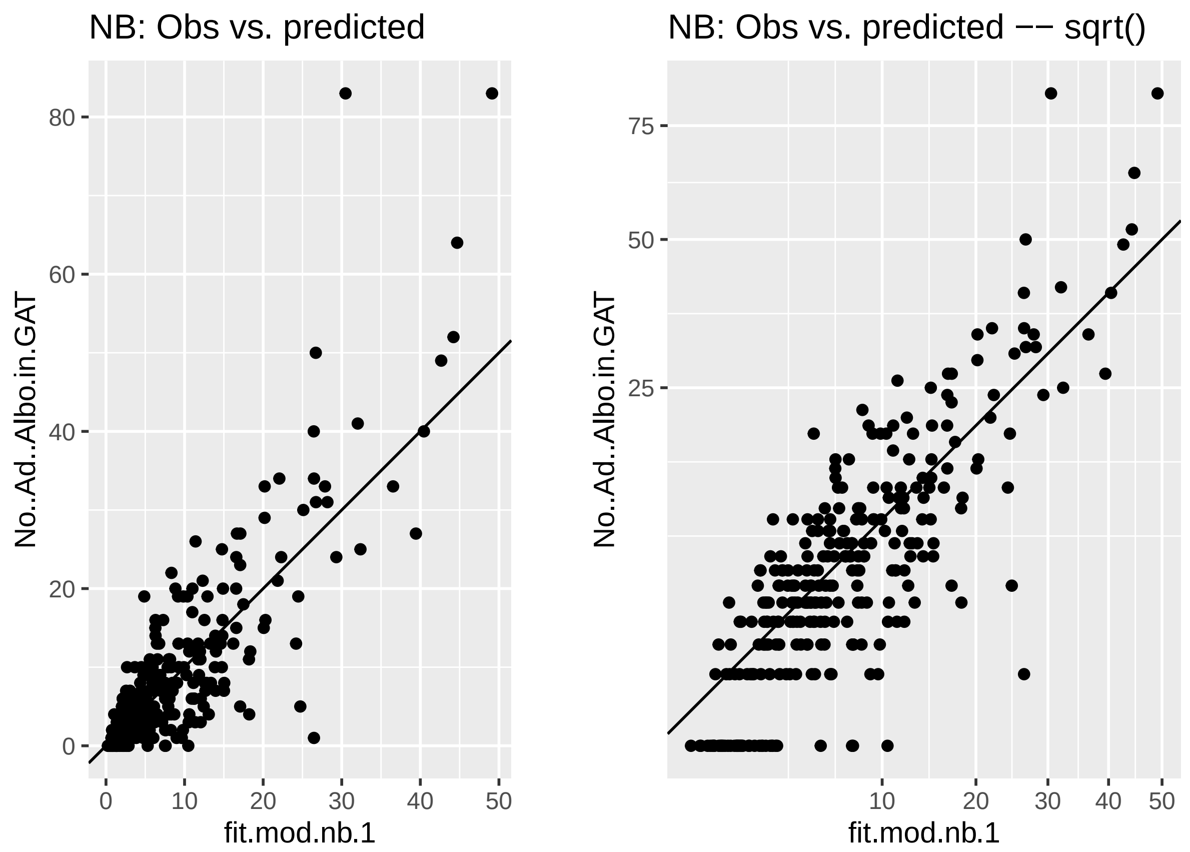

The model fit looks quite reasonable.

6 Conclusions

For this dataset we can conclude that:

- The effect "Intervention" vs. "Non-intervention" is clearly present and biologically relevant.
- In particular, this effect changes over time from a minimum of about 2 to a maximum in early August of about 4.
- The effect of time is non-linear (approximated here with a quadratic function).
- There does not seem to be relevant differences among municipalities.
- There are relevant differences among traps.
- *ALTITUDE* and the number of days in the field appear not to play a relevant role.

7 Session Information

```
sessionInfo()
```

```
R version 4.0.4 (2021-02-15)
Platform: x86_64-w64-mingw32/x64 (64-bit)
Running under: Windows 10 x64 (build 19041)
```

```
Matrix products: default

locale:
[1] LC_COLLATE=English_Switzerland.1252  LC_CTYPE=English_Switzerland.1252
[3] LC_MONETARY=English_Switzerland.1252 LC_NUMERIC=C
[5] LC_TIME=English_Switzerland.1252

attached base packages:
[1] stats     graphics  grDevices utils     datasets  methods   base

other attached packages:
[1] tidyr_1.1.2       gridExtra_2.3     lubridate_1.7.9.2 glmmTMB_1.0.2.1
[5] ggplot2_3.3.3     lattice_0.20-41   dplyr_1.0.2       checkpoint_0.4.10
[9] knitr_1.31

loaded via a namespace (and not attached):
 [1] Rcpp_1.0.5        nloptr_1.2.2.2    pillar_1.4.7      compiler_4.0.4
 [5] TMB_1.7.19        tools_4.0.4       boot_1.3-26       digest_0.6.27
 [9] lme4_1.1-26       statmod_1.4.35    nlme_3.1-152      evaluate_0.14
[13] lifecycle_0.2.0   tibble_3.0.4      gtable_0.3.0      mgcv_1.8-33
[17] pkgconfig_2.0.3   rlang_0.4.10      Matrix_1.3-2      cli_2.2.0
[21] yaml_2.2.1        xfun_0.22         withr_2.3.0       stringr_1.4.0
[25] generics_0.1.0    vctrs_0.3.6       grid_4.0.4        tidyselect_1.1.0
[29] glue_1.4.2        R6_2.5.0          fansi_0.4.1       rmarkdown_2.7
[33] minqa_1.2.4       farver_2.0.3      purrr_0.3.4       magrittr_2.0.1
[37] scales_1.1.1      ellipsis_0.3.1    htmltools_0.5.1.1 splines_4.0.4
[41] MASS_7.3-53       assertthat_0.2.1  colorspace_2.0-0  labeling_0.4.2
[45] utf8_1.1.4        stringi_1.5.3     munsell_0.5.0     crayon_1.3.4
```
