# Supplementary material for: Effectiveness of integrated Aedes albopictus management in southern Switzerland
Source: Parasit Vectors. 2021 Aug 16;14:405. doi: 10.1186/s13071-021-04903-2 (PMC8365973; doi:10.1186/s13071-021-04903-2)
Supplement: Supplementary file 13 — Additional file 13: Text S6. This pdf file documents the full analysis of the number of Aedes albopictus eggs collected in 2012, 2013 [14] and 2019. This pdf was created with Rmarkdown and allows for the full reproducibility of the analysis. [file 13071_2021_4903_MOESM13_ESM.pdf]

Additional file 13: Data Analysis 'Suter' plus 2019

Dr. Matteo Tanadini | Zurich Data Scientists (ZDS)

March 24, 2021

Contents

1 Reproducibility **3**

2 Loading packages **3**

3 Getting data **3**

4 Graphical Analysis **5**

4.1 AREA 5

4.2 MUNICIPALITY 6

4.3 TRAP.ID.fac 6

4.4 DATE 8

4.5 ALTITUDE 9

5 Modelling **10**

5.1 Generalised Mixed-Effects Models with zero-inflation 10

5.1.1 Fitting the model 10

5.1.2 Inspecting the overall results of the model fitting 11

5.1.3 Inspecting the estimated variabilities of the random effects 14

5.1.4 Inspecting other model coefficients 16

5.2 Visualising the model fit 16

5.2.1 Expected values 16

5.2.2 Expected values for the "counts" part 23

5.2.3 Expected values for the "zero-inflation" part 25

5.3 Quantifying effects 27

5.3.1 Ratio with expected values 27

5.3.2 Ratio with expected values for the "counts" part 29

5.3.3 Ratio with expected values for the "zero-inflation" part 30

5.4 Model checking 32

5.4.1 Checking the model equation . . . 32
5.4.2 Checking the structure of the random effects . . . 33
5.4.3 Checking normality of the random effects . . . 36
5.4.4 Checking the mean-variance relationship assumption . . . 37
5.4.5 Checking whether zero-inflation is needed . . . 38
5.4.6 Graphically evaluating the goodness-of-fit . . . 39

6 Conclusions **40**

7 Session Information **41**

1 Reproducibility

In order to make the analysis fully reproducible, we “freeze” package versions using the *{checkpoint}* package. In particular, we use all packages versions available on CRAN on first of March 2021.

```
## (in this chunk messages are omitted)
##
library(checkpoint)
checkpoint("2021-01-01",
           checkpointLocation = "../")
```

2 Loading packages

We load all add-on packages used in this analysis.

```
## (messages are omitted from this chunk)
##
library(dplyr)
library(lattice)
library(ggplot2)
library(glmmTMB)
library(lubridate)
library(gridExtra)
library(tidyr)
library(tibble)
```

3 Getting data

Note that to ensure reproducibility, the platform-agnostic file type “RDS” is used. Nevertheless, the corresponding “csv” file is also provided.

```
d.suter.plus <- readRDS("../0_Data_Preparation_For_CH_vs_I/Created_Datasets/InterventionPaper_SuterPlus
##
str(d.suter.plus)
```

```
tibble [3,351 x 9] (S3: tbl_df/tbl/data.frame)
 $ AREA        : Factor w/ 2 levels "Intervention",..: 1 1 1 1 1 1 1 1 1 1 ...
 $ MUNICIPALITY: Factor w/ 30 levels "Oligate Comasco",..: 21 21 21 21 21 21 21 21 21 21 ...
 $ TRAP.ID.fac : Factor w/ 176 levels "0018_1","0018_2",..: 1 1 1 1 1 1 1 1 1 1 ...
 $ DATE        : POSIXct[1:3351], format: "2012-07-08" "2012-07-29" ...
 $ N.ALBOPICTUS: num [1:3351] 0 0 79 61 11 5 0 0 0 0 ...
 $ ALTITUDE    : num [1:3351] 296 296 296 296 296 296 296 296 296 296 ...
 $ Year        : Factor w/ 3 levels "2012","2013",..: 1 1 1 1 1 1 1 1 1 2 ...
 $ Day         : num [1:3351] 190 211 225 239 253 267 281 309 323 143 ...
 $ study       : Factor w/ 2 levels "Flacio","Suter": 2 2 2 2 2 2 2 2 2 2 ...
```

```
print(d.suter.plus, n = 5, width = Inf)
```

```
# A tibble: 3,351 x 9
  AREA         MUNICIPALITY TRAP.ID.fac DATE                N.ALBOPICTUS
  <fct>        <fct>        <fct>       <dttm>                     <dbl>
1 Intervention Mendrisio    0018_1      2012-07-08 00:00:00            0
2 Intervention Mendrisio    0018_1      2012-07-29 00:00:00            0
3 Intervention Mendrisio    0018_1      2012-08-12 00:00:00           79
4 Intervention Mendrisio    0018_1      2012-08-26 00:00:00           61
5 Intervention Mendrisio    0018_1      2012-09-09 00:00:00           11
  ALTITUDE Year    Day study
     <dbl> <fct> <dbl> <fct>
1      296 2012    190 Suter
2      296 2012    211 Suter
3      296 2012    225 Suter
4      296 2012    239 Suter
5      296 2012    253 Suter
# ... with 3,346 more rows
```

4 Graphical Analysis

We graphically analyse the marginal effect that the available predictors have on the response variable (i.e. “number of eggs”).

4.1 AREA

The predictor *AREA* defines whether the trap is to be found in a “treated” site (i.e. “Intervention”) or not (i.e. “Non-intervention”).

Note that since very many graphs have a similar structure, we first create a “proto graph” that is then adapted for each predictor.

```
gg.proto <- ggplot(data = d.suter.plus,
                   mapping = aes(y = N.ALBOPICTUS)) +
  scale_y_sqrt() +
  geom_hline(yintercept = 0)
```

Let’s now recreate the graph for the effect of *AREA* with the response variable appropriately transformed.

```
gg.proto +
  geom_boxplot(mapping = aes(x = AREA))
```

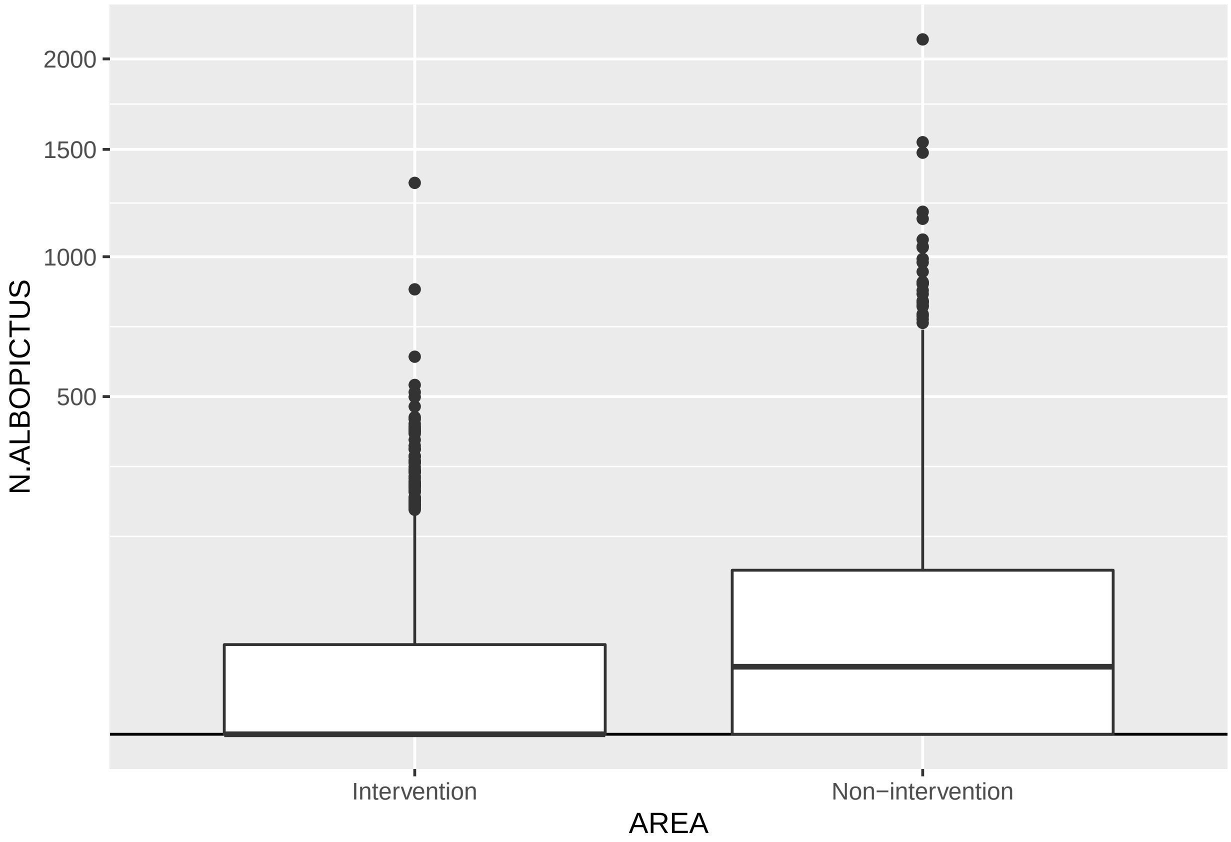

There seems to be quite a difference between the "Intervention" and the "non-intervention" groups. It is not entirely clear, whether the square-root transformation successfully stabilised the variance. Note, however, that the modelling will anyhow be done in the log-transformed space (as a negative binomial model with canonical linkn will be used).

4.2 MUNICIPALITY

```
gg.proto +
  geom_boxplot(mapping = aes(x = MUNICIPALITY,
                             colour = AREA)) +
  facet_grid(~AREA, scales = "free_x") +
  theme(axis.text.x = element_text(angle = 45))
```

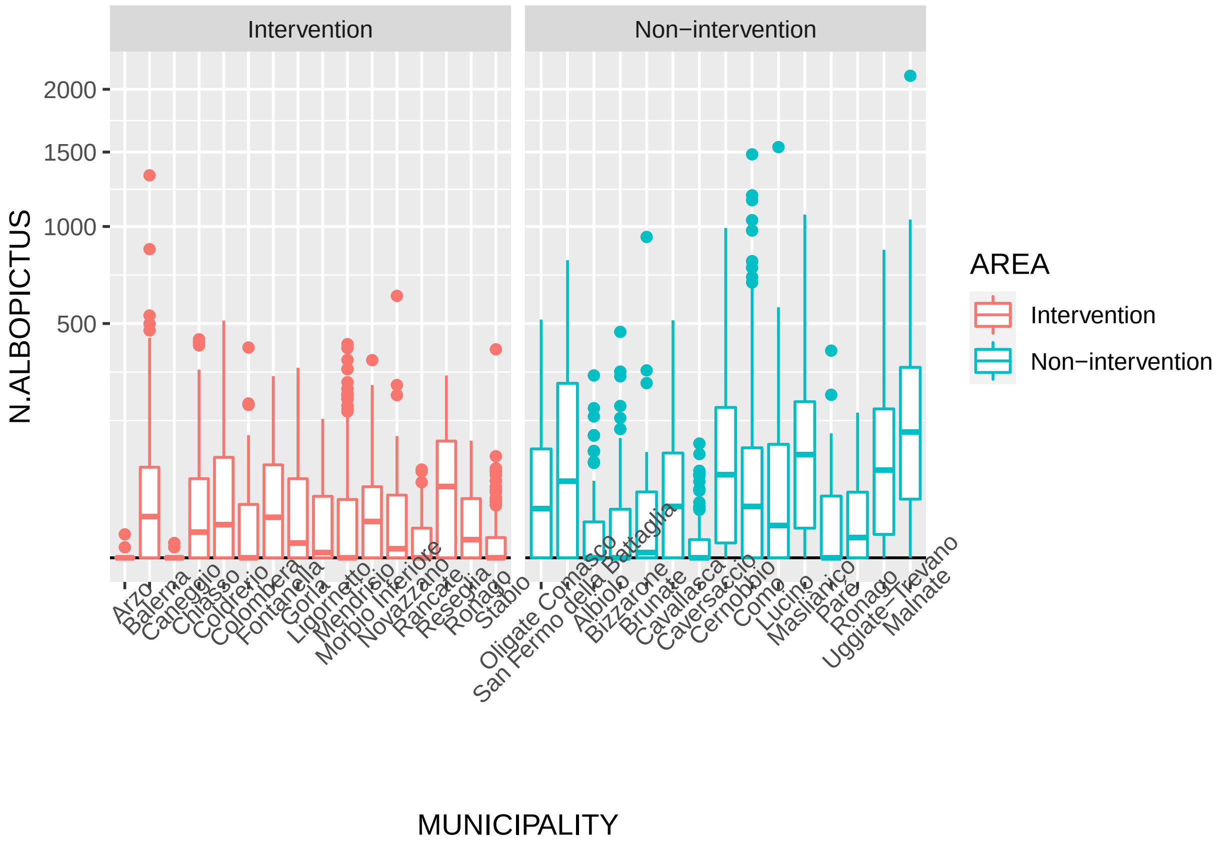

In terms of median values, there is some non-negligeable variation among municipalities that belong to the same *AREA* group. Compared to the "nr. of eggs" data, we do see more variation among municipalities that is not due to *AREA* only.

4.3 TRAP.ID.fac

```r
gg.box.traps <- gg.proto +
  geom_boxplot(mapping = aes(x = TRAP.ID.fac,
                             colour = MUNICIPALITY),
               show.legend = FALSE,
               alpha = 0.2) +
  theme(strip.text.x = element_blank()) +
  facet_grid(~AREA, scales = "free_x")
##
gg.box.traps
```

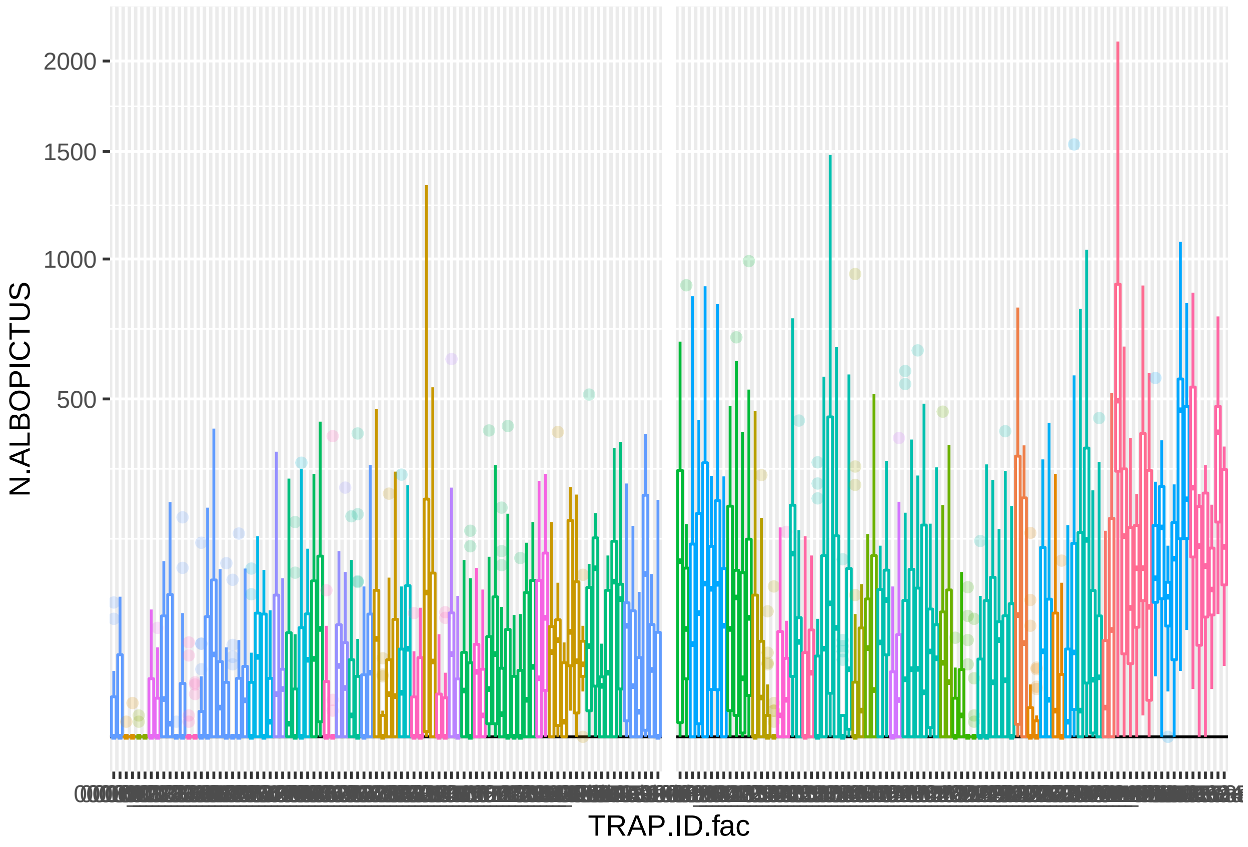

In terms of medians there is some non-negligible variation within municipalities (i.e. among traps of the same site).

Note that very median are on zero. Let's double check the proportion of zeros-present in the data.

```r
table(d.suter.plus$N.ALBOPICTUS == 0) %>% prop.table()
```

```
FALSE  TRUE
 0.56  0.44
```

About half of the observations are zeros. This does not imply per se that the data is zero-inflated (Poisson model model with low rates also lead to this situation). However, this implies that we will take zero-inflated models into consideration.

4.4 DATE

Let's include the time component in these graphs. Here we visualise the counts for each trap over time (as a solid line). Note that panelling is used to differentiate among *AREA* levels AND *Year* levels. A smoother is added in each panel.

```
## (messages are omitted in this chunk)
##
gg.all <- ggplot(data = d.suter.plus,
                 mapping = aes(y = N.ALBOPICTUS,
                               x = Day,
                               group = TRAP.ID.fac, ## trap.id.year
                               colour = AREA)) +
  geom_hline(yintercept = 0) +
  scale_y_sqrt(breaks = c(50,100,200,400,800,1600),
               minor_breaks = NULL)
##
gg.all +
  geom_line(alpha = 0.2) +
  geom_smooth(mapping = aes(group = AREA)) +
  facet_grid(~Year)
```

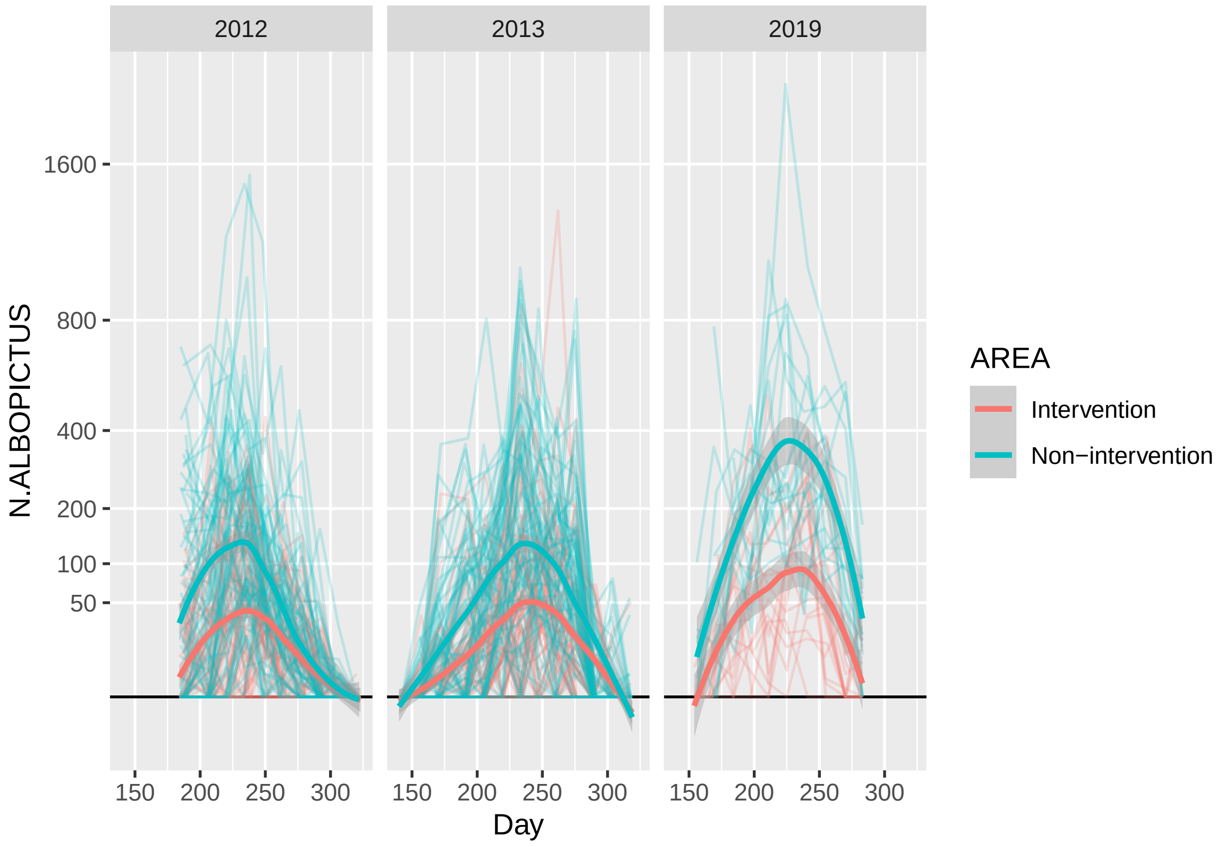

There is a clear non-linear seasonal pattern in all 6 groups. This pattern is then modelled as a quadratic effect. They seasonal shapes seem to differ among panels, which indicates the need of interaction terms.

For the main publication part we also reproduce the same graph in the original scale.

```
## (messages are omitted in this chunk)
##
gg.all +
  geom_line(alpha = 0.1) +
  geom_smooth(mapping = aes(group = AREA)) +
  facet_grid(~Year) +
  scale_y_continuous()
```

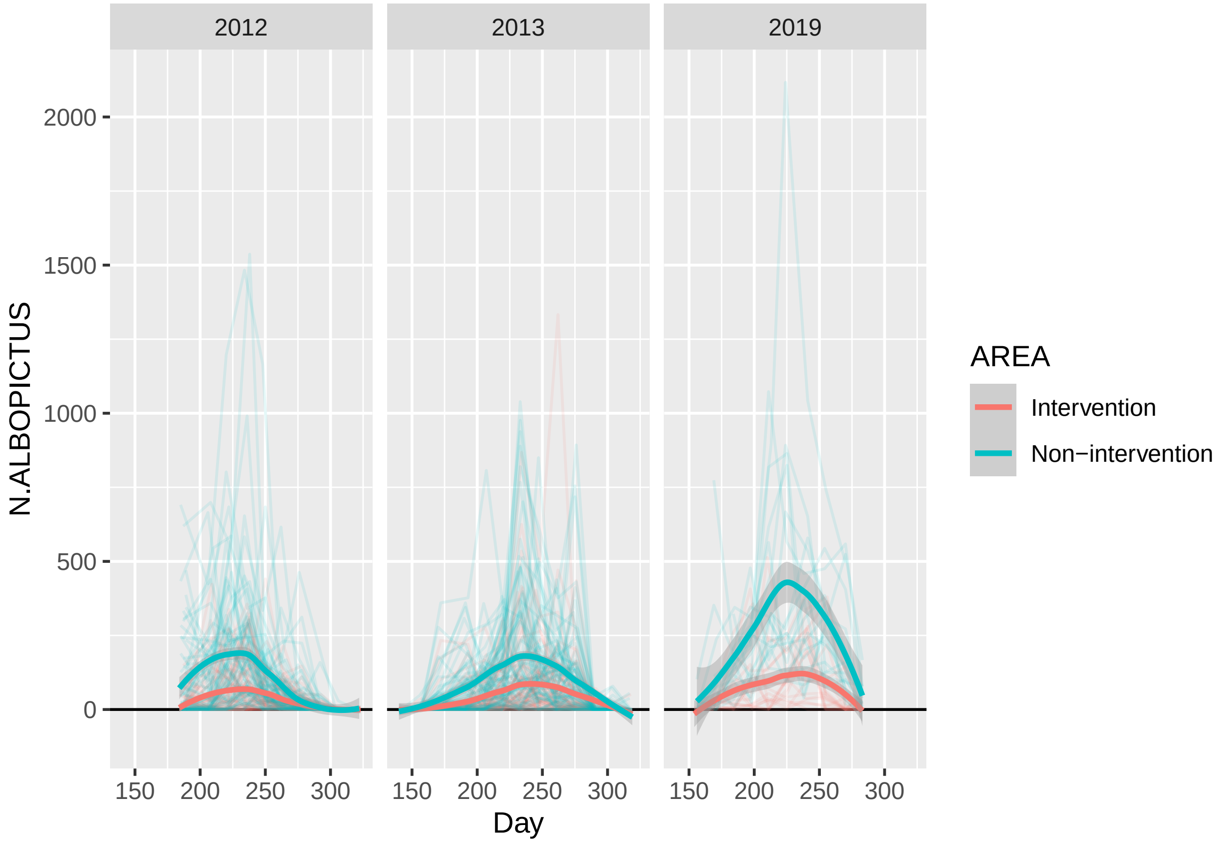

The actual counts highlights the dramatic difference between the the two treatments and how this difference has increased in 2019.

4.5 ALTITUDE

Let's visualise the marginal effect of altitude while panelling for *AREA*.

```
'geom_smooth()' using method = 'gam' and formula 'y ~ s(x, bs = "cs")'
```

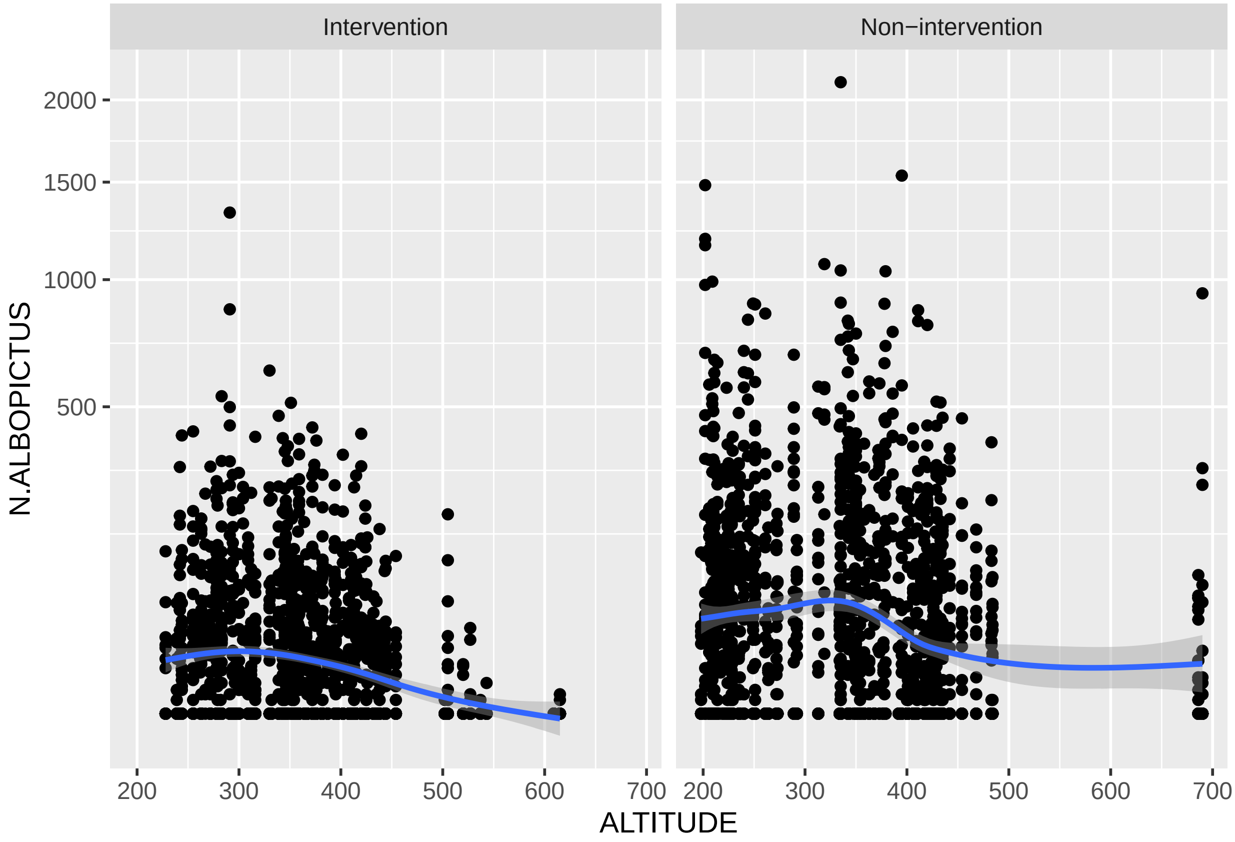

Altitude may have a slight negatice effect on counts, which makes sense biologically.

5 Modelling

5.1 Generalised Mixed-Effects Models with zero-inflation

5.1.1 Fitting the model

Let's fit the starting model. This dataset contains one additional dimension to what we have seen so far: the variable *Year*. Indeed, this data was collected in 2012, 2013 and 2019. Note, that the variable "number of days in the field" is not present in the Suter et al data.

It is important to note that we allow the model to be zero-inflated. Further down it will be formally shown that this is indeed needed. The model equation for the two parts of the model is the same.

```
## (this chunk is not evaluated)
##
system.time(
  mod.nb.1 <- glmmTMB(N.ALBOPICTUS ~ AREA *
                         scale(poly(Day, degree = 2)) *
                         Year +
                         scale(ALTITUDE) +
                         (1 | TRAP.ID.fac) + (1 | MUNICIPALITY),
                       ziformula = ~ .,
```

```r
                        family = "truncated_nbinom2",
                        data = d.suter.plus)

) ## end of system time
##
saveRDS(mod.nb.1,
        file = "Models/GlmmZINB_Suter_And_2019_TimePoly2_1.RDS")
```

The model fits in about 1 minute and there are no convergence problems.

5.1.2 Inspecting the overall results of the model fitting

Let's look at the summary.

```r
mod.nb.1 <- readRDS("Models/GlmmZINB_Suter_And_2019_TimePoly2_1.RDS")
summary(mod.nb.1)
```

```
 Family: truncated_nbinom2  ( log )
Formula:
N.ALBOPICTUS ~ AREA * scale(poly(Day, degree = 2)) * Year + scale(ALTITUDE) +
    (1 | TRAP.ID.fac) + (1 | MUNICIPALITY)
Zero inflation:                ~.
Data: d.suter.plus

     AIC      BIC   logLik deviance df.resid
   22980    23243   -11447    22894     3308

Random effects:

Conditional model:
 Groups       Name        Variance Std.Dev.
 TRAP.ID.fac  (Intercept) 0.2873   0.536
 MUNICIPALITY (Intercept) 0.0968   0.311
Number of obs: 3351, groups:  TRAP.ID.fac, 176; MUNICIPALITY, 30

Zero-inflation model:
 Groups       Name        Variance Std.Dev.
 TRAP.ID.fac  (Intercept) 0.594    0.771
 MUNICIPALITY (Intercept) 0.731    0.855
Number of obs: 3351, groups:  TRAP.ID.fac, 176; MUNICIPALITY, 30

Overdispersion parameter for truncated_nbinom2 family (): 1.16

Conditional model:
                                                            Estimate Std. Error
(Intercept)                                                   2.8663     0.1512
AREANon-intervention                                          0.5026     0.2055
scale(poly(Day, degree = 2))1                                -0.4222     0.0945
scale(poly(Day, degree = 2))2                                -1.4013     0.1288
Year2013                                                      0.6923     0.1295
Year2019                                                      0.5537     0.2745
scale(ALTITUDE)                                              -0.1670     0.0857
```

```
AREANon-intervention:scale(poly(Day, degree = 2))1           -0.5623     0.1221
AREANon-intervention:scale(poly(Day, degree = 2))2           -0.1135     0.1668
AREANon-intervention:Year2013                                -0.1469     0.1618
AREANon-intervention:Year2019                                 0.4460     0.3715
scale(poly(Day, degree = 2))1:Year2013                        0.5124     0.1292
scale(poly(Day, degree = 2))2:Year2013                        0.5379     0.1641
scale(poly(Day, degree = 2))1:Year2019                       -0.1883     0.2060
scale(poly(Day, degree = 2))2:Year2019                        0.1918     0.2592
AREANon-intervention:scale(poly(Day, degree = 2))1:Year2013   0.4235     0.1632
AREANon-intervention:scale(poly(Day, degree = 2))2:Year2013  -0.1097     0.2088
AREANon-intervention:scale(poly(Day, degree = 2))1:Year2019   0.5821     0.2608
AREANon-intervention:scale(poly(Day, degree = 2))2:Year2019  -0.0569     0.3251
                                                             z value Pr(>|z|)
(Intercept)                                                   18.95  < 2e-16
AREANon-intervention                                           2.45   0.0145
scale(poly(Day, degree = 2))1                                 -4.47  7.9e-06
scale(poly(Day, degree = 2))2                                -10.88  < 2e-16
Year2013                                                       5.35  9.0e-08
Year2019                                                       2.02   0.0437
scale(ALTITUDE)                                               -1.95   0.0514
AREANon-intervention:scale(poly(Day, degree = 2))1            -4.61  4.1e-06
AREANon-intervention:scale(poly(Day, degree = 2))2            -0.68   0.4963
AREANon-intervention:Year2013                                 -0.91   0.3638
AREANon-intervention:Year2019                                  1.20   0.2300
scale(poly(Day, degree = 2))1:Year2013                         3.97  7.3e-05
scale(poly(Day, degree = 2))2:Year2013                         3.28   0.0010
scale(poly(Day, degree = 2))1:Year2019                        -0.91   0.3607
scale(poly(Day, degree = 2))2:Year2019                         0.74   0.4593
AREANon-intervention:scale(poly(Day, degree = 2))1:Year2013    2.60   0.0095
AREANon-intervention:scale(poly(Day, degree = 2))2:Year2013   -0.53   0.5992
AREANon-intervention:scale(poly(Day, degree = 2))1:Year2019    2.23   0.0256
AREANon-intervention:scale(poly(Day, degree = 2))2:Year2019   -0.18   0.8610

(Intercept)                                                  ***
AREANon-intervention                                         *
scale(poly(Day, degree = 2))1                                ***
scale(poly(Day, degree = 2))2                                ***
Year2013                                                     ***
Year2019                                                     *
scale(ALTITUDE)                                              .
AREANon-intervention:scale(poly(Day, degree = 2))1           ***
AREANon-intervention:scale(poly(Day, degree = 2))2
AREANon-intervention:Year2013
AREANon-intervention:Year2019
scale(poly(Day, degree = 2))1:Year2013                       ***
scale(poly(Day, degree = 2))2:Year2013                       **
scale(poly(Day, degree = 2))1:Year2019
scale(poly(Day, degree = 2))2:Year2019
AREANon-intervention:scale(poly(Day, degree = 2))1:Year2013 **
AREANon-intervention:scale(poly(Day, degree = 2))2:Year2013
AREANon-intervention:scale(poly(Day, degree = 2))1:Year2019 *
AREANon-intervention:scale(poly(Day, degree = 2))2:Year2019
---
Signif. codes:  0 '***' 0.001 '**' 0.01 '*' 0.05 '.' 0.1 ' ' 1
```

```
Zero-inflation model:
                                                         Estimate Std. Error
(Intercept)                                                0.4098     0.2775
AREANon-intervention                                      -0.7837     0.3880
scale(poly(Day, degree = 2))1                             -0.4022     0.1548
scale(poly(Day, degree = 2))2                              2.1941     0.1911
Year2013                                                   0.0226     0.1674
Year2019                                                  -0.1164     0.4021
scale(ALTITUDE)                                            0.4141     0.1437
AREANon-intervention:scale(poly(Day, degree = 2))1         0.1521     0.2206
AREANon-intervention:scale(poly(Day, degree = 2))2         0.4614     0.2830
AREANon-intervention:Year2013                             -0.0878     0.2353
AREANon-intervention:Year2019                             -1.6976     0.7579
scale(poly(Day, degree = 2))1:Year2013                     0.6740     0.1965
scale(poly(Day, degree = 2))2:Year2013                     0.1027     0.2362
scale(poly(Day, degree = 2))1:Year2019                     1.7834     0.3649
scale(poly(Day, degree = 2))2:Year2019                     0.4650     0.4531
AREANon-intervention:scale(poly(Day, degree = 2))1:Year2013   0.1508     0.2729
AREANon-intervention:scale(poly(Day, degree = 2))2:Year2013  -0.5303     0.3361
AREANon-intervention:scale(poly(Day, degree = 2))1:Year2019   0.4287     0.8102
AREANon-intervention:scale(poly(Day, degree = 2))2:Year2019   0.8221     1.1280
                                                         z value Pr(>|z|)
(Intercept)                                                 1.48   0.1397
AREANon-intervention                                       -2.02   0.0434
scale(poly(Day, degree = 2))1                              -2.60   0.0094
scale(poly(Day, degree = 2))2                              11.48   <2e-16
Year2013                                                    0.14   0.8925
Year2019                                                   -0.29   0.7722
scale(ALTITUDE)                                             2.88   0.0040
AREANon-intervention:scale(poly(Day, degree = 2))1          0.69   0.4903
AREANon-intervention:scale(poly(Day, degree = 2))2          1.63   0.1031
AREANon-intervention:Year2013                              -0.37   0.7089
AREANon-intervention:Year2019                              -2.24   0.0251
scale(poly(Day, degree = 2))1:Year2013                      3.43   0.0006
scale(poly(Day, degree = 2))2:Year2013                      0.44   0.6635
scale(poly(Day, degree = 2))1:Year2019                      4.89    1e-06
scale(poly(Day, degree = 2))2:Year2019                      1.03   0.3048
AREANon-intervention:scale(poly(Day, degree = 2))1:Year2013    0.55   0.5804
AREANon-intervention:scale(poly(Day, degree = 2))2:Year2013   -1.58   0.1146
AREANon-intervention:scale(poly(Day, degree = 2))1:Year2019    0.53   0.5967
AREANon-intervention:scale(poly(Day, degree = 2))2:Year2019    0.73   0.4661

(Intercept)
AREANon-intervention                                     *
scale(poly(Day, degree = 2))1                            **
scale(poly(Day, degree = 2))2                            ***
Year2013
Year2019
scale(ALTITUDE)                                          **
AREANon-intervention:scale(poly(Day, degree = 2))1
AREANon-intervention:scale(poly(Day, degree = 2))2
AREANon-intervention:Year2013
AREANon-intervention:Year2019                            *
```

```
scale(poly(Day, degree = 2))1:Year2013                      ***
scale(poly(Day, degree = 2))2:Year2013
scale(poly(Day, degree = 2))1:Year2019                      ***
scale(poly(Day, degree = 2))2:Year2019
AREANon-intervention:scale(poly(Day, degree = 2))1:Year2013
AREANon-intervention:scale(poly(Day, degree = 2))2:Year2013
AREANon-intervention:scale(poly(Day, degree = 2))1:Year2019
AREANon-intervention:scale(poly(Day, degree = 2))2:Year2019
---
Signif. codes:  0 '***' 0.001 '**' 0.01 '*' 0.05 '.' 0.1 ' ' 1
```

Note that the p-values indicate that the three-fold interaction among *Day*, *Year* and *AREA* is clearly needed for the "conditional" part, but not for the "zero-inflation" part. We can double check this with formal inference.

```
## (this chunk is not evaluated)
##
mod.nb.2 <- update(mod.nb.1,
                   . ~ . - AREA:scale(poly(Day, degree = 2)):Year)
saveRDS(mod.nb.2,
        file = "Models/GlmmZINB_Suter_And_2019_TimePoly2_2.RDS")
```

```
mod.nb.2 <- readRDS("Models/GlmmZINB_Suter_And_2019_TimePoly2_2.RDS")
anova(mod.nb.1,
      mod.nb.2,
      test = "Chisq")
```

```
Data: d.suter.plus
Models:
mod.nb.2: N.ALBOPICTUS ~ AREA + scale(poly(Day, degree = 2)) + Year + scale(ALTITUDE) + , zi=~., disp=~
mod.nb.2:     (1 | TRAP.ID.fac) + (1 | MUNICIPALITY) + AREA:scale(poly(Day, , zi=~., disp=~1
mod.nb.2:     degree = 2)) + AREA:Year + scale(poly(Day, degree = 2)):Year, zi=~., disp=~1
mod.nb.1: N.ALBOPICTUS ~ AREA * scale(poly(Day, degree = 2)) * Year + scale(ALTITUDE) + , zi=~., disp=~
mod.nb.1:     (1 | TRAP.ID.fac) + (1 | MUNICIPALITY), zi=~., disp=~1
         Df   AIC   BIC logLik deviance Chisq Chi Df Pr(>Chisq)
mod.nb.2 35 22978 23192 -11454    22908
mod.nb.1 43 22980 23243 -11447    22894  13.9      8      0.085 .
---
Signif. codes:  0 '***' 0.001 '**' 0.01 '*' 0.05 '.' 0.1 ' ' 1
```

As expected the p-value is borderline. In order to keep the structure of both model parts identical we don't drop the three-fold interaction from the zero-inflation part.

5.1.3 Inspecting the estimated variabilities of the random effects

Let's inspect the estimates for the variance components.

```
VarCorr(mod.nb.1)
```

```
Conditional model:
 Groups       Name        Std.Dev.
```

```
 TRAP.ID.fac  (Intercept) 0.536
 MUNICIPALITY (Intercept) 0.311

Zero-inflation model:
 Groups       Name        Std.Dev.
 TRAP.ID.fac  (Intercept) 0.771
 MUNICIPALITY (Intercept) 0.855
```

Apparently, for both model parts, the two random effects *TRAP.ID,fac* and *MUNICIPALITY* are of similar magnitude. To better compare the estimates we should look at their confidence intervals.

The code to estimate these confidence intervals is quite long and cumbersome (the default function to get confidence from the *{glmmTMB}* intervals requires some tweaks when dealing with zero-inflated negative binomial models). Therefore, the code is omitted here, but available as an **R** file.

```
Warning: Removed 1 rows containing missing values (geom_point).

Warning: Removed 1 row(s) containing missing values (geom_path).
```

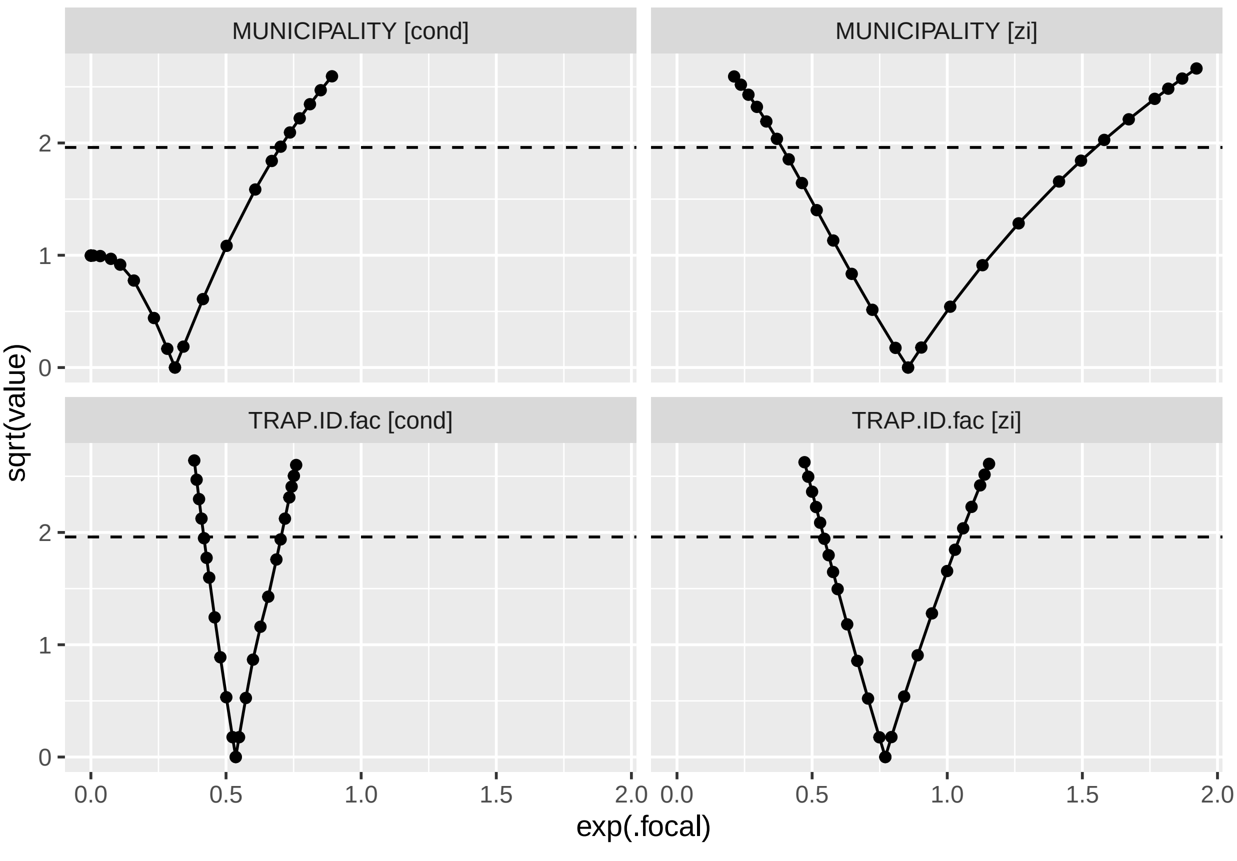

The "profile z-plot" shows a few interesting patterns:

- As expected, the confidence intervals for *MUNICIPALITY* are larger thano those for *TRAP.ID.fac*. This is explained by the larger number of levels (there are 176 traps and 30 municipalities).

- The 95% confidence interval for the random effect *MUNICIPALITY* in the conditional part of the model (i.e. counts) contains zero.
- Its point estimate is smaller than the one for *TRAP.ID.fac* for the conditional part. Its confidence interval is also close to zero than the one for *TRAP.ID.fac* counts part.
- The opposite is true for the zero-inflation part of the model.
- Indeed, for the zero-inflation part, *MUNICIPALITY* has a larger point estimate than *TRAP.ID.fac*. Its confidence interval is generally more far away from zero.

To translate these results about the estimated variabilities of the random effects and their associated confidence intervals into biological results:

- **presence/absence is a process regulated at a larger spatial scale. Indeed, *MUNICIPALITY* play a more relevant role here.**
- **On the other hand, abundance is a process regulated at a lower spatial scale. Indeed, *TRAP.ID.fac* play a more relevant role.**

5.1.4 Inspecting other model coefficients

Note that the coefficients for the zero-inflation part refer to the probability of the response being zero. This may somehow appear counterintuitive (other packages implemented this differently). We inspect the coefficient for *ALTITUDE* to make an example:

```
fixef(mod.nb.1)$cond["scale(ALTITUDE)"]
```

```
scale(ALTITUDE)
          -0.17
```

So, here we can conclude that altitude has a negative effect on the number of eggs.

Let's now turn our attention to the regression coefficient of *ALTITUDE* for the zero-inflation part.

```
fixef(mod.nb.1)$zi["scale(ALTITUDE)"]
```

```
scale(ALTITUDE)
           0.41
```

In this case, the coefficient is negative. This is to interpreted as the probability of absences increases with altitude. This is in agreement with the interpreation for the "counts" part. In both cases, higher altitudes are associated with less favourable conditions.

5.2 Visualising the model fit

5.2.1 Expected values

We start by visualising the fitted values of the model (in red) along with the observed values (in gray). Note that we visualise the expected value here. For a zero-inlfated model this is $\mu * (1 - p)$. All these plots still use a square-root transformed y-axis.

```
## (warnings are omitted from this chunk)
##
d.suter.plus$fit.mod.nb.1 <- fitted(mod.nb.1)
## equivalent to predict(., type = "response")
##
ggplot(data = d.suter.plus,
       mapping = aes(y = N.ALBOPICTUS,
                     x = Day,
                     group = TRAP.ID.fac)) +
  scale_y_sqrt() +
  geom_line(alpha = 0.2) +
  facet_grid(AREA ~ Year) +
  geom_line(mapping = aes(y = fit.mod.nb.1),
            col = "red",
            alpha = 0.2)
```

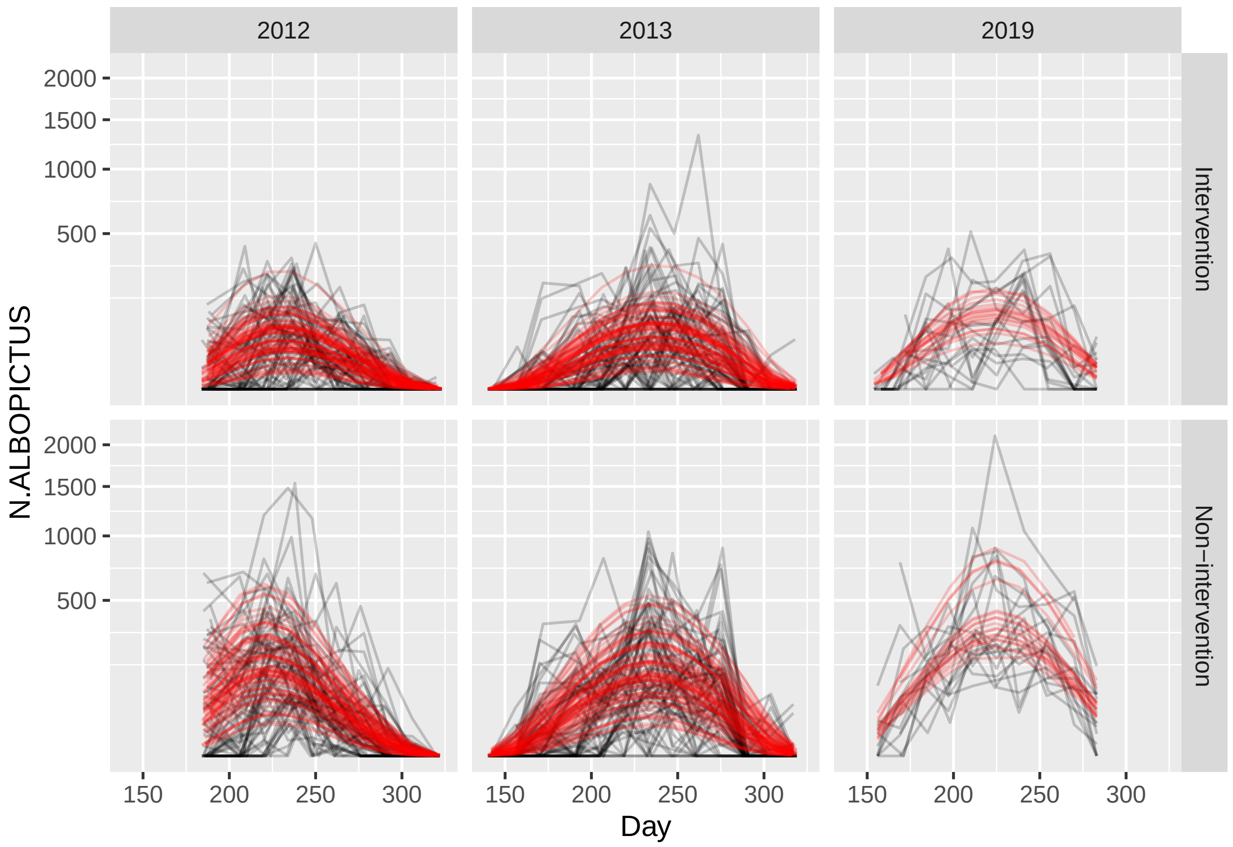

The model fits looks quite reasonable. Note that the warnings omitted here are harmless (see developers comments about these warnings here).

In the next graph we are going to show the predictions at population level (i.e. without the estimated random effects). In addition, we keep we make predictions on a finer grid and set *ALTITUDE* to its mean value.

```
## (warnings and messages are omitted from this chunk)
## (results are omitted from this chunk)
##
## 0. Preparing data
d.year.ranges <- d.suter.plus %>%
  group_by(Year) %>%
  summarise(min.date = min(DATE),
            max.date = max(DATE)) %>%
  as.data.frame()
##
d.year.ranges
##
##
## 1. Creating the prediction dataset with expand.grid()
d.predict <- expand.grid(
  AREA = levels(d.suter.plus$AREA),
  # Year = levels(d.suter.plus$Year),
  DATE = c(
    ## 2012
    seq(from = d.year.ranges[1,2],
        to = d.year.ranges[1,3],
    by = "day"),
    ## 2013
        seq(from = d.year.ranges[2,2],
        to = d.year.ranges[2,3],
    by = "day"),
    ## 2019
    seq(from = d.year.ranges[3,2],
        to = d.year.ranges[3,3],
    by = "day")),
  ALTITUDE = mean(d.suter.plus$ALTITUDE),
  ## NB: predict.glmmTMB() requires RE even for pop-level predictions.
  TRAP.ID.fac = "BAL-11a",
  MUNICIPALITY = "Balerna")
##
dim(d.predict) ## There are 450 days in this range (x2 for AREA)
str(d.predict)
##
## 2. Converting Date to Day and Year
d.predict$Day <- yday(d.predict$DATE)
d.predict$Year <- year(d.predict$DATE)
##
dim(d.predict)
str(d.predict)
##
## 3. Making predictions at population level
d.predict$pred.pop.level.mod.nb.1 <- predict(mod.nb.1,
                                   type = "response",
                                   newdata = d.predict,
                                   re.form = NA)
##
## 4. Producing the graph
ggplot(data = d.suter.plus,
```

```
      mapping = aes(y = N.ALBOPICTUS,
                    x = Day)) +
  scale_y_sqrt(breaks = c(0, 50,100,150,200,250,300,
                          350,500,1000,2000),
               minor_breaks = FALSE) +
  geom_line(mapping = aes(group = TRAP.ID.fac),
            alpha = 0.05) +
  facet_wrap(~ Year) +
  ## adding pop-level predictions
  geom_line(data = d.predict,
            size = 1,
            mapping = aes(y = pred.pop.level.mod.nb.1,
                          col = AREA))
```

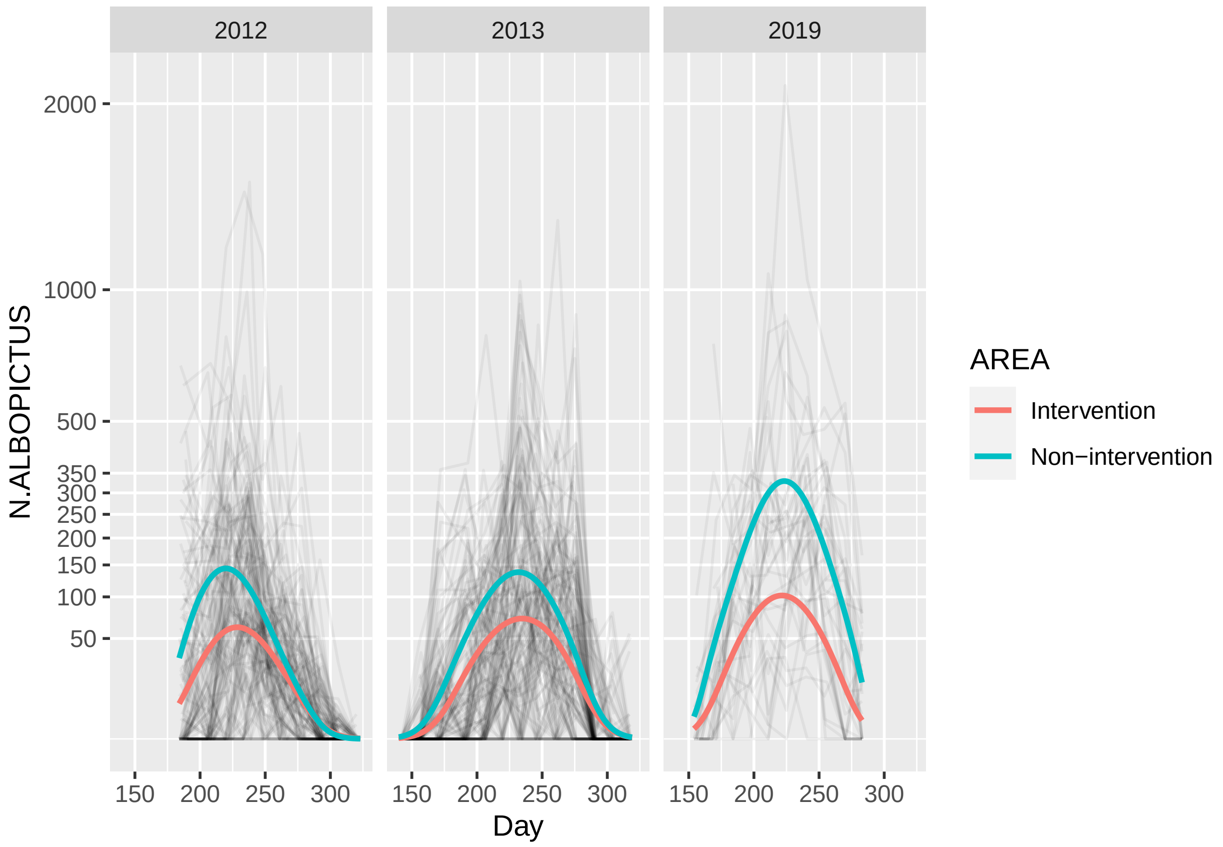

This graph clearly highlights the fact that the absolute number of insects has increased from 2012-2013 to 2019 (mind the y-scale!).

Let's reproduce the same graph in the "natural" untransformed scale.

```
ggplot(data = d.suter.plus,
      mapping = aes(y = N.ALBOPICTUS,
                    x = Day)) +
  geom_line(mapping = aes(group = TRAP.ID.fac),
            alpha = 0.05) +
```

```
  facet_wrap(~ Year) +
  ## adding pop-level predictions
  geom_line(data = d.predict,
            size = 1,
            mapping = aes(y = pred.pop.level.mod.nb.1,
                          col = AREA))
```

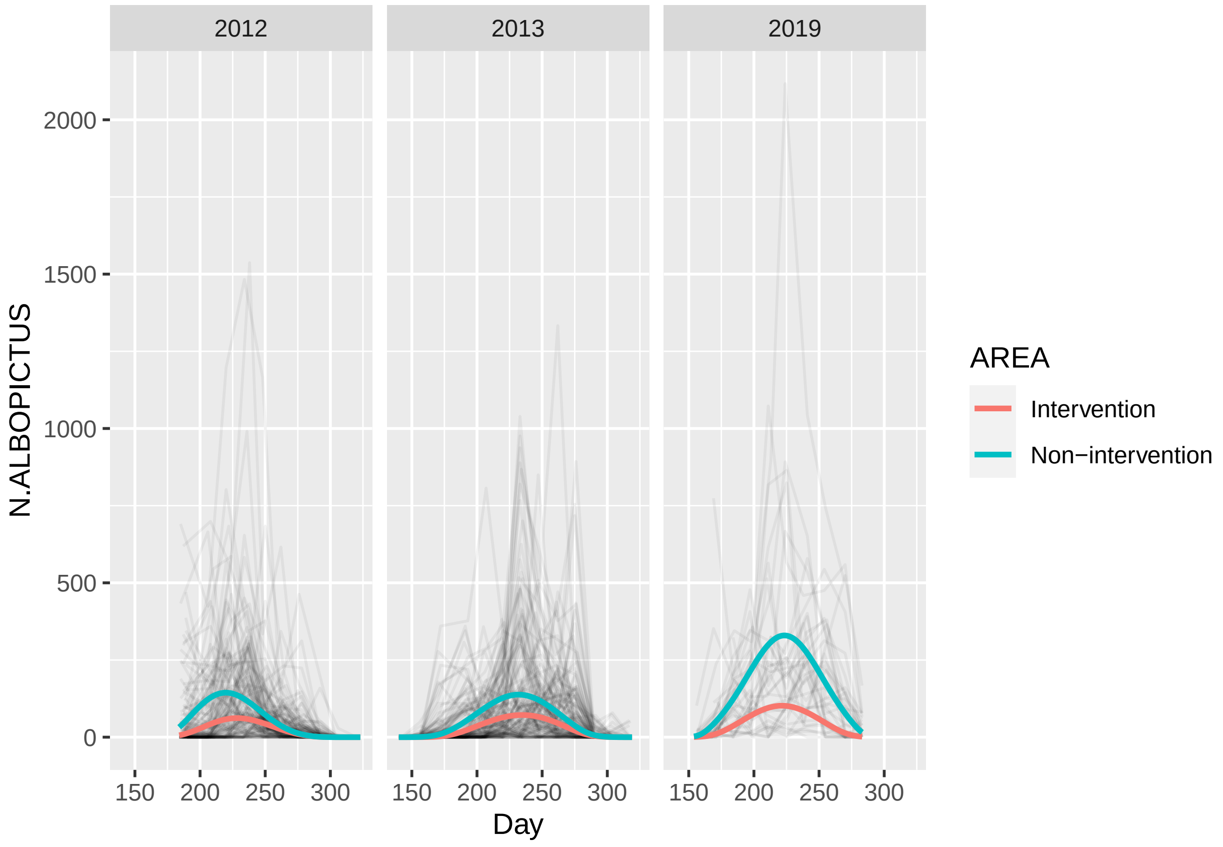

Due to the skewness of the data, we can't really appreciate the changes in the area of interest (i.e. between 0 and 400 counts). We therefore, zoom in to better compare curves.

```
ggplot(data = d.suter.plus,
       mapping = aes(y = N.ALBOPICTUS,
                     x = Day)) +
  scale_y_continuous(breaks = c(0,50,75,100,150,200,250,300,350,400),
                     minor_breaks = FALSE) +
  geom_line(mapping = aes(group = TRAP.ID.fac),
            alpha = 0.05) +
  facet_wrap(~ Year) +
  ## adding pop-level predictions
  geom_line(data = d.predict,
            size = 1,
            mapping = aes(y = pred.pop.level.mod.nb.1,
                          col = AREA)) +
   coord_cartesian(ylim = c(0, 400)) ## the "zoom"
```

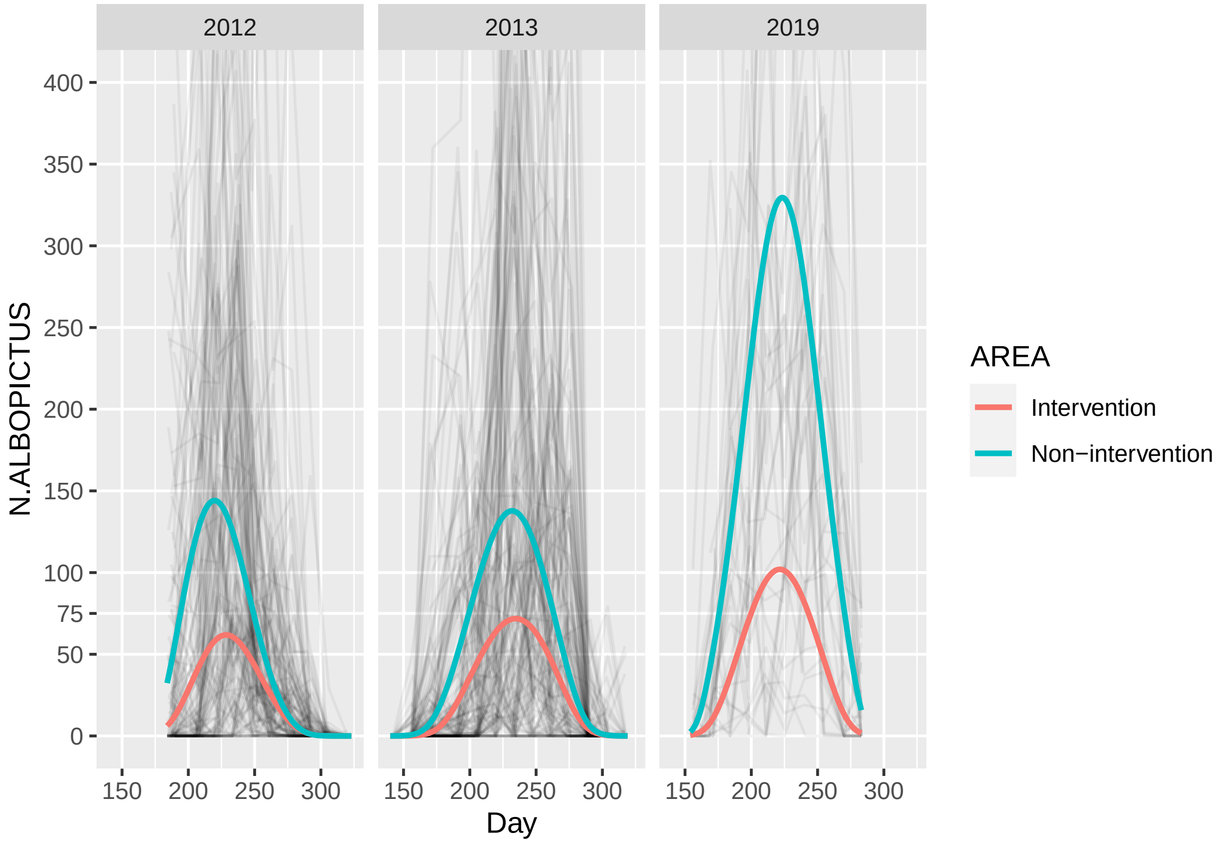

This graph highlights once again the dramatic increase of counts between 2012-2013 and 2019. In particular, the increase in counts over time is massively higher in "Non-intervention" sites than in "Non-intervention" sites.

Note, that despite the zooming, the absolute differences are biologically very relevant! Indeed, the peak in the "Non-intervention" sites goes from less than 150 in 2012 and 2013 to about 330 in 2019. On the other hand, the increase for "Intervention" sites is much more moderate (i.e. from about 65 to about 100). See exact number at peak below.

```
## (messages are omitted from this chunk)
##
d.predict %>%
  group_by(Year, AREA) %>%
  summarise(Max = max(pred.pop.level.mod.nb.1))
```

```
# A tibble: 6 x 3
# Groups:   Year [3]
   Year AREA                Max
  <dbl> <fct>             <dbl>
1  2012 Intervention      61.8
2  2012 Non-intervention 144.
3  2013 Intervention      71.8
4  2013 Non-intervention 138.
5  2019 Intervention     102.
6  2019 Non-intervention 330.
```

Keep in mind that ratio between "Non-intervention" and "Intervention" are not necessarily maximal at peak. See further down.

Same graph with Dates on the x-axis.

```
ggplot(data = d.suter.plus,
       mapping = aes(y = N.ALBOPICTUS,
                     x = DATE)) +
  scale_y_continuous(breaks = c(0,50,75,100,150,200,250,300,350,400),
               minor_breaks = FALSE) +
  geom_line(mapping = aes(group = TRAP.ID.fac),
            alpha = 0.05) +
  facet_wrap(~ Year, scales = "free_x") +
  ## adding pop-level predictions
  geom_line(data = d.predict,
            size = 1,
            mapping = aes(y = pred.pop.level.mod.nb.1,
                          col = AREA)) +
   coord_cartesian(ylim = c(0, 400)) ## the "zoom"
```

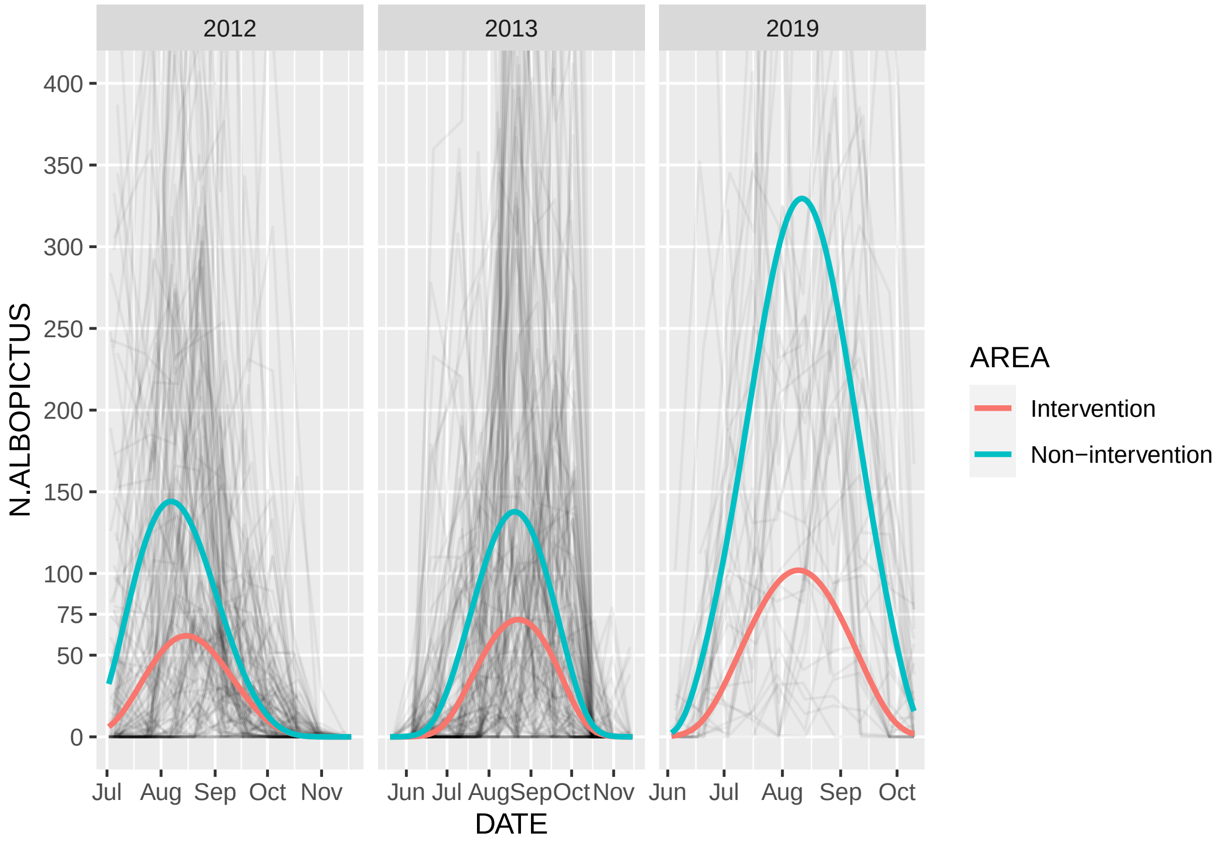

Note that on the above graph, the x-range is adapted to each panel.

5.2.2 Expected values for the "counts" part

Let's first visualise the expected value for the "counts" part.

```
## (warnings are omitted from this chunk)
##
## Fitted values with RE
d.suter.plus$fit.mod.nb.1.conditional <- predict(mod.nb.1,
                                     type = "conditional")
##
## Fitted values withOUT RE (population level)
d.predict$pred.pop.level.mod.nb.1.conditional <- predict(mod.nb.1,
                                   type = "conditional",
                                   newdata = d.predict,
                                   re.form = NA)
##
## Graph
ggplot(data = d.suter.plus,
       mapping = aes(y = fit.mod.nb.1.conditional,
                     x = Day,
                     group = TRAP.ID.fac)) +
  scale_y_sqrt(breaks = 0:10*100) +
  geom_line(alpha = 0.2) +
  facet_grid(AREA ~ Year) +
  geom_line(data = d.predict,
            mapping = aes(y = pred.pop.level.mod.nb.1.conditional),
            colour = "red")
```

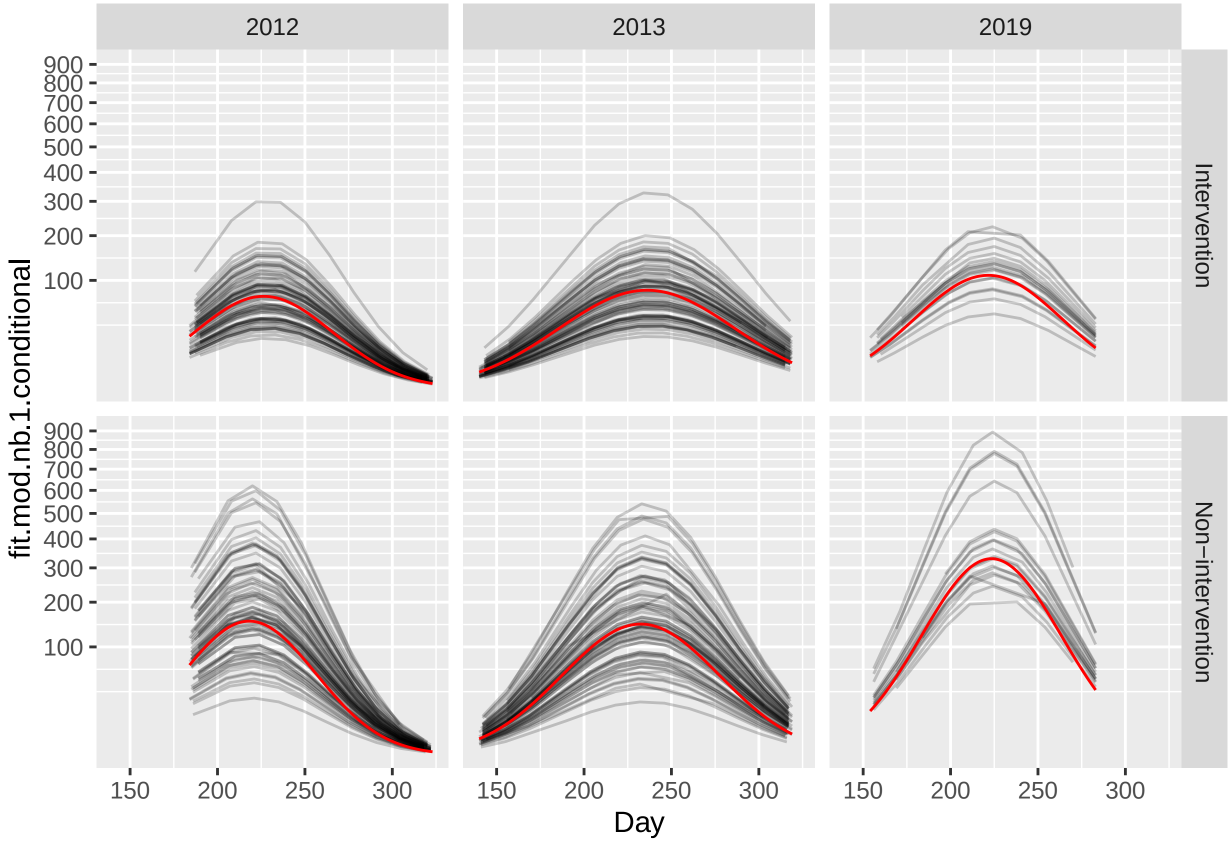

There is quite some variability among traps. Remember that this variability is equivally explained by trap-level variation as well as municipality-level variability.

We may want to dig deeper and look at the variability broken down in traps and municipality.

```
d.suter.plus$Year..Trap <- interaction(d.suter.plus$Year,
                                       d.suter.plus$TRAP.ID.fac,
                                       drop = TRUE)
##
ggplot(data = d.suter.plus,
       mapping = aes(y = fit.mod.nb.1.conditional,
                     x = Day,
                     group = Year..Trap,
                     colour = Year)) +
  geom_hline(yintercept = c(0,1)) +
  geom_line(alpha = 0.7) +
  facet_wrap(~ MUNICIPALITY)
```

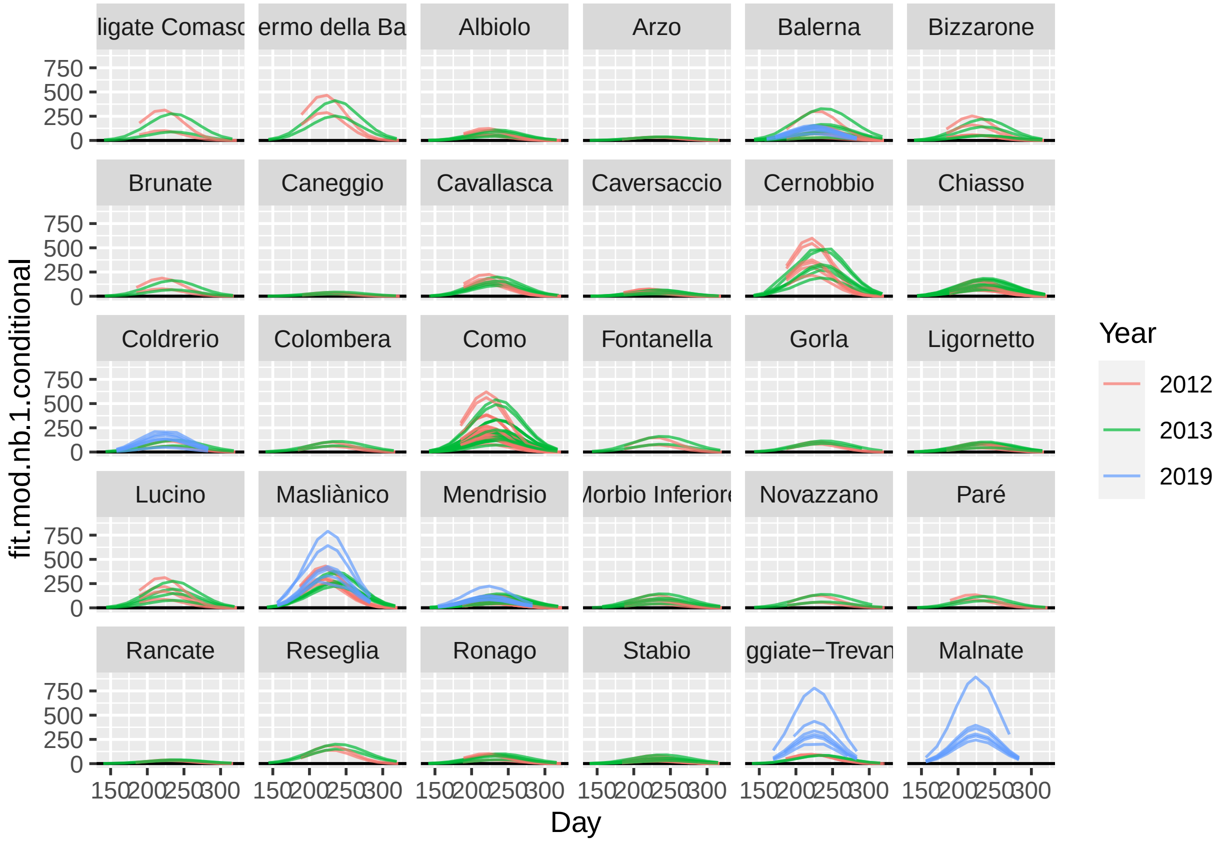

For the counts part, the intra-municipality variability seems pretty much to depend on the mean value only. In other words, municipalities with high mean values show high variability. This is expected from the multiplicative nature of the counts model (link function is the natural-logarithm). However, due to the trap random effects, it would be theoretically possible that municipalities with the same mean, have diffenent variabilities. This, indeed, what happens for the "zero-inflation" part.

5.2.3 Expected values for the "zero-inflation" part

Let's now turn our attention to the fitted values for the probability of eggs absence. Remember that the model fitted to the "zero-inflation" part is a binary Mixed-Effects Model (with canonical link).

```
## (warnings are omitted from this chunk)
##
## Fitted values with RE
d.suter.plus$fit.mod.nb.1.zprob <- predict(mod.nb.1,
                                           type = "zprob")
##
## Fitted values withOUT RE (population level)
d.predict$pred.pop.level.mod.nb.1.zprob <- predict(mod.nb.1,
                                          type = "zprob",
                                          newdata = d.predict,
                                          re.form = NA)
##
## Graph
ggplot(data = d.suter.plus,
```

```
      mapping = aes(y = fit.mod.nb.1.zprob,
                    x = Day,
                    group = TRAP.ID.fac)) +
  geom_hline(yintercept = c(0,1)) +
  geom_line(alpha = 0.2) +
  facet_grid(AREA ~ Year) +
  geom_line(data = d.predict,
            mapping = aes(y = pred.pop.level.mod.nb.1.zprob),
            colour = "red")
```

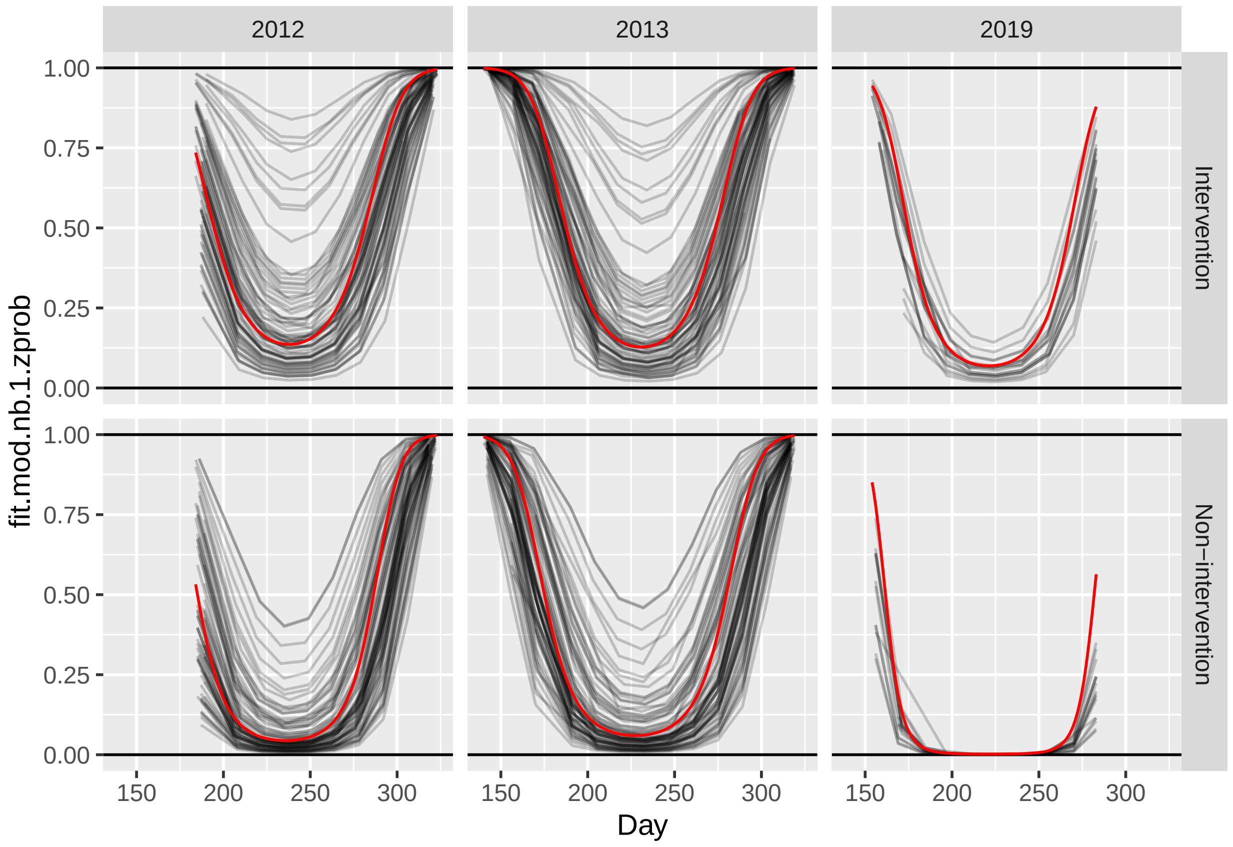

Interestingly, the variation between traps is extremely high in years 2012 and 2013. Indeed, some traps have probability of absence close to 90%, while others close to 0%. In the 2019 the situation changes dramatically. Essentially all traps have very low probability of absences. This is particular true for "Non-intervention" site where the probability of absence is virtually zero.

Again, we can dig deeper and break down this variability for traps and municipality.

```
ggplot(data = d.suter.plus,
      mapping = aes(y = fit.mod.nb.1.zprob,
                    x = Day,
                    group = Year..Trap,
                    colour = Year)) +
  geom_hline(yintercept = c(0,1)) +
```

```
  geom_line(alpha = 0.7) +
  facet_wrap(~ MUNICIPALITY)
```

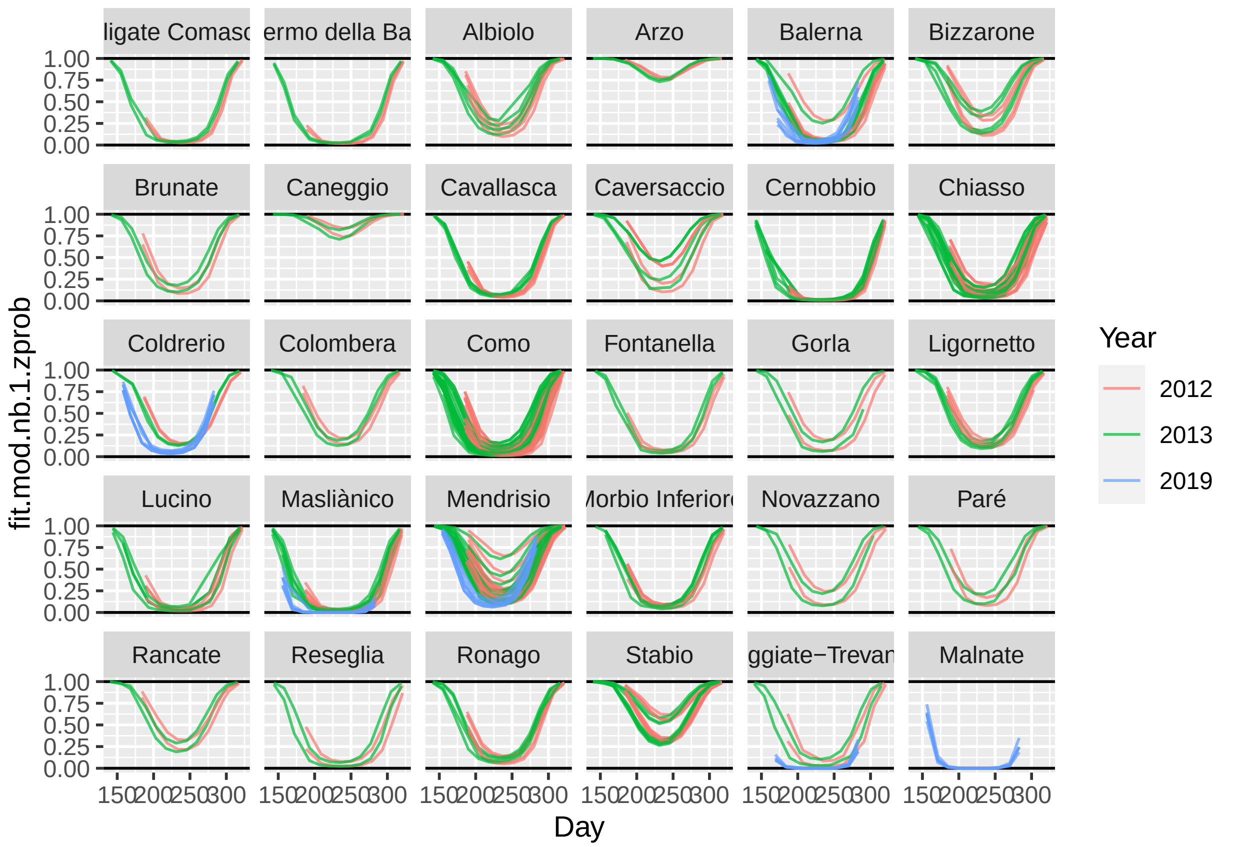

Some municipalities have very high probabilities of absences (e.g. Arzo). Note also that the intra-municipality variation can change a lot. Compare for example "Como" and "Mendrisio".

5.3 Quantifying effects

5.3.1 Ratio with expected values

In this model an interaction is present, therefore, we cannot simply look at the *Non-intervention* coefficient to evaluate the differences between "Non-intervention" and "Intervention". In particular, in this model the difference between the two groups changes over time and differ among years.

Let's visualise how the ratio between the two groups changes over time for each year. We first adapt the *d.predict* dataset and compute the ratio between predicted number of adults in "Non-intervention" and "Intervention".

```
## (results and warnings are omitted from this chunk)
##
d.predict.short.wide <- d.predict %>%
  select(AREA, pred.pop.level.mod.nb.1,
         Year, DATE, Day) %>%
```

```
  spread(key = AREA,
         value = pred.pop.level.mod.nb.1)
##
# dim(d.predict.short.wide)
# head(d.predict.short.wide)
# tail(d.predict.short.wide)
##
d.predict.short.wide$ratio <- d.predict.short.wide$`Non-intervention` /
  d.predict.short.wide$Intervention
##
# head(d.predict.short.wide)
# tail(d.predict.short.wide)
```

We the visualise the ratio evolution over time.

```
ggplot(data = d.predict.short.wide,
       mapping = aes(y = ratio,
                     x = Day)) +
  geom_line(col = "red") +
  geom_hline(yintercept = 1) + ## H0: ratio is 1
  facet_grid(~Year)
```

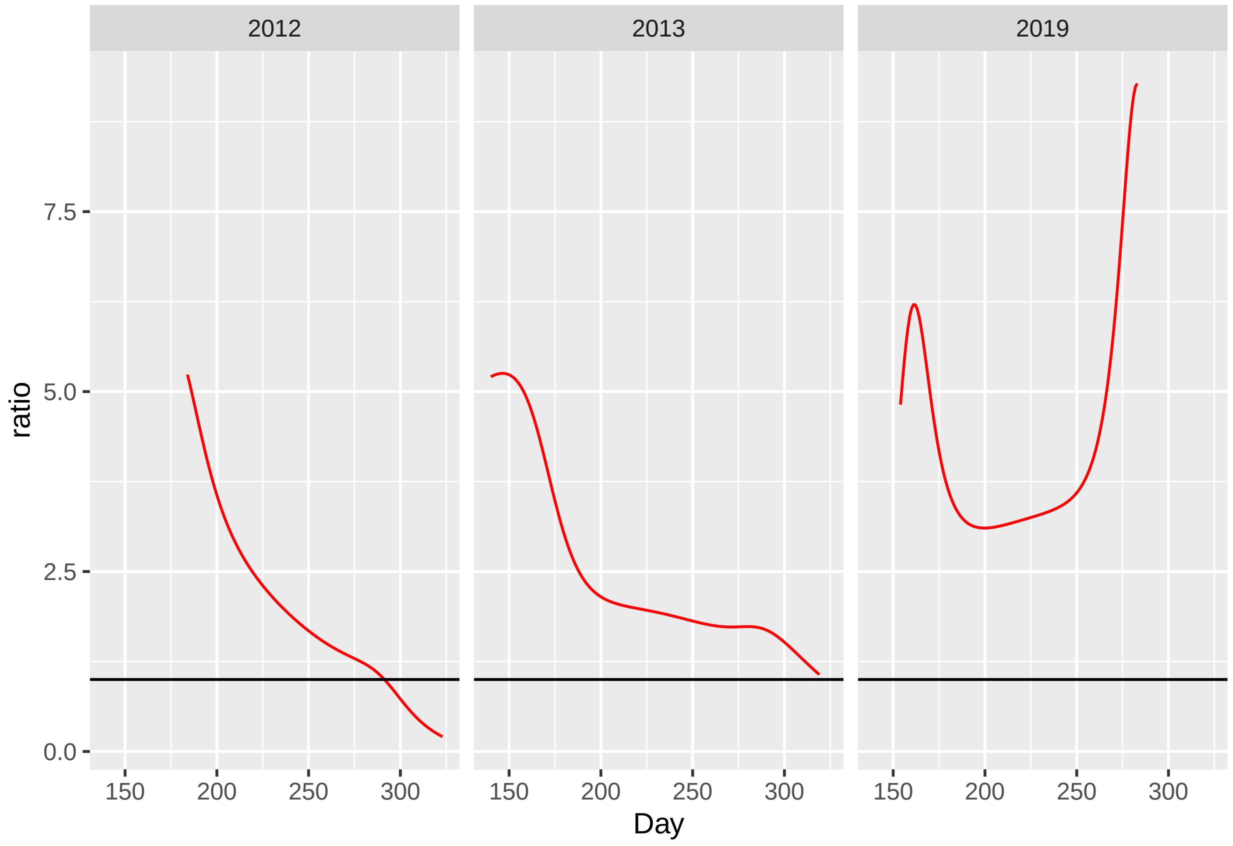

This graph clearly shows that the ratio between "Non-Intervention" and "Intervention" inreases from 2013 to and to 2019 (on average it is always higher). Interestingly, the shape of the ratio also changes over time.

5.3.2 Ratio with expected values for the "counts" part

```
## (warnings are omitted from this chunk)
##
d.predict$pred.pop.level.mod.nb.1.cond <- predict(
  mod.nb.1,
  type = "cond",
  newdata = d.predict,
  re.form = NA)

d.predict.short.wide.cond <- d.predict %>%
  select(AREA, pred.pop.level.mod.nb.1.cond,
         Year, DATE, Day) %>%
  spread(key = AREA,
         value = pred.pop.level.mod.nb.1.cond)
##
head(d.predict.short.wide.cond)
```

```
  Year       DATE Day Intervention Non-intervention
1 2012 2012-07-02 184           23               69
2 2012 2012-07-03 185           25               72
3 2012 2012-07-04 186           26               76
4 2012 2012-07-05 187           27               79
5 2012 2012-07-06 188           29               82
6 2012 2012-07-07 189           30               86
```

```
anyNA(d.predict.short.wide.cond)
```

```
[1] FALSE
```

```
##
d.predict.short.wide.cond$ratio.cond <- d.predict.short.wide.cond$`Non-intervention` /
  d.predict.short.wide.cond$Intervention


ggplot(data = d.predict.short.wide.cond,
       mapping = aes(y = ratio.cond,
                     x = Day)) +
  geom_line(col = "red") +
  # scale_y_continuous(limits = c(0,6.5)) +
  geom_hline(yintercept = 1) + ## H0: ratio is 1
  facet_grid(~Year)
```

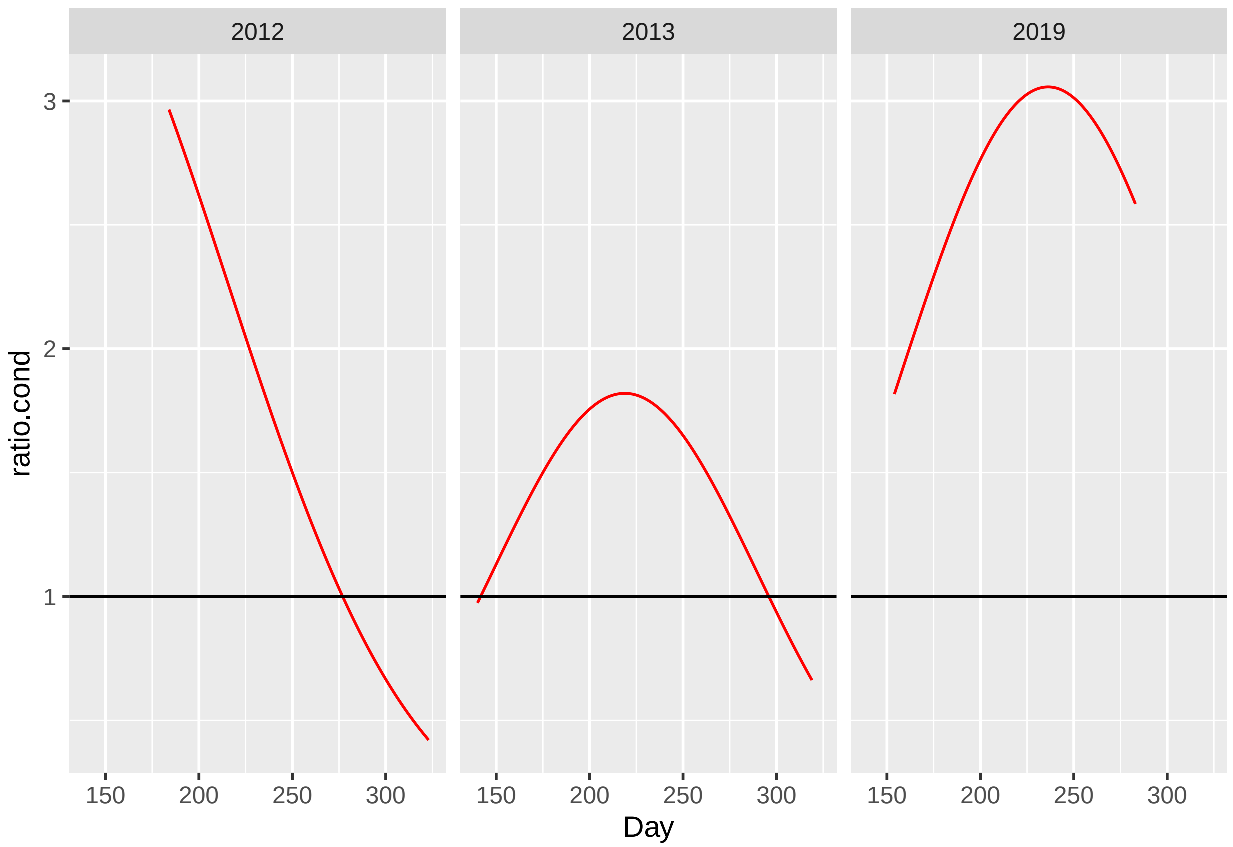

The ratio for the counts part seem to increase from 2012-2013 to 2019.

5.3.3 Ratio with expected values for the "zero-inflation" part

We reproduce now the same graph for the "zero-inflation" part. Note that for the sake of clearness, we display the ratio of probabilities of presences rather than absences.

```
## (warnings are omitted from this chunk)
##
d.predict$pred.pop.level.mod.nb.1.zi <- predict(
  mod.nb.1,
  type = "zprob",
  newdata = d.predict,
  re.form = NA)

d.predict.short.wide.zi <- d.predict %>%
  select(AREA, pred.pop.level.mod.nb.1.zi,
         Year, DATE, Day) %>%
  spread(key = AREA,
         value = pred.pop.level.mod.nb.1.zi)
##
head(d.predict.short.wide.zi)
```

```
  Year       DATE Day Intervention Non-intervention
```

```
1 2012 2012-07-02 184         0.74             0.53
2 2012 2012-07-03 185         0.71             0.50
3 2012 2012-07-04 186         0.69             0.47
4 2012 2012-07-05 187         0.67             0.44
5 2012 2012-07-06 188         0.65             0.41
6 2012 2012-07-07 189         0.63             0.39
```

```
anyNA(d.predict.short.wide.zi)
```

```
[1] FALSE
```

```
##
d.predict.short.wide.zi$ratio.zi <- d.predict.short.wide.zi$Intervention /
  d.predict.short.wide.zi$`Non-intervention` ## note: opposite here!


ggplot(data = d.predict.short.wide.zi,
       mapping = aes(y = ratio.zi,
                     x = Day)) +
  scale_y_continuous(breaks = c(0,1:5,1:4* 10),
                     minor_breaks = FALSE) +
  geom_line(col = "red") +
  # scale_y_continuous(limits = c(0,6.5)) +
  geom_hline(yintercept = 1) + ## H0: ratio is 1
  facet_grid(~Year)
```

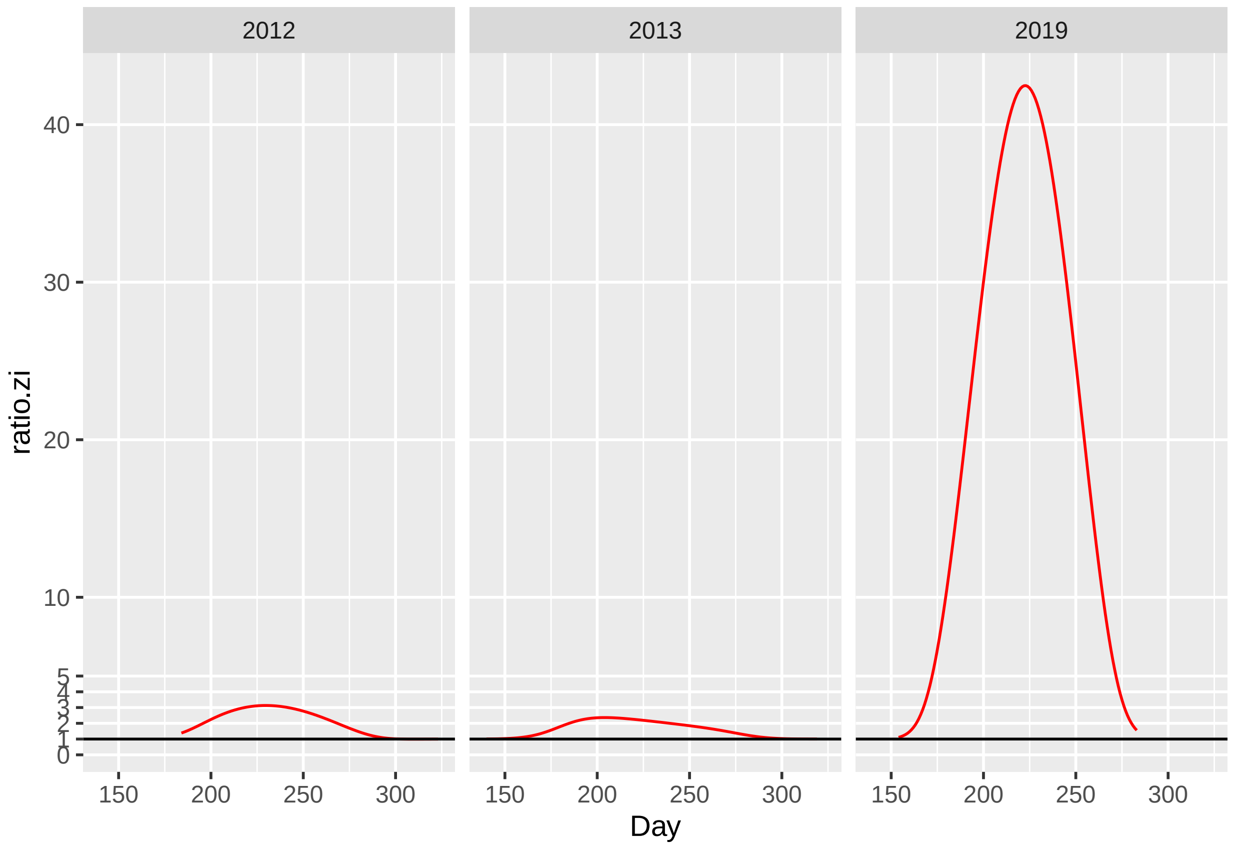

Interestingly, the ratio explodes in 2019. If the probability to have a presence in 2012 and 2013 was about was 2-3 times higher in the "Non-Intervention" sites, this probability jumps to about 40 in 2019.

5.4 Model checking

5.4.1 Checking the model equation

Let's check the assumption that, in the linear predictor scale, the effect of *Day* is quadratic. To test hypotheses we use the "response" residuals (nothing else is implemented for ZINB models).

```
## (messages and warnings are omitted from this chunk)
##
d.suter.plus$res.mod.nb.1 <- resid(mod.nb.1)
##
ggplot(data = d.suter.plus,
       mapping = aes(y = res.mod.nb.1,
                     x = Day)) +
  geom_hline(yintercept = 0) +
  geom_point() +
  facet_wrap(Year ~ AREA, scales = "free_x") +
  geom_smooth()
```

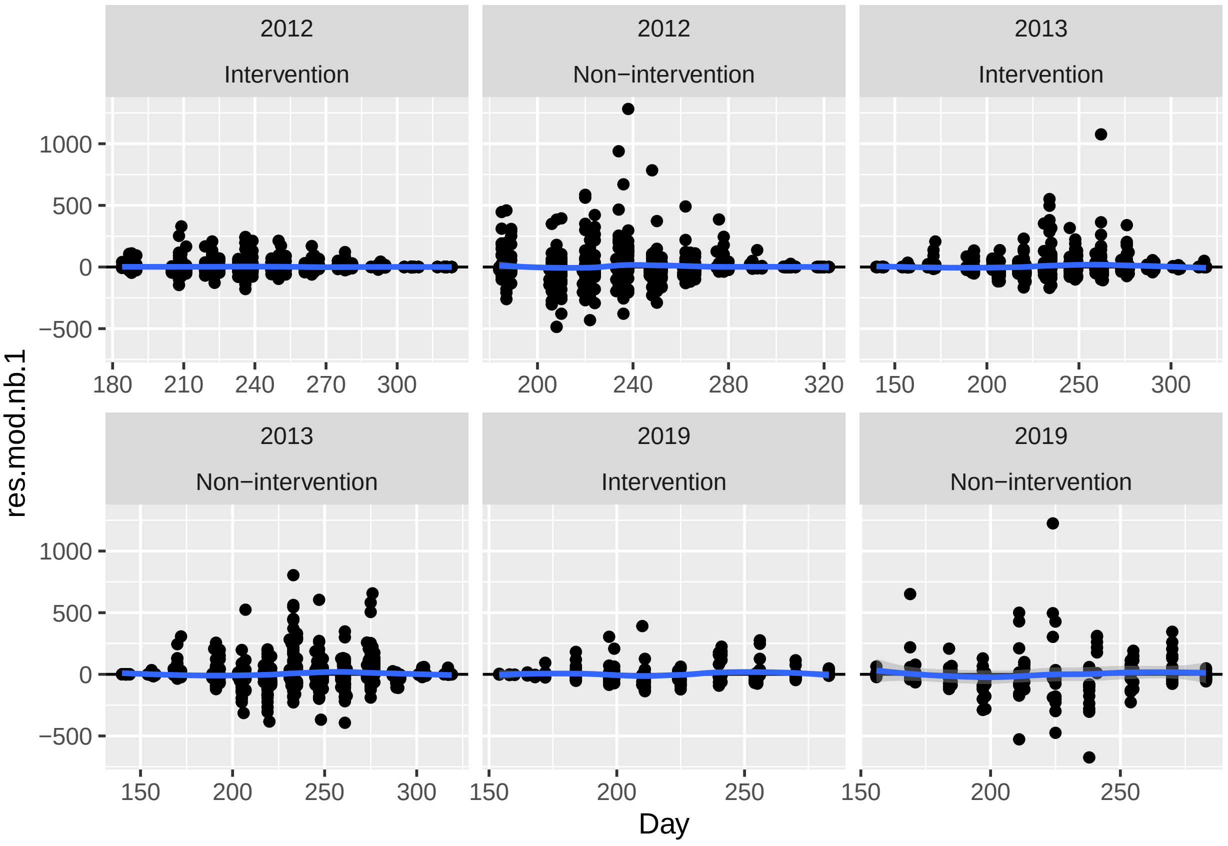

The model assumptions seem to be fulfilled. The approximation of the non-linearity through a quadratic polynomials worked well.

5.4.2 Checking the structure of the random effects

Let's now check at trap AND municipality level whether assuming a simple random intercept is enough to model these short time series. In other words, we are graphically testing whether time correlation is present.

Note, that for eggs and adults 2019 we did not check the structure of the random effect municipality because its estimated variance was close to zero. Here, the estimated variability of municipality is close to the one of traps.

Note also, that ideally we would use the "working residuals" (i.e. the unstandardised residuals in the linear predictor space) to carry such an analysis. Unfortunately, the *residuals.glmmTMB()* method does not implement this type of residuals (yet).

5.4.2.1 Municipality We plot the residuals against *Day* and panel for *MUNICIPALITY*. In each panel, traps are highlighted via solid lines connecting points. Colors highlight different years.

```
d.suter.plus$Year..Trap <- interaction(d.suter.plus$Year,
                                       d.suter.plus$TRAP.ID.fac,
                                       drop = TRUE)
##
ggplot(data = d.suter.plus,
       mapping = aes(y = res.mod.nb.1,
                     x = Day,
                     group = Year..Trap,
                     colour = Year)) +
  geom_hline(yintercept = 0) +
  geom_line(alpha = 0.5) +
  geom_point(alpha = 0.5, size = 0.1) +
  facet_wrap(. ~ MUNICIPALITY, scales = "free_y")
```

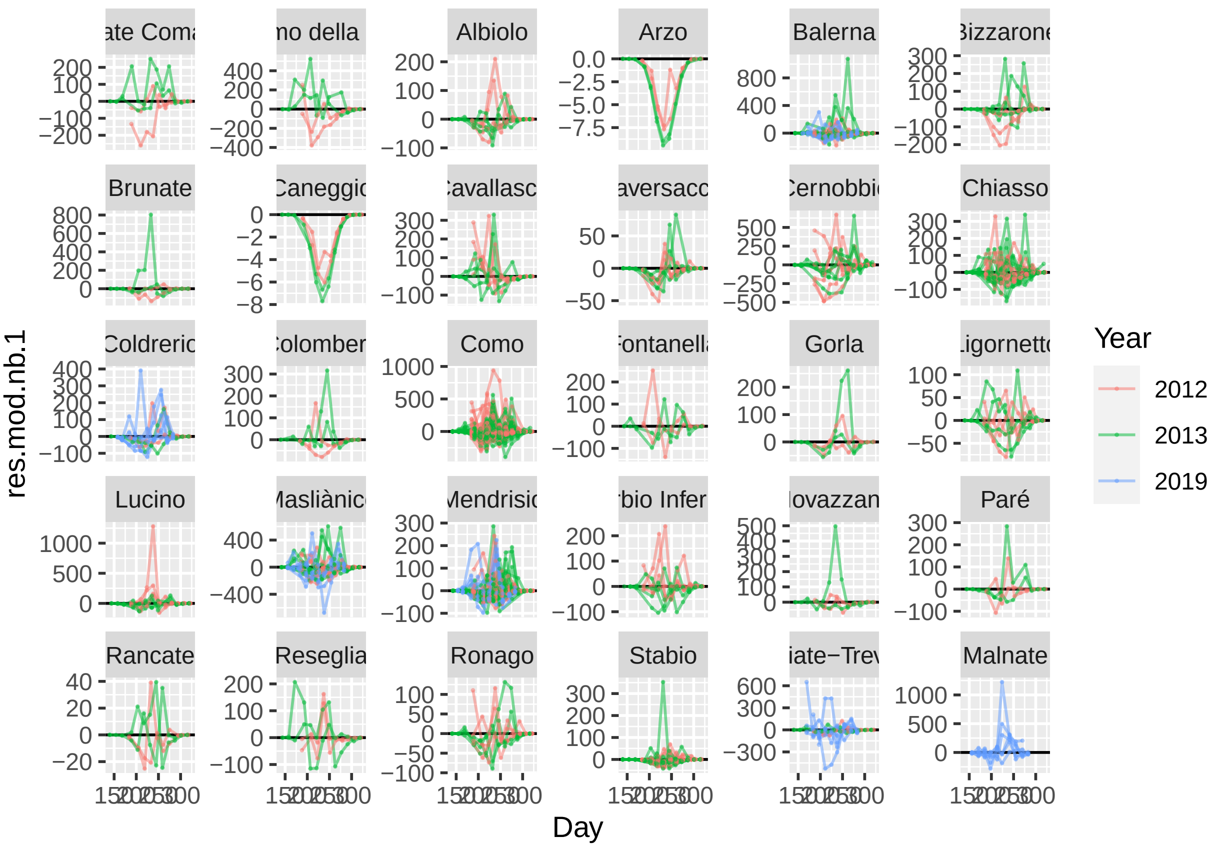

There is no temporal structure left in almost all municipalities. Only in “Arzo” and “Caneggio” there is some clear structure left.

Let’s inspect the observed values along with the predicted values for these two sites.

```
ggplot(data = d.suter.plus %>%
       filter(MUNICIPALITY %in%
                  c("Arzo", "Caneggio")),
       mapping = aes(y = N.ALBOPICTUS,
                     x = Day,
                     group = Year..Trap)) +
  geom_line(alpha  = 0.5) +
  facet_grid(MUNICIPALITY ~ Year) +
  geom_line(mapping = aes(y = fit.mod.nb.1),
            colour = "red",
            alpha = 0.5)
```

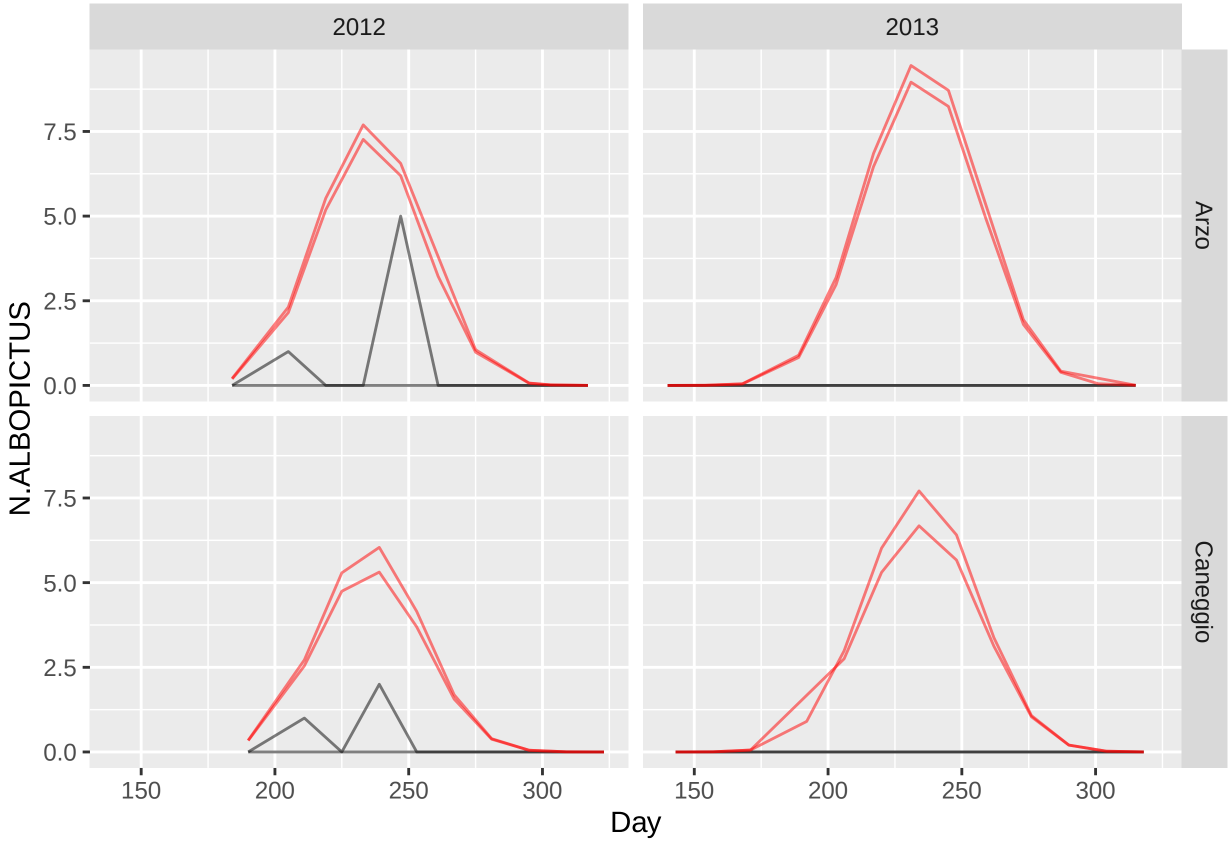

This makes sense. These two places have essentially zero counts all the time. This explains the structure left in the residuals.

We expect the random intercept for these two municipalities for the zero-inflation part to be very high. Let's inspect that:

```
d.ranef.mod.nb.1 <- ranef(mod.nb.1) %>%
  as.data.frame() %>%
  filter(grpvar == "MUNICIPALITY") %>%
  filter(component == "zi") %>%
  select(grp, condval, condsd) %>%
  remove_rownames() %>%
  arrange(desc(condval))
##
head(d.ranef.mod.nb.1, n = 10)
```

```
          grp condval condsd
1        Arzo    1.51   0.72
2    Caneggio    1.40   0.74
3 Caversaccio    1.39   0.50
4   Bizzarone    1.07   0.49
5      Stabio    0.96   0.40
6     Albiolo    0.96   0.48
7     Rancate    0.35   0.55
8        Paré    0.29   0.57
9   Mendrisio    0.28   0.32
```

```
10   Colombera    0.16   0.56
```

Indeed, “Arzo” and “Caneggio” have the largest conditional modes for the zero-inflation part. As *MUNICIPALITY* is take as a random effect, this behaviour is expected (shrinkage of the random effects).

5.4.3 Checking normality of the random effects

The negative binomial model we fitted assumes a normal distribution of the random effects (in the linear predictor space).

```
par(mfrow = c(2,2))
qqnorm(unlist(ranef(mod.nb.1)$cond$MUNICIPALITY),
       main = "Cond. modes MUNICIPALITY (cond)")
qqline(unlist(ranef(mod.nb.1)$cond$MUNICIPALITY))
##
qqnorm(unlist(ranef(mod.nb.1)$cond$TRAP.ID.fac),
              main = "Cond. modes TRAP.ID.fac (cond)")
qqline(unlist(ranef(mod.nb.1)$cond$TRAP.ID.fac))
##
qqnorm(unlist(ranef(mod.nb.1)$zi$MUNICIPALITY),
       main = "Cond. modes MUNICIPALITY (zi)")
qqline(unlist(ranef(mod.nb.1)$cond$MUNICIPALITY))
##
qqnorm(unlist(ranef(mod.nb.1)$zi$TRAP.ID.fac),
              main = "Cond. modes TRAP.ID.fac (zi)")
qqline(unlist(ranef(mod.nb.1)$cond$TRAP.ID.fac))
```

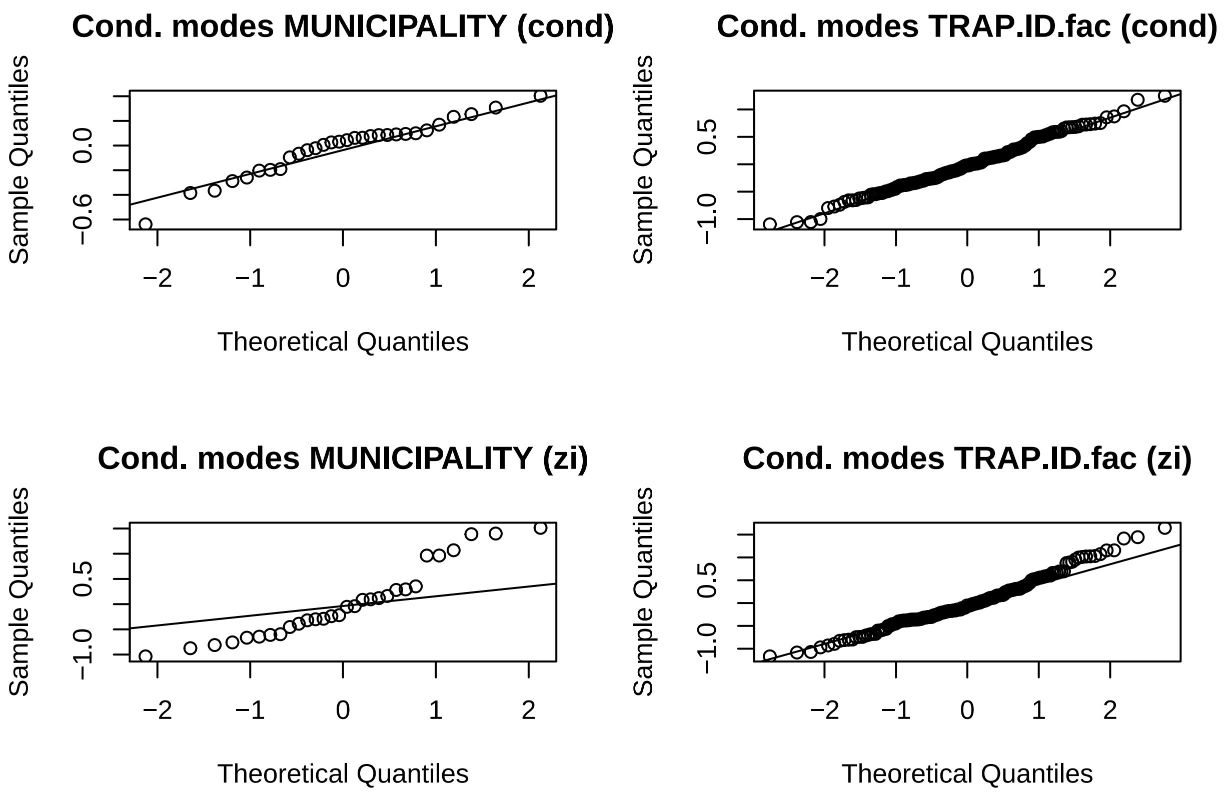

The conditional modes of trap "cond", trap "zi" and municipality "cond" appear to follow a normal distribution. On the other hand, the conditional modes for municipality "zi" appear to be long-tailed. Generalised Mixed-Models are fairly resistant to these deviations.

5.4.4 Checking the mean-variance relationship assumption

The model fitted here assumes that the variance of the observations increases quadratically with the mean value (i.e. family is "truncated_nbinom2"). Let's formally check this.

```
## (warnings are omitted from this chunk)
## (this chunk is cached)
##
mod.nb.1.lin <- update(mod.nb.1, family = "truncated_nbinom1")
##
AIC(mod.nb.1, mod.nb.1.lin)
```

```
             df   AIC
mod.nb.1     43 22980
mod.nb.1.lin 43 23094
```

```
BIC(mod.nb.1, mod.nb.1.lin)
```

```
             df   BIC
mod.nb.1     43 23243
mod.nb.1.lin 43 23357
```

Indeed, both information criteria agree with the current choice of the family.

5.4.5 Checking whether zero-inflation is needed

We first check graphically whether the zero-inflation is present. Note that we are simulating from the fitted model.

```
set.seed(2)
##
xy.1 <- densityplot(~sqrt(simulate(mod.nb.1)),
                   xlim = c(-10, 50),
                   ylim = c(0, 0.2),
                    main = "Simulated from model")
##
xy.2 <- densityplot(~sqrt(d.suter.plus$N.ALBOPICTUS),
                    xlim = c(-10, 50),
                    ylim = c(0, 0.2),
                    main = "Observed data")
##
plot(xy.1, split = c(1,1, 2,1))
plot(xy.2, split = c(2,1, 2,1), newpage = FALSE)
```

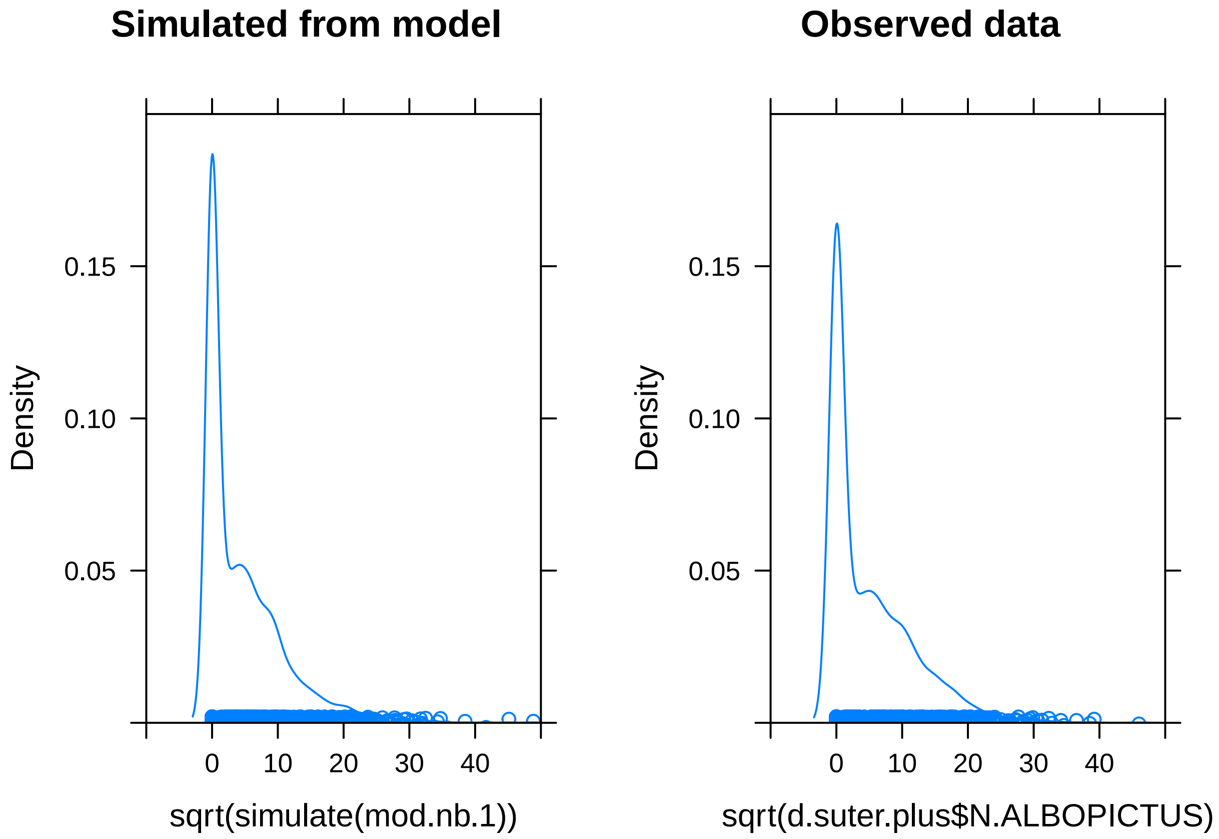

The densities of the two plots look similar. There may be a slight excess of zeros in the simulated part.

Let's now fit the models with no zero-inflation and compare the information criteria.

```
## (warnings are omitted from this chunk)
## (this chunk is cached and depedens on the mod.nb.1 object)
##
mod.nb.1.notZI.linear <- update(mod.nb.1,
                                family = "nbinom1",
                                ziformula = NULL)
mod.nb.1.notZI.quadratic <- update(mod.nb.1,
                                   family = "nbinom2",
                                   ziformula = NULL)
##
AIC(mod.nb.1, mod.nb.1.notZI.linear, mod.nb.1.notZI.quadratic)
```

```
                         df   AIC
mod.nb.1                 43 22980
mod.nb.1.notZI.linear    22 23278
mod.nb.1.notZI.quadratic 22 24250
```

```
BIC(mod.nb.1, mod.nb.1.notZI.linear, mod.nb.1.notZI.quadratic)
```

```
                         df   BIC
mod.nb.1                 43 23243
mod.nb.1.notZI.linear    22 23413
mod.nb.1.notZI.quadratic 22 24384
```

Both information criteria agree that zero-inflation improves the model.

5.4.6 Graphically evaluating the goodness-of-fit

Observed values and fitted values are correlated to quantify the goodness of fit. This is the graphical equivalent of an R^2. Note that fitted values do include the estimated random effects here. Due to the skewness of the data, the graph is produced in the quare-root-transformed space as well.

```
gg.obsPred.NB <- ggplot(data = d.suter.plus,
       mapping = aes(y = N.ALBOPICTUS,
                     x = fit.mod.nb.1)) +
  geom_point(alpha = 0.2) +
  geom_abline(intercept = 0, slope = 1) +
  ggtitle("NB: Obs vs. predicted") +
  coord_fixed()
##
gg.obsPred.NB.Sqrt <- ggplot(data = d.suter.plus,
       mapping = aes(y = sqrt(N.ALBOPICTUS),
                     x = sqrt(fit.mod.nb.1))) +
  geom_point(alpha = 0.2) +
  geom_abline(intercept = 0, slope = 1) +
  ggtitle("NB: Obs vs. predicted -- sqrt()") +
  coord_fixed()
##
grid.arrange(gg.obsPred.NB, gg.obsPred.NB.Sqrt, ncol = 2)
```

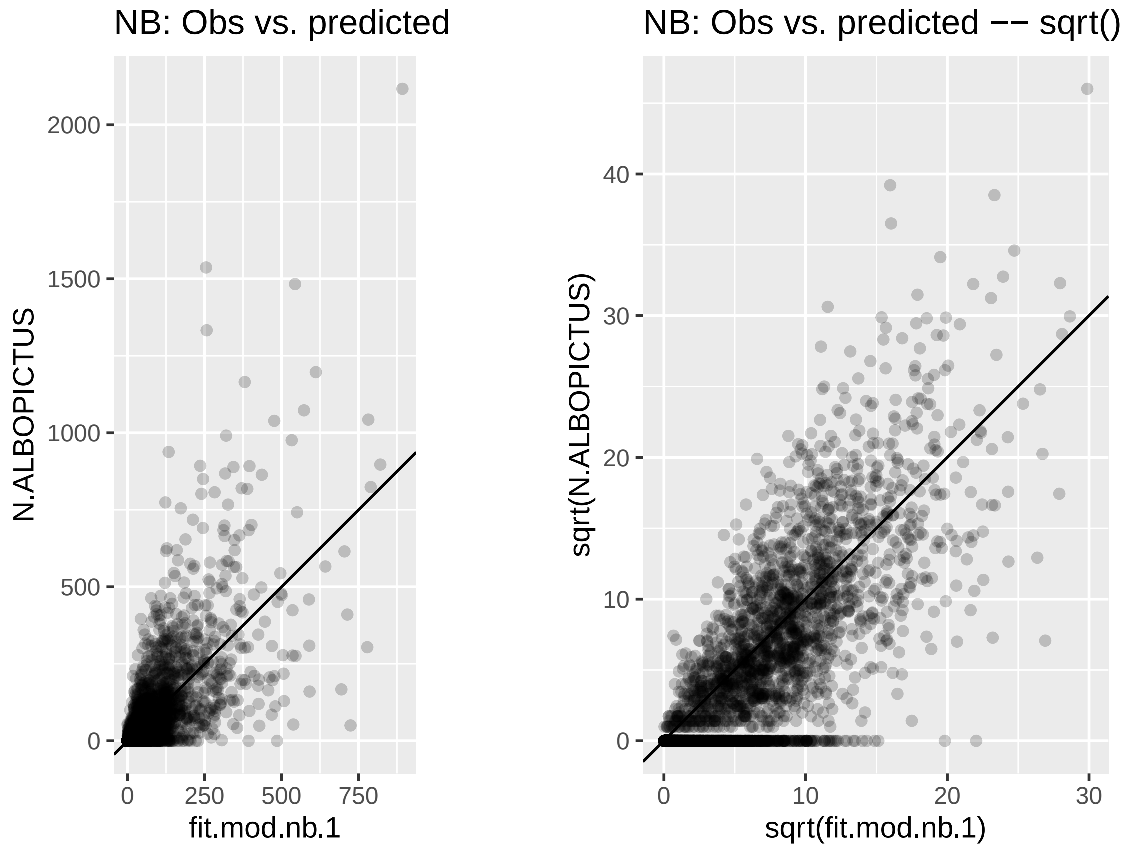

The model fit looks reasonable.

6 Conclusions

For this dataset we can conclude that:

- The effect of time is non-linear (approximated here with a quadratic function).
- The effect "Intervention" vs. "Non-intervention" is clearly present and biologically relevant.
- In particular, the seasonal shapes for "Non-intervention" show consistently larger values.
- The absolute difference between "Non-intervention" and "Intervention" seems to have increased from the period 2012-2013 to 2019.
- There are relevant differences among municipalities.
- There are relevant differences among traps.
- presence/absence is regulated at a larger spatial scale (i.e. *MUNICIPALITY* plays a more relevant role).
- abundance is regulated at smaller spatial scale (i.e. *TRAP.ID.fac* play s a more relevant role).

7 Session Information

```
sessionInfo()
```

```
R version 4.0.4 (2021-02-15)
Platform: x86_64-w64-mingw32/x64 (64-bit)
Running under: Windows 10 x64 (build 19041)

Matrix products: default

locale:
[1] LC_COLLATE=English_Switzerland.1252  LC_CTYPE=English_Switzerland.1252
[3] LC_MONETARY=English_Switzerland.1252 LC_NUMERIC=C
[5] LC_TIME=English_Switzerland.1252

attached base packages:
[1] stats     graphics  grDevices utils     datasets  methods   base

other attached packages:
 [1] tibble_3.0.4      tidyr_1.1.2       gridExtra_2.3     lubridate_1.7.9.2
 [5] glmmTMB_1.0.2.1   ggplot2_3.3.3     lattice_0.20-41   dplyr_1.0.2
 [9] checkpoint_0.4.10 knitr_1.31

loaded via a namespace (and not attached):
 [1] Rcpp_1.0.5        nloptr_1.2.2.2    pillar_1.4.7      compiler_4.0.4
 [5] TMB_1.7.19        tools_4.0.4       boot_1.3-26       digest_0.6.27
 [9] lme4_1.1-26       statmod_1.4.35    nlme_3.1-152      evaluate_0.14
[13] lifecycle_0.2.0   gtable_0.3.0      mgcv_1.8-33       pkgconfig_2.0.3
[17] rlang_0.4.10      Matrix_1.3-2      cli_2.2.0         yaml_2.2.1
[21] xfun_0.22         withr_2.3.0       stringr_1.4.0     generics_0.1.0
[25] vctrs_0.3.6       grid_4.0.4        tidyselect_1.1.0  glue_1.4.2
[29] R6_2.5.0          fansi_0.4.1       rmarkdown_2.7     minqa_1.2.4
[33] farver_2.0.3      purrr_0.3.4       magrittr_2.0.1    scales_1.1.1
[37] ellipsis_0.3.1    htmltools_0.5.1.1 splines_4.0.4     MASS_7.3-53
[41] assertthat_0.2.1  colorspace_2.0-0  labeling_0.4.2    utf8_1.1.4
[45] stringi_1.5.3     munsell_0.5.0     crayon_1.3.4
```
