# Supplementary material for: Effectiveness of integrated Aedes albopictus management in southern Switzerland
Source: Parasit Vectors. 2021 Aug 16;14:405. doi: 10.1186/s13071-021-04903-2 (PMC8365973; doi:10.1186/s13071-021-04903-2)
Supplement: Supplementary file 14 — Additional file 14: Text S7. This pdf file documents additional data analyses of the number of Aedes albopictus eggs collected in 2019 performed at a reviewer’s request. This pdf was created with Rmarkdown and allows for the full reproducibility of the analysis. [file 13071_2021_4903_MOESM14_ESM.pdf]

Additional file 14: Additional Data Analyses Nr. eggs 2019

Dr. Matteo Tanadini | Zurich Data Scientists (ZDS)

May 26, 2021

Contents

1 Reproducibility **2**

2 Loading packages **2**

3 Getting data **2**

4 Graphical Analysis **4**
4.1 MUNICIPALITY 4
4.2 Date.when.ovitrap.collected 4
4.3 Spatial structure 5

5 "Original" Generalised Mixed-Effects Model **7**

6 Generalised Mixed-Effects Models with correlated Random Effects **8**
6.1 Estimating spatial correlation 8
6.2 Including spatial correlation among traps in GLMM models 13
6.3 "Matern" method (trap) 13
6.4 "Gaussian" method (trap) 18
6.5 "Exponential" method (trap) 18
6.6 Including spatial correlation among traps and Municipalities in GLMM models 22
6.7 "Matern" method (municipality and trap) 23
6.8 "Gaussian" method (municipality and trap) 28
6.9 "Exponential" method (municipality and trap) 28

7 Dropping municipality from the random effects **33**

8 Municipality as a fixed effect **35**

9 Bayesian GLMM **37**

10 Session Information **41**

1 Reproducibility

In order to make the analysis fully reproducible, we "freeze" package versions using the *{checkpoint}* package. In particular, we use all packages versions available on CRAN on first of May 2021.

```
## (in this chunk messages are omitted)
##
library(checkpoint)
checkpoint("2021-05-01",
           checkpointLocation = "../")
```

2 Loading packages

We load all add-on packages used in this analysis.

```
## (messages are omitted from this chunk)
##
library(dplyr)
library(lattice)
library(ggplot2)
library(glmmTMB)
library(lubridate)
library(gridExtra)
library(sp)
library(geoR)
library(multcomp)
library(rstanarm)
```

3 Getting data

Note that to ensure reproducibility, the platform-agnostic file type "RDS" is used. Nevertheless, the corresponding "csv" file is also provided.

```
d.eggs.2019 <- readRDS("../0_Data_Preparation_For_CH_vs_I/Created_Datasets/InterventionPaper_eggs.RDS")
##
str(d.eggs.2019)
```

```
tibble [327 x 14] (S3: tbl_df/tbl/data.frame)
 $ WGS84.LAT                  : num [1:327] 45.8 45.8 45.8 45.8 45.8 ...
 $ WGS84.LNG                  : num [1:327] 9 9 9 9 9 ...
 $ ALTITUDE                   : num [1:327] 263 263 263 263 263 263 263 263 316 316 ...
 $ AREA                       : Factor w/ 2 levels "Intervention",..: 1 1 1 1 1 1 1 1 1 1 ...
 $ MUNICIPALITY               : Factor w/ 6 levels "Balerna","Coldrerio",..: 1 1 1 1 1 1 1 1 1 1 ...
 $ Date.when.ovitrap.installed: POSIXct[1:327], format: "2019-05-23" "2019-06-09" ...
 $ Date.when.ovitrap.collected: POSIXct[1:327], format: "2019-06-09" "2019-06-21" ...
 $ No..Days.ovitrap.in.field  : num [1:327] 17 12 14 14 15 14 16 13 12 13 ...
 $ Week.when.ovitrap.collected: num [1:327] 23 25 31 33 35 37 39 41 25 29 ...
 $ No..eggs.AEDES             : num [1:327] 0 29 0 52 202 34 0 57 0 407 ...
 $ No..Eggs.AEDES.in.14.days  : num [1:327] 0 33.8 0 52 188.5 ...
 $ TRAP.ID.fac                : Factor w/ 36 levels "BAL-11a","BAL-2b",..: 1 1 1 1 1 1 1 1 2 2 ...
 $ Day.ovitrap.collected      : num [1:327] 160 172 211 225 240 254 270 283 172 197 ...
 $ no.eggs.normalised.14.days : num [1:327] 0 33.8 0 52 188.5 ...
```

```
print(d.eggs.2019, n = 5, width = Inf)
```

```
# A tibble: 327 x 14
  WGS84.LAT WGS84.LNG ALTITUDE AREA         MUNICIPALITY
      <dbl>     <dbl>    <dbl> <fct>        <fct>
1      45.8      9.00      263 Intervention Balerna
2      45.8      9.00      263 Intervention Balerna
3      45.8      9.00      263 Intervention Balerna
4      45.8      9.00      263 Intervention Balerna
5      45.8      9.00      263 Intervention Balerna
  Date.when.ovitrap.installed Date.when.ovitrap.collected
  <dttm>                      <dttm>
1 2019-05-23 00:00:00         2019-06-09 00:00:00
2 2019-06-09 00:00:00         2019-06-21 00:00:00
3 2019-07-16 00:00:00         2019-07-30 00:00:00
4 2019-07-30 00:00:00         2019-08-13 00:00:00
5 2019-08-13 00:00:00         2019-08-28 00:00:00
  No..Days.ovitrap.in.field Week.when.ovitrap.collected No..eggs.AEDES
                      <dbl>                       <dbl>          <dbl>
1                        17                          23              0
2                        12                          25             29
3                        14                          31              0
4                        14                          33             52
5                        15                          35            202
  No..Eggs.AEDES.in.14.days TRAP.ID.fac Day.ovitrap.collected
                      <dbl> <fct>                       <dbl>
1                       0   BAL-11a                       160
2                      33.8 BAL-11a                       172
3                       0   BAL-11a                       211
4                      52   BAL-11a                       225
5                     189.  BAL-11a                       240
  no.eggs.normalised.14.days
                       <dbl>
1                        0
2                       33.8
3                        0
4                       52
5                      189.
# ... with 322 more rows
```

4 Graphical Analysis

We don't reproduce all plots, but only the relevant ones for this analysis.

4.1 MUNICIPALITY

```
ggplot(data = d.eggs.2019,
                   mapping = aes(y = No..eggs.AEDES)) +
  scale_y_sqrt() +
  geom_hline(yintercept = 0) +
  geom_boxplot(mapping = aes(x = MUNICIPALITY,
                             colour = AREA))
```

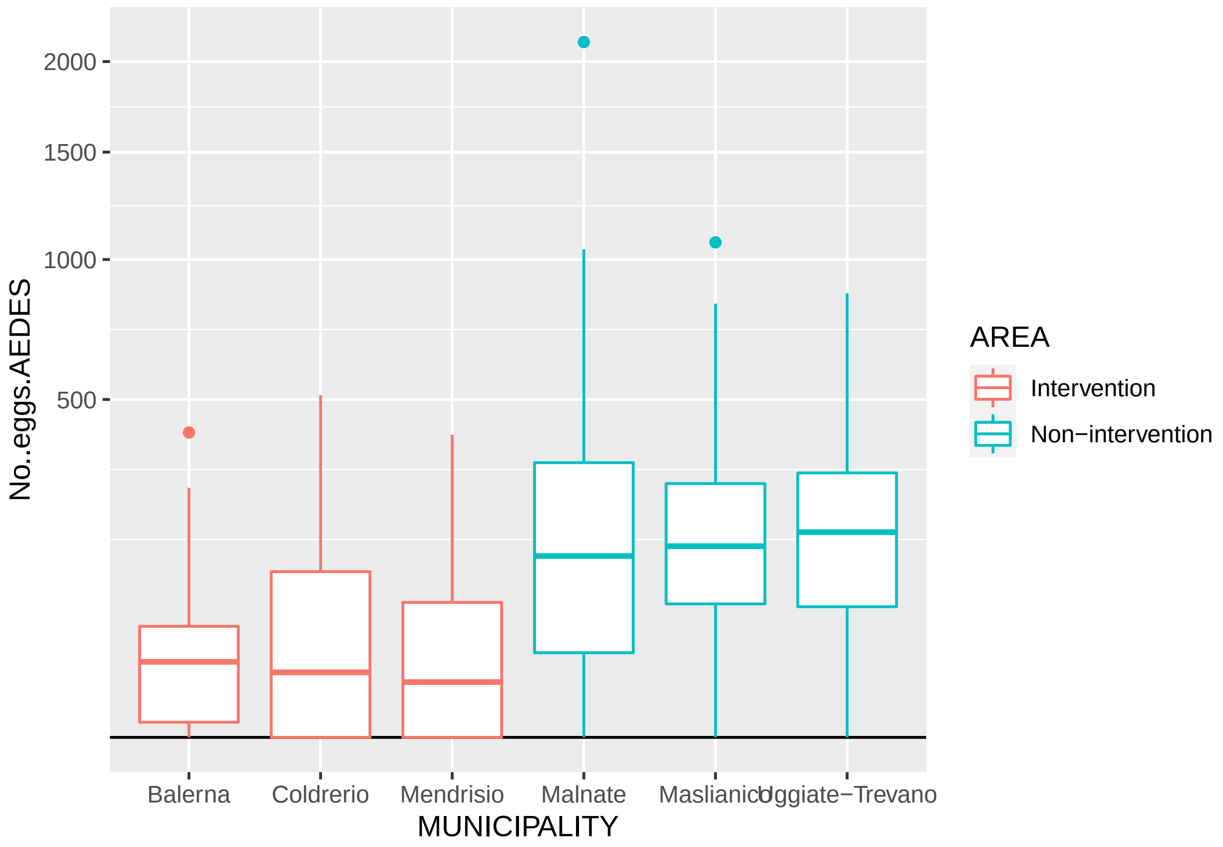

This graph highlights that there almost no variation in medians among municipalities in the same AREA.

4.2 Date.when.ovitrap.collected

Let's include the time component in these graphs. Here we visualise the counts for each trap over time (as a solid line). Note that panelling is used to differentiate among *AREA* levels. Indeed, as *AREA* is the predictor of main interest in this analysis, we may want to include its interactions (when needed) in the model. A smoother is added in both panels to highlight the "shared" time trend within both groups. Note that the y-axis is square-root transformed.

```
## (messages are omitted from this chunk)
##
```

```
ggplot(data = d.eggs.2019,
       mapping = aes(y = No..eggs.AEDES,
                     x = Date.when.ovitrap.collected,
                     group = TRAP.ID.fac)) +
  scale_y_sqrt(breaks = c(0,50,100,200,500,1000,2000),
               minor_breaks = FALSE) +
  geom_hline(yintercept = 0) +
  geom_line(alpha = 0.4) +
  facet_wrap(~AREA) +
  geom_smooth(mapping = aes(group = 1), colour = "#3366FF")
```

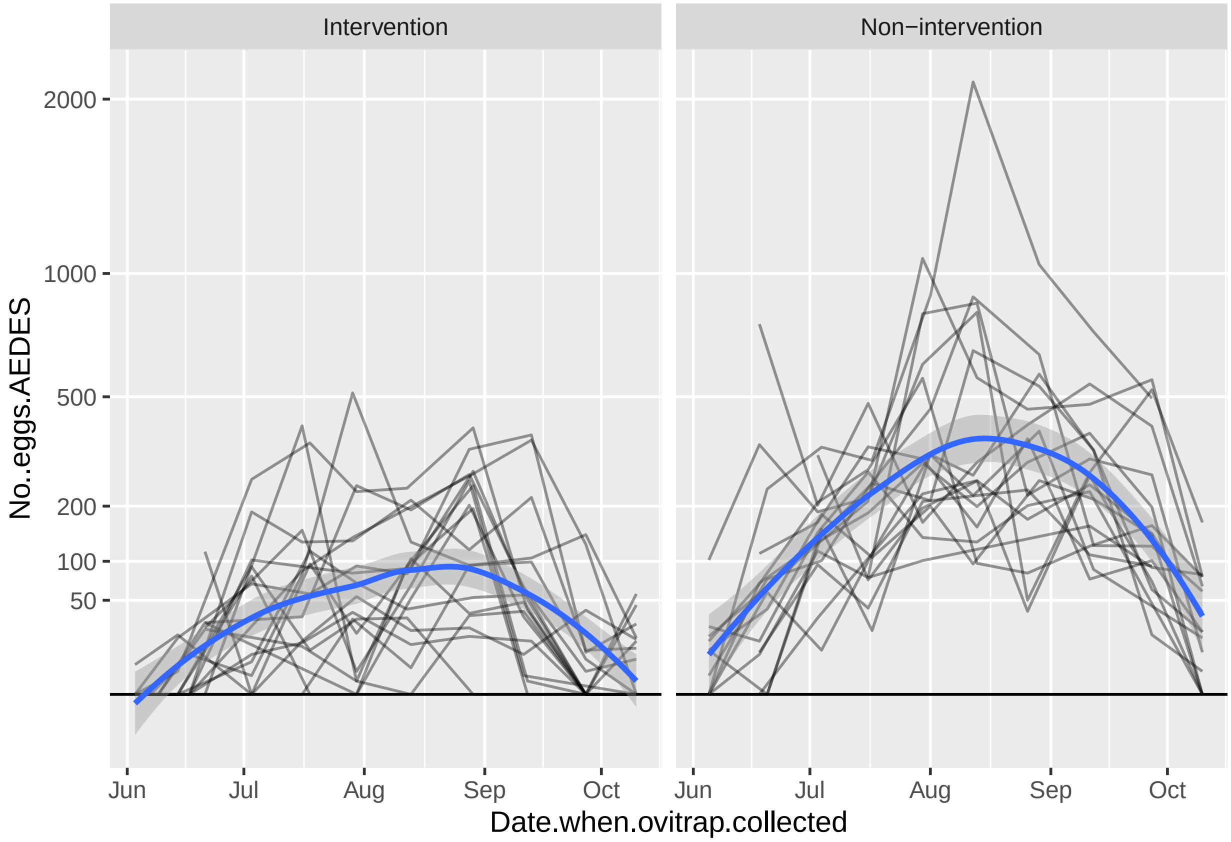

There is a clear bow-shaped seasonal pattern in both groups. This pattern is then modelled as a quadratic effect. Considering that we are fitting a multiplicative model (i.e. link function is the natural logarithm), it is possible that an interaction between *AREA* and *Date* is not needed.

4.3 Spatial structure

Let's visualise the spatial structure of the 36 traps. We use colours to discriminate between "Intervention" and "Non-intervention" sites.

```
plot(WGS84.LAT ~ WGS84.LNG,
     data = d.eggs.2019,
     col = AREA,
     asp = 1)
```

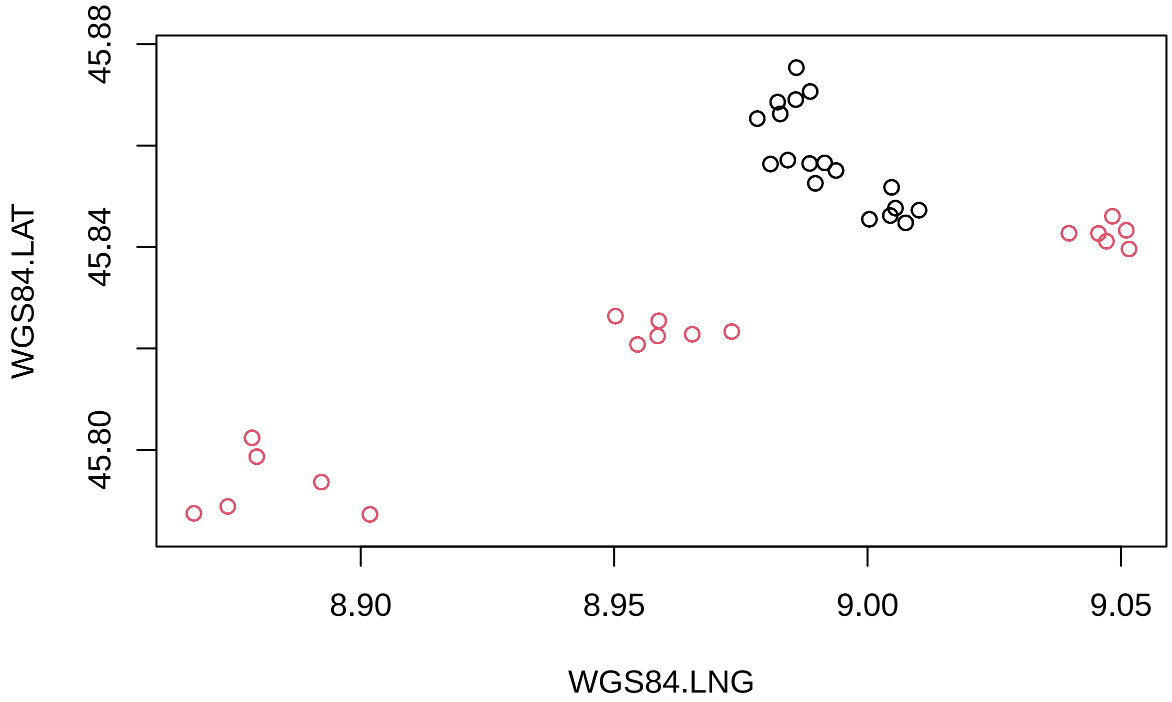

Let's now highlight the six municipalities.

```
xyplot(WGS84.LAT ~ WGS84.LNG,
       data = d.eggs.2019,
       groups = MUNICIPALITY,
       type = c("p", "g"),
       auto.key = list(space = "right"),
       aspect = 1)
```

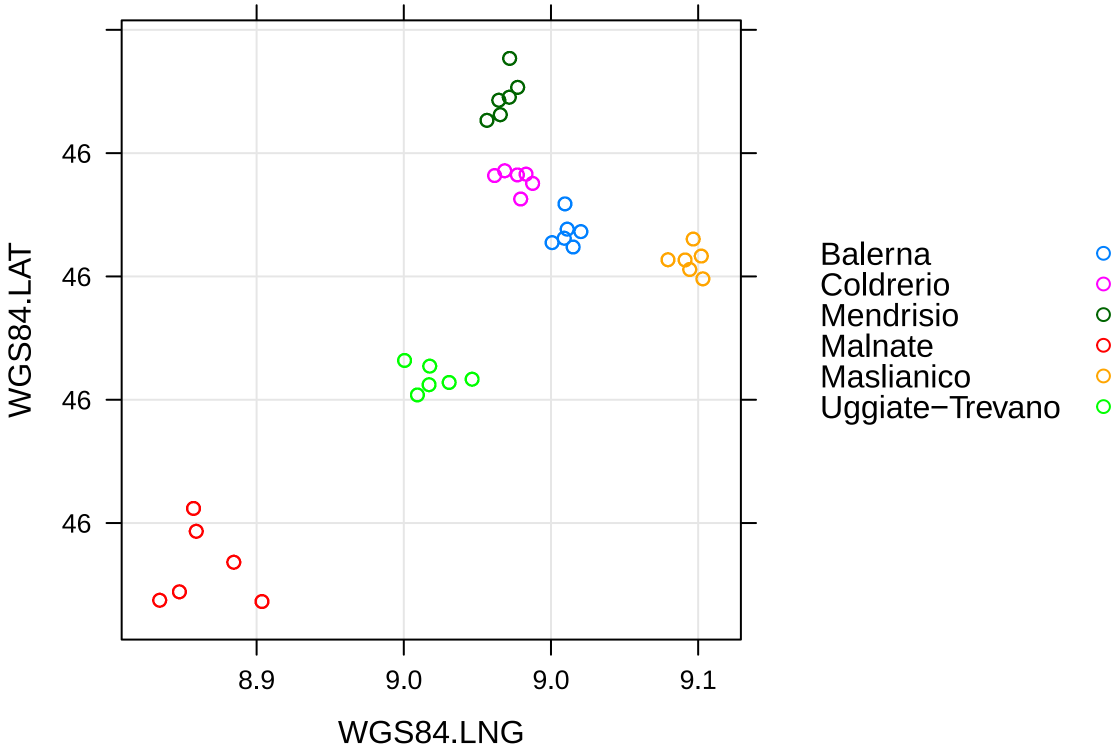

There are six traps in each municipality. Each trap is measured 10 times.

5 "Original" Generalised Mixed-Effects Model

In this section we refit the "original" model fitted in appendix 11.

```
## (warning are omitted from this chunk)
##
mod.nb.1 <- glmmTMB(No..eggs.AEDES ~ AREA +
                      poly(Day.ovitrap.collected, degree = 2) +
                      scale(ALTITUDE) +
                      No..Days.ovitrap.in.field +
                      (1 | TRAP.ID.fac) + (1 | MUNICIPALITY),
                    family = "nbinom1",
                    data = d.eggs.2019)
##
summary(mod.nb.1)
```

```
 Family: nbinom1  ( log )
Formula:
No..eggs.AEDES ~ AREA + poly(Day.ovitrap.collected, degree = 2) +
    scale(ALTITUDE) + No..Days.ovitrap.in.field + (1 | TRAP.ID.fac) +
    (1 | MUNICIPALITY)
Data: d.eggs.2019

     AIC      BIC   logLik deviance df.resid
```

```
    3336     3370    -1659     3318      318

Random effects:

Conditional model:
 Groups       Name        Variance Std.Dev.
 TRAP.ID.fac  (Intercept) 1.76e-01 4.20e-01
 MUNICIPALITY (Intercept) 4.51e-09 6.72e-05
Number of obs: 327, groups:  TRAP.ID.fac, 36; MUNICIPALITY, 6

Overdispersion parameter for nbinom1 family ():  121

Conditional model:
                                         Estimate Std. Error z value Pr(>|z|)
(Intercept)                                2.4370     0.4678    5.21  1.9e-07
AREANon-intervention                       1.3387     0.1723    7.77  7.8e-15
poly(Day.ovitrap.collected, degree = 2)1   3.9889     1.0696    3.73  0.00019
poly(Day.ovitrap.collected, degree = 2)2 -15.5900     1.0701  -14.57  < 2e-16
scale(ALTITUDE)                            0.0415     0.0807    0.51  0.60690
No..Days.ovitrap.in.field                  0.0909     0.0307    2.96  0.00310

(Intercept)                              ***
AREANon-intervention                     ***
poly(Day.ovitrap.collected, degree = 2)1 ***
poly(Day.ovitrap.collected, degree = 2)2 ***
scale(ALTITUDE)
No..Days.ovitrap.in.field                **
---
Signif. codes:  0 '***' 0.001 '**' 0.01 '*' 0.05 '.' 0.1 ' ' 1
```

6 Generalised Mixed-Effects Models with correlated Random Effects

6.1 Estimating spatial correlation

To start with, we estimate how correlated are the random effects estimated in the "original" model.

Here we focus on the trap random effects. The estimation of the spatial correlation at municipality level is not feasible as only 6 observations are present. To be more precise, it is technically possible to estimate spatial correlation at municipality level, however, this estimation will not be reliable.

We first extract the estimated random effects from the original model and put them in a dataframe with their coordinates.

```r
## i) extract RE estimates
d.RE <- ranef(mod.nb.1)$cond$TRAP.ID.fac %>%
  as.data.frame() %>%
  rename("est.int" = `(Intercept)`) %>%
  tibble::rownames_to_column("TRAP.ID.fac")
##
str(d.RE)
```

```
'data.frame':   36 obs. of  2 variables:
 $ TRAP.ID.fac: chr  "BAL-11a" "BAL-2b" "BAL-5b" "BAL-6b" ...
 $ est.int    : num  -0.159881 -0.113879 -0.498255 0.531151 -0.000199 ...
```

```
 - attr(*, "condVar")= num [1, 1, 1:36] 0.0877 0.0795 0.0843 0.0801 0.0835 ...
```

```
##
## ii) join  with coordinates
d.RE.coor <- d.RE %>%
  left_join(unique(d.eggs.2019[ ,c("TRAP.ID.fac", "WGS84.LAT",
                                   "WGS84.LNG",
                                   "MUNICIPALITY")]))
```

```
Joining, by = "TRAP.ID.fac"
```

```
##
head(d.RE.coor)
```

```
  TRAP.ID.fac est.int WGS84.LAT WGS84.LNG MUNICIPALITY
1     BAL-11a -0.1599        46         9      Balerna
2      BAL-2b -0.1139        46         9      Balerna
3      BAL-5b -0.4983        46         9      Balerna
4      BAL-6b  0.5312        46         9      Balerna
5      BAL-8b -0.0002        46         9      Balerna
6      BAL-9a  0.0050        46         9      Balerna
```

```
str(d.RE.coor)
```

```
'data.frame':   36 obs. of  5 variables:
 $ TRAP.ID.fac : chr  "BAL-11a" "BAL-2b" "BAL-5b" "BAL-6b" ...
 $ est.int     : num  -0.159881 -0.113879 -0.498255 0.531151 -0.000199 ...
 $ WGS84.LAT   : num  45.8 45.9 45.8 45.8 45.8 ...
 $ WGS84.LNG   : num  9 9 9.01 9.01 9 ...
 $ MUNICIPALITY: Factor w/ 6 levels "Balerna","Coldrerio",..: 1 1 1 1 1 1 2 2 2 2 ...
 - attr(*, "condVar")= num [1, 1, 1:36] 0.0877 0.0795 0.0843 0.0801 0.0835 ...
```

Let's visualise the estimated random effects over space.

```
ggplot(data = d.RE.coor,
       mapping = aes(y = WGS84.LAT,
                     x = WGS84.LNG,
                     col = est.int)) +
  geom_point() +
  scale_colour_gradientn(colours = c("blue", "gray", "red"),
                         values = c(0, 0.5, 1)) +
  coord_fixed(ratio = 1)
```

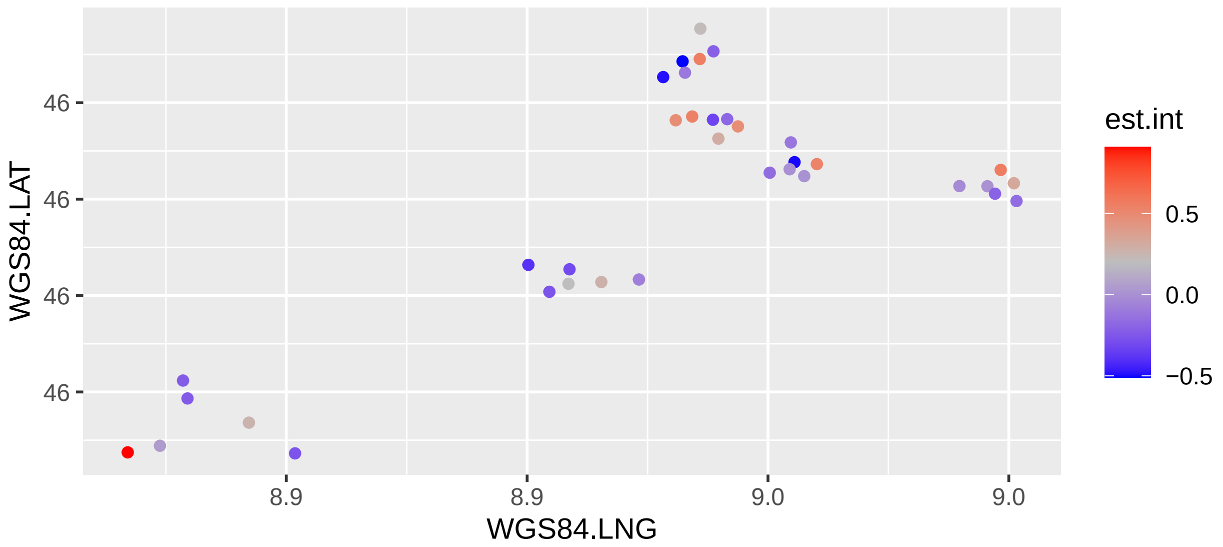

This graph, does not highlight any spatial correlation among traps. (Note that the colours are mapped (internally) to the 0-1 range).

Let's reproduce a similar graph with bubbleplots (package {sp}).

```
d.RE.coor.Sp <- d.RE.coor
coordinates(d.RE.coor.Sp) <- c("WGS84.LNG", "WGS84.LAT")
## str(d.coo.3)
bubble(d.RE.coor.Sp, zcol = "est.int", aspect = 1)
```

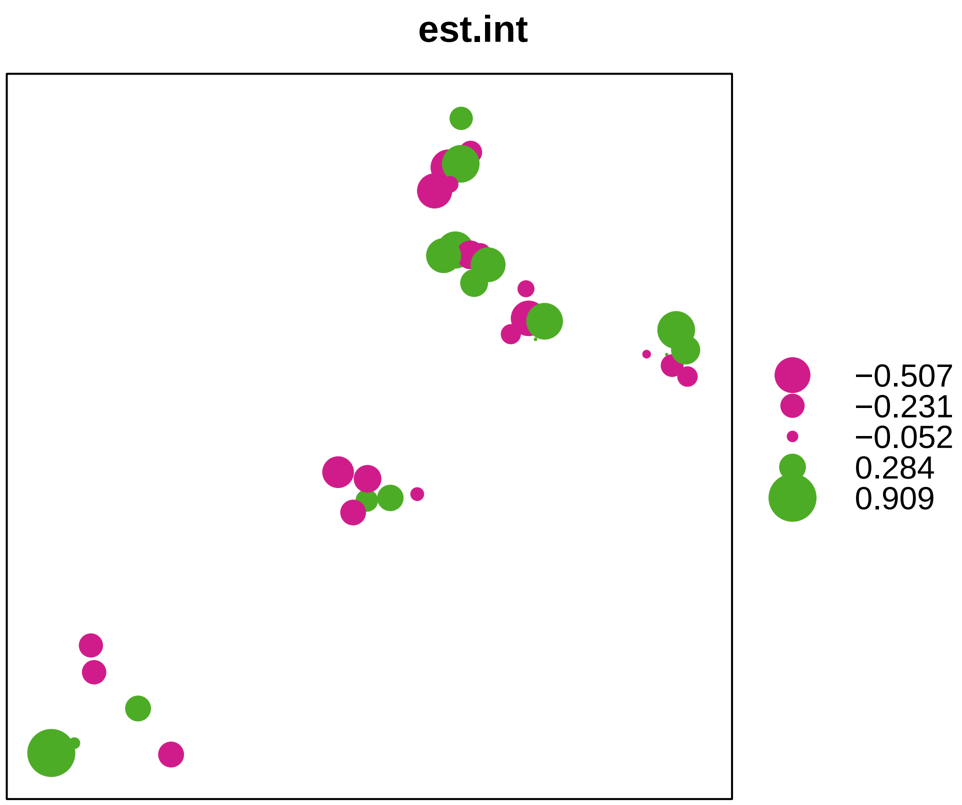

This graph, as expected, does not highlight any spatial correlation among traps either.

Let's formally estimate the spatial correlation among traps. We first compute a variogram at default distances.

```
plot(variog(data = d.RE.coor$est.int,
            coords =
              d.RE.coor[ , c("WGS84.LNG",
                             "WGS84.LAT")]))
```

```
variog: computing omnidirectional variogram
```

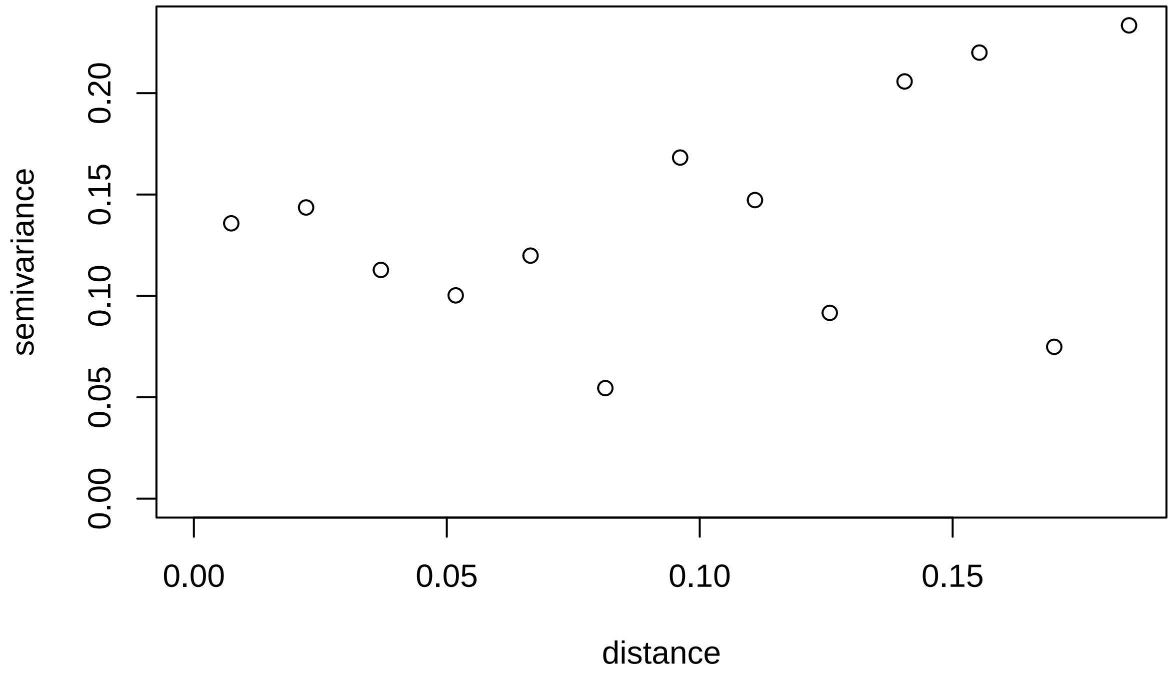

There is no evidence of spatial correlation.

As traps are geographically clustered we adapt the scale at which spatial correlation is estimated such that we can better focus on the “intra-municipality-scale”. To do so, we compute the maximal distance within traps of Malnate, which shows the largest within-municipality scatter among all municipalities.

```
max(dist(x = d.RE.coor[d.RE.coor$MUNICIPALITY == "Malnate",
                       c("WGS84.LAT", "WGS84.LNG")]))
```

```
[1] 0.035
```

The max distance within Malnate is 0.035. This distance covers all distances present in the data within a given municipality.

```
plot(variog(data = d.RE.coor$est.int,
            coords = d.RE.coor[,
                               c("WGS84.LNG",
                                 "WGS84.LAT")],
            max.dist = 0.035))
```

```
variog: computing omnidirectional variogram
```

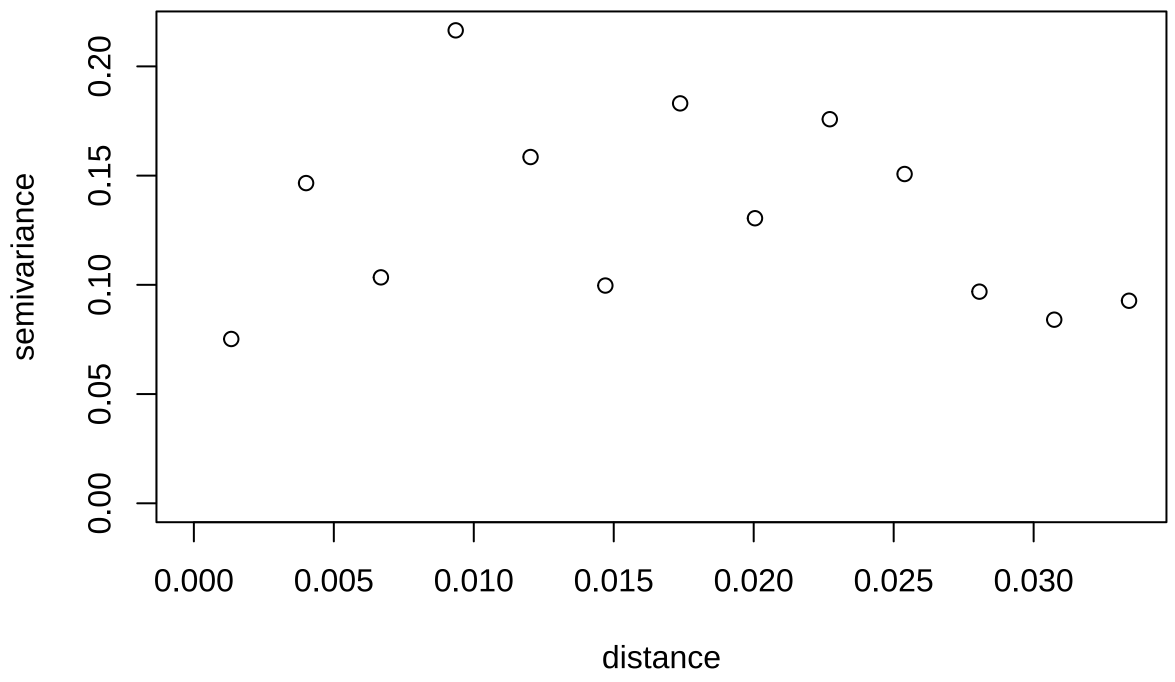

Once again, there is no evidence for the estimated random effect for traps to be correlated in space.

6.2 Including spatial correlation among traps in GLMM models

We first create the coordinates object as required by *glmmTMB()*.

```
d.eggs.2019$pos <- numFactor(d.eggs.2019$WGS84.LNG,
                  d.eggs.2019$WGS84.LAT)
##
# parseNumLevels(levels(d.eggs.2019$pos))
```

As there are three implementations of spatially-autocorrelated random effects, we use all of them. We start with models where only traps can be spatially-autocorrelated. As mentioned before it is not sensible to estimate spatial correlation among the six municipalities.

6.3 "Matern" method (trap)

```
mod.nb.mat <- glmmTMB(No..eggs.AEDES ~ AREA +
                      poly(Day.ovitrap.collected, degree = 2) +
                      scale(ALTITUDE) +
                      No..Days.ovitrap.in.field +
                      ##
                      mat(pos + 0 | TRAP.ID.fac) +
                      ##
                      (1 | MUNICIPALITY),
                  family = "nbinom1",
                  data = d.eggs.2019)
```

```
Warning in Matrix::sparseMatrix(dims = c(0, 0), i = integer(0), j =
integer(0), : 'giveCsparse' has been deprecated; setting 'repr = "T"' for you

Warning in Matrix::sparseMatrix(dims = c(0, 0), i = integer(0), j =
integer(0), : 'giveCsparse' has been deprecated; setting 'repr = "T"' for you

Warning in Matrix::sparseMatrix(dims = c(0, 0), i = integer(0), j =
integer(0), : 'giveCsparse' has been deprecated; setting 'repr = "T"' for you

Warning in fitTMB(TMBStruc): Model convergence problem; non-positive-definite
Hessian matrix. See vignette('troubleshooting')
```

```
##
options(max.print = 2000)
summary(mod.nb.mat)
```

```
 Family: nbinom1  ( log )
Formula:
No..eggs.AEDES ~ AREA + poly(Day.ovitrap.collected, degree = 2) +
    scale(ALTITUDE) + No..Days.ovitrap.in.field + mat(pos + 0 |
    TRAP.ID.fac) + (1 | MUNICIPALITY)
Data: d.eggs.2019

     AIC      BIC   logLik deviance df.resid
      NA       NA       NA       NA      316

Random effects:

Conditional model:
 Groups       Name                      Variance Std.Dev. Corr
 TRAP.ID.fac  pos(8.901817,45.787266) 1.76e-01 4.20e-01
              pos(8.867073,45.787479) 1.76e-01 4.20e-01 1.00
              pos(8.873767,45.788844) 1.76e-01 4.20e-01 1.00 1.00
              pos(8.892238,45.793641) 1.76e-01 4.20e-01 1.00 1.00 1.00
              pos(8.879491,45.798663) 1.76e-01 4.20e-01 1.00 1.00 1.00 1.00
              pos(8.87856,45.802371)  1.76e-01 4.20e-01 1.00 1.00 1.00 1.00
              pos(8.95462,45.82078)   1.76e-01 4.20e-01 0.99 0.99 0.99 0.99
              pos(8.95859,45.82244)   1.76e-01 4.20e-01 0.99 0.99 0.99 0.99
              pos(8.96541,45.8228)    1.76e-01 4.20e-01 0.99 0.98 0.99 0.99
              pos(8.97321,45.82333)   1.76e-01 4.20e-01 0.99 0.98 0.98 0.99
              pos(8.9588,45.82545)    1.76e-01 4.20e-01 0.99 0.99 0.99 0.99
              pos(8.95026,45.82637)   1.76e-01 4.20e-01 0.99 0.99 0.99 0.99
              pos(9.0516,45.83961)    1.76e-01 4.20e-01 0.97 0.96 0.96 0.97
              pos(9.04714,45.84113)   1.76e-01 4.20e-01 0.97 0.96 0.96 0.97
              pos(9.04556,45.84268)   1.76e-01 4.20e-01 0.97 0.96 0.96 0.97
              pos(9.03975,45.84271)   1.76e-01 4.20e-01 0.97 0.96 0.96 0.97
              pos(9.05106,45.8433)    1.76e-01 4.20e-01 0.97 0.96 0.96 0.97
              pos(9.00753,45.84477)   1.76e-01 4.20e-01 0.98 0.97 0.97 0.98
              pos(9.00037,45.84548)   1.76e-01 4.20e-01 0.98 0.97 0.97 0.98
              pos(9.04832,45.84606)   1.76e-01 4.20e-01 0.97 0.96 0.96 0.97
              pos(9.0045,45.8462)     1.76e-01 4.20e-01 0.98 0.97 0.97 0.98
              pos(9.01016,45.84727)   1.76e-01 4.20e-01 0.98 0.97 0.97 0.98
              pos(9.00551,45.84767)   1.76e-01 4.20e-01 0.98 0.97 0.97 0.98
              pos(9.00475,45.85177)   1.76e-01 4.20e-01 0.98 0.97 0.97 0.98
```

```
             pos(8.98971,45.85256)   1.76e-01 4.20e-01 0.98 0.97 0.98 0.98
             pos(8.99377,45.85509)   1.76e-01 4.20e-01 0.98 0.97 0.98 0.98
             pos(8.98085,45.85636)   1.76e-01 4.20e-01 0.98 0.98 0.98 0.98
             pos(8.98858,45.85647)   1.76e-01 4.20e-01 0.98 0.97 0.98 0.98
             pos(8.99154,45.85659)   1.76e-01 4.20e-01 0.98 0.97 0.98 0.98
             pos(8.98427,45.85714)   1.76e-01 4.20e-01 0.98 0.98 0.98 0.98
             pos(8.97825,45.86532)   1.76e-01 4.20e-01 0.98 0.98 0.98 0.98
             pos(8.98278,45.86623)   1.76e-01 4.20e-01 0.98 0.97 0.98 0.98
             pos(8.98227,45.86858)   1.76e-01 4.20e-01 0.98 0.97 0.98 0.98
             pos(8.98584,45.86907)   1.76e-01 4.20e-01 0.98 0.97 0.98 0.98
             pos(8.98868,45.87067)   1.76e-01 4.20e-01 0.98 0.97 0.97 0.98
             pos(8.98596,45.87537)   1.76e-01 4.20e-01 0.98 0.97 0.97 0.98
MUNICIPALITY (Intercept)             4.51e-09 6.72e-05
```

```
1.00
0.99 0.99
0.99 0.99 1.00
0.99 0.99 1.00 1.00
0.99 0.99 1.00 1.00 1.00
0.99 0.99 1.00 1.00 1.00 1.00
0.99 0.99 1.00 1.00 1.00 1.00 1.00
0.96 0.96 0.99 0.99 0.99 0.99 0.99 0.98
0.96 0.96 0.99 0.99 0.99 0.99 0.99 0.99 1.00
0.96 0.96 0.99 0.99 0.99 0.99 0.99 0.99 1.00 1.00
0.97 0.97 0.99 0.99 0.99 0.99 0.99 0.99 1.00 1.00 1.00
0.96 0.96 0.99 0.99 0.99 0.99 0.99 0.98 1.00 1.00 1.00 1.00
0.98 0.98 0.99 0.99 1.00 1.00 1.00 0.99 1.00 1.00 1.00 1.00 1.00
0.98 0.98 1.00 1.00 1.00 1.00 1.00 0.99 1.00 1.00 1.00 1.00 1.00 1.00
0.96 0.96 0.99 0.99 0.99 0.99 0.99 0.99 1.00 1.00 1.00 1.00 1.00 1.00 1.00
0.98 0.98 0.99 1.00 1.00 1.00 1.00 0.99 1.00 1.00 1.00 1.00 1.00 1.00 1.00
0.97 0.97 0.99 0.99 1.00 1.00 0.99 0.99 1.00 1.00 1.00 1.00 1.00 1.00 1.00
0.98 0.98 0.99 0.99 1.00 1.00 1.00 0.99 1.00 1.00 1.00 1.00 1.00 1.00 1.00
0.98 0.98 0.99 0.99 1.00 1.00 1.00 0.99 1.00 1.00 1.00 1.00 1.00 1.00 1.00
0.98 0.98 1.00 1.00 1.00 1.00 1.00 1.00 0.99 0.99 0.99 1.00 0.99 1.00 1.00
0.98 0.98 1.00 1.00 1.00 1.00 1.00 1.00 0.99 0.99 0.99 1.00 0.99 1.00 1.00
0.98 0.98 1.00 1.00 1.00 1.00 1.00 1.00 0.99 0.99 0.99 0.99 0.99 1.00 1.00
0.98 0.98 1.00 1.00 1.00 1.00 1.00 1.00 0.99 0.99 0.99 1.00 0.99 1.00 1.00
0.98 0.98 1.00 1.00 1.00 1.00 1.00 1.00 0.99 0.99 0.99 1.00 0.99 1.00 1.00
0.98 0.98 1.00 1.00 1.00 1.00 1.00 1.00 0.99 0.99 0.99 0.99 0.99 1.00 1.00
0.98 0.98 1.00 1.00 1.00 1.00 1.00 1.00 0.99 0.99 0.99 0.99 0.99 1.00 1.00
0.98 0.98 0.99 1.00 1.00 1.00 1.00 1.00 0.99 0.99 0.99 0.99 0.99 1.00 1.00
0.98 0.98 0.99 1.00 1.00 1.00 1.00 1.00 0.99 0.99 0.99 0.99 0.99 1.00 1.00
0.98 0.98 0.99 0.99 1.00 1.00 1.00 0.99 0.99 0.99 0.99 0.99 0.99 1.00 1.00
0.98 0.98 0.99 0.99 0.99 1.00 0.99 0.99 0.99 0.99 0.99 0.99 0.99 1.00 1.00
0.98 0.98 0.99 0.99 0.99 0.99 0.99 0.99 0.99 0.99 0.99 0.99 0.99 1.00 1.00
```

1.00														
1.00	1.00													
1.00	1.00	1.00												
1.00	1.00	1.00	1.00											
0.99	1.00	1.00	1.00	1.00										
0.99	1.00	1.00	1.00	1.00	1.00									
0.99	1.00	1.00	1.00	1.00	1.00	1.00								
0.99	1.00	1.00	1.00	1.00	1.00	1.00	1.00							
0.99	1.00	1.00	1.00	1.00	1.00	1.00	1.00	1.00						
0.99	1.00	1.00	1.00	1.00	1.00	1.00	1.00	1.00	1.00					
0.99	1.00	1.00	1.00	1.00	1.00	1.00	1.00	1.00	1.00	1.00				
0.99	1.00	1.00	1.00	1.00	1.00	1.00	1.00	1.00	1.00	1.00	1.00			
0.99	1.00	1.00	1.00	1.00	1.00	1.00	1.00	1.00	1.00	1.00	1.00	1.00		
0.99	1.00	1.00	1.00	1.00	1.00	1.00	1.00	1.00	1.00	1.00	1.00	1.00	1.00	
0.99	1.00	1.00	1.00	1.00	1.00	1.00	1.00	1.00	1.00	1.00	1.00	1.00	1.00	1.00
0.99	1.00	1.00	1.00	1.00	1.00	1.00	1.00	1.00	1.00	1.00	1.00	1.00	1.00	1.00

```
 1.00

Number of obs: 327, groups:  TRAP.ID.fac, 36; MUNICIPALITY, 6

Overdispersion parameter for nbinom1 family ():  121

Conditional model:
                                        Estimate Std. Error z value Pr(>|z|)
(Intercept)                               2.4370     0.4678    5.21  1.9e-07
AREANon-intervention                      1.3387     0.1723    7.77  7.8e-15
poly(Day.ovitrap.collected, degree = 2)1   3.9889     1.0696    3.73  0.00019
poly(Day.ovitrap.collected, degree = 2)2 -15.5900     1.0701  -14.57  < 2e-16
scale(ALTITUDE)                           0.0415     0.0807    0.51  0.60690
No..Days.ovitrap.in.field                 0.0909     0.0307    2.96  0.00310

(Intercept)                              ***
AREANon-intervention                     ***
poly(Day.ovitrap.collected, degree = 2)1 ***
poly(Day.ovitrap.collected, degree = 2)2 ***
scale(ALTITUDE)
No..Days.ovitrap.in.field                **
---
Signif. codes:  0 '***' 0.001 '**' 0.01 '*' 0.05 '.' 0.1 ' ' 1
```

```
##
mod.nb.mat$sdr$pdHess
```

```
[1] FALSE
```

The model does not converge. As a matter of fact, this is a fairly complicated model to estimate for such a simple design. In particular, trying to estimated spatially correlated random effects in the GLMM framework based on 36 sites is ambitious. Indeed, GLMMs are a fairly complex machinery even when random effects are assumed to be independent.

Surprisingly, all the estimates for the random effects are identical to those of the model with no spatial correlation.

```
options(scipen = 999)
VarCorr(mod.nb.1)
```

```
Conditional model:
 Groups       Name        Std.Dev.
 TRAP.ID.fac  (Intercept) 0.4196126
 MUNICIPALITY (Intercept) 0.0000672
```

```
options(scipen = 0)
```

The inferential results for the *AREA* predictor are also identical to the model where no spatial correlation is included (i.e. the original analysis). This is unexpected. We would have expected at least some small changes in the standard deviations of the estimates.

6.4 "Gaussian" method (trap)

```
mod.nb.gau <- glmmTMB(No..eggs.AEDES ~ AREA +
                        poly(Day.ovitrap.collected, degree = 2) +
                        scale(ALTITUDE) +
                        No..Days.ovitrap.in.field +
                        ##
                        gau(pos + 0 | TRAP.ID.fac) +
                        ##
                        (1 | MUNICIPALITY),
                      family = "nbinom1",
                      data = d.eggs.2019)
```

```
Warning in Matrix::sparseMatrix(dims = c(0, 0), i = integer(0), j =
integer(0), : 'giveCsparse' has been deprecated; setting 'repr = "T"' for you

Warning in Matrix::sparseMatrix(dims = c(0, 0), i = integer(0), j =
integer(0), : 'giveCsparse' has been deprecated; setting 'repr = "T"' for you

Warning in Matrix::sparseMatrix(dims = c(0, 0), i = integer(0), j =
integer(0), : 'giveCsparse' has been deprecated; setting 'repr = "T"' for you

Warning in (function (start, objective, gradient = NULL, hessian = NULL, : NA/
NaN function evaluation

Error in (function (start, objective, gradient = NULL, hessian = NULL, : gradient function must return [illegible]

Timing stopped at: 2.49 0.01 2.5
```

```
##
# summary(mod.nb.gau)
```

This model does not converge either. An error is returned and no model is fitted.

6.5 "Exponential" method (trap)

```
mod.nb.exp <- glmmTMB(No..eggs.AEDES ~ AREA +
                        poly(Day.ovitrap.collected, degree = 2) +
                        scale(ALTITUDE) +
                        No..Days.ovitrap.in.field +
                        ##
                        exp(pos + 0 | TRAP.ID.fac) +
                        ##
                        (1 | MUNICIPALITY),
```

```
                  family = "nbinom1",
                  data = d.eggs.2019)
```

```
Warning in Matrix::sparseMatrix(dims = c(0, 0), i = integer(0), j =
integer(0), : 'giveCsparse' has been deprecated; setting 'repr = "T"' for you

Warning in Matrix::sparseMatrix(dims = c(0, 0), i = integer(0), j =
integer(0), : 'giveCsparse' has been deprecated; setting 'repr = "T"' for you

Warning in Matrix::sparseMatrix(dims = c(0, 0), i = integer(0), j =
integer(0), : 'giveCsparse' has been deprecated; setting 'repr = "T"' for you

Warning in fitTMB(TMBStruc): Model convergence problem; non-positive-definite
Hessian matrix. See vignette('troubleshooting')
```

```
##
summary(mod.nb.exp)
```

```
 Family: nbinom1  ( log )
Formula:
No..eggs.AEDES ~ AREA + poly(Day.ovitrap.collected, degree = 2) +
    scale(ALTITUDE) + No..Days.ovitrap.in.field + exp(pos + 0 |
    TRAP.ID.fac) + (1 | MUNICIPALITY)
Data: d.eggs.2019

     AIC      BIC   logLik deviance df.resid
      NA       NA       NA       NA      317

Random effects:

Conditional model:
 Groups       Name                       Variance Std.Dev. Corr
 TRAP.ID.fac  pos(8.901817,45.787266) 1.76e-01 4.20e-01
              pos(8.867073,45.787479) 1.76e-01 4.20e-01 0.97
              pos(8.873767,45.788844) 1.76e-01 4.20e-01 0.97 0.99
              pos(8.892238,45.793641) 1.76e-01 4.20e-01 0.99 0.97 0.98
              pos(8.879491,45.798663) 1.76e-01 4.20e-01 0.98 0.98 0.99 0.99
              pos(8.87856,45.802371)  1.76e-01 4.20e-01 0.97 0.98 0.99 0.98
              pos(8.95462,45.82078)   1.76e-01 4.20e-01 0.94 0.91 0.92 0.93
              pos(8.95859,45.82244)   1.76e-01 4.20e-01 0.94 0.91 0.91 0.93
              pos(8.96541,45.8228)    1.76e-01 4.20e-01 0.93 0.90 0.91 0.92
              pos(8.97321,45.82333)   1.76e-01 4.20e-01 0.92 0.89 0.90 0.92
              pos(8.9588,45.82545)    1.76e-01 4.20e-01 0.93 0.91 0.91 0.93
              pos(8.95026,45.82637)   1.76e-01 4.20e-01 0.94 0.91 0.92 0.94
              pos(9.0516,45.83961)    1.76e-01 4.20e-01 0.85 0.83 0.83 0.85
              pos(9.04714,45.84113)   1.76e-01 4.20e-01 0.86 0.83 0.83 0.85
              pos(9.04556,45.84268)   1.76e-01 4.20e-01 0.86 0.83 0.84 0.85
              pos(9.03975,45.84271)   1.76e-01 4.20e-01 0.86 0.83 0.84 0.86
              pos(9.05106,45.8433)    1.76e-01 4.20e-01 0.85 0.83 0.83 0.85
              pos(9.00753,45.84477)   1.76e-01 4.20e-01 0.89 0.86 0.87 0.88
              pos(9.00037,45.84548)   1.76e-01 4.20e-01 0.89 0.86 0.87 0.89
              pos(9.04832,45.84606)   1.76e-01 4.20e-01 0.85 0.83 0.83 0.85
              pos(9.0045,45.8462)     1.76e-01 4.20e-01 0.89 0.86 0.87 0.88
              pos(9.01016,45.84727)   1.76e-01 4.20e-01 0.88 0.86 0.86 0.88
              pos(9.00551,45.84767)   1.76e-01 4.20e-01 0.89 0.86 0.87 0.88
```

```
            pos(9.00475,45.85177)   1.76e-01 4.20e-01 0.89 0.86 0.86 0.88
            pos(8.98971,45.85256)   1.76e-01 4.20e-01 0.90 0.87 0.88 0.89
            pos(8.99377,45.85509)   1.76e-01 4.20e-01 0.89 0.87 0.87 0.89
            pos(8.98085,45.85636)   1.76e-01 4.20e-01 0.90 0.88 0.88 0.90
            pos(8.98858,45.85647)   1.76e-01 4.20e-01 0.89 0.87 0.88 0.89
            pos(8.99154,45.85659)   1.76e-01 4.20e-01 0.89 0.87 0.87 0.89
            pos(8.98427,45.85714)   1.76e-01 4.20e-01 0.90 0.87 0.88 0.89
            pos(8.97825,45.86532)   1.76e-01 4.20e-01 0.90 0.87 0.88 0.89
            pos(8.98278,45.86623)   1.76e-01 4.20e-01 0.89 0.87 0.87 0.89
            pos(8.98227,45.86858)   1.76e-01 4.20e-01 0.89 0.87 0.87 0.89
            pos(8.98584,45.86907)   1.76e-01 4.20e-01 0.89 0.87 0.87 0.89
            pos(8.98868,45.87067)   1.76e-01 4.20e-01 0.89 0.86 0.87 0.88
            pos(8.98596,45.87537)   1.76e-01 4.20e-01 0.89 0.86 0.87 0.88
MUNICIPALITY (Intercept)            4.51e-09 6.72e-05
```

```
1.00
0.92 0.92
0.92 0.92 1.00
0.91 0.91 0.99 0.99
0.91 0.91 0.98 0.99 0.99
0.92 0.92 0.99 1.00 0.99 0.99
0.93 0.93 0.99 0.99 0.98 0.98 0.99
0.84 0.84 0.91 0.91 0.92 0.92 0.91 0.90
0.84 0.84 0.91 0.91 0.92 0.93 0.91 0.91 1.00
0.84 0.84 0.91 0.91 0.92 0.93 0.92 0.91 0.99 1.00
0.85 0.85 0.92 0.92 0.93 0.93 0.92 0.91 0.99 0.99 0.99
0.84 0.84 0.91 0.91 0.92 0.92 0.91 0.90 1.00 1.00 0.99 0.99
0.87 0.87 0.94 0.95 0.95 0.96 0.95 0.94 0.96 0.96 0.96 0.97 0.96
0.88 0.88 0.95 0.95 0.96 0.97 0.95 0.95 0.95 0.95 0.96 0.96 0.95 0.99
0.84 0.84 0.91 0.91 0.92 0.92 0.91 0.90 0.99 0.99 1.00 0.99 1.00 0.96 0.95
0.87 0.88 0.95 0.95 0.96 0.96 0.95 0.94 0.95 0.96 0.96 0.97 0.95 1.00 1.00
0.87 0.87 0.94 0.94 0.95 0.96 0.95 0.94 0.96 0.96 0.96 0.97 0.96 1.00 0.99
0.87 0.87 0.94 0.95 0.95 0.96 0.95 0.94 0.95 0.96 0.96 0.97 0.96 1.00 0.99
0.87 0.87 0.94 0.95 0.95 0.96 0.95 0.94 0.95 0.96 0.96 0.96 0.95 0.99 0.99
0.88 0.89 0.95 0.96 0.96 0.97 0.96 0.95 0.94 0.94 0.94 0.95 0.94 0.98 0.99
0.88 0.88 0.95 0.95 0.96 0.96 0.96 0.95 0.94 0.95 0.95 0.95 0.94 0.98 0.99
0.89 0.89 0.96 0.96 0.96 0.97 0.96 0.96 0.93 0.93 0.94 0.94 0.93 0.97 0.98
0.88 0.88 0.95 0.96 0.96 0.96 0.96 0.95 0.94 0.94 0.94 0.95 0.94 0.98 0.98
0.88 0.88 0.95 0.95 0.96 0.96 0.96 0.95 0.94 0.94 0.95 0.95 0.94 0.98 0.99
0.89 0.89 0.95 0.96 0.96 0.97 0.96 0.96 0.93 0.94 0.94 0.94 0.93 0.97 0.98
0.89 0.89 0.95 0.95 0.96 0.96 0.96 0.95 0.93 0.93 0.93 0.94 0.93 0.96 0.97
0.88 0.88 0.95 0.95 0.95 0.96 0.95 0.95 0.93 0.93 0.94 0.94 0.93 0.97 0.97
0.88 0.88 0.95 0.95 0.95 0.95 0.95 0.95 0.93 0.93 0.93 0.94 0.93 0.97 0.97
0.88 0.88 0.94 0.95 0.95 0.95 0.95 0.95 0.93 0.93 0.94 0.94 0.93 0.97 0.97
0.88 0.88 0.94 0.94 0.95 0.95 0.95 0.94 0.93 0.94 0.94 0.94 0.93 0.97 0.97
0.88 0.88 0.94 0.94 0.95 0.95 0.94 0.94 0.93 0.93 0.93 0.94 0.93 0.96 0.97
```

0.96														
0.96	0.99													
0.96	1.00	1.00												
0.96	0.99	0.99	1.00											
0.94	0.98	0.98	0.98	0.99										
0.95	0.99	0.98	0.99	0.99	1.00									
0.93	0.97	0.97	0.97	0.98	0.99	0.99								
0.94	0.98	0.98	0.98	0.98	1.00	0.99	0.99							
0.94	0.98	0.98	0.98	0.99	1.00	1.00	0.99	1.00						
0.94	0.98	0.97	0.98	0.98	0.99	0.99	1.00	1.00	0.99					
0.93	0.97	0.96	0.97	0.97	0.98	0.98	0.99	0.99	0.98	0.99				
0.93	0.97	0.97	0.97	0.97	0.98	0.98	0.99	0.99	0.99	0.99	1.00			
0.93	0.97	0.97	0.97	0.97	0.98	0.98	0.99	0.99	0.98	0.99	0.99	1.00		
0.94	0.97	0.97	0.97	0.97	0.98	0.98	0.99	0.99	0.99	0.99	0.99	1.00	1.00	
0.94	0.97	0.97	0.97	0.98	0.98	0.98	0.98	0.99	0.99	0.99	0.99	0.99	0.99	1.00
0.93	0.97	0.96	0.97	0.97	0.98	0.98	0.98	0.98	0.98	0.98	0.99	0.99	0.99	0.99

```
 0.99

Number of obs: 327, groups:  TRAP.ID.fac, 36; MUNICIPALITY, 6

Overdispersion parameter for nbinom1 family ():  121

Conditional model:
                                         Estimate Std. Error z value Pr(>|z|)
(Intercept)                                2.4370     0.4678    5.21  1.9e-07
AREANon-intervention                       1.3387     0.1723    7.77  7.8e-15
poly(Day.ovitrap.collected, degree = 2)1   3.9889     1.0696    3.73  0.00019
poly(Day.ovitrap.collected, degree = 2)2 -15.5900     1.0701  -14.57  < 2e-16
scale(ALTITUDE)                            0.0415     0.0807    0.51  0.60690
No..Days.ovitrap.in.field                  0.0909     0.0307    2.96  0.00310

(Intercept)                              ***
AREANon-intervention                     ***
poly(Day.ovitrap.collected, degree = 2)1 ***
poly(Day.ovitrap.collected, degree = 2)2 ***
scale(ALTITUDE)
No..Days.ovitrap.in.field                **
---
Signif. codes:  0 '***' 0.001 '**' 0.01 '*' 0.05 '.' 0.1 ' ' 1
```

```
##
mod.nb.exp$sdr$pdHess
```

```
[1] FALSE
```

Also this model has troubles in converging and all estimates remain unaffected regardless of whether spatial autocorrelation of the random effects is considered or not.

6.6 Including spatial correlation among traps and Municipalities in GLMM models

We add here the model that allow municipality random effects to be correlated in space. We believe that this model should not be fitted as having six unique sites does not allow to estimate a spatial correlation in

a reliable and sensible manner. Nevertheless, we report this model for the sake of completeness.

6.7 "Matern" method (municipality and trap)

```
mod.nb.mat.2 <- glmmTMB(No..eggs.AEDES ~ AREA +
                      poly(Day.ovitrap.collected, degree = 2) +
                      scale(ALTITUDE) +
                      No..Days.ovitrap.in.field +
                      ##
                      mat(pos + 0 | TRAP.ID.fac) +
                      mat(pos + 0 | MUNICIPALITY),
                      ##
                  family = "nbinom1",
                  data = d.eggs.2019)
```

```
Warning in Matrix::sparseMatrix(dims = c(0, 0), i = integer(0), j =
integer(0), : 'giveCsparse' has been deprecated; setting 'repr = "T"' for you

Warning in Matrix::sparseMatrix(dims = c(0, 0), i = integer(0), j =
integer(0), : 'giveCsparse' has been deprecated; setting 'repr = "T"' for you

Warning in Matrix::sparseMatrix(dims = c(0, 0), i = integer(0), j =
integer(0), : 'giveCsparse' has been deprecated; setting 'repr = "T"' for you

Warning in fitTMB(TMBStruc): Model convergence problem; non-positive-definite
Hessian matrix. See vignette('troubleshooting')
```

```
##
summary(mod.nb.mat.2)
```

```
 Family: nbinom1  ( log )
Formula:
No..eggs.AEDES ~ AREA + poly(Day.ovitrap.collected, degree = 2) +
    scale(ALTITUDE) + No..Days.ovitrap.in.field + mat(pos + 0 |
    TRAP.ID.fac) + mat(pos + 0 | MUNICIPALITY)
Data: d.eggs.2019

     AIC      BIC   logLik deviance df.resid
      NA       NA       NA       NA      314

Random effects:

Conditional model:
 Groups       Name                     Variance Std.Dev. Corr
 TRAP.ID.fac  pos(8.901817,45.787266) 1.76e-01 0.41961
              pos(8.867073,45.787479) 1.76e-01 0.41961  1.00
              pos(8.873767,45.788844) 1.76e-01 0.41961  1.00 1.00
              pos(8.892238,45.793641) 1.76e-01 0.41961  1.00 1.00 1.00
              pos(8.879491,45.798663) 1.76e-01 0.41961  1.00 1.00 1.00 1.00
              pos(8.87856,45.802371)  1.76e-01 0.41961  1.00 1.00 1.00 1.00
              pos(8.95462,45.82078)   1.76e-01 0.41961  0.99 0.99 0.99 0.99
              pos(8.95859,45.82244)   1.76e-01 0.41961  0.99 0.99 0.99 0.99
              pos(8.96541,45.8228)    1.76e-01 0.41961  0.99 0.98 0.99 0.99
              pos(8.97321,45.82333)   1.76e-01 0.41961  0.99 0.98 0.98 0.99
              pos(8.9588,45.82545)    1.76e-01 0.41961  0.99 0.99 0.99 0.99
```

	pos(8.95026,45.82637)	1.76e-01	0.41961	0.99	0.99	0.99	0.99
	pos(9.0516,45.83961)	1.76e-01	0.41961	0.97	0.96	0.96	0.97
	pos(9.04714,45.84113)	1.76e-01	0.41961	0.97	0.96	0.96	0.97
	pos(9.04556,45.84268)	1.76e-01	0.41961	0.97	0.96	0.96	0.97
	pos(9.03975,45.84271)	1.76e-01	0.41961	0.97	0.96	0.96	0.97
	pos(9.05106,45.8433)	1.76e-01	0.41961	0.97	0.96	0.96	0.97
	pos(9.00753,45.84477)	1.76e-01	0.41961	0.98	0.97	0.97	0.98
	pos(9.00037,45.84548)	1.76e-01	0.41961	0.98	0.97	0.97	0.98
	pos(9.04832,45.84606)	1.76e-01	0.41961	0.97	0.96	0.96	0.97
	pos(9.0045,45.8462)	1.76e-01	0.41961	0.98	0.97	0.97	0.98
	pos(9.01016,45.84727)	1.76e-01	0.41961	0.98	0.97	0.97	0.98
	pos(9.00551,45.84767)	1.76e-01	0.41961	0.98	0.97	0.97	0.98
	pos(9.00475,45.85177)	1.76e-01	0.41961	0.98	0.97	0.97	0.98
	pos(8.98971,45.85256)	1.76e-01	0.41961	0.98	0.97	0.98	0.98
	pos(8.99377,45.85509)	1.76e-01	0.41961	0.98	0.97	0.98	0.98
	pos(8.98085,45.85636)	1.76e-01	0.41961	0.98	0.98	0.98	0.98
	pos(8.98858,45.85647)	1.76e-01	0.41961	0.98	0.97	0.98	0.98
	pos(8.99154,45.85659)	1.76e-01	0.41961	0.98	0.97	0.98	0.98
	pos(8.98427,45.85714)	1.76e-01	0.41961	0.98	0.98	0.98	0.98
	pos(8.97825,45.86532)	1.76e-01	0.41961	0.98	0.98	0.98	0.98
	pos(8.98278,45.86623)	1.76e-01	0.41961	0.98	0.97	0.98	0.98
	pos(8.98227,45.86858)	1.76e-01	0.41961	0.98	0.97	0.98	0.98
	pos(8.98584,45.86907)	1.76e-01	0.41961	0.98	0.97	0.98	0.98
	pos(8.98868,45.87067)	1.76e-01	0.41961	0.98	0.97	0.97	0.98
	pos(8.98596,45.87537)	1.76e-01	0.41961	0.98	0.97	0.97	0.98
MUNICIPALITY	pos(8.901817,45.787266)	2.08e-06	0.00144				
	pos(8.867073,45.787479)	2.08e-06	0.00144	0.00			
	pos(8.873767,45.788844)	2.08e-06	0.00144	0.00	0.00		
	pos(8.892238,45.793641)	2.08e-06	0.00144	0.00	0.00	0.00	
	pos(8.879491,45.798663)	2.08e-06	0.00144	0.00	0.00	0.00	0.00
	pos(8.87856,45.802371)	2.08e-06	0.00144	0.00	0.00	0.00	0.00
	pos(8.95462,45.82078)	2.08e-06	0.00144	0.00	0.00	0.00	0.00
	pos(8.95859,45.82244)	2.08e-06	0.00144	0.00	0.00	0.00	0.00
	pos(8.96541,45.8228)	2.08e-06	0.00144	0.00	0.00	0.00	0.00
	pos(8.97321,45.82333)	2.08e-06	0.00144	0.00	0.00	0.00	0.00
	pos(8.9588,45.82545)	2.08e-06	0.00144	0.00	0.00	0.00	0.00
	pos(8.95026,45.82637)	2.08e-06	0.00144	0.00	0.00	0.00	0.00
	pos(9.0516,45.83961)	2.08e-06	0.00144	0.00	0.00	0.00	0.00
	pos(9.04714,45.84113)	2.08e-06	0.00144	0.00	0.00	0.00	0.00

1.00								
0.99	0.99							
0.99	0.99	1.00						
0.99	0.99	1.00	1.00					
0.99	0.99	1.00	1.00	1.00				
0.99	0.99	1.00	1.00	1.00	1.00			
0.99	0.99	1.00	1.00	1.00	1.00	1.00		
0.96	0.96	0.99	0.99	0.99	0.99	0.99	0.98	
0.96	0.96	0.99	0.99	0.99	0.99	0.99	0.99	1.00

0.96 0.96 0.99 0.99 0.99 0.99 0.99 0.99 1.00 1.00
0.97 0.97 0.99 0.99 0.99 0.99 0.99 0.99 1.00 1.00 1.00
0.96 0.96 0.99 0.99 0.99 0.99 0.99 0.98 1.00 1.00 1.00 1.00
0.98 0.98 0.99 0.99 1.00 1.00 1.00 0.99 1.00 1.00 1.00 1.00 1.00
0.98 0.98 1.00 1.00 1.00 1.00 1.00 0.99 1.00 1.00 1.00 1.00 1.00 1.00
0.96 0.96 0.99 0.99 0.99 0.99 0.99 0.99 1.00 1.00 1.00 1.00 1.00 1.00 1.00
0.98 0.98 0.99 1.00 1.00 1.00 1.00 0.99 1.00 1.00 1.00 1.00 1.00 1.00 1.00
0.97 0.97 0.99 0.99 1.00 1.00 0.99 0.99 1.00 1.00 1.00 1.00 1.00 1.00 1.00
0.98 0.98 0.99 0.99 1.00 1.00 1.00 0.99 1.00 1.00 1.00 1.00 1.00 1.00 1.00
0.98 0.98 0.99 0.99 1.00 1.00 1.00 0.99 1.00 1.00 1.00 1.00 1.00 1.00 1.00
0.98 0.98 1.00 1.00 1.00 1.00 1.00 1.00 0.99 0.99 0.99 1.00 0.99 1.00 1.00
0.98 0.98 1.00 1.00 1.00 1.00 1.00 1.00 0.99 0.99 0.99 1.00 0.99 1.00 1.00
0.98 0.98 1.00 1.00 1.00 1.00 1.00 1.00 0.99 0.99 0.99 0.99 0.99 1.00 1.00
0.98 0.98 1.00 1.00 1.00 1.00 1.00 1.00 0.99 0.99 0.99 1.00 0.99 1.00 1.00
0.98 0.98 1.00 1.00 1.00 1.00 1.00 1.00 0.99 0.99 0.99 1.00 0.99 1.00 1.00
0.98 0.98 1.00 1.00 1.00 1.00 1.00 1.00 0.99 0.99 0.99 0.99 0.99 1.00 1.00
0.98 0.98 1.00 1.00 1.00 1.00 1.00 1.00 0.99 0.99 0.99 0.99 0.99 1.00 1.00
0.98 0.98 0.99 1.00 1.00 1.00 1.00 1.00 0.99 0.99 0.99 0.99 0.99 1.00 1.00
0.98 0.98 0.99 1.00 1.00 1.00 1.00 1.00 0.99 0.99 0.99 0.99 0.99 1.00 1.00
0.98 0.98 0.99 0.99 1.00 1.00 1.00 0.99 0.99 0.99 0.99 0.99 0.99 1.00 1.00
0.98 0.98 0.99 0.99 0.99 1.00 0.99 0.99 0.99 0.99 0.99 0.99 0.99 1.00 1.00
0.98 0.98 0.99 0.99 0.99 0.99 0.99 0.99 0.99 0.99 0.99 0.99 0.99 1.00 1.00

0.01
0.00 0.00
0.00 0.00 0.01
0.00 0.00 0.00 0.00
0.00 0.00 0.00 0.00 0.00
0.00 0.00 0.00 0.01 0.00 0.00
0.00 0.00 0.00 0.00 0.00 0.00 0.00
0.00 0.00 0.00 0.00 0.00 0.00 0.00 0.00
0.00 0.00 0.00 0.00 0.00 0.00 0.00 0.00 0.01

```
1.00
1.00 1.00
1.00 1.00 1.00
1.00 1.00 1.00 1.00
0.99 1.00 1.00 1.00 1.00
0.99 1.00 1.00 1.00 1.00 1.00
0.99 1.00 1.00 1.00 1.00 1.00 1.00
0.99 1.00 1.00 1.00 1.00 1.00 1.00 1.00
0.99 1.00 1.00 1.00 1.00 1.00 1.00 1.00 1.00
0.99 1.00 1.00 1.00 1.00 1.00 1.00 1.00 1.00 1.00
0.99 1.00 1.00 1.00 1.00 1.00 1.00 1.00 1.00 1.00 1.00
0.99 1.00 1.00 1.00 1.00 1.00 1.00 1.00 1.00 1.00 1.00 1.00
0.99 1.00 1.00 1.00 1.00 1.00 1.00 1.00 1.00 1.00 1.00 1.00 1.00
0.99 1.00 1.00 1.00 1.00 1.00 1.00 1.00 1.00 1.00 1.00 1.00 1.00 1.00
0.99 1.00 1.00 1.00 1.00 1.00 1.00 1.00 1.00 1.00 1.00 1.00 1.00 1.00 1.00
0.99 1.00 1.00 1.00 1.00 1.00 1.00 1.00 1.00 1.00 1.00 1.00 1.00 1.00 1.00
```

```
 1.00
```

```
 [ reached getOption("max.print") -- omitted 22 rows ]
Number of obs: 327, groups:  TRAP.ID.fac, 36; MUNICIPALITY, 6

Overdispersion parameter for nbinom1 family ():  121

Conditional model:
                                        Estimate Std. Error z value Pr(>|z|)
(Intercept)                               2.4370     0.4678    5.21  1.9e-07
AREANon-intervention                      1.3387     0.1723    7.77  7.8e-15
poly(Day.ovitrap.collected, degree = 2)1   3.9889     1.0696    3.73  0.00019
poly(Day.ovitrap.collected, degree = 2)2 -15.5900     1.0701  -14.57  < 2e-16
scale(ALTITUDE)                           0.0415     0.0807    0.51  0.60691
No..Days.ovitrap.in.field                 0.0909     0.0307    2.96  0.00310

(Intercept)                              ***
AREANon-intervention                     ***
poly(Day.ovitrap.collected, degree = 2)1 ***
poly(Day.ovitrap.collected, degree = 2)2 ***
scale(ALTITUDE)
No..Days.ovitrap.in.field                **
---
Signif. codes:  0 '***' 0.001 '**' 0.01 '*' 0.05 '.' 0.1 ' ' 1
```

```
##
mod.nb.mat.2$sdr$pdHess
```

```
[1] FALSE
```

Also this model has troubles in converging and all estimates remain unaffected regardless of whether spatial autocorrelation of the random effects is considered or not.

6.8 "Gaussian" method (municipality and trap)

```
mod.nb.gau.2 <- glmmTMB(No..eggs.AEDES ~ AREA +
                          poly(Day.ovitrap.collected, degree = 2) +
                          scale(ALTITUDE) +
                          No..Days.ovitrap.in.field +
                          ##
                          gau(pos + 0 | TRAP.ID.fac) +
                          gau(pos + 0 | MUNICIPALITY),
                          ##
                      family = "nbinom1",
                      data = d.eggs.2019)
```

```
Warning in Matrix::sparseMatrix(dims = c(0, 0), i = integer(0), j =
integer(0), : 'giveCsparse' has been deprecated; setting 'repr = "T"' for you

Warning in Matrix::sparseMatrix(dims = c(0, 0), i = integer(0), j =
integer(0), : 'giveCsparse' has been deprecated; setting 'repr = "T"' for you

Warning in Matrix::sparseMatrix(dims = c(0, 0), i = integer(0), j =
integer(0), : 'giveCsparse' has been deprecated; setting 'repr = "T"' for you

Warning in (function (start, objective, gradient = NULL, hessian = NULL, : NA/
NaN function evaluation

Error in (function (start, objective, gradient = NULL, hessian = NULL, : gradient function must return [illegible]

Timing stopped at: 3.06 0.01 3.08
```

This model does not converge either. An error is returned and no model is fitted.

6.9 "Exponential" method (municipality and trap)

```
mod.nb.exp.2 <- glmmTMB(No..eggs.AEDES ~ AREA +
                          poly(Day.ovitrap.collected, degree = 2) +
                          scale(ALTITUDE) +
                          No..Days.ovitrap.in.field +
                          ##
                          exp(pos + 0 | TRAP.ID.fac) +
                          exp(pos + 0 | MUNICIPALITY),
                          ##
                      family = "nbinom1",
                      data = d.eggs.2019)
```

```
Warning in Matrix::sparseMatrix(dims = c(0, 0), i = integer(0), j =
integer(0), : 'giveCsparse' has been deprecated; setting 'repr = "T"' for you

Warning in Matrix::sparseMatrix(dims = c(0, 0), i = integer(0), j =
```

```
integer(0), : 'giveCsparse' has been deprecated; setting 'repr = "T"' for you

Warning in Matrix::sparseMatrix(dims = c(0, 0), i = integer(0), j =
integer(0), : 'giveCsparse' has been deprecated; setting 'repr = "T"' for you

Warning in fitTMB(TMBStruc): Model convergence problem; non-positive-definite
Hessian matrix. See vignette('troubleshooting')
```

```
##
summary(mod.nb.exp.2)
```

```
 Family: nbinom1  ( log )
Formula:
No..eggs.AEDES ~ AREA + poly(Day.ovitrap.collected, degree = 2) +
    scale(ALTITUDE) + No..Days.ovitrap.in.field + exp(pos + 0 |
    TRAP.ID.fac) + exp(pos + 0 | MUNICIPALITY)
Data: d.eggs.2019

     AIC      BIC   logLik deviance df.resid
      NA       NA       NA       NA      316

Random effects:

Conditional model:
 Groups       Name                       Variance Std.Dev. Corr
 TRAP.ID.fac  pos(8.901817,45.787266) 0.17334  0.4163
              pos(8.867073,45.787479) 0.17334  0.4163   0.97
              pos(8.873767,45.788844) 0.17334  0.4163   0.97 0.99
              pos(8.892238,45.793641) 0.17334  0.4163   0.99 0.97 0.98
              pos(8.879491,45.798663) 0.17334  0.4163   0.98 0.98 0.99 0.99
              pos(8.87856,45.802371)  0.17334  0.4163   0.97 0.98 0.99 0.98
              pos(8.95462,45.82078)   0.17334  0.4163   0.94 0.91 0.92 0.93
              pos(8.95859,45.82244)   0.17334  0.4163   0.94 0.91 0.91 0.93
              pos(8.96541,45.8228)    0.17334  0.4163   0.93 0.90 0.91 0.92
              pos(8.97321,45.82333)   0.17334  0.4163   0.92 0.89 0.90 0.92
              pos(8.9588,45.82545)    0.17334  0.4163   0.93 0.91 0.91 0.93
              pos(8.95026,45.82637)   0.17334  0.4163   0.94 0.91 0.92 0.94
              pos(9.0516,45.83961)    0.17334  0.4163   0.85 0.83 0.83 0.85
              pos(9.04714,45.84113)   0.17334  0.4163   0.86 0.83 0.83 0.85
              pos(9.04556,45.84268)   0.17334  0.4163   0.86 0.83 0.84 0.85
              pos(9.03975,45.84271)   0.17334  0.4163   0.86 0.83 0.84 0.86
              pos(9.05106,45.8433)    0.17334  0.4163   0.85 0.83 0.83 0.85
              pos(9.00753,45.84477)   0.17334  0.4163   0.89 0.86 0.87 0.88
              pos(9.00037,45.84548)   0.17334  0.4163   0.89 0.86 0.87 0.89
              pos(9.04832,45.84606)   0.17334  0.4163   0.85 0.83 0.83 0.85
              pos(9.0045,45.8462)     0.17334  0.4163   0.89 0.86 0.87 0.88
              pos(9.01016,45.84727)   0.17334  0.4163   0.88 0.86 0.86 0.88
              pos(9.00551,45.84767)   0.17334  0.4163   0.89 0.86 0.87 0.88
              pos(9.00475,45.85177)   0.17334  0.4163   0.89 0.86 0.86 0.88
              pos(8.98971,45.85256)   0.17334  0.4163   0.90 0.87 0.88 0.89
              pos(8.99377,45.85509)   0.17334  0.4163   0.89 0.87 0.87 0.89
              pos(8.98085,45.85636)   0.17334  0.4163   0.90 0.88 0.88 0.90
              pos(8.98858,45.85647)   0.17334  0.4163   0.89 0.87 0.88 0.89
              pos(8.99154,45.85659)   0.17334  0.4163   0.89 0.87 0.87 0.89
              pos(8.98427,45.85714)   0.17334  0.4163   0.90 0.87 0.88 0.89
```

	pos(8.97825,45.86532)	0.17334	0.4163	0.90	0.87	0.88	0.89
	pos(8.98278,45.86623)	0.17334	0.4163	0.89	0.87	0.87	0.89
	pos(8.98227,45.86858)	0.17334	0.4163	0.89	0.87	0.87	0.89
	pos(8.98584,45.86907)	0.17334	0.4163	0.89	0.87	0.87	0.89
	pos(8.98868,45.87067)	0.17334	0.4163	0.89	0.86	0.87	0.88
	pos(8.98596,45.87537)	0.17334	0.4163	0.89	0.86	0.87	0.88
MUNICIPALITY	pos(8.901817,45.787266)	0.00274	0.0523				
	pos(8.867073,45.787479)	0.00274	0.0523	0.00			
	pos(8.873767,45.788844)	0.00274	0.0523	0.00	0.00		
	pos(8.892238,45.793641)	0.00274	0.0523	0.00	0.00	0.00	
	pos(8.879491,45.798663)	0.00274	0.0523	0.00	0.00	0.00	0.00
	pos(8.87856,45.802371)	0.00274	0.0523	0.00	0.00	0.00	0.00
	pos(8.95462,45.82078)	0.00274	0.0523	0.00	0.00	0.00	0.00
	pos(8.95859,45.82244)	0.00274	0.0523	0.00	0.00	0.00	0.00
	pos(8.96541,45.8228)	0.00274	0.0523	0.00	0.00	0.00	0.00
	pos(8.97321,45.82333)	0.00274	0.0523	0.00	0.00	0.00	0.00
	pos(8.9588,45.82545)	0.00274	0.0523	0.00	0.00	0.00	0.00
	pos(8.95026,45.82637)	0.00274	0.0523	0.00	0.00	0.00	0.00
	pos(9.0516,45.83961)	0.00274	0.0523	0.00	0.00	0.00	0.00
	pos(9.04714,45.84113)	0.00274	0.0523	0.00	0.00	0.00	0.00

1.00														
0.92	0.92													
0.92	0.92	1.00												
0.91	0.91	0.99	0.99											
0.91	0.91	0.98	0.99	0.99										
0.92	0.92	0.99	1.00	0.99	0.99									
0.93	0.93	0.99	0.99	0.98	0.98	0.99								
0.84	0.84	0.91	0.91	0.92	0.92	0.91	0.90							
0.84	0.84	0.91	0.91	0.92	0.93	0.91	0.91	1.00						
0.84	0.84	0.91	0.91	0.92	0.93	0.92	0.91	0.99	1.00					
0.85	0.85	0.92	0.92	0.93	0.93	0.92	0.91	0.99	0.99	0.99				
0.84	0.84	0.91	0.91	0.92	0.92	0.91	0.90	1.00	1.00	0.99	0.99			
0.87	0.87	0.94	0.95	0.95	0.96	0.95	0.94	0.96	0.96	0.96	0.97	0.96		
0.88	0.88	0.95	0.95	0.96	0.97	0.95	0.95	0.95	0.95	0.96	0.96	0.95	0.99	
0.84	0.84	0.91	0.91	0.92	0.92	0.91	0.90	0.99	0.99	1.00	0.99	1.00	0.96	0.95
0.87	0.88	0.95	0.95	0.96	0.96	0.95	0.94	0.95	0.96	0.96	0.97	0.95	1.00	1.00
0.87	0.87	0.94	0.94	0.95	0.96	0.95	0.94	0.96	0.96	0.96	0.97	0.96	1.00	0.99
0.87	0.87	0.94	0.95	0.95	0.96	0.95	0.94	0.95	0.96	0.96	0.97	0.96	1.00	0.99
0.87	0.87	0.94	0.95	0.95	0.96	0.95	0.94	0.95	0.96	0.96	0.96	0.95	0.99	0.99
0.88	0.89	0.95	0.96	0.96	0.97	0.96	0.95	0.94	0.94	0.94	0.95	0.94	0.98	0.99
0.88	0.88	0.95	0.95	0.96	0.96	0.96	0.95	0.94	0.95	0.95	0.95	0.94	0.98	0.99
0.89	0.89	0.96	0.96	0.96	0.97	0.96	0.96	0.93	0.93	0.94	0.94	0.93	0.97	0.98
0.88	0.88	0.95	0.96	0.96	0.96	0.96	0.95	0.94	0.94	0.94	0.95	0.94	0.98	0.98
0.88	0.88	0.95	0.95	0.96	0.96	0.96	0.95	0.94	0.94	0.95	0.95	0.94	0.98	0.99
0.89	0.89	0.95	0.96	0.96	0.97	0.96	0.96	0.93	0.94	0.94	0.94	0.93	0.97	0.98
0.89	0.89	0.95	0.95	0.96	0.96	0.96	0.95	0.93	0.93	0.93	0.94	0.93	0.96	0.97
0.88	0.88	0.95	0.95	0.95	0.96	0.95	0.95	0.93	0.93	0.94	0.94	0.93	0.97	0.97
0.88	0.88	0.95	0.95	0.95	0.95	0.95	0.95	0.93	0.93	0.93	0.94	0.93	0.97	0.97

0.88	0.88	0.94	0.95	0.95	0.95	0.95	0.95	0.93	0.93	0.94	0.94	0.93	0.97	0.97
0.88	0.88	0.94	0.94	0.95	0.95	0.95	0.94	0.93	0.94	0.94	0.94	0.93	0.97	0.97
0.88	0.88	0.94	0.94	0.95	0.95	0.94	0.94	0.93	0.93	0.93	0.94	0.93	0.96	0.97

0.00								
0.00	0.00							
0.00	0.00	0.00						
0.00	0.00	0.00	0.00					
0.00	0.00	0.00	0.00	0.00				
0.00	0.00	0.00	0.01	0.00	0.00			
0.00	0.00	0.00	0.00	0.00	0.00	0.00		
0.00	0.00	0.00	0.00	0.00	0.00	0.00	0.00	
0.00	0.00	0.00	0.00	0.00	0.00	0.00	0.00	0.00

0.96														
0.96	0.99													
0.96	1.00	1.00												
0.96	0.99	0.99	1.00											
0.94	0.98	0.98	0.98	0.99										
0.95	0.99	0.98	0.99	0.99	1.00									
0.93	0.97	0.97	0.97	0.98	0.99	0.99								
0.94	0.98	0.98	0.98	0.98	1.00	0.99	0.99							
0.94	0.98	0.98	0.98	0.99	1.00	1.00	0.99	1.00						
0.94	0.98	0.97	0.98	0.98	0.99	0.99	1.00	1.00	0.99					
0.93	0.97	0.96	0.97	0.97	0.98	0.98	0.99	0.99	0.98	0.99				
0.93	0.97	0.97	0.97	0.97	0.98	0.98	0.99	0.99	0.99	0.99	1.00			
0.93	0.97	0.97	0.97	0.97	0.98	0.98	0.99	0.99	0.98	0.99	0.99	1.00		
0.94	0.97	0.97	0.97	0.97	0.98	0.98	0.99	0.99	0.99	0.99	0.99	1.00	1.00	
0.94	0.97	0.97	0.97	0.98	0.98	0.98	0.98	0.99	0.99	0.99	0.99	0.99	0.99	1.00
0.93	0.97	0.96	0.97	0.97	0.98	0.98	0.98	0.98	0.98	0.98	0.99	0.99	0.99	0.99

0.99

```
 [ reached getOption("max.print") -- omitted 22 rows ]
Number of obs: 327, groups:  TRAP.ID.fac, 36; MUNICIPALITY, 6

Overdispersion parameter for nbinom1 family ():  121

Conditional model:
                                         Estimate Std. Error z value Pr(>|z|)
(Intercept)                                2.4371     0.4678    5.21  1.9e-07
AREANon-intervention                       1.3387     0.1723    7.77  7.8e-15
poly(Day.ovitrap.collected, degree = 2)1   3.9889     1.0696    3.73  0.00019
poly(Day.ovitrap.collected, degree = 2)2 -15.5901     1.0701  -14.57  < 2e-16
scale(ALTITUDE)                            0.0415     0.0807    0.51  0.60697
No..Days.ovitrap.in.field                  0.0909     0.0307    2.96  0.00310

(Intercept)                              ***
AREANon-intervention                     ***
poly(Day.ovitrap.collected, degree = 2)1 ***
poly(Day.ovitrap.collected, degree = 2)2 ***
scale(ALTITUDE)
No..Days.ovitrap.in.field                **
---
Signif. codes:  0 '***' 0.001 '**' 0.01 '*' 0.05 '.' 0.1 ' ' 1
```

```
##
mod.nb.exp.2$sdr$pdHess
```

```
[1] FALSE
```

Also this model has troubles in converging and all estimates remain unaffected regardless of whether spatial autocorrelation of the random effects is considered or not.

To summarise this section: **By looking at semi-variograms we were not able to see any indication that the trap random effects are spatially correlated. The three models we fitted where trap random effects can be correlated did not show any difference with the original analysis.**

Note that we also fitted where municipality random effects allowed to be correlated. We added these models, none of which converged, for the sake of completeness. Indeed, we believe that six observations is not a reasonable sample to estimate in a reliable manner spatial correlation.

So, including spatially correlated random effects does not lead to any change in the results. In other words, the model does not benefit from including the spatial correlation.

7 Dropping municipality from the random effects

Prof. Martin Mächler suggested to drop Municipality from the analysis following the Occam's Razor principle.

```r
mod.nb.NoMuni <- update(mod.nb.1, . ~ . - (1 | MUNICIPALITY))
```

```
Warning in Matrix::sparseMatrix(dims = c(0, 0), i = integer(0), j =
integer(0), : 'giveCsparse' has been deprecated; setting 'repr = "T"' for you

Warning in Matrix::sparseMatrix(dims = c(0, 0), i = integer(0), j =
integer(0), : 'giveCsparse' has been deprecated; setting 'repr = "T"' for you

Warning in Matrix::sparseMatrix(dims = c(0, 0), i = integer(0), j =
integer(0), : 'giveCsparse' has been deprecated; setting 'repr = "T"' for you
```

```r
##
summary(mod.nb.NoMuni)
```

```
 Family: nbinom1  ( log )
Formula:
No..eggs.AEDES ~ AREA + poly(Day.ovitrap.collected, degree = 2) +
    scale(ALTITUDE) + No..Days.ovitrap.in.field + (1 | TRAP.ID.fac)
Data: d.eggs.2019

     AIC      BIC   logLik deviance df.resid
    3334     3364    -1659     3318      319

Random effects:

Conditional model:
 Groups      Name        Variance Std.Dev.
 TRAP.ID.fac (Intercept) 0.176    0.42
Number of obs: 327, groups:  TRAP.ID.fac, 36

Overdispersion parameter for nbinom1 family ():  121

Conditional model:
                                         Estimate Std. Error z value Pr(>|z|)
(Intercept)                                2.4370     0.4678    5.21  1.9e-07
AREANon-intervention                       1.3387     0.1723    7.77  7.8e-15
poly(Day.ovitrap.collected, degree = 2)1   3.9889     1.0696    3.73  0.00019
poly(Day.ovitrap.collected, degree = 2)2 -15.5901     1.0701  -14.57  < 2e-16
scale(ALTITUDE)                            0.0415     0.0807    0.51  0.60690
No..Days.ovitrap.in.field                  0.0909     0.0307    2.96  0.00310

(Intercept)                              ***
AREANon-intervention                     ***
poly(Day.ovitrap.collected, degree = 2)1 ***
poly(Day.ovitrap.collected, degree = 2)2 ***
scale(ALTITUDE)
No..Days.ovitrap.in.field                **
---
Signif. codes:  0 '***' 0.001 '**' 0.01 '*' 0.05 '.' 0.1 ' ' 1
```

Let's look at the estimated effect for "AREA".

```r
exp(fixef(mod.nb.NoMuni)$cond["AREANon-intervention"])
```

```
AREANon-intervention
                 3.8
```

As expected the results are virtually the same.

Note, that in our previous analysis we preferred not to drop “municipality” such that no model selection is carried out and all p-values can be interpret as usual (this is a more philosophical point and irrelevant here).

8 Municipality as a fixed effect

Let’s take municipality as a fixed effect and (therefore) drop “AREA” from the model. We will then run a post-hoc test to compare the two “AREA” groups.

```r
## (warning are omitted from this chunk)
##
mod.nb.1.Muni_Fixed <- glmmTMB(No..eggs.AEDES ~ MUNICIPALITY +
                                 # AREA +
                      poly(Day.ovitrap.collected, degree = 2) +
                      scale(ALTITUDE) +
                      No..Days.ovitrap.in.field +
                      (1 | TRAP.ID.fac),
                  family = "nbinom1",
                  data = d.eggs.2019)
##
summary(mod.nb.1.Muni_Fixed)
```

```
 Family: nbinom1  ( log )
Formula:
No..eggs.AEDES ~ MUNICIPALITY + poly(Day.ovitrap.collected, degree = 2) +
    scale(ALTITUDE) + No..Days.ovitrap.in.field + (1 | TRAP.ID.fac)
Data: d.eggs.2019

     AIC      BIC   logLik deviance df.resid
    3336     3382    -1656     3312      315

Random effects:

Conditional model:
 Groups      Name        Variance Std.Dev.
 TRAP.ID.fac (Intercept) 0.145    0.381
Number of obs: 327, groups:  TRAP.ID.fac, 36

Overdispersion parameter for nbinom1 family ():  121

Conditional model:
                                         Estimate Std. Error z value Pr(>|z|)
(Intercept)                                2.5183     0.5221    4.82  1.4e-06
MUNICIPALITYColdrerio                      0.1373     0.3880    0.35  0.72353
MUNICIPALITYMendrisio                     -0.3762     0.3979   -0.95  0.34448
MUNICIPALITYMalnate                        1.2266     0.3893    3.15  0.00163
MUNICIPALITYMaslianico                     1.7003     0.3212    5.29  1.2e-07
MUNICIPALITYUggiate-Trevano                0.8065     0.5218    1.55  0.12222
poly(Day.ovitrap.collected, degree = 2)1   4.1140     1.0715    3.84  0.00012
poly(Day.ovitrap.collected, degree = 2)2 -15.6556     1.0676  -14.66  < 2e-16
scale(ALTITUDE)                            0.2745     0.2084    1.32  0.18776
No..Days.ovitrap.in.field                  0.0909     0.0307    2.96  0.00309

(Intercept)                              ***
```

```
MUNICIPALITYColdrerio
MUNICIPALITYMendrisio
MUNICIPALITYMalnate                    **
MUNICIPALITYMaslianico                 ***
MUNICIPALITYUggiate-Trevano
poly(Day.ovitrap.collected, degree = 2)1 ***
poly(Day.ovitrap.collected, degree = 2)2 ***
scale(ALTITUDE)
No..Days.ovitrap.in.field              **
---
Signif. codes:  0 '***' 0.001 '**' 0.01 '*' 0.05 '.' 0.1 ' ' 1
```

Let's look at the single parameters. Note that the intercept refers to Balerna and that all the other municipalities are the difference to the reference (treatment contrasts are being used).

```
fixef(mod.nb.1.Muni_Fixed)$cond[1:6]
```

```
                (Intercept)       MUNICIPALITYColdrerio
                       2.52                        0.14
      MUNICIPALITYMendrisio         MUNICIPALITYMalnate
                      -0.38                        1.23
     MUNICIPALITYMaslianico MUNICIPALITYUggiate-Trevano
                       1.70                        0.81
```

There seems to be some differences between "intervention" and non "intervention" municipalities. Let's formally compare these two groups.

```
## (message are omitted from this chunk)
##
levels(d.eggs.2019$MUNICIPALITY)
```

```
[1] "Balerna"         "Coldrerio"       "Mendrisio"       "Malnate"
[5] "Maslianico"      "Uggiate-Trevano"
```

```
glht.contrast <- glht(mod.nb.1.Muni_Fixed,
     linfct = mcp(MUNICIPALITY = c(-1/3, -1/3, -1/3,
                                   1/3, 1/3, 1/3)))
summary(glht.contrast)
```

```
     Simultaneous Tests for General Linear Hypotheses

Multiple Comparisons of Means: User-defined Contrasts


Fit: glmmTMB(formula = No..eggs.AEDES ~ MUNICIPALITY + poly(Day.ovitrap.collected,
    degree = 2) + scale(ALTITUDE) + No..Days.ovitrap.in.field +
    (1 | TRAP.ID.fac), data = d.eggs.2019, family = "nbinom1",
    ziformula = ~0, dispformula = ~1)

Linear Hypotheses:
       Estimate Std. Error z value Pr(>|z|)
1 == 0    1.324      0.163    8.13  4.4e-16 ***
---
Signif. codes:  0 '***' 0.001 '**' 0.01 '*' 0.05 '.' 0.1 ' ' 1
(Adjusted p values reported -- single-step method)
```

The post-hoc test confirms that there is strong evidence for a difference between the two groups. We can take the exponential of this difference to quantify the multiplicative difference number of eggs in "Non-

intervention" municipalities.

```
exp(1.324)
```

```
[1] 3.8
```

There are almost 4 times more eggs in non-treated municipalities. **This is fully consistent with the other analysis where "MUNICIPALITY" is taken as a random effect and "AREA" as a fixed effect.**

9 Bayesian GLMM

We fit a Bayesian Mixed-Effects Model using the {rstanarm} package.

```
stan.nb.1 <- stan_glmer.nb(No..eggs.AEDES ~ AREA +
                      poly(Day.ovitrap.collected, degree = 2) +
                      scale(ALTITUDE) +
                      No..Days.ovitrap.in.field +
                      (1 | TRAP.ID.fac) + (1 | MUNICIPALITY),
                  ## NB: stan_glmer.nb() implements NB2
                  data = d.eggs.2019)
```

```
SAMPLING FOR MODEL 'count' NOW (CHAIN 1).
Chain 1:
Chain 1: Gradient evaluation took 0 seconds
Chain 1: 1000 transitions using 10 leapfrog steps per transition would take 0 seconds.
Chain 1: Adjust your expectations accordingly!
Chain 1:
Chain 1:
Chain 1: Iteration:    1 / 2000 [  0%]  (Warmup)
Chain 1: Iteration:  200 / 2000 [ 10%]  (Warmup)
Chain 1: Iteration:  400 / 2000 [ 20%]  (Warmup)
Chain 1: Iteration:  600 / 2000 [ 30%]  (Warmup)
Chain 1: Iteration:  800 / 2000 [ 40%]  (Warmup)
Chain 1: Iteration: 1000 / 2000 [ 50%]  (Warmup)
Chain 1: Iteration: 1001 / 2000 [ 50%]  (Sampling)
Chain 1: Iteration: 1200 / 2000 [ 60%]  (Sampling)
Chain 1: Iteration: 1400 / 2000 [ 70%]  (Sampling)
Chain 1: Iteration: 1600 / 2000 [ 80%]  (Sampling)
Chain 1: Iteration: 1800 / 2000 [ 90%]  (Sampling)
Chain 1: Iteration: 2000 / 2000 [100%]  (Sampling)
Chain 1:
Chain 1:  Elapsed Time: 8.054 seconds (Warm-up)
Chain 1:                4.758 seconds (Sampling)
Chain 1:                12.812 seconds (Total)
Chain 1:

SAMPLING FOR MODEL 'count' NOW (CHAIN 2).
Chain 2:
Chain 2: Gradient evaluation took 0 seconds
Chain 2: 1000 transitions using 10 leapfrog steps per transition would take 0 seconds.
Chain 2: Adjust your expectations accordingly!
Chain 2:
Chain 2:
Chain 2: Iteration:    1 / 2000 [  0%]  (Warmup)
```

```
Chain 2: Iteration:  200 / 2000 [ 10%]  (Warmup)
Chain 2: Iteration:  400 / 2000 [ 20%]  (Warmup)
Chain 2: Iteration:  600 / 2000 [ 30%]  (Warmup)
Chain 2: Iteration:  800 / 2000 [ 40%]  (Warmup)
Chain 2: Iteration: 1000 / 2000 [ 50%]  (Warmup)
Chain 2: Iteration: 1001 / 2000 [ 50%]  (Sampling)
Chain 2: Iteration: 1200 / 2000 [ 60%]  (Sampling)
Chain 2: Iteration: 1400 / 2000 [ 70%]  (Sampling)
Chain 2: Iteration: 1600 / 2000 [ 80%]  (Sampling)
Chain 2: Iteration: 1800 / 2000 [ 90%]  (Sampling)
Chain 2: Iteration: 2000 / 2000 [100%]  (Sampling)
Chain 2:
Chain 2:  Elapsed Time: 7.714 seconds (Warm-up)
Chain 2:                5.138 seconds (Sampling)
Chain 2:                12.852 seconds (Total)
Chain 2:

SAMPLING FOR MODEL 'count' NOW (CHAIN 3).
Chain 3:
Chain 3: Gradient evaluation took 0 seconds
Chain 3: 1000 transitions using 10 leapfrog steps per transition would take 0 seconds.
Chain 3: Adjust your expectations accordingly!
Chain 3:
Chain 3:
Chain 3: Iteration:    1 / 2000 [  0%]  (Warmup)
Chain 3: Iteration:  200 / 2000 [ 10%]  (Warmup)
Chain 3: Iteration:  400 / 2000 [ 20%]  (Warmup)
Chain 3: Iteration:  600 / 2000 [ 30%]  (Warmup)
Chain 3: Iteration:  800 / 2000 [ 40%]  (Warmup)
Chain 3: Iteration: 1000 / 2000 [ 50%]  (Warmup)
Chain 3: Iteration: 1001 / 2000 [ 50%]  (Sampling)
Chain 3: Iteration: 1200 / 2000 [ 60%]  (Sampling)
Chain 3: Iteration: 1400 / 2000 [ 70%]  (Sampling)
Chain 3: Iteration: 1600 / 2000 [ 80%]  (Sampling)
Chain 3: Iteration: 1800 / 2000 [ 90%]  (Sampling)
Chain 3: Iteration: 2000 / 2000 [100%]  (Sampling)
Chain 3:
Chain 3:  Elapsed Time: 7.2 seconds (Warm-up)
Chain 3:                4.379 seconds (Sampling)
Chain 3:                11.579 seconds (Total)
Chain 3:

SAMPLING FOR MODEL 'count' NOW (CHAIN 4).
Chain 4:
Chain 4: Gradient evaluation took 0 seconds
Chain 4: 1000 transitions using 10 leapfrog steps per transition would take 0 seconds.
Chain 4: Adjust your expectations accordingly!
Chain 4:
Chain 4:
Chain 4: Iteration:    1 / 2000 [  0%]  (Warmup)
Chain 4: Iteration:  200 / 2000 [ 10%]  (Warmup)
Chain 4: Iteration:  400 / 2000 [ 20%]  (Warmup)
Chain 4: Iteration:  600 / 2000 [ 30%]  (Warmup)
Chain 4: Iteration:  800 / 2000 [ 40%]  (Warmup)
```

```
Chain 4: Iteration: 1000 / 2000 [ 50%]  (Warmup)
Chain 4: Iteration: 1001 / 2000 [ 50%]  (Sampling)
Chain 4: Iteration: 1200 / 2000 [ 60%]  (Sampling)
Chain 4: Iteration: 1400 / 2000 [ 70%]  (Sampling)
Chain 4: Iteration: 1600 / 2000 [ 80%]  (Sampling)
Chain 4: Iteration: 1800 / 2000 [ 90%]  (Sampling)
Chain 4: Iteration: 2000 / 2000 [100%]  (Sampling)
Chain 4:
Chain 4:  Elapsed Time: 7.766 seconds (Warm-up)
Chain 4:                8.867 seconds (Sampling)
Chain 4:                16.633 seconds (Total)
Chain 4:
```

```
Warning: There were 1 divergent transitions after warmup. See
http://mc-stan.org/misc/warnings.html#divergent-transitions-after-warmup
to find out why this is a problem and how to eliminate them.
```

```
Warning: Examine the pairs() plot to diagnose sampling problems
```

```
##
## Takes about 1 minute to fit
##
# summary(stan.nb.1)
```

Let's first inspect the estimated effect of "AREA".

```
options(digits = 5)
fixef(stan.nb.1)["AREANon-intervention"]
```

```
AREANon-intervention
              1.4234
```

```
exp(fixef(stan.nb.1)["AREANon-intervention"])
```

```
AREANon-intervention
              4.1513
```

```
##
# summary(stan.nb.1,
#         pars = "AREANon-intervention",
#         digits = 4)
```

The Bayesian method estimate the effect to be larger than the (approximate) Likelihood approach. So, this analysis adds evidence that there a strong difference in the two areas.

Let's look at the "Bayesian posterior uncertainty intervals" associated with the "AREA" coefficient.

```
posterior_interval(stan.nb.1,
                   pars = "AREANon-intervention")
```

```
                          5%    95%
AREANon-intervention 0.96258 1.9251
```

```
##
exp(posterior_interval(stan.nb.1,
                       pars = "AREANon-intervention"))
```

```
                         5%    95%
AREANon-intervention 2.6184 6.8561
```

So, there is clear evidence that this effect differs from zero.

Let's look at the random effects estimates and the associated intervals.

```
summary(stan.nb.1,
        pars = c("Sigma[TRAP.ID.fac:(Intercept),(Intercept)]",
                 "Sigma[MUNICIPALITY:(Intercept),(Intercept)]"),
        digits = 4)
```

```
Model Info:
 function:     stan_glmer.nb
 family:       neg_binomial_2 [log]
 formula:      No..eggs.AEDES ~ AREA + poly(Day.ovitrap.collected, degree = 2) +
       scale(ALTITUDE) + No..Days.ovitrap.in.field + (1 | TRAP.ID.fac) +
       (1 | MUNICIPALITY)
 algorithm:    sampling
 sample:       4000 (posterior sample size)
 priors:       see help('prior_summary')
 observations: 327
 groups:       TRAP.ID.fac (36), MUNICIPALITY (6)

Estimates:
                                            mean   sd     10%    50%    90%
Sigma[TRAP.ID.fac:(Intercept),(Intercept)]  0.2719 0.1293 0.1261 0.2522 0.4422
Sigma[MUNICIPALITY:(Intercept),(Intercept)] 0.0653 0.1705 0.0005 0.0192 0.1609

MCMC diagnostics
                                            mcse   Rhat   n_eff
Sigma[TRAP.ID.fac:(Intercept),(Intercept)]  0.0035 0.9993 1383
Sigma[MUNICIPALITY:(Intercept),(Intercept)] 0.0039 1.0035 1870

For each parameter, mcse is Monte Carlo standard error, n_eff is a crude measure of effective sample si
```

Let's represent these a posteriori distribution graphically.

```
plot(stan.nb.1,
     pars = c("Sigma[TRAP.ID.fac:(Intercept),(Intercept)]",
              "Sigma[MUNICIPALITY:(Intercept),(Intercept)]"))
```

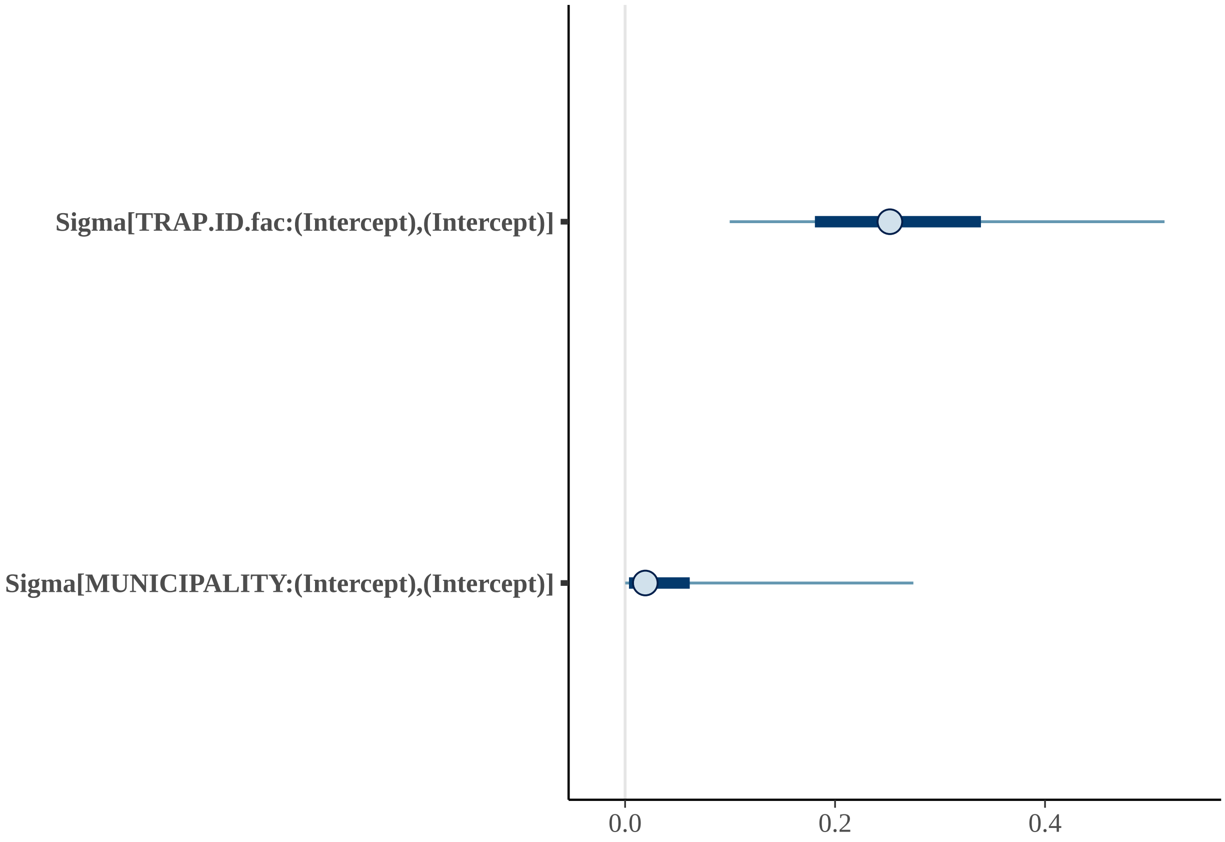

These results confirm the ones obtained with the *glmmTMB()* function. Municipality play a much smaller role and its RE is close to zero.

To summarise, these results **the Bayesian approach confirms that there is biologically relevant difference between "intervention" and "Non-intervention".** In particular, the Bayesian model suggests that there are about 4 times more eggs in the "Non-intervention" area.

10 Session Information

```
sessionInfo()
```

```
R version 4.1.0 (2021-05-18)
Platform: x86_64-w64-mingw32/x64 (64-bit)
Running under: Windows 10 x64 (build 19043)

Matrix products: default

locale:
[1] LC_COLLATE=English_Switzerland.1252  LC_CTYPE=English_Switzerland.1252
[3] LC_MONETARY=English_Switzerland.1252 LC_NUMERIC=C
[5] LC_TIME=English_Switzerland.1252

attached base packages:
[1] stats     graphics  grDevices utils     datasets  methods   base
```

```
other attached packages:
 [1] rstanarm_2.21.1   Rcpp_1.0.6        multcomp_1.4-17   TH.data_1.0-10   
 [5] MASS_7.3-54       survival_3.2-11   mvtnorm_1.1-1     geoR_1.8-1       
 [9] sp_1.4-5          gridExtra_2.3     lubridate_1.7.10 glmmTMB_1.0.2.1  
[13] ggplot2_3.3.3     lattice_0.20-44   dplyr_1.0.6       checkpoint_0.4.10
[17] knitr_1.33       

loaded via a namespace (and not attached):
 [1] minqa_1.2.4             colorspace_2.0-1        ellipsis_0.3.2         
 [4] ggridges_0.5.3          rsconnect_0.8.18        markdown_1.1           
 [7] base64enc_0.1-3         rstudioapi_0.13         farver_2.1.0           
[10] rstan_2.21.2            DT_0.18                 fansi_0.5.0            
[13] codetools_0.2-18        splines_4.1.0           shinythemes_1.2.0      
[16] bayesplot_1.8.0         jsonlite_1.7.2          nloptr_1.2.2.2         
[19] shiny_1.6.0             compiler_4.1.0          Matrix_1.3-3           
[22] fastmap_1.1.0           cli_2.5.0               later_1.2.0            
[25] htmltools_0.5.1.1       prettyunits_1.1.1       tools_4.1.0            
[28] igraph_1.2.6            gtable_0.3.0            glue_1.4.2             
[31] reshape2_1.4.4          V8_3.4.2                vctrs_0.3.8            
[34] nlme_3.1-152            crosstalk_1.1.1         xfun_0.23              
[37] stringr_1.4.0           ps_1.6.0                lme4_1.1-27            
[40] mime_0.10               miniUI_0.1.1.1          lifecycle_1.0.0        
[43] gtools_3.8.2            zoo_1.8-9               scales_1.1.1           
[46] colourpicker_1.1.0      promises_1.2.0.1        parallel_4.1.0         
[49] sandwich_3.0-0          inline_0.3.18           shinystan_2.5.0        
[52] TMB_1.7.20              yaml_2.2.1              curl_4.3.1             
[55] loo_2.4.1               StanHeaders_2.21.0-7    stringi_1.6.1          
[58] dygraphs_1.1.1.6        RandomFieldsUtils_0.5.3 boot_1.3-28            
[61] pkgbuild_1.2.0          rlang_0.4.11            pkgconfig_2.0.3        
[64] matrixStats_0.58.0      evaluate_0.14           purrr_0.3.4            
[67] rstantools_2.1.1        htmlwidgets_1.5.3       labeling_0.4.2         
[70] tidyselect_1.1.1        processx_3.5.2          plyr_1.8.6             
[73] magrittr_2.0.1          R6_2.5.0                generics_0.1.0         
[76] mgcv_1.8-35             pillar_1.6.1            withr_2.4.2            
[79] xts_0.12.1              tibble_3.1.2            crayon_1.4.1           
[82] utf8_1.2.1              rmarkdown_2.8           RandomFields_3.3.8     
[85] grid_4.1.0              callr_3.7.0             splancs_2.01-42        
[88] threejs_0.3.3           digest_0.6.27           xtable_1.8-4           
[91] httpuv_1.6.1            RcppParallel_5.1.4      stats4_4.1.0           
[94] munsell_0.5.0           tcltk_4.1.0             shinyjs_2.0.0          
```
