# Supplementary material for: Effectiveness of integrated Aedes albopictus management in southern Switzerland
Source: Parasit Vectors. 2021 Aug 16;14:405. doi: 10.1186/s13071-021-04903-2 (PMC8365973; doi:10.1186/s13071-021-04903-2)
Supplement: Supplementary file 15 — Additional file 15: Figure S2. Study area in the 2012 and 2013 evaluation of the Ticino intervention programme [14]. The map was prepared using the geographic information system (GIS) software ArcGIS version 10.0 (ESRI Inc., USA). [file 13071_2021_4903_MOESM15_ESM.pdf]

Additional file 15 Fig. S2. Study area in the 2012 and 2013 evaluation of the Ticino intervention programme [14].

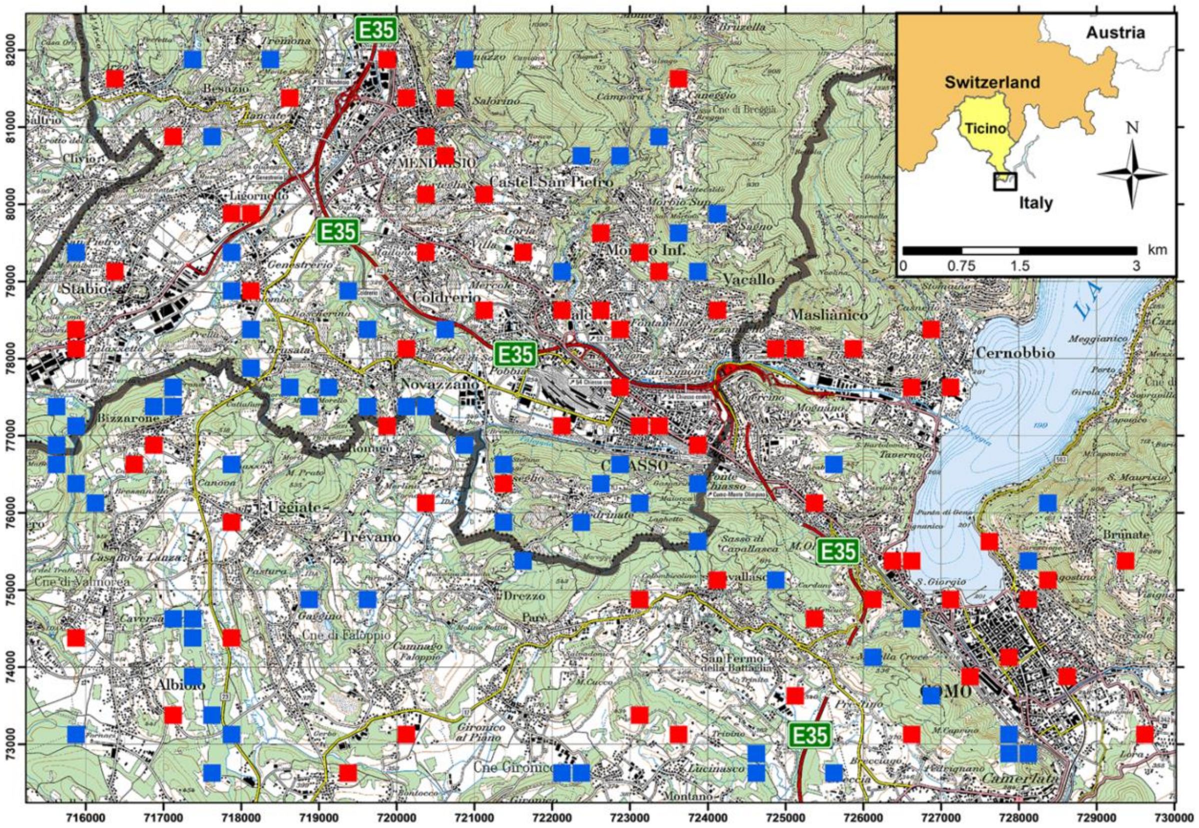

Study area and ovitrap positions. The red and blue squares represent sampling grid cells in urban (red) and sylvatic (blue) environments. In each country 35 grid cells were randomly allocated to either the urban or sylvatic environment. Within selected grid cells two ovitraps were placed at a minimum distance of 50 m between them to avoid interference in mosquito attraction. In total, there were 280 ovitraps (2 countries x 2 environments x 35 cells x 2 ovitraps). The thick grey line denotes the Swiss-Italian border with the intervention area (Ticino, Switzerland) in the North and the non-intervention area (Lombardy, Italy) in the South. The orange line, crossing the Swiss-Italian border, shows the European route E35. The numbers at the left and at the bottom indicate the Swiss km co-ordinates. The map was prepared using the geographic information system (GIS) software ArcGIS version 10.0 (ESRI Inc., USA).
